# Comparative structural profiling of trichome specialized metabolites in tomato (*Solanumlycopersicum*) and *S. habrochaites*: acylsugar profiles revealed by UHPLC/MS and NMR

**DOI:** 10.1007/s11306-013-0585-y

**Published:** 2013-09-19

**Authors:** Banibrata Ghosh, Thomas C. Westbrook, A. Daniel Jones

**Affiliations:** 1 Department of Biochemistry and Molecular Biology, Michigan State University, 603 Wilson Road, Biochemistry Building, Room 212, East Lansing, MI 48824 USA; 2 Department of Chemistry, Michigan State University, East Lansing, MI 48824 USA

**Keywords:** Acylsugars, Glandular trichomes, Comparative profiling, Collision-induced dissociation, Metabolite identification

## Abstract

Many plants accumulate large quantities of specialized metabolites in secretory glandular trichomes (SGTs), which are specialized epidermal cells. In the genus *Solanum*, SGTs store a diverse collection of glucose and sucrose esters. Profiling of extracts from two accessions (LA1777 and LA1392) of *Solanum habrochaites* using ultra-high performance liquid chromatography–mass spectrometry (UHPLC/MS) revealed wide acylsugar diversity, with up to 11 isomers annotated for each individual elemental formula. These isomers arise from differences in ester chain lengths and their positions of substitution or branching. Since fragment ion masses were not sufficient to distinguish all isomers, 24 acylsucroses were purified from *S. habrochaites* accessions and cultivated tomato (*Solanum lycopersicum* M82) and characterized using NMR spectroscopy. Two-dimensional NMR spectra yielded assignments of positions of substitution of specific acyl groups, and locations of branching. The range of substitution was wider than reported earlier, and in contrast to previous reports, tetra- and penta-acylsucroses were substituted at position 2 with acyl groups other than acetate. Because UHPLC/MS fails to yield sufficient information about structure diversity, and quantitative NMR of acylsugar mixtures is confounded by structural redundancy, the strategic combination of NMR and UHPLC/MS provides a powerful approach for profiling a class of metabolites with great structural diversity across genotypes.

**Electronic supplementary material:**

The online version of this article (doi:10.1007/s11306-013-0585-y) contains supplementary material, which is available to authorized users.

## Introduction

The plant kingdom is estimated to synthesize a phenomenally diverse suite of bioactive specialized metabolites (Crozier et al. 2006), many of which serve as bioactive chemical defenses and attractants for pollinators (Waterman 1992). Humans have long valued these substances as medicines, flavors, and fragrances (Balandrin et al. 1985), and they provide a mechanism for engineering crops for resistance to insects and disease (Gasser and Fraley 1989). The diversity of specialized plant metabolites has been attributed to gene duplication, domain swapping, and mutation, which can lead to diversification of biosynthetic enzyme functions and accumulation of multiple chemical variants within structural classes of metabolites including terpenes (Pichersky and gang 2000).

In the plant family Solanaceae, which encompasses tomato, potato, peppers, tobacco, nightshade, and petunia, specialized acylsugar metabolites accumulate in specific epidermal cells known as secretory glandular trichomes (SGTs) (McDowell et al. 2011; Schilmiller et al. 2008; Wagner 1991). SGTs are hair-like epidermal outgrowths, normally found on plant leaves and stems, and their accessibility facilitates investigation of gene expression and chemistry of individual cell types. These metabolites are believed to have important biological functions including plants’ self-defense against insects and herbivores (Stipanovic 1983; Weinhold and Baldwin 2011). Among other important bioactive compounds synthesized/stored in SGTs in species across the Solanaceae are acylsugars (Slocombe et al. 2008), which are non-volatile metabolites that constitute a significant proportion of leaf biomass in some Solanaceous species (Fobes et al. 1985). Acylsugars exhibit insecticidal properties (Chortyk et al. 1996) and also have potential as food-grade fat substitute (Farooq and Haque 1992), emulsifiers in food products (Garti et al. 2000), surfactants (Hill and Rhode 1999) and antibiotics (Chortyk et al. 1993). In *Solanum* tissues, known acylsugars are based on glucose or sucrose cores esterified to 2–5 straight or branched chain aliphatic acids each with 2–12 carbon atoms (Arrendale et al. 1990; Ghosh et al. 2011; King et al. 1990, 1993).

Current understanding of how new metabolic pathways and networks evolve and yield differences in plant tissue chemistry is not yet deep, but advances in genomics when combined with improved definition of chemical phenotypes promise to accelerate advances in this area (Kliebenstein and Osbourn 2012). In tomato and its wild relatives, discoveries of the genes and pathways responsible for acylsugar biosynthesis are an active area of research (Schilmiller et al. 2012; Kim et al. 2012), and stand to benefit from improved assessment of acylsugar phenotypes among different *Solanum* genotypes. Definition of acylsugar phenotypes has relied on LC/MS analyses (Schilmiller et al. 2010, 2012; Kim et al. 2012) and NMR profiling (Mirhezhad et al. 2010) of tissue extracts, but many knowledge gaps remain including structures of acylsugar metabolites. Knowledge of *Solanum* acylsugar structures is essential to define roles of biosynthetic enzymes in modification of specific positions of the sugar core by individual acyl groups. At present, such information is limited to acylsugar structures reported in earlier studies, though many of these are acylglucoses derived from the tomato relative *Solanum pennellii* and more distant relatives including petunia and tuberous *Solanum* species (Burke et al. 1987; King et al. 1988; Singh et al. 2003). Structures of a small number (~10) of acylsucroses from tomato and its close relatives have been reported (King et al. 1990; Schilmiller et al. 2010), and have shown acyl substitution distributed over the pyranose ring (at the 2, 3, 4, and 6 positions) and the furanose (1′ and 3′ positions), but the scarcity of metabolite structures has hindered assessment of their structural diversity. Different *Solanum* genotypes exhibit substantial acylsugar diversity (both qualitative and quantitative) underscoring potential differences in functions of their expressed biosynthetic enzymes as well as pools of available precursors (Kim et al. 2012). In light of these findings, definition of acylsugar structures and assessments of differences in acylsugar diversity will aid discovery of new genes and pathways, as demonstrated in the assignment of function to acyltransferase enzymes involved in their formation (Schilmiller et al. 2012; Kim et al. 2012). Discovery of such genes will open doors to metabolic engineering of cultivated tomato and other species for controlled productions of beneficial metabolites (Stephanopoulos 1999).

Much of our current effort aims to define metabolic phenotypes of glandular trichomes of tomato and its wild relatives so they may be compared across genotypes. Phenotyping has relied on mass spectrometric analyses, milligram-scale purification, and NMR characterization of purified metabolites. Mass spectra alone have not distinguished isomers differing in positions of acyl substitution or in acyl chain branching. To resolve such structural ambiguities, we demonstrate a combination of ultra-high performance liquid chromatography–mass spectrometry (UHPLC/MS) and NMR for comparative structural profiling of acylsugar metabolites from leaves from *Solanum habrochaites* accessions LA1777 and LA1392 and cultivated tomato *Solanum lycopersicum* M82 (LA3475).

## Materials and methods

### General

The plants were germinated from seeds obtained from the C. M. Rick Tomato Genetics Resource Center (University of California-Davis, CA,USA). Plants were grown in a growth chamber at 28 °C and 86 % relative humidity. Lights in the growth chamber were on for 17 h and off for 7 h during each day. Additional plant metadata are included in supplementary material. All plants used for metabolite profiling were grown from cuttings, and tissues were harvested for metabolite extractions 18 days after cuttings were taken. All plants used for purification of acylsugars were between 6–10 months old post-germination when tissues were harvested. These plants were also grown from seed in the same growth chamber at 28 °C and 86 % relative humidity for 6 weeks, and were then transferred to a laboratory windowsill with ample sunlight after 6 weeks to accumulate sufficient tissue for metabolite isolation. ^1^H NMR spectra were recorded using an Avance 900 (at 900 MHz) NMR spectrometer (Bruker) and ^13^C NMR spectra were recorded at 225 MHz on the same spectrometer at the Michigan State University Max T. Rogers NMR Facility. Metabolite purification was performed using either a Model 680 Waters Gradient Controller coupled with a Model 512 HPLC pump or a Waters Model 2795 HPLC system. Additional details regarding purification are in supplementary material. All solvents were HPLC grade. Chemical shifts are reported relative to peaks of solvent CD_3_OD (δ = 3.31 ppm for ^1^H and 49.1 ppm for ^13^C), CDCl_3_ (δ = 7.24 ppm for ^1^H and 77.0 ppm for ^13^C) and CD_3_CN (δ = 1.94 ppm for ^1^H and 118.26 ppm for ^13^C).

### UHPLC/MS analyses of* S. habrochaites* and cultivated tomato acylsugars

Ten leaflets from the node adjacent to the apical tissue from of each of *S. habrochaites* accessions LA1777, LA1392, and cultivated tomato (*S. lycopersicum* M82) were harvested by cutting the petioles at the stem. Leaflets ranged from 25–75 % of mature leaflet size. Leaflets were immediately dipped into 10 mL of methanol separately for each plant for 2 min. Each extract was quantitatively transferred to a 15-mL polypropylene centrifuge tube, and solvent was evaporated to dryness under nitrogen. Residues were redissolved by adding 0.5 mL acetonitrile/water (4/1 v/v) to each tube followed by vortexing for two minutes. These solutions were centrifuged at 25 °C and 2,627×*g* for 10 min. Aliquots (200 μL) of each supernatant were transferred into 250-μL glass inserts placed in 2 mL HPLC vials. These were used directly for UHPLC/MS analyses.

UHPLC/MS analyses were performed using a Shimadzu LC-20AD ternary pump coupled to a SIL-5000 autosampler, column oven, and Waters LCT Premier Mass Spectrometer. Separations were performed using an Ascentis Express C18 Analytical HPLC column (2.1 × 100 mm, 2.7 μm). The mobile phase consisted of aqueous 10 mM ammonium formate, pH 2.64 (Solvent A) and acetonitrile (Solvent B) using a linear gradient elution of 1 % B at 0–1 min, 1–80 % B at 1–100 min, 80–100 % B at 100–101 min, 100 % B at 101–105 min and 1 % B at 105–106 min. A 4 min re-equilibration time was used between analyses. The solvent flow rate was 0.3 mL/min and the column temperature was 40 °C. Analyses were performed using W optics (resolution 9000) in both positive and negative ion modes. Source parameters were as follows: capillary voltage 2,500 V, sample cone voltage 10 V, desolvation temperature 350 °C, source temperature 100 °C, cone gas flow 40 L/h and desolvation gas flow 350 L/h for the negative ion mode. For positive ion mode the capillary voltage was set to 3,000 V with other parameters unchanged. Mass spectrum acquisition was performed from *m/z* 50 to 1,500 in both the positive and negative ion modes with a scan time of 0.1 s. The fragment ions were obtained by rapid switching of aperture 1 voltage in five increments (10, 20, 40, 60 and 80 V) in the ion transit region of the mass spectrometer, providing quasi-simultaneous generation of spectra under fragmenting and non-fragmenting conditions, with data collected in separate acquisition functions for each collision voltage. Spectra were acquired in centroid format using ‘dynamic range enhancement’ (DRE).

### Purification of acylsugar metabolites

About 100–130 leaflets of individual plants of *S. habrochaites* LA1777, LA1392, and LA1362 (only used for metabolite purification) and *S. lycopersicum* M82 were harvested and placed in a 1 L beaker. Methanol (500–1,000 mL) was added and the mixture was stirred with a glass rod for 2 min. The mixture was quickly transferred into a 1 L glass bottle through filter paper using a Buchner funnel. Solvent was evaporated to dryness under vacuum using rotary evaporation, and the residue was re-dissolved in 3 mL of acetonitrile: water (4/1 v/v) using ultrasonication for 10 min followed by centrifugation at 2,627×*g* for 2 min at 25 °C. Supernatants were collected and transferred to autosampler vials. Purification was performed using Waters Automated Gradient Controller (Model 680) coupled with Waters HPLC system (Model 512) and a Dionex Acclaim 120 C18 HPLC column (4.6 × 150 mm, 5 μm). Eluted fractions were collected in a LKB fraction collector in 1-min fractions for 10–15 injections, using an injection volume of 150 μL for each injection. Additional details regarding individual acylsugar purification, NMR spectra, and structure elucidation are in supplementary material.

## Results and discussion

Aside from a few reports of acylsugar metabolite structures (King et al. 1990, 1993; Schilmiller et al. 2010), the majority of acylsucrose metabolites in the genus *Solanum* have not been fully characterized before. In this investigation, 24 acylsucroses were purified and subjected to structure elucidation using UHPLC/MS and NMR analyses, and to our knowledge, 21 of these are novel compounds. A summary of the metabolite identities is presented in Table 1, which contains UHPLC retention times and high resolution mass measurements, and in the supplementary material, the latter of which contains details regarding InChI key identifications, NMR chemical shift assignments, and the complete set of 1D and 2D NMR spectra. Given that most of these are novel metabolites, their identifications should be considered as meeting Metabolomics Standards Initiative level 1 criteria because connectivities have been established based on the NMR and mass spectrometric characterization described herein (Sumner et al. 2007).

**Table 1:** NMR-elucidated structures of acylsugars purified from leaf dip extracts of *S. habrochaites* LA1777, LA1392, LA1362, and M82 including accurate mass data and UHPLC retention times for each isomer

Acylsugar^a^	Retention time (min)	Experimental *m/z* of [M + formate]^−^	Theoretical *m/z* of [M + formate]^−^	R_2_	R_3_	R_4_	R_1′_	R_3′_	R_6′_	Metabolite abundances		
										*S. lycopersicu*m M82	*S. habrochait*es LA1392	*S. habrochait*es LA1777
Triacylsucroses (7)												
S3:19[5]^b^	63.30	695.3575	695.34956	H	iC4	iC5	H	iC10	H	+	++	+++
S3:19[9]^b^	65.79	695.3550	695.34956	iC4	iC10	iC5	H	H	H	ND	++	ND
S3:20[4]^b^	68.82	709.3716	709.36521	H	aiC5	iC5	H	iC10	H	+	+++	ND
S3:21[1]^b^	70.63	723.3868	723.38086	H	aiC5	iC5	H	aiC11	H	ND	++	+
S3:21[5]^b^	72.75	723.3917	723.38086	iC5	aiC11	iC5	H	H	H	+	++	++
S3:22[4]^b^	77.89	737.3903	737.39651	H	nC12	iC5	H	iC5	H	++	+++	++
S3:22[5]^b^	78.88	737.4048	737.39651	aiC5	nC12	iC5	H	H	H	ND	+++	+
Tetraacylsucroses (15)												
S4:16[3]^b^	49.31	667.2884	667.28187	C2	iC4	iC5	H	iC5	H	++	++	+++
S4:17[2]^b,d^	52.86	681.3039	681.29752	C2	aiC5	iC5	H	iC5	H	+++	+++	+++
S4:19[7]^b^	60.82	709.3358	709.32882	iC4	iC5	iC5	H	H	iC5	ND	++	++
S4:20[6]^b^	64.58	723.3523	723.34447	iC5	iC5	iC5	H	H	iC5	ND	+++	+
S4:20[7]^d^	65.47	723.3598	723.34447	C2	iC4	iC4	H	iC10	H	++	++	+++
S4:21[2]^d^	69.00	737.3712	737.36012	C2	iC4	iC5	H	iC10	H	++	+++	+++
S4:22[2]^d^	72.54	751.3755	751.37577	C2	aiC5	iC5	H	iC10	H	+	+++	+++
S4:22[3]^b^	72.83	751.3834	751.37577	C2	iC4	iC5	H	aiC11	H	ND	++	++
S4:22[6]^d^	74.98	751.3832	751.37577	C2	iC4	iC4	H	nC12	H	+	+	+++
S4:23[3]^b^	76.41	765.4035	765.39142	C2	aiC5	iC5	H	aiC11	H	ND	++	++
S4:23[5]^b^	77.52	765.4016	765.39142	C2	iC4	iC5	H	iC12	H	+	++	++
S4:23[6]^d^	78.56	765.3979	765.39142	C2	iC4	iC5	H	nC12	H	+	++	+++
S4:24[5]^d^	81.19	779.4138	779.40707	C2	aiC5	iC5	H	iC12	H	+	++	++
S4:24[6]^d^	82.19	779.4132	779.40707	C2	aiC5	iC5	H	nC12	H	+	++	++
S4:24[8]^d^	84.06	779.4109	779.40707	C2	nC12	iC5	H	iC5	H	++	++	++
Pentaacylsucroses (2)												
S5:24[3]^b,e^	77.76	793.3953	793.38634	aiC5	iC4	iC5	iC5	H	iC5	ND	+++	++
S5:25[4]^b,e^	81.42	807.4112	807.40199	iC5	iC5	iC5	iC5	H	iC5	ND	+++	++

Abbreviations used for acylsugar annotations are given in the text. Numbers in parentheses indicate relative order of elution of the corresponding isomer (isomer number as described in Fig. 4). The relative abundance indicators were generated by using the formula Relative Abundance (X) = (peak area * 100/dry weight). Indicators +++, ++, + and ND were used when X > 1000, 1000 > X > 100, 100 > X > 1 and X < 1 respectively. Purified acylsugars were matched in the various accessions based on their retention times, molecular and fragment masses and CID fragmentation behavior. ND = not detected

^a^ **C2** = acetate, **iC4** = (CH_3_)_2_CHCO, **iC5** = (CH_3_)_2_CHCH_2_CO, **aiC5** = CH_3_CH_2_(CH_3_)CHCO, **iC10** = (CH_3_)_2_CH(CH_2_)_6_CO, **aiC11** = CH_3_CH_2_(CH_3_)CH(CH_2_)_6_CO, **iC12** = (CH_3_)_2_CH(CH_2_)_8_CO, **nC12** = CH_3_(CH_2_)_9_CO

^b^Reference metabolite purified from *S. habrochaites* LA1392

^c^Reference metabolite purified from *S. lycopersicum* M82

^d^Reference metabolite purified from *S. habrochaites* LA1777

^e^Reference metabolite purified from *S. habrochaites* LA1362

To simplify the nomenclature of acylsugars, we use a single letter to define the sugar core (“S” for sucrose and “G” for glucose) followed by a number indicating the total number of acyl groups attached to the sugar core. This number is followed by a colon and another number designating total number of carbon atoms in all attached acyl groups (Schilmiller et al. 2010). The numbers of carbon atoms in individual acyl groups are included in parentheses in ascending order separated by commas. For example, a sucrose tetraester having an acetate, C4, C5 and C10 acyl groups will be annotated as S4:21(2,4,5,10). To distinguish isomeric acylsugars, particularly when complete structures are not yet available, a number is included in brackets (e.g. S4:21[2]) that designates the relative order of chromatographic elution within each set of isomeric acylsugars. Differences in acyl group branching are designated by using the abbreviations ‘n’ for straight chain, ‘ai’ for ante-iso and ‘i’ for iso branching when the branching is known.

### Acylsugar profiling using UHPLC/MS

**Fig. 1:**
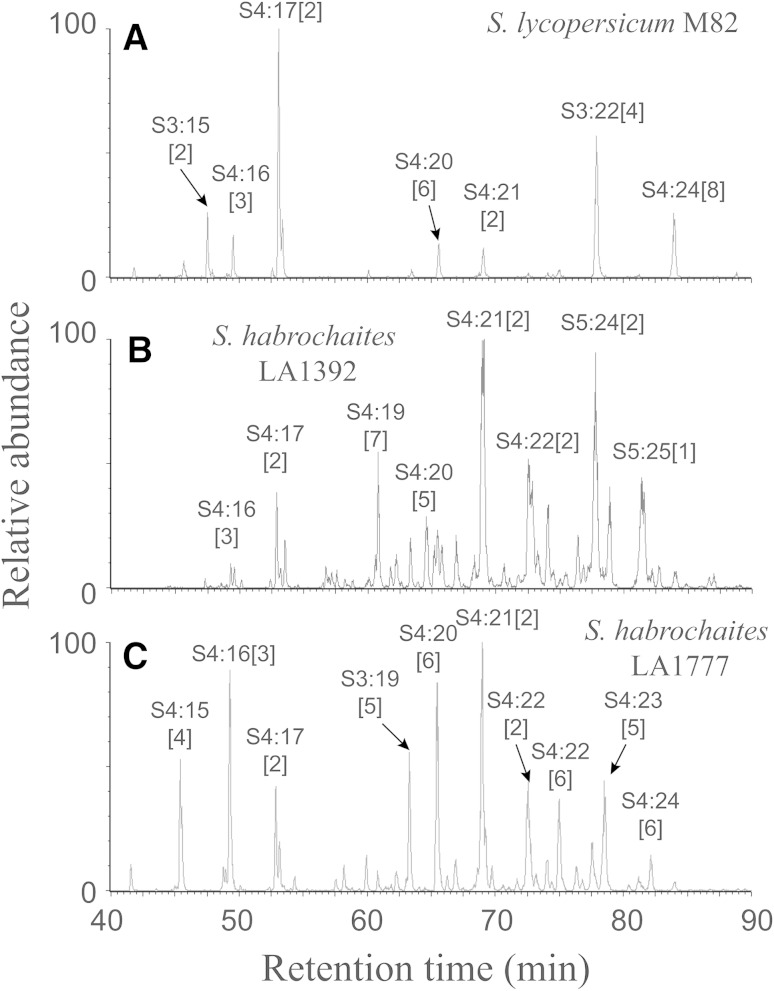
Extracted ion UHPLC/MS chromatograms (summation of signals for *m/z* 639, 653, 667, 681, 695, 709, 723, 737, 751, 765, 779, 793, 807) of leaf extracts of (**a**) cultivated tomato *S. lycopersicum* M82 and *S. habrochaites* accessions LA1392 and LA1777 (**b** and **c** respectively), showing trichome-derived acylsugars (formate adducts) at aperture 1 voltage = 10 V and negative ion mode electrospray ionization. Abbreviations used for acylsugar annotation are as described in the text. The number in the square brackets next to the acylsugar nomenclature designates each isomer’s chromatographic elution order

**Fig. 2:**
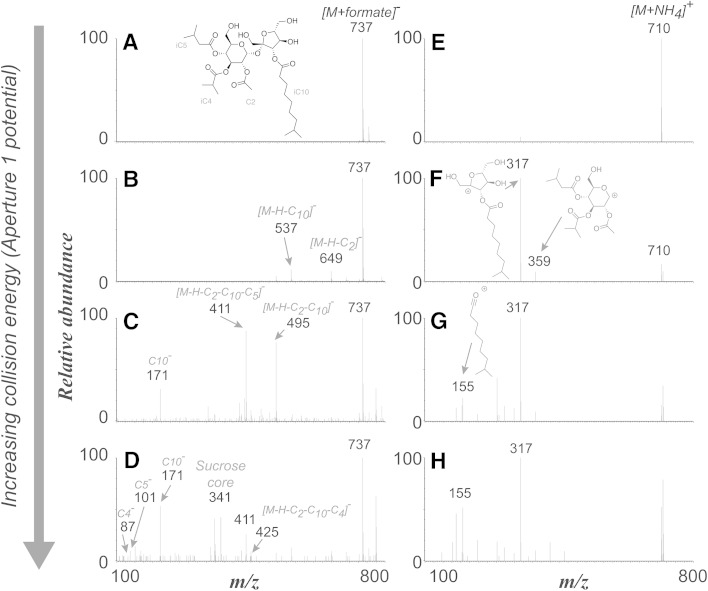
Multiplexed CID mass spectra of acylsucrose S4:21[2] (2,4,5,10) from *S. habrochaites* LA1777 using negative and positive mode electrospray ionization. ESI (−) aided assignment of the acyl groups attached to the sucrose core (acetate, C4, C5 and C10) whereas ESI (+) showed that acetate, C4 and C5 are on pyranose (*m/z* 359 fragment) and C10 is on the furanose ring of the sucrose (*m/z* 317 fragment) respectively. **a**–**d** and **e**–**h** indicate Aperture 1 potential of 10, 40, 60 and 80 volts using negative and positive mode respectively

When methanolic extracts of leaflets from two *S. habrochaites* accessions (LA1777 and LA1392) and tomato (*S. lycopersicum* M82) were subjected to UHPLC/MS analysis, chromatographic peaks corresponding to 82 sucrose tetraesters, 34 sucrose triesters and 13 sucrose pentaesters were annotated in the combined UHPLC/MS data set encompassing all three accessions (Fig. 1). No acylglucoses were detected in any of these accessions. Acylsugars were annotated through a combination of pseudomolecular and fragment ion masses, the latter being generated by CID. In the negative-ion mass spectra, the formate adduct was the most abundant ion formed for all acylsugars at the lowest CID potential (10 V). At the highest CID potential (80 V), the individual acid anions (e.g. *m/z* 87 for iC4 carboxylate, *m/z* 101 for C5) in these acylsugars became apparent. Between these two extremes (20, 40 and 60 V), fragment ions corresponding to [M−H]^−^ and successive neutral losses of the ester groups as ketenes (e.g. 84 Da for C5 ketenes) reached their maximum relative abundances. Information in the negative mode CID spectra alone made it possible to assign the number of carbon atoms to each ester group. In contrast, the positive mode CID spectra provided complementary information about the corresponding acylsugar structures. At the lowest CID voltage, ammonium adducts ([M+NH_4_]^+^) of the acylsugars were the most abundant ions. However, as the CID potential was increased, fragment ions formed from cleavage of the glycosidic bond between the pyranose and furanose ring of sucrose, and their masses indicated the mass shifts caused by acyl substitutions on glucopyranose and fructofuranose rings. The dominant positive ion fragment usually arises from cleavage of the glycosidic bond, with retention of charge on the 5-membered furanose ring. The mass of this fragment indicates the substitutions of acyl groups on the furanose ring (positions 1′ to 6′), and the mass of a less abundant fragment derived from the pyranose ring indicates the total substituents attached to positions 2–6. Figure 2 illustrates how negative- and positive-mode ESI spectra of individual acylsugars aided annotation of acyl groups and their distribution on the hexose rings. When isomerism of the acylsugars was a result of esterification by acids with different chain lengths, CID spectra distinguished these isomers in the form of fragment ions generated by neutral losses of ketenes (e.g. 84 Da neutral loss for C5) and from carboxylate anion masses such as *m/z* 171 for the C10 anion). However, CID spectra did not provide information about specific positions of substitution or branching of these acyl groups because no cross-ring fragment or acyl chain fragment ions were observed.

Examination of the masses of the pseudomolecular ions and coincident fragment ions generated using multiplexed CID conditions revealed more isomers than were observed in a recent report of *S. habrochaites* acylsugars (Kim et al. 2012) owing to the use of a longer column and 110-min gradient elution. Multiple isomers were evident from multiple chromatographic peaks with matching masses of formate adducts [M+HCOO]^−^ in negative mode, and ammonium adduct [M+NH_4_]^+^ in positive mode. Mass spectra often fail to distinguish differences in positions of acyl group substitution or branching isomers because distinguishing fragment ions are not formed. In some cases, differences in masses of observed fragment ions indicate that isomers arise from differences in chain lengths of acyl groups. When the fragment ions have the same masses for different chromatographic peaks, the results require that isomerism is based on differences in positions of acyl group substitution and/or differences in branching positions within acyl groups.

### Acylsugar structure elucidation using NMR spectroscopy

**Fig. 3:**
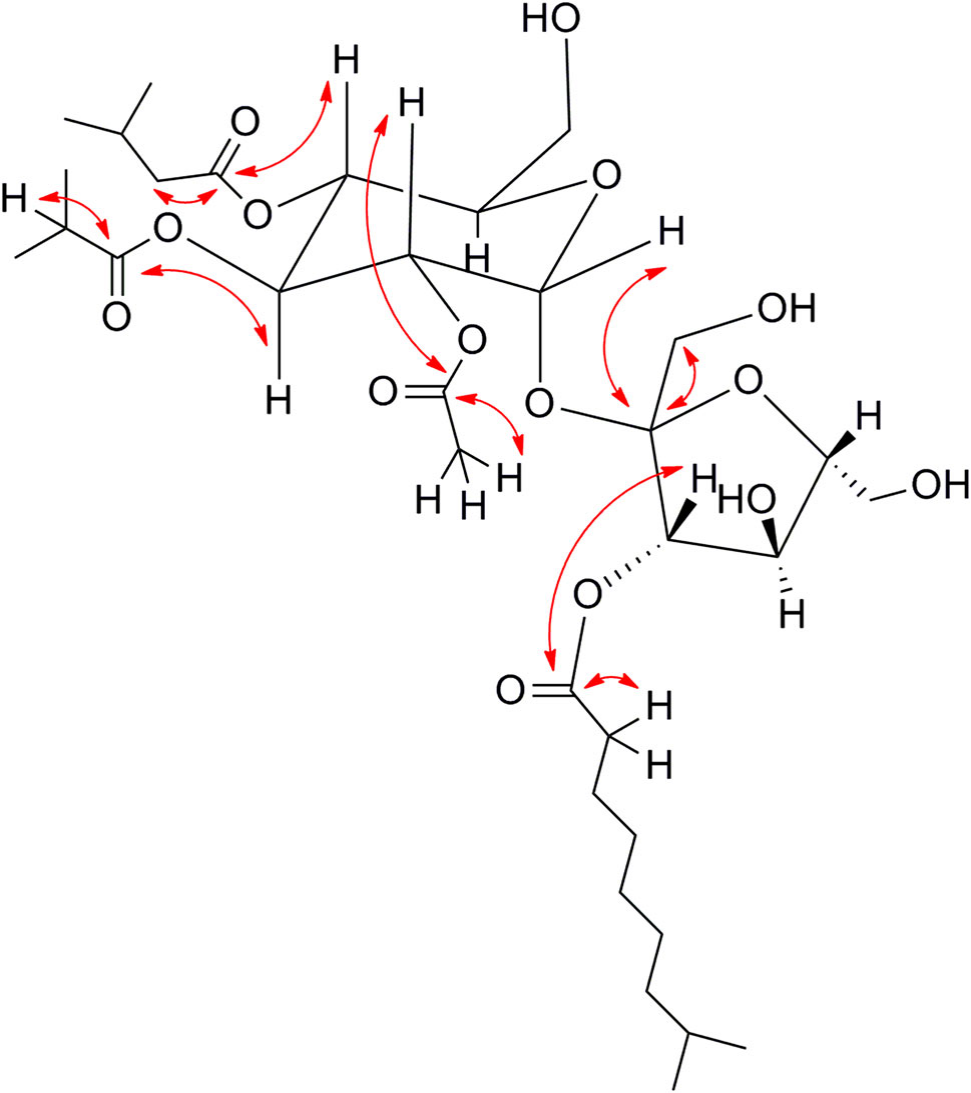
HMBC correlations used to assign positions of substitution of the acyl groups and quaternary carbons in acylsugar S4:21[2] (2,4,5,10) from *S. habrochaites* LA1777

To assess the structural diversity among acylsugars with a long-term goal of probing the genetic factors that control this diversity, 1D (^1^H and ^13^C) and 2D (HSQC, COSY, HMBC, TOCSY and NOESY) NMR spectra were generated for 24 acylsugars purified from tomato and three *S. habrochaites* accessions. The proton resonances (determined from ^1^H and COSY spectra) in all purified acylsugars fell in three distinct chemical shift regions, 0.6–1.8 ppm (aliphatic hydrogens from the acyl groups), 1.9–2.6 ppm (H′s α to the ester groups) and 3.2–5.8 ppm (sucrose ring H′s). The ^13^C, HSQC and HMBC spectra were used to assign carbon chemical shifts of ring and acyl group carbons, beginning with the protons at the 1-position which are furthest downfield (δ 5.49–5.76 ppm; supplementary material). Acylation on the various sugar hydroxyl groups shifts the corresponding ring proton resonances about 1 ppm further downfield, and this information, when combined with ^1^H–^1^H couplings established from ^1^H NMR spectra allows for recognition of the positions on the sucrose core that are acyl-substituted. Positions of substitution of individual acyl groups on the sucrose core were determined from HMBC spectra, which show spin correlations between ^1^H and ^13^C nuclei separated by more than one bond. In all cases, the hydrogen(s) α to the ester carbonyl of the acyl groups and the sucrose ring hydrogen attached to the carbon at which the acid was esterified showed cross-peak(s) with the corresponding ester carbonyl carbon in the HMBC spectra. For example, acylsugar S4:21[2] showed four carbonyl carbon resonances (172.0, 177.9, 173.6, and 175.3 ppm) correlated with ^1^H resonances α to ester groups at 2.00, 2.48, a pair at 2.15 and 2.21, and 2.50 ppm and to sucrose ring ^1^H resonances at 4.88, 5.43, 5.12, and 5.38 ppm (positions 2, 3, 4, and 3′). Further correlations confirmed from 2D NMR spectra identified the acyl groups as C2, iC4, iC5, and iC10 at these positions (Fig. 3). Finally, coupled HSQC experiments showed the coupling constant of the anomeric hydrogen with the anomeric carbon is in the range of ~170 Hz, indicating β orientation of the anomeric hydrogens (α glycosidic linkage). Based on the coupling constants of the pyranose and furanose ring hydrogens, ^13^C NMR shifts, NOESY and coupled HSQC experiments, the relative stereochemistry for all ring hydrogens was assigned based on NMR spectral characteristics of carbohydrates (Duus et al. 2000). Table 1 presents structures of all acylsugars in LA1777, LA1392 and M82 that have been unequivocally established by 1D and 2D NMR spectra, with assessment of their relative levels determined by their UHPLC/MS peak areas normalized to tissue dry weight.

### Comparison of acylsugar profiles in two* Solanum habrochaites* accessions

**Fig. 4:**
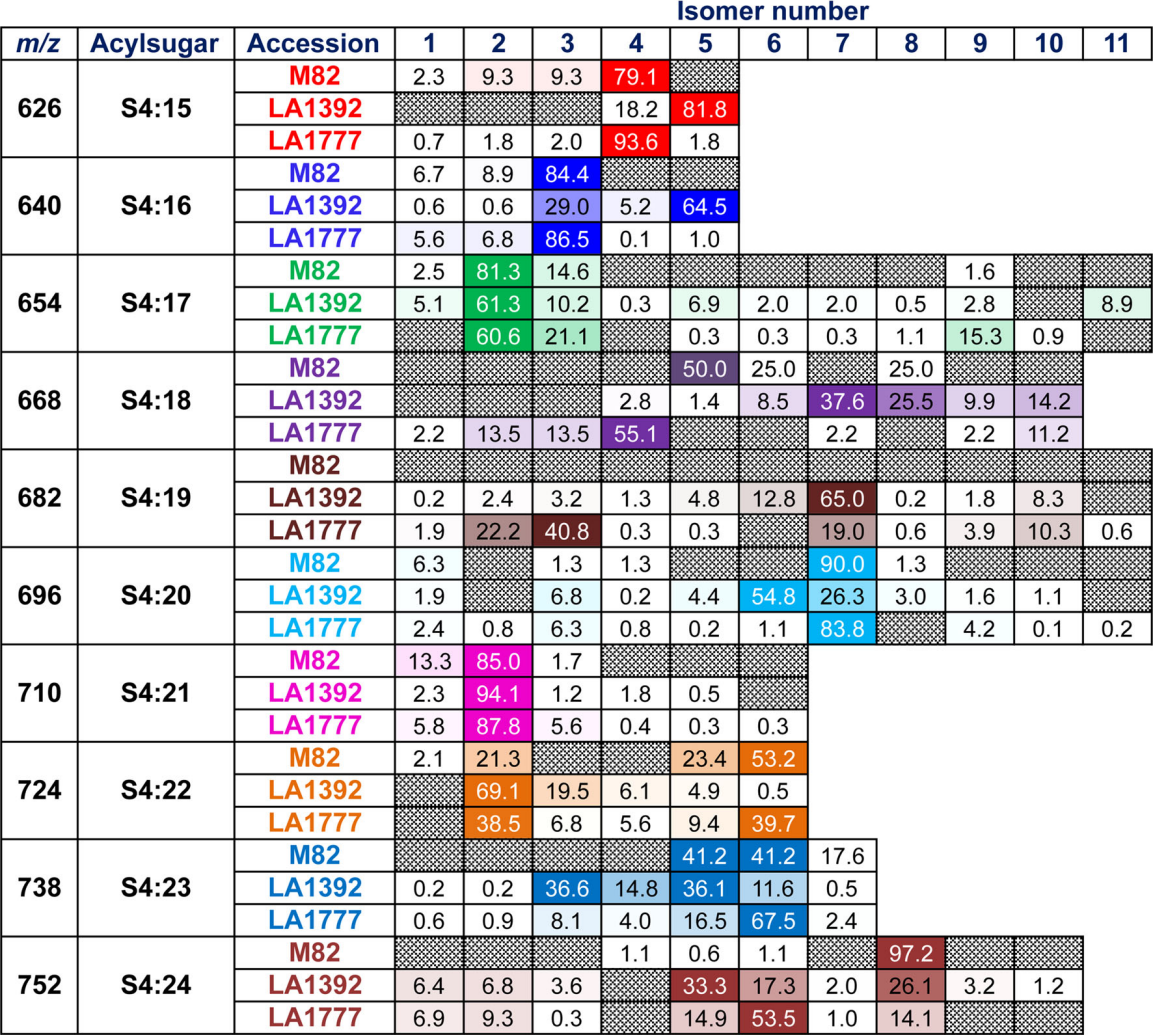
Heat map showing the isomers associated with sucrose tetraesters detected in extracts of *S. habrochaites* LA1777 and LA1392 and *S. lycopersicum* M82. Isomer annotations are based on order of chromatographic elution, with higher numbers indicating greater retention. Numbers and shadings within the heat *map boxes* indicate percentage of an isomer among its other isomeric counterparts as calculated from LC/MS extracted ion chromatogram peak areas using positive ion mode electrospray ionization. *Gray boxes* indicate isomers detected in at least one accession, but were below detection limit for a specific accession

Despite the similarity and close proximity of the natural habitats of the two *S. habrochaites* accessions (3,150 and 3,120 m elevation for LA1777 and LA1392 respectively; approximately 12 km between sampling sites as calculated from coordinates at http://tgrc.ucdavis.edu/), they showed notable differences in acylsugar profiles (Fig. 1). Not only did they exhibit qualitative differences (e.g. total number of isomers, substitution and branching of acyl groups) in their respective acylsugar pools, quantitative differences of individual acylsugar contents were also prominent among these three plants (see Fig. 4; Table 1 and supplementary material for isomeric sucrose triesters and pentaesters). Figure 4 illustrates the differences in sucrose tetraester isomers in *S. habrochaites* LA1777, LA1392 and *S. lycopersicum* M82 (for similar comparisons on sucrose triesters and sucrose pentaesters, see supplementary material). For example, one of the most abundant S4:22 isomers (Isomer 6 with C2, iC4, iC4, and nC12) in LA1777 was present in LA1392, but at minimal abundance. Whether these differences arise from differences in acyltransferase expression levels, catalytic efficiencies, or precursor (e.g. CoA ester) substrate abundances remains to be determined.

Two sucrose pentaesters rich in C5 acyl groups (S5:24[3] and S5:25[4]), three triesters (S3:19[9], S3:21[5], and S3:22[5]), and two tetraesters (S4:19[7] and S4:20[6]) are unusual in being substituted with iC5 esters at the 2-positions. The two pentaesters were ~tenfold more abundant in LA1392 compared to LA1777. Since the pentaesters with the same molecular masses, chromatographic retention times, and CID mass spectra were substantially more abundant in another *S. habrochaites* accession (LA1362), they were purified from this accession instead of LA1392.

### Acylsugar profiles in tomato (*S. lycopersicum* M82)

Though our earlier rapid LC/MS screening of *S. lycopersicum* documented only four acylsucrose metabolites (Schilmiller et al. 2010), the current study documented 15 acylsugar metabolites in tomato, six of which were abundant. These included one triacylsucrose substituted on the 3, 4, and 3′ positions (S3:22[4]), and five tetraacylsucroses substituted at 2, 3, 4, and 3′ positions, with acetate at the 2-position (including S4:16[3], S4:17[2], S4:20[7], S4:21[2], and S4:24[8], Fig. 1). The longer chain (C10–C12) acyl groups were found on either the 3- or 3′ positions, but not both. No tetraacylsucroses with iC5 at the 2-position were observed as *S. lycopersicum* metabolites, but a trace of triacylsucrose S3:21[5] that has iC5 at position 2 was observed (Table 1). The narrower range of acylsugars in tomato relative to all *S. habrochaites* accessions investigated to date is consistent with the loss of trichome-derived specialized metabolites during tomato domestication Rodriguez et al. (1993). Much of this can be attributed to less incorporation of isobutyrate (iC4), isodecanoate (iC10) and 8-methyldecanoate (aiC11) esters in *S. lycopersicum* acylsugars, whereas both *S. habrochaites* accessions accumulated several abundant acylsugars esterified to these acyl groups.

### Diversity and conservation in position-selective acylation

A growing body of evidence suggests that acyl substitution in acylsucroses depends on multiple acyltransferase enzymes that vary in positional selectivity. (Schilmiller et al. 2012; Kim et al. 2012). Consistent with previous reports of acylsugar structures from *S. habrochaites* LA1777 (King et al. 1993), the NMR spectra documented acetylation at the 2-position of sucrose in 13 of the 15 purified tetraacylsucroses in the current study. This finding is in accord with selective 2-position acetylation catalyzed by BAHD acyltransferase *SlAT2* (Kim et al. 2012). However, two of the five triacylsucroses in this accession were esterified at the 2-position by C5 esters; iC5 in one case (S3:21[5]), and aiC5 in the other (S3:22[5]). In addition, the NMR spectra revealed pentaacylsucroses S5:24[3] and S5:25[4], in which the 2-position was substituted with iC5 instead of acetate, in both *S. habrochaites* accessions but not in *S. lycopersicum*.

NMR spectra demonstrated that the greatest diversity of acyl groups attached at specific positions on the sucrose was observed at the 3-position on the pyranose ring, which spanned the range of acyl groups from iC4 to nC12 but did not include acetyl. The 3′-position also exhibited diversity of substitution, with all groups except iC4 and aiC5 detected at this site. The longer acyl groups (C10, C11 and C12) in the purified sucrose tri- and tetraesters were not observed except at either the 3- or 3′ positions but not at both, as consistent with earlier findings (King et al. 1990, 1993). In contrast, the 4-position displayed the least diversity of acyl groups among the acylated positions, with 22 of 24 structures having iC5 at the 4-position (see Table 1), the only two exceptions having iC4. None of the purified metabolites showed evidence for acylation at positions 6, or 4′, which stands in contrast to the report of iC4 acylation at the 6-position (King et al. 1993). Only pentaesters showed substitution at the 1′ position.

Though the order in which acyl groups are added during acylsugar biosynthesis remains uncertain, the observed selectivity of substitution in acylsucroses is consistent with the actions of site- and substrate-selective activity of multiple acyltransferases. The two *S. habrochaites* accessions in this study accumulated sucrose triesters with two distinct substitution at 3, 4, and 3′ positions (normally eluting earlier) and 2,3, and 4 positions (eluting later). The 3, 4, 3′-substituted sucrose triesters are particularly revealing in that they are anticipated direct precursors (or hydrolysis products) of 2,3,4,3′-substituted sucrose tetraesters that are abundant in both *S. habrochaites* accessions. Existence of 2,3,4- substituted acylsugars in *S. habrochaites* was previously reported in accession LA1353 (King et al. 1990), but not in LA1777. Triesters purified from LA1392 included those esterified to a long chain acid (iC10, aiC11 or nC12) at the 3-position of sucrose, but long-chain substitution at this position was only observed in triesters when iC4 or aiC5, and not acetyl, was esterified at the 2-position. One tetraester, S4:24[8], exhibited a long chain (nC12) at the 3-position, and was observed in *S. lycopersicum* in great relative abundance.

Substitution on the furanose moiety was proposed by King and colleagues to involve acylation at the 2, 3, and 4 positions preceding furanose ring acylation (1′ acylation from their observations), and that a long chain acyl group on the pyranose ring inhibits further acylation on the furanose ring (King et al. 1990). However, purified sucrose tetraester (S4:24[8]) esterified to nC12 at the 3-position and iC5 at the 3′-position demonstrates that furanose acylation is not incompatible with long chain groups at position 3. This particular acylsucrose tetraester was the third-most abundant acylsugar in *S. lycopersicum* based on UHPLC/MS peak areas, and had similar absolute abundances in LA1777 and LA1392 (40 and 52 % respectively of the abundance in M82 when normalized to leaflet dry weights). Moreover, using sucrose triesters and acetyl CoA as substrates and recombinant enzyme *SlAT2*, the sucrose triester was reported to be acetylated at position 2 even in presence of a long chain acyl group (Schilmiller et al. 2012). These findings suggest that the pattern of acyl substitution is governed in part by the substrate selectivity of acyltransferases, and long chain acylation at 3- or 3′- position does not prevent further acylation.

To our knowledge, acylation at the 6′-position has not been reported in tomato acylsugars, though it has been documented in *Petunia sp* metabolites (Begum et al. 2004). Acylation at the 6′ position was not observed in any of the purified *S. lycopersicum* metabolites, but iC5 acylation was observed at the 6′ position in *S. habrochaites* accessions in the form of tetraesters S4:19[7], S4:20[6] and pentaesters S5:24[3] and S5:25[4]. Levels of these metabolites were higher in LA1392 than in LA1777 (e.g. 2- and 20-fold for S4:19[7] and S4:20[6]). When acylation occurred at the 6′-position, no acyl substitution was ever observed at the 3′-position.

Finally, the relative abundances of various branched and linear acyl groups are expected to reflect a complex network of contributions from substrate pool sizes and substrate-selective activities of biosynthetic (and degradative) enzymes in glandular trichomes. UHPLC/MS profiling revealed four dominant LA1777 acylsugars with nC12 acyl groups, S4:22[6], S4:23[6], S4:24[6] and S4:24[8], based on integrated UHPLC/MS peak areas from extracted ion chromatograms for *m/z* 199.17 (C12 anion) at CID potential of 60 V. Of these, the first three were substituted with nC12 at the 3′ position, and S4:24[8] was substituted with nC12 at position 3. These four metabolites accounted for about 70 % of C12-containing acylsugars in LA1777. In contrast, two of three most abundant C12-substituted acylsugars in LA1392, S4:23[5] and S4:24[5], were esters of 10-methylundecanoic acid (iC12), and they accounted for ~30 % of C12 acyls in LA1392 determined the same way. The most abundant nC12 ester in this accession was triacylsucrose S3:22[5], which accounted for ~60 % of all C12 esters.

### Diversity in acyl groups and implications for elongation mechanisms

This comparative structural profiling of acylsugars also casts light on the acid elongation process in tomato and its wild relatives. Aliphatic acid elongation is believed to take place in tomato via fatty acid synthase (FAS)-mediated pathway (2-carbon elongation) using acetyl-acyl carrier protein or acetyl CoA (Kroumova and Wagner 2003; van der Hoeven and Steffens 2000). When this mechanism is at work, iso-branched acids with even numbers of carbon atoms are expected to be synthesized from 2-oxo-3-methylbutyric acid (either a precursor or product of valine). By way of comparison, iso-branched acids with odd numbers of carbon are believed synthesized from 2-oxo-4-methylvaleric acid (leucine pathway) and anteiso-branched acids are explained by derivation from 2-oxo-3-methylvaleric acid (isoleucine pathway). All C10 acyl groups in acylsugars whose structures were elucidated by NMR were iso-branched, while all C11 acyl groups determined by NMR were anteiso-branched (branching two carbons removed from the terminus). In contrast, the purified acylsugars with C12 acyl groups were either straight chain (normal fatty acid pathway) or iso-branched. This is consistent with the FAS-mediated acyl elongation pathway where branched even-carbon acids are synthesized from valine, straight chain acids are formed via normal fatty acid biosynthesis mechanisms, and odd-carbon acids by elongation of leucine or isoleucine, all with the increment of two carbons per elongation cycle (Kroumova and Wagner 2003; van der Hoeven and Steffens 2000).

## Conclusions

This investigation has documented far greater complexity and diversity in acylsucrose metabolite structures than was apparent in earlier reports, yet acylation patterns exhibit a remarkable selectivity that differentiates genotypes. These advances have relied in large part on the powerful combination of modern UHPLC/MS and high field NMR spectroscopy. Metabolite profiles are dynamic in nature, and the abundance of individual metabolites will probably depend on environmental conditions, stages of trichome development, and genetic factors. The detailed biochemical basis for differences in metabolite structures across multiple genotypes remains to be revealed, and testing of functions of candidate biosynthetic enzymes will require deep profiling of specialized metabolites and preparation of suitable substrates such as those identified in this work. Such detailed understanding of the events that control levels of individual acylsugars is expected to facilitate future investigations regarding the physiological and ecological roles of these metabolites and the genes involved in their accumulation.

How does one compare chemical phenotypes among genetic variants of non-model plants when the metabolites within a structural class have not previously been identified? In the case of acylsucroses from tomato and its relatives, tandem mass spectrometry alone was not sufficient to distinguish metabolite structures, particularly with regard to positions of substitution and branching. Such structural variations lie at the heart of the differences between metabolite profiles, and are assumed to arise in large part from differences in enzyme substrate specificity that result from mutation. Only through comprehensive comparisons of both structures and abundances of metabolites within a class can the discovery of key biosynthetic and regulatory genes be accelerated, as is needed to explain plant chemical diversity. We recognize that some members of a metabolite class may have abundances too low to allow for their isolation and complete characterization. In such cases, these may be annotated through chromatographic retention times and fragment ion MS/MS spectra. Owing to the complexity of acylsugar profiles, neither retention time nor accurate molecular masses are sufficient to provide unambiguous metabolite identification, and researchers are advised to establish metabolite identity through additional supporting information including NMR analyses. The research community is encouraged to pursue deeper elucidation of specialized metabolite structures, particularly in non-model plants where many of these substances have yet to be identified.

## Electronic supplementary material

Below is the link to the electronic supplementary material.

Supplementary material 1 (PDF 5169 kb)
