# Supplementary material for: Comparative structural profiling of trichome specialized metabolites in tomato (Solanumlycopersicum) and S. habrochaites: acylsugar profiles revealed by UHPLC/MS and NMR
Source: Metabolomics. 2013 Sep 19;10(3):496–507. doi: 10.1007/s11306-013-0585-y (PMC3984663; doi:10.1007/s11306-013-0585-y)
Supplement: Supplementary file 1 — Supplementary material 1 (PDF 5169 kb) [file 11306_2013_585_MOESM1_ESM.pdf]

Comparative Structural Profiling of Trichome Specialized Metabolites in Tomato (*Solanum lycopersicum*) and *S. habrochaites*: Acylsugar Profiles Revealed by LC/MS and NMR

Banibrata Ghosh[1], Thomas C. Westbrook[1] and A. Daniel Jones[1,2]*

[1]*Department of Biochemistry and Molecular Biology, Michigan State University, East Lansing, MI 48824*

[2]*Department of Chemistry, Michigan State University, East Lansing, MI 48824*

*Address correspondence to: A. Daniel Jones

E-mail: jonesar4@msu.edu

FAX: 517-353-9334

Supplemental Information

Index

LC-MS metadata **S2-S4**

NMR metadata **S5-S7**

Plant metadata **S8**

Heat map for trichome-derived isomeric sucrose tri-esters and penta-esters **S9**

Tabular ^{1}H and ^{13}C chemical shift data of the purified acylsugars **S10-S11**

Purification of *S. habrochaites* and *S. lycopersicum* acylsugars **S12-S18**

Detailed spectral information of purified acylsugars **S19-S186**

LC-MS Metadata

Lab PI	Prof. A. D. Jones
Analyst	Dr. Banibrata Ghosh
LC system	Prominence Liquid Chromatograph (High Performance)
Manufacturer	Shimadzu
Degasser	DGU-20A5
Solvent Delivery	LC-20AD
System Controller	SCL-10AVP
Autosampler	SIL-5000
Column Oven	CTO-20A
Column	Ascentis Express C18 Analytical HPLC column, 10 cm x 0.21 cm, 2.7 µm
Manufacturer of column	Supelco
Catalogue number	53823-U
Serial number	USRB003199
Packing Lot number	S12026
Injection volume	10 µL
Flow rate	0.3 mL/min
Mobile phases	
A	10 mM Ammonium formate in water (adjusted to pH 2.80 with formic acid)
B	Acetonitrile
Gradient Profile	Gradient program started with 1% B/99% A, held for one minute, increased to 80% B/20% A at hundred minutes, then to 100% B at 101 minutes, held at 100% B for 4 minutes. decreased to 1% B/99% A in one minute and held at 1% B/99% A for

	four minutes resulting to a total analysis time of 110 minutes
Column oven temperature	40˚C
Sample temperature in the autosampler	10˚C
Inlet method name	Bani_Acylsugar_110min
Mass Spectrometer	Micromass® LCT Premier™ time-of-flight (TOF) mass spectrometer
Manufacturer	Waters
Software	MassLynx™ Version 4.1
Ionization source	Electrospray ionization (ESI)
Data acquisition	W mode, centroid
Polarity	Negative and Positive
Mass Range	*m/z* 50-1500
Aperture 1 voltage	
Function 1	10 V
Function 2	20 V
Function 3	40 V
Function 4	60 V
Function 5	80 V
MS method file	Bani_110min_acylsugars_ESINEG (Negative mode)<br>Bani_110min_acylsugars_ESIPOS (Positive mode)
Sample handling	Ten leaflets from the node adjacent to the apical tissue from of each of *S. habrochaites* accessions LA1777, LA1392, and cultivated tomato (*S. lycopersicum* M82) were harvested by cutting the petioles at the stem. Leaflets were immediately dipped into 10 mL of methanol in beakers separately for each plant for 2 minutes. Each extract was quantitatively transferred to a 15-mL polypropylene centrifuge tube, and solvent was evaporated to dryness under nitrogen. Residues were redissolved by adding 0.5 mL acetonitrile/water (4/1 v/v) to each tube followed by vortexing for two minutes. These solutions were centrifuged at 25 °C and 2627x*g* for 10 minutes. Aliquots

Sample handling	Ten leaflets from the node adjacent to the apical tissue from of each of *S. habrochaites* accessions LA1777, LA1392, and cultivated tomato (*S. lycopersicum* M82) were harvested by cutting the petioles at the stem. Leaflets were immediately dipped into 10 mL of methanol in beakers separately for each plant for 2 minutes. Each extract was quantitatively transferred to a 15-mL polypropylene centrifuge tube, and solvent was evaporated to dryness under nitrogen. Residues were redissolved by adding 0.5 mL acetonitrile/water (4/1 v/v) to each tube followed by vortexing for two minutes. These solutions were centrifuged at 25 °C and 2627x*g* for 10 minutes. Aliquots (200 µL) of each supernatant were transferred into 250-µL glass inserts placed in 2 mL HPLC vials. These were used directly for LC/MS analyses.
Sample storage temperature	-20˚C
Protocol when analyzing the samples	Before each set of analyses, the mass spectrometer mass axis was calibrated using 0.1% phosphoric acid. After column equilibration, one methanol/water (4/1 v/v) blank injection was performed. M82, LA1392 and LA1777 extracts were injected sequentially after that followed by another methanol/water (4/1 v/v) blank injection. Same order of injections was

NMR metadata

Analysis description	
a) Supervisor	Mr. Kermit Johnson and Dr. Daniel Holmes
b) Operator	Mr. Kermit Johnson
c) Institution	Michigan State University
d) Date and time of data acquisition	June 22nd, 2012-March 11th, 2013
Sample description	
a) Field frequency lock	Methanol-d_4, chloroform-d_1, acetonitrile-d_3
b) Additional solute	None
c) Solvent	CD_3OD, $CDCl_3$, CD_3CN (300 μL)
d) Chemical shift standard	Solvent
e) Concentration standard	none
Instrument description	
a) Geographic location of the instrument	42.7164, -84.4677
b) Magnet	Manufacturer and model: Bruker ultrastabilized<br>Field strength: 899.54 MHz (21.14 T)
c) Probe	Manufacturer and model: Bruker TCI triple-resonance inverse detection cryoprobe
d) Console	Manufacturer and model: Bruker Avance
e) Acquisition computer	Manufacturer and model: Dell T3500<br>Operating System and version number: CentOS 5.8<br>Software and version number: Topspin 2.1.6
Acquisition parameters	
Parameters recorded once for each sample	
a) Acquisition parameters file reference	^{1}H:<br>/opt/topspin216/exp/stan/nmr/lists/pp/zg<br>^{13}C:<br>/opt/topspin216/exp/stan/nmr/lists/pp/zgpg30<br>HSQC:<br>/opt/topspin216/exp/stan/nmr/lists/pp/hsqcedetgpsp<br>HMBC: /opt/topspin216/exp/stan/nmr/lists/pp/hmbcgpndqf<br>COSY: /opt/topspin216/exp/stan/nmr/lists/pp/cosygpmfph
b) Shaped pulse file reference	Gauss, Gaus1.1000, Sinc1.1000, Crp60_xflit.2
c) Sample details	Tube and size: Shigemi (5 mm) NMR tube<br>Temperature: 298 K
d) Instrument operation details	Sample spinning rate: No spinning,<br>Pulse sequence names with literature reference:<br>^{1}H: zg<br>^{13}C: zgpg30,<br>HSQC: hsqcedetgpsp (G. Bodenhausen, D. J. Ruben, *Chem. Phys. Lett.* **1980**, *69*, 185-189.)<br>HMBC: hmbcgpndqf (A. Bax, M. F. Summers, *J. Am. Chem. Soc.* **1986**, *108*, 2093-2094)<br>COSY: cosygpmfph (W. P. Aue, J. Karhan, R. R. Ernst, *J. Chem. Phys.* **1976**, *64*, 4226-4227.)<br>NOSEY: noseygpphpr (S. Macura, Y. Huang, D. Suter, R. R. Ernst, *J. Magn. Reson.* **1981**, *43*, 259-281)

e) Data acquisition details	Steady state scans:<br>^{1}H: 0<br>^{13}C: 8<br>HSQC: 8<br>HMBC: 16<br>COSY: 4<br>Number of scans:<br>^{1}H: 64<br>^{13}C: 12500<br>HSQC: 32<br>HMBC: 40<br>COSY: 24
Parameters recorded once for each NMR analysis dimension	
a) Instrument operation details	Radiation frequency:<br>^{1}H: 899.5395<br>^{13}C: 226.2109<br>HSQC: 899.5495, 226.2064<br>HMBC: 899.5408, 226.2131<br>COSY: 899.5394<br>Acquisition nucleus:<br>^{1}H (90° = 9 μs), except for ^{13}C (90° = 13 μs) NMR experiments
b) Data acquisition details	Dwell time:<br>^{1}H: 27 μs<br>^{13}C: 9.225 μs<br>HSQC: 36.9 μs<br>HMBC: 36.9 μs<br>COSY: 55.6 μs<br>Number of data points acquired:<br>^{1}H: 148144<br>^{13}C: 65536<br>HSQC: 1024, 400<br>HMBC: 4096, 400<br>COSY: 2048, 400
Parameters recorded for higher dimensions	Encoding scheme:<br>HSQC: Echo-Antiecho<br>HMBC: QF<br>COSY: States-TPPI
Quality control	None
FID and spectral processing parameters	
a) Parameters recorded once when the raw data set is processed	Data transformation: Fourier transform<br>Processing software: Topspin 2.1.6
b) Parameters recorded once for each NMR analysis dimension	Number of data points in spectrum:

	^{1}H: 262144<br>^{13}C: 131072<br>HSQC: 2048, 2048<br>HMBC: 4096, 400<br>COSY: 2048, 400<br><br>Window function details:<br>^{1}H: exponential (0.3 Hz)<br>^{13}C: exponential (2 Hz)<br>HSQC: QSINE (SSB = 2)<br>HMBC: SINE (SSB = 0)<br>COSY: QSINE (SSB = 2, 1)

Plant Metadata

Species	*Solanum habrochaites*<br>*Solanum lycopersicum*
Genotype(s)	*S. habrochaites* LA1777<br>*S. habrochaites* LA1392<br>*S. lycopersicum* LA3475 *(*M82)
Organ	Leaf
Organ specification	Leaflets (adjacent to the apical tissue); leaflets were between 25% and 75% of mature leaflet size.
Cell type	Extraction procedure selectively extracts metabolites from glandular trichomes
Biosource amount	10 leaflets per genotype
Growth support	4:1 Baccto/sand
Growth location	Plant growth chamber 21, Michigan State University
Light	16 h/8 h; 150 $\mu mol{\text -}m^{-2}{\text -}s^{-1}$
Humidity	86%
Temperature	28 ˚C
Watering regime	Every day between 4 and 5 pm
Nutritional regime	Three times per week; 1 teaspoon/gal Scott's General Purpose 20:20:20 fertilizer
Date(s) of plant establishment	May 8th, 2013; propagated from stem cuttings
Harvest date, time	May 26th, 2013, between 1400 and 1800
Plant growth stage	18 days
Metabolism quenching method	Extraction into methanol
Harvest method	Leaflets were harvested using forceps at the junction of the stem and petiole.
Sample storage	Tissue samples were processed immediately after harvest; extracts were stored in 1.5 mL centrifuge tubes and transferred to 2 mL HPLC vials with 250 µL glass inserts for LC/MS analysis. After analysis, the extracts were stored in a -20 ˚C degree freezer.

Heat map showing the isomers associated with sucrose triesters and pentaesters detected in extracts of *S. habrochaites* LA1777 and LA1392 and *S. lycopersicum* M82. Isomer annotations are based on order of chromatographic elution, with higher numbers indicating greater retention. Numbers and shadings within the heat map boxes indicate percentage of an isomer among its other isomeric counterparts as calculated from LC-MS extracted ion chromatogram peak areas. Gray boxes indicate isomers detected in at least one accession, but were below detection limit for the specific accession.

			Isomer number									
m/z	SE	Accession number	1	2	3	4	5	6	7	8	9	10
668	S3:19	M82				12.5	87.5					
		LA1392	3.5	10.0	2.2	0.4	26.5		0.4	32.2	24.8	
		LA1777	1.1	0.4	0.8	2.7	93.2	1.9				
682	S3:20	M82	7.7		11.5	80.8						
		LA1392	14.1	0.7	12.1	72.6				0.5		
		LA1777	50.8	4.2	8.4		35.0				1.6	
696	S3:21	M82			14.5		14.3			71.4		
		LA1392	7.7	4.0	5.3	2.6	41.2	16.0	5.9	5.9	4.2	7.3
		M1777	8.1	4.6	14.6		60.0	0.4		11.9	0.4	
710	S3:22	M82	1.1	0.4		98.6						
		LA1392	4.3	1.8	4.7	37.8	51.4					
		LA1777	16.7	48.9		31.1	3.3					
766	S5:24	M82										
		LA1392	0.3	8.3	82.8	0.9	0.5	5.8		1.5		
		LA1777	1.7		73.1	4.0	17.1	0.6	1.7	1.7		
780	S5:25	M82										
		LA1392			1.8	93.7	4.6					
		LA1777	1.0	3.0	5.0	75.0	16.0					

Acylsugar core ^{1}H chemical shifts

#	Solvent	Acylsugar	Acyl groups	Positions	C1	C2	C3	C4	C5	C6	C1'	C3'	C4'	C5'	C6'
1	$CDCl_3$	S3:19[5]	4,5,10	3,4,3'	5.49	3.71	**5.22**	**4.93**	4.07	3.59; 3.64	3.64;3.74	**5.04**	4.66	3.97	3.71;3.91
2	$CDCl_3$	S3:19[9]	4,5,10	2,3,4	5.73	**4.87**	**5.55**	**4.95**	4.14	3.62; 3.66	3.53;3.61	4.22	4.32	3.78	3.71;3.88
3	$CDCl_3$	S3:20[4]	5,5,10	3,4,3'	5.49	3.71	**5.23**	**4.93**	4.06	3.59; 3.63	3.65; 3.73	**5.04**	4.65	3.97	3.70; 3.90
4	$CDCl_3$	S3:21[1]	5,5,11	3,4,3'	5.49	3.70	**5.22**	**4.93**	4.06	3.59; 3.64	3.65; 3.73	**5.04**	4.66	3.97	3.70; 3.90
5	$CDCl_3$	S3:21[5]	5,5,11	2,3,4	5.75	**4.87**	**5.55**	**4.94**	4.16	3.61; 3.65	3.53; 3.61	4.22	4.32	3.77	3.71; 3.88
6	$CDCl_3$	S3:22[4]	5,5,12	3,4,3'	5.51	3.71	**5.22**	**4.90**	4.06	3.59; 3.63	3.65; 3.74	**5.05**	4.65	3.97	3.72; 3.91
7	$CDCl_3$	S3:22[5]	5,5,12	2,3,4	5.76	**4.87**	**5.55**	**4.93**	4.16	3.60; 3.65	3.52; 3.60	4.22	4.32	3.77	3.71; 3.88
8	$CDCl_3$	S4:16[3]	2,4,5,5	2,3,4,3'	5.60	**4.93**	**5.46**	**4.95**	4.13	3.61	3.50; 3.61	**5.20**	4.58	3.94	3.72; 3.91
9	$CDCl_3$	S4:17[2]	2,5,5,5	2,3,4,3'	5.61	**4.91**	**5.49**	**4.94**	4.13	3.61	3.50; 3.62	**5.18**	4.60	3.93	3.72; 3.91
10	$CDCl_3$	S4:19[7]	4,5,5,5	2,3,4,6'	5.57	**5.00**	**5.52**	**4.98**	4.11	3.61	3.58; 3.61	4.21	4.11	3.99	**4.21; 4.48**
11	$CDCl_3$	S4:20[6]	5,5,5,5	2,3,4,6'	5.58	**4.99**	**5.52**	**4.97**	4.15	3.61; 3.67	3.57; 3.61	4.21	4.12	3.98	**4.20; 4.47**
12	$CDCl_3$	S4:20[7]	2,4,4,10	2,3,4,3'	5.60	**4.93**	**5.45**	**4.93**	4.14	3.60	3.52; 3.63	**5.16**	4.61	3.93	3.71; 3.90
13	$CDCl_3$	S4:21[2]	2,4,5,10	2,3,4,3'	5.64	**4.88**	**5.43**	**5.12**	4.12	3.56; 3.67	3.44; 3.58	**5.38**	4.28	3.88	3.78
14	$CDCl_3$	S4:22[2]	2,5,5,10	2,3,4,3'	5.61	**4.86**	**5.47**	**4.91**	4.11	3.59	3.51; 3.63	**5.13**	4.55	3.90	3.69; 3.87
15	$CDCl_3$	S4:22[3]	2,4,5,11	2,3,4,3'	5.60	**4.91**	**5.48**	**4.95**	4.13	3.62	3.52; 3.62	**5.16**	4.60	3.92	3.71; 3.90
16	CD_3OD	S4:22[6]	2,4,4,12	2,3,4,3'	5.64	**4.88**	**5.45**	**5.11**	4.14	3.55; 3.66	3.44; 3.58	**5.39**	4.29	3.88	3.77
17	$CDCl_3$	S4:23[3]	2,5,5,11	2,3,4,3'	5.62	**4.89**	**5.50**	**4.94**	4.13	3.62	3.53; 3.64	**5.15**	4.60	3.91	3.70; 3.90
18	$CDCl_3$	S4:23[5]	2,4,5,12	2,3,4,3'	5.60	**4.91**	**5.47**	**4.95**	4.14	3.61	3.52; 3.63	**5.16**	4.60	3.92	3.71; 3.90
19	$CDCl_3$	S4:23[6]	2,4,5,12	2,3,4,3'	5.60	**4.91**	**5.47**	**4.95**	4.13	3.62	3.52; 3.62	**5.16**	4.59	3.92	3.71; 3.90
20	$CDCl_3$	S4:24[5]	2,5,5,12	2,3,4,3'	5.61	**4.88**	**5.49**	**4.93**	4.13	3.61	3.52; 3.63	**5.14**	4.60	3.90	3.71; 3.90
21	$CDCl_3$	S4:24[6]	2,5,5,12	2,3,4,3'	5.61	**4.89**	**5.50**	**4.94**	4.13	3.62	3.52; 3.64	**5.15**	4.60	3.90	3.71; 3.90
22	$CDCl_3$	S4:24[8]	2,5,5,12	2,3,4,3'	5.60	**4.90**	**5.47**	**4.93**	4.12	3.61	3.50; 3.61	**5.19**	4.60	3.94	3.71; 3.90
23	CD_3CN	S5:24[3]	4,5,5,5,5	2,3,4,1',6'	5.53	**4.93**	**5.45**	**5.02**	4.09	3.48; 3.59	**4.00**	3.97	3.94	3.82	**4.24**
24	$CDCl_3$	S5:25[4]	5,5,5,5,5	2,3,4,1',6'	5.53	**4.89**	**5.47**	**5.00**	4.09	3.48; 3.59	**4.00**	3.97	3.93	3.82	**4.24**

Resonances highlighted in bold are those on which an ester group is attached.

Acylsugar core ^{13}C chemical shifts

#	Solvent	Acylsugar	Acyl groups	Positions	C1	C2	C3	C4	C5	C6	C1'	C2'	C3'	C4'	C5'	C6'
1	$CDCl_3$	S3:19[5]	4,5,10	3,4,3'	91.9	70.8	**72.5**	**67.7**	72.9	61.4	64.9	103.5	**81.5**	71.5	81.9	59.6
2	$CDCl_3$	S3:19[9]	4,5,10	2,3,4	89.2	**70.7**	**68.9**	**68.4**	72.0	61.4	64.8	104.7	79.0	73.7	82.0	59.8
3	$CDCl_3$	S3:20[4]	5,5,10	3,4,3'	92.0	70.9	**72.4**	**67.7**	72.9	61.4	64.9	103.6	**81.4**	71.4	81.9	59.6
4	$CDCl_3$	S3:21[1]	5,5,11	3,4,3'	92.0	70.9	**72.4**	**67.7**	72.9	61.4	64.9	103.6	**81.4**	71.4	81.9	59.6
5	$CDCl_3$	S3:21[5]	5,5,11	2,3,4	89.1	**70.6**	**68.9**	**68.4**	71.9	61.4	64.6	104.5	78.8	73.4	81.7	59.9
6	$CDCl_3$	S3:22[4]	5,5,12	3,4,3'	91.8	70.7	**72.7**	**67.8**	72.8	61.4	65.0	103.5	**81.4**	71.3	81.9	59.6
7	$CDCl_3$	S3:22[5]	5,5,12	2,3,4	88.9	**70.7**	**68.9**	**68.4**	72.0	61.3	64.7	104.5	78.8	73.3	81.7	59.9
8	$CDCl_3$	S4:16[3]	2,4,5,5	2,3,4,3'	89.4	**70.3**	**69.0**	**68.2**	72.1	61.6	64.5	103.9	**79.5**	71.2	82.5	59.7
9	$CDCl_3$	S4:17[2]	2,5,5,5	2,3,4,3'	89.4	**70.4**	**68.8**	**68.4**	72.1	61.6	64.5	103.9	**79.6**	71.2	82.4	59.6
10	$CDCl_3$	S4:19[7]	4,5,5,5	2,3,4,6'	89.3	**70.1**	**69.2**	**68.6**	72.2	61.6	64.3	104.4	78.7	75.9	79.4	**64.2**
11	$CDCl_3$	S4:20[6]	5,5,5,5	2,3,4,6'	89.2	**70.1**	**69.3**	**68.7**	72.0	61.5	64.2	104.3	78.6	75.8	79.4	**64.3**
12	$CDCl_3$	S4:20[7]	2,4,4,10	2,3,4,3'	89.5	**70.4**	**68.8**	**68.2**	72.2	61.6	64.6	104.0	**80.0**	71.3	82.4	59.6
13	$CDCl_3$	S4:21[2]	2,4,5,10	2,3,4,3'	90.6	**72.0**	**71.4**	**69.7**	72.1	61.7	64.8	105.1	**79.0**	73.6	82.3	63.2
14	$CDCl_3$	S4:22[2]	2,5,5,10	2,3,4,3'	89.4	**70.5**	**68.6**	**68.4**	71.9	61.6	64.5	104.0	**79.8**	71.4	82.3	59.8
15	$CDCl_3$	S4:22[3]	2,4,5,11	2,3,4,3'	89.5	**70.4**	**68.8**	**68.2**	71.1	61.6	64.5	104.0	**80.0**	71.3	82.4	59.6
16	CD_3OD	S4:22[6]	2,4,4,12	2,3,4,3'	90.6	**72.0**	**71.3**	**69.7**	72.0	61.6	64.8	105.1	**78.8**	73.6	84.3	63.2
17	$CDCl_3$	S4:23[3]	2,5,5,11	2,3,4,3'	89.3	**70.4**	**68.6**	**68.2**	71.9	61.5	64.3	104.0	**79.9**	71.2	82.2	59.4
18	$CDCl_3$	S4:23[5]	2,4,5,12	2,3,4,3'	89.5	**70.4**	**68.8**	**68.2**	72.1	61.6	64.5	104.0	**79.9**	71.2	82.4	59.6
19	$CDCl_3$	S4:23[6]	2,4,5,12	2,3,4,3'	89.5	**70.4**	**68.8**	**68.2**	72.1	61.6	64.5	104.0	**79.9**	71.3	82.4	59.6
20	$CDCl_3$	S4:24[5]	2,5,5,12	2,3,4,3'	89.3	**70.4**	**68.6**	**68.5**	71.9	61.6	64.4	103.8	**79.8**	71.0	82.1	59.4
21	$CDCl_3$	S4:24[6]	2,5,5,12	2,3,4,3'	89.4	**70.6**	**68.8**	**68.4**	72.1	61.7	64.5	104.0	**80.1**	71.3	82.4	59.6
22	$CDCl_3$	S4:24[8]	2,5,5,12	2,3,4,3'	89.4	**70.0**	**68.6**	**68.0**	71.8	61.3	64.1	103.8	**79.4**	70.8	82.2	59.3
23	CD_3CN	S5:24[3]	4,5,5,5,5	2,3,4,1',6'	90.0	**70.8**	**70.4**	**69.3**	71.4	61.7	**63.3**	104.0	77.8	75.1	80.3	**63.3**
24	$CDCl_3$	S5:25[4]	5,5,5,5,5	2,3,4,1',6'	89.9	**70.9**	**70.2**	**69.3**	71.7	61.6	**63.3**	104.0	77.8	75.1	80.3	**65.4**

Resonances labeled in bold are those on which an ester group is attached.

1.1. Purification of *S. habrochaites* LA1777 acylsugar metabolites

S. habrochaites LA1777 plants were germinated from seeds obtained from the C. M. Rick Tomato Genetics Resource Center (University of California Davis, CA USA). The plants were grown in a growth chamber at 28 ˚C and 86% relative humidity using a light/dark cycle (150 µmol m^{-2} s^{-1}) of 17 h/7 h until 6 weeks post-germination, and then were transferred to a laboratory window sill with ample sunlight. At 6 months post-germination, 130 leaflets were harvested and placed in a 1 L beaker, to which 1000 mL of 100% methanol was added. The mixture was stirred for 2 minutes with a glass rod, and then quickly transferred into a 1 L glass bottle through a Buchner funnel fitted with filter paper. Solvent was evaporated to dryness under vacuum using a rotary evaporator, and the residue was re-dissolved in 3 mL of acetonitrile: water (4/1 v/v) with ultrasonication for 10 minutes followed by transfer to a polypropylene centrifuge tube and centrifugation at 2627x*g* for 2 min at 25 ˚C. Supernatants were collected in HPLC vials each with glass inserts (300 µL in each) and vials were sonicated for 10 minutes. Subsequent metabolite purification was performed using a Waters Automated Gradient Controller (Model 680) coupled with a Waters HPLC pump (Model 512) and a Dionex Acclaim 120 C18 HPLC column (4.6 x 150 mm, 5 µm). The mobile phase consisted of 0.15% formic acid in water, pH 2.65 (Solvent A) and acetonitrile (Solvent B) using linear gradient elution of 1% B at 0 min, 58% B at 5 min and held at 58% B at 5-10 min, 65% B at 45 min, 100% B at 47 min. Solvent composition was held at 100% B at 47-52 min and then brought back to 1%B at 53 min and held at 1%B at 53-55 min. The solvent flow rate was 1.5 mL/min and the column temperature was 40 ˚C. Eluted fractions were collected in a LKB fraction collector in 1-min fractions for 15 injections, using an injection volume of 150 µL for each injection.

1.1.1. Purification of S4:17[2] from *S. habrochaites* LA1777

This compound was collected at 11 min as a slightly impure fraction as judged by negative ion mode electrospray ionization mass spectrometric analysis. See next section for spectral information.

1.1.2. Purification of S4:20[7] from *S. habrochaites* LA1777

This compound was collected at 16 min as an impure fraction that also contained isomers of bergamotenoic acid (deposited as white crystals and identified by X-ray crystallography). In order to remove bergamotenoic acid from desired acylsugar S4:20[7], this fraction was subjected to another round of purification using Waters Automated Gradient Controller (Model 680) coupled with Waters HPLC system (Model 512) and a Dionex C18 HPLC column (4.6 x 150 mm, 5 um) with mobile phases containing ammonium formate in water, pH 6.25 (Solvent A) and acetonitrile (Solvent B) using linear gradient elution of 1% B at 0-1 min, 50% B at 5 min and held at 50% B at 5-25 min, 100% B at 27 min, held at 100% B at 27-31 min and then brought back to 1% B at 33 min and held at 1%B at 33-35 min. The solvent flow rate was 1.5 mL/min and the column temperature was 40

˚C. Finally, residual traces of bergamotenoic acid isomers were removed using Supelclean™ LC-SAX SPE tubes (Strong anion exchange columns). The column was equilibrated with 20 mL of 0.1 M ammonium acetate followed by 40 mL of 10 mM ammonium acetate. Fraction containing S4:20[7] was dried and re-dissolved in 1600 µL of acetonitrile and 400 µL of ammonium acetate (10 mM), passed through the column under vacuum (flow rate: ~ 1 drop/sec), concentrated and dried to afford pure S4:20[7]. See next section for full spectral information.

1.1.3. Purification of S4:21[2] from *S. habrochaites* LA1777

This compound was collected between 19-21 min as pure fractions. See next section for full spectral information.

1.1.4. Purification of S4:22[2] from *S. habrochaites* LA1777

This compound was collected between 23-25 min as a slightly impure fraction. See next section for full spectral information.

1.1.5. Purification of S4:22[6] from *S. habrochaites* LA1777

This compound was collected at 29 min as a pure fraction. See next section for full spectral information.

1.1.6. Purification of S4:23[6] from *S. habrochaites* LA1777

This compound was collected at 35 min as a pure fraction. See next section for full spectral information.

1.1.7. Purification of S4:24[6] from *S. habrochaites* LA1777

This compound was collected between 42-43 min as pure fractions. See next section for full spectral information.

1.1.8. Purification of S4:24[8] from *S. habrochaites* LA1777

This compound was collected at 47 min as a pure fraction. See next section for full spectral information.

1.2. Purification of *S. habrochaites* LA1392 acylsugar metabolites

S. habrochaites LA1392 plants were germinated from seeds obtained from the C. M. Rick Tomato Genetics Resource Center (University of California Davis, CA USA). The plants were grown in a growth chamber at 28 ˚C and 86% relative humidity using a light/dark cycle (150 µmol m^{-2} s^{-1}) of 17 h/7 h until 6 weeks post-germination, and then were transferred to a laboratory window sill with ample sunlight. At 6 months post-germination, 100 leaflets were harvested and placed in a 1 L beaker, to which 500 mL of 100% methanol was added. The mixture was stirred for 2 minutes with a glass rod, and then quickly transferred into a 1 L glass bottle through a Buchner funnel fitted with filter paper. Solvent was evaporated to dryness under vacuum using a rotary evaporator, and the residue was re- dissolved in 3 mL of acetonitrile:water (4/1 v/v) with ultrasonication for 10 minutes followed by transfer to a polypropylene centrifuge tube and centrifugation at 2627x*g* for 2 min at 25 ˚C. Supernatants were collected in HPLC vials each with glass inserts (300 µL in each) and vials were sonicated for 10 minutes. The purification was performed using Waters Automated Gradient Controller (Model 680) coupled with Waters HPLC system (Model 512) and a Dionex Acclaim 120 C18 HPLC column (4.6 x 150 mm, 5 µm). The mobile phase was consisted of 0.15% formic acid in water, pH 2.62 (Solvent A) and acetonitrile (Solvent B) using a gradient elution of 1% B at 0-1 min, 48% B at 5 min and held at 48% B at 5-15 min, 65% B at 55 min, 100% B at 57 min. Solvent composition was held at 100% B at 57-60 min and then brought back to 1% B at 62 min and held at 1% B from 62-65 min. The solvent flow rate was 1.5 mL/min and the column temperature was 40 ˚C. Eluted fractions were collected in an LKB fraction collector in 1-min fractions for 14 injections, using an injection volume of 150 µL for each injection.

1.2.1. Purification of S3:19[5] from *S. habrochaites* LA1392

This compound was collected at 28 min as a pure fraction. See next section for spectral information.

1.2.2. Purification of S3:19[9] from *S. habrochaites* LA1392

This compound was collected at 33 min as a pure fraction. See next section for spectral information.

1.2.3. Purification of S3:20[4] from *S. habrochaites* LA1392

This compound was collected at 34 min as a pure fraction. See next section for spectral information.

1.2.4. Purification of S3:21[1] from *S. habrochaites* LA1392

This compound was collected at 41 min as a pure fraction. See next section for spectral information.

1.2.5. Purification of S3:21[5] from *S. habrochaites* LA1392

This compound was collected between 45-46 min as an impure fraction. This compound was re-purified using an isocratic flow of 25% of 0.15% formic acid in water, pH 2.62(Solvent A) and 75% methanol (Solvent B). See next section for spectral information.

1.2.6. Purification of S3:22[5] from *S. habrochaites* LA1392

This compound was collected at 56 min as a slightly impure fraction. . See next section for spectral information.

1.2.7. Purification of S4:16[3] from *S. habrochaites* LA1392

This compound was collected at 13 min as a slightly impure fraction. See next section for spectral information.

1.2.8. Purification of S4:17[2] from *S. habrochaites* LA1392

This compound was collected at 15 min as a pure fraction. See next section for spectral information.

1.2.9. Purification of S4:19[7] from *S. habrochaites* LA1392

This compound was collected at 24 min as a pure fraction. See next section for spectral information.

1.2.10. Purification of S4:20[6] from *S. habrochaites* LA1392

This compound was collected between 29-30 min as a slightly impure fraction and was re-purified using an isocratic flow of 52% of 0.15% formic acid in water, pH 2.70 (Solvent A) and 48% of acetonitrile (Solvent B). See next section for spectral information.

1.2.11. Purification of S4:22[3] from *S. habrochaites* LA1392

This compound was collected at 45 min as an impure fraction and was re-purified using an isocratic flow of 25% of 0.15% formic acid in water, pH 2.62 (Solvent A) and 75% of methanol (Solvent B). See next section for spectral information.

1.2.12. Purification of S4:23[3] from *S. habrochaites* LA1392

This compound was collected at 51 min as an impure fraction and was re-purified using an isocratic flow of 42% of 0.15% formic acid in water, pH 2.69 (Solvent A) and 58% of acetonitrile (Solvent B). See next section for spectral information.

1.2.13. Purification of S4:23[5] from *S. habrochaites* LA1392

This compound was collected at 53 min as a pure fraction. See next section for spectral information.

1.2.14. Purification of S4:24[5] from *S. habrochaites* LA1392

This compound was collected at 59 min as a pure fraction. See next section for spectral information.

1.3. Purification of *S. habrochaites* LA1362 acylsugar metabolites (pentaesters)

S. habrochaites LA1362 plants were solely used for purification purposes and were germinated from seeds obtained from the C. M. Rick Tomato Genetics Resource Center (University of California Davis, CA USA). The plants were grown in a growth chamber at 28 ˚C and 86% relative humidity using a light/dark cycle (150 µmol m^{-2} s^{-1}) of 17 h/7 h until 6 weeks post-germination, and then were transferred to a laboratory window sill with ample sunlight. At 10 months post-germination, 115 leaflets were harvested and placed in a 1 L beaker, to which 1000 mL of 100% methanol was added. The mixture was stirred for 2 minutes with a glass rod, and then quickly transferred into a 1 L glass bottle through a Buchner funnel fitted with filter paper. Solvent was evaporated to dryness under vacuum using a rotary evaporator, and the residue was re-dissolved in 3 mL of acetonitrile:water (4/1 v/v) with ultrasonication for 10 minutes followed by transfer to a polypropylene centrifuge tube and centrifugation at 2627x*g* for 2 min at 25 ˚C. Supernatants were collected in HPLC vials each with glass inserts (200 µL in each) and vials were sonicated for 10 minutes. Subsequent metabolite purification was performed using a Waters 2795 HPLC system and a Dionex Acclaim 120 C18 HPLC column (4.6 x 150 mm, 5 µm). The mobile phase consisted of 0.15% formic acid in water, pH 2.67 (Solvent A) and acetonitrile (Solvent B) using linear gradient elution of 1% B at 0-1 min, 66% B at 5 min and held at 66% B at 5-25 min, 100% B at 26 min. Solvent composition was held at 100% B at 26-30 min and then brought back to 1%B at 33 min and held at 1%B at 33-35 min. The solvent flow rate was 1.5 mL/min and the column temperature was 40 ˚C. Eluted fractions were collected in a LKB fraction collector in 1-min fractions for 10 injections, using an injection volume of 200 µL for each injection.

1.2.14. Purification of S5:24[3] from *S. habrochaites* LA1362

This compound was collected at 15 min as an impure fraction and was re-purified using an isocratic flow of 40% of 0.15% formic acid in water, pH 2.69 (Solvent A) and 60% of acetonitrile (Solvent B). This compound was also purified from *S. habrochaites* accession LA1392 (matching retention time and mass spectra) for NMR purposes. See next section for spectral information.

1.2.15. Purification of S5:25[4] from *S. habrochaites* LA1362

This compound was collected at 19 min as a pure fraction. This compound was also purified from *S. habrochaites* accession LA1392 (matching retention time and mass spectra) for NMR purposes. See next section for spectral information.

1.3. Purification of *S. lycopersicum* M82 acylsugar metabolite

S. lycopersicum M82 (cultivated tomato) plants were germinated from seeds obtained from the C. M. Rick Tomato Genetics Resource Center (University of California Davis, CA USA). The plants were grown in a growth chamber at 28 ˚C and 86% relative humidity using a light/dark cycle (150 µmol m^{-2} s^{-1}) of 17 h/7 h until 6 weeks post-germination, and then were transferred to a laboratory window sill with ample sunlight. At 6 months post-germination, 125 leaflets were harvested and placed in a 1 L beaker, to which 800 mL of 100% methanol was added. The mixture was stirred for 2 minutes with a glass rod, and then quickly transferred into a 1 L glass bottle through a Buchner funnel fitted with filter paper. Solvent was evaporated to dryness under vacuum using a rotary evaporator, and the residue was re-dissolved in 3 mL of acetonitrile:water (4/1 v/v) with ultrasonication for 10 minutes followed by transfer to a polypropylene centrifuge tube and centrifugation at 2627x*g* for 2 min at 25 ˚C. Supernatants were collected in HPLC vials each with glass inserts (200 µL in each) and vials were sonicated for 10 minutes. Subsequent metabolite purification was performed using a Waters 2795 HPLC system and a Dionex Acclaim 120 C18 HPLC column (4.6 x 150 mm, 5 µm). The mobile phase consisted of 0.15% formic acid in water, pH 2.74 (Solvent A) and acetonitrile (Solvent B) using linear gradient elution of 1% B at 0-1 min, 65% B at 5 min, 70% B at 25 min, 100% B at 27 min. Solvent composition was held at 100% B at 27-31 min and then brought back to 1%B at 33 min and held at 1%B at 33-35 min. The solvent flow rate was 1.5 mL/min and the column temperature was 40 ˚C. Eluted fractions were collected in a LKB fraction collector in 1-min fractions for 13 injections, using an injection volume of 150 µL for each injection.

1.3.1. Purification of S3:22[4] from *S. lycopersicum* M82

This compound was collected at 18 min as a pure fraction. See next section for spectral information.

S3:19[5] (4,5,10)

Purified from *S. habrochaites* LA1392

HRMS: (ESI) *m/z* calcd for $C_{32}H_{55}O_{16}^{-}$ ([M+HCOO^{-}]): 695.3496, found: 695.3575

Material recovered: 0.5 - 1 mg

NMR solvent: $CDCl_3$

InChI Key: QNNIESRRUPMSBP-LLJBAAJASA-N

Carbon # (group)	^{1}H (ppm)	^{13}C (ppm)
1(CH)	5.49 (d, J = 3.8 Hz)	91.9 ($^{1}J_{CH}$ = 177.3 Hz)
2(CH)	3.71 (m)	70.8
3(CH)	5.22 (t, J = 10.0 Hz)	72.5
3-O-		
-1(CO)		177.1
-2(CH)	2.55 (m)	34.0
-3(CH_3) x 2	1.16 (d, J = 7.0 Hz)	18.8, 18.9
4(CH)	4.93 (t, J = 10.1 Hz)	67.7
4-O		
-1(CO)		172.5
-2(CH_2)	2.17 (dd, J = 15.0, 7.1 Hz), 2.19 (dd, J = 15.0, 7.1 Hz)[a]	43.0
-3(CH)	2.06 (m)	25.4
-4(CH_3) x 2	0.94 (d, J = 6.8 Hz)	See below[b]
5(CH)	4.07 (m)	72.9
6(CH_2)	3.59 (m), 3.64 (m)	61.4
1' (CH_2)	3.64 (m), 3.74 (d, J = 13.3 Hz)	64.9
2' (C)		103.5
3' (CH)	5.04 (d, J = 8.0 Hz)	81.5

3'-O		
-1(CO)		176.0
-2(CH_2)	2.51 (m)	34.2
-3(CH_2)	1.73 (m)	24.9
-4(CH_2)	1.38 (m)	29.2
-5(CH_2)	1.30 (m)	29.5
-6(CH_2)	1.30 (m)	27.3
-7(CH_2)	1.20 (m)	39.0
-8(CH)	1.52 (m)	27.9
-9(CH_3) x 2	0.86 (d, J = 6.6 Hz)	See below[b]
4' (CH)	4.66 (t, J = 8.3 Hz)	71.5
5' (CH)	3.97 (m)	81.9
6' (CH_2)	3.71 (m), 3.91 (dd, J = 13.0, 2.2 Hz)	59.6

[a]Higher order multiplet derived from the constants using gNMR

[b] ^{13}C peak could not be unequivocally assigned among resonances at 22.3, 22.3, 22.4 and 22.6 ppm

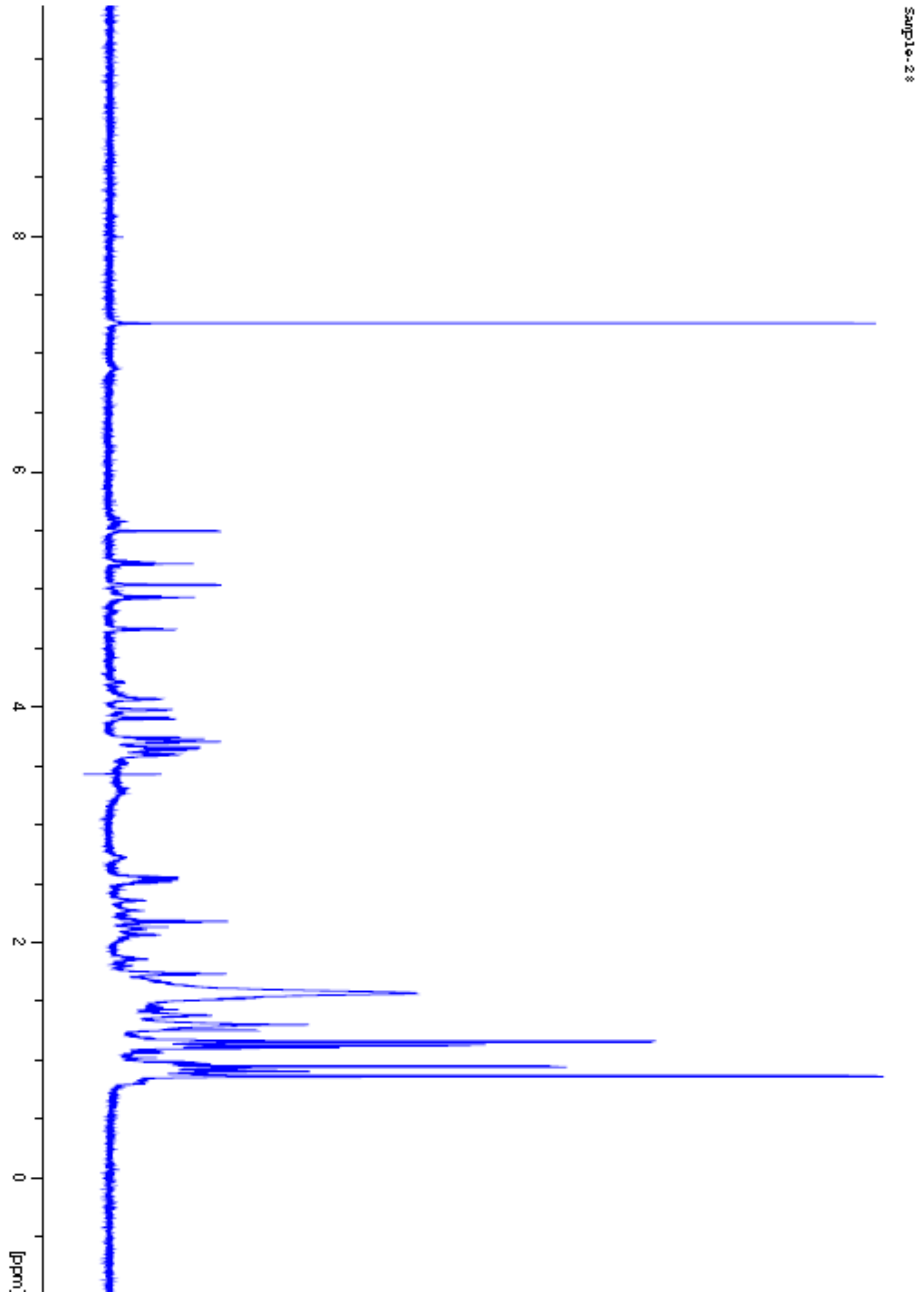
8
6
4
2
0
[ppm]

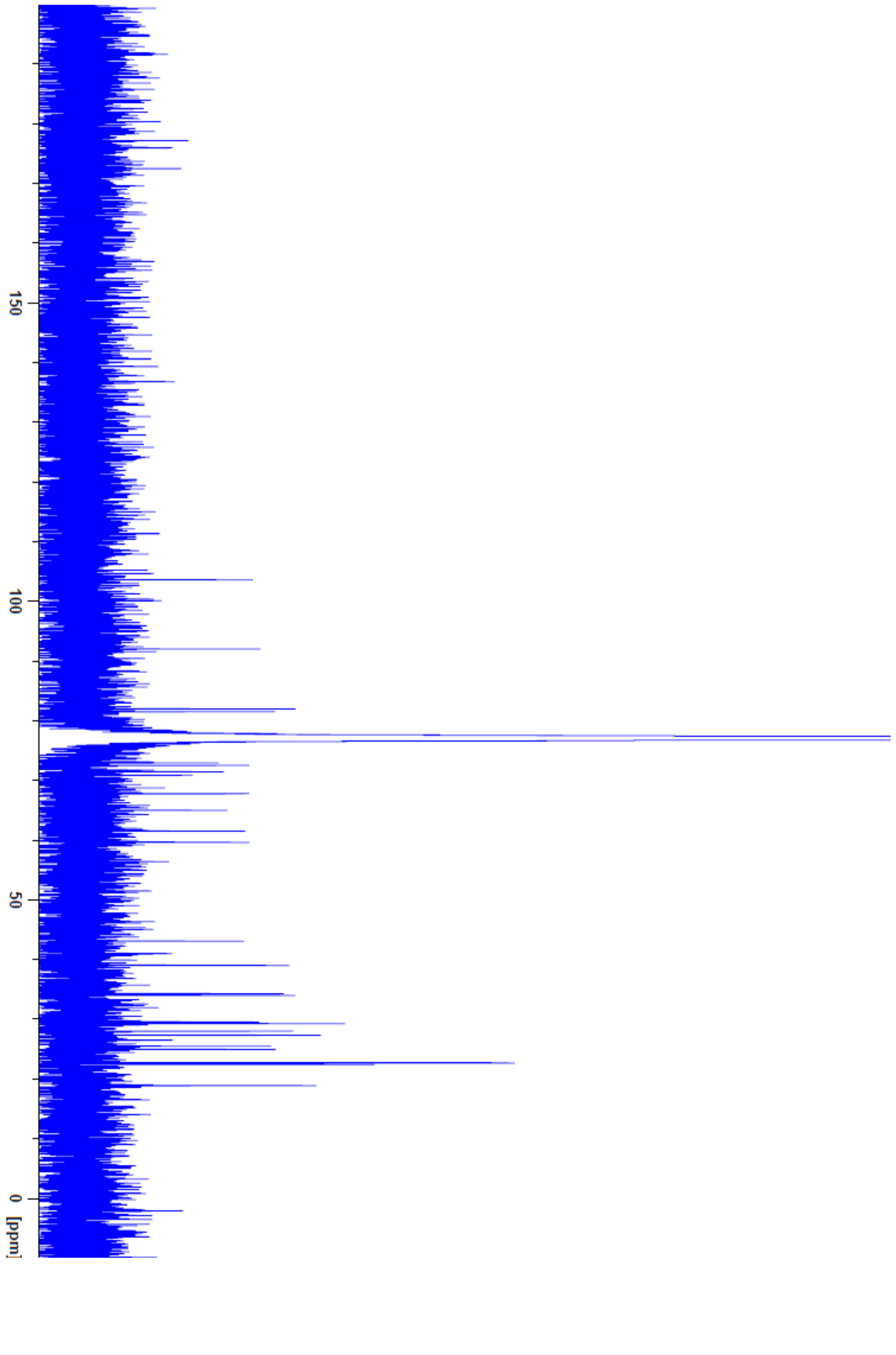
150
100
50
0
[ppm]

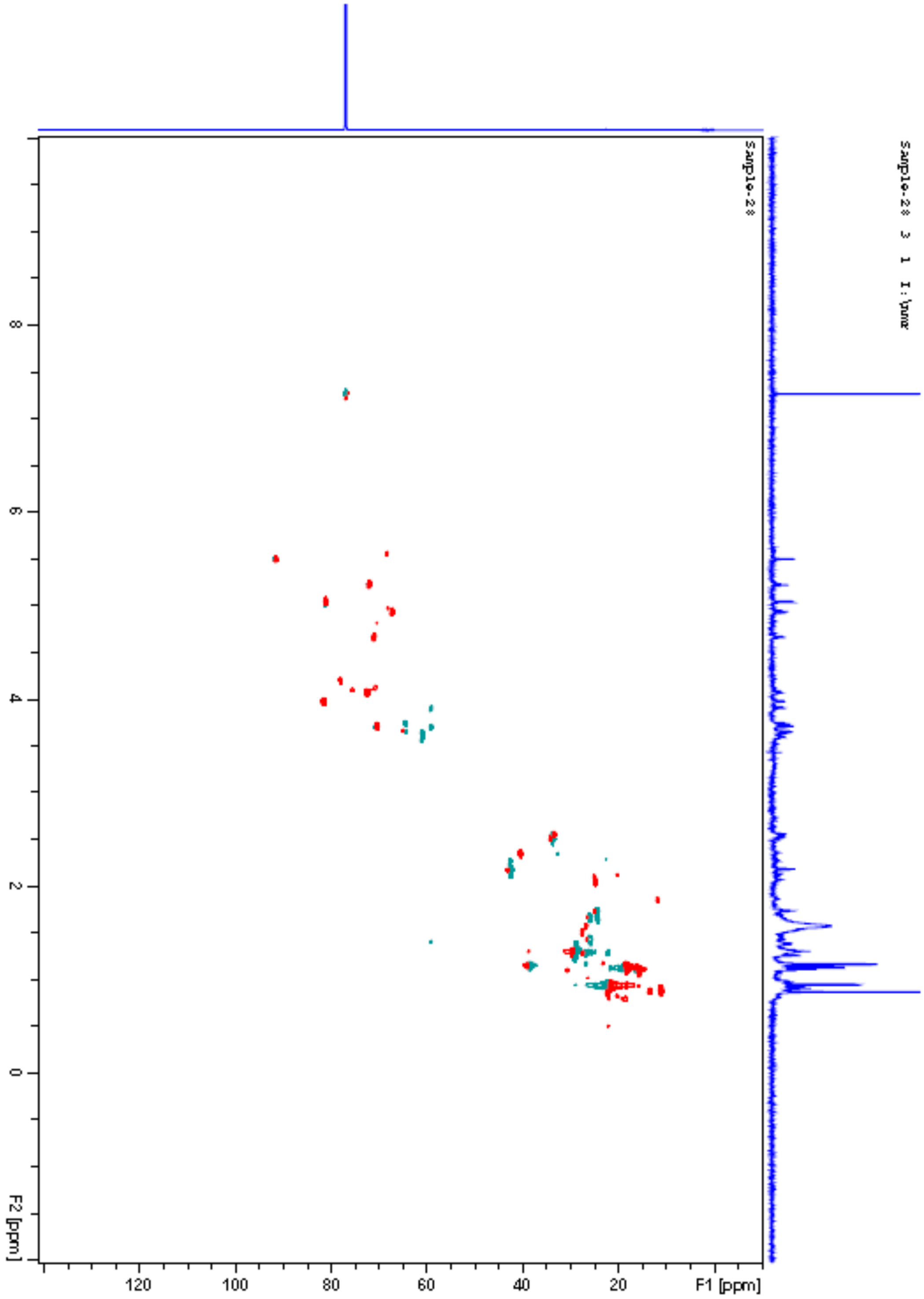
F1 [ppm]
F2 [ppm]

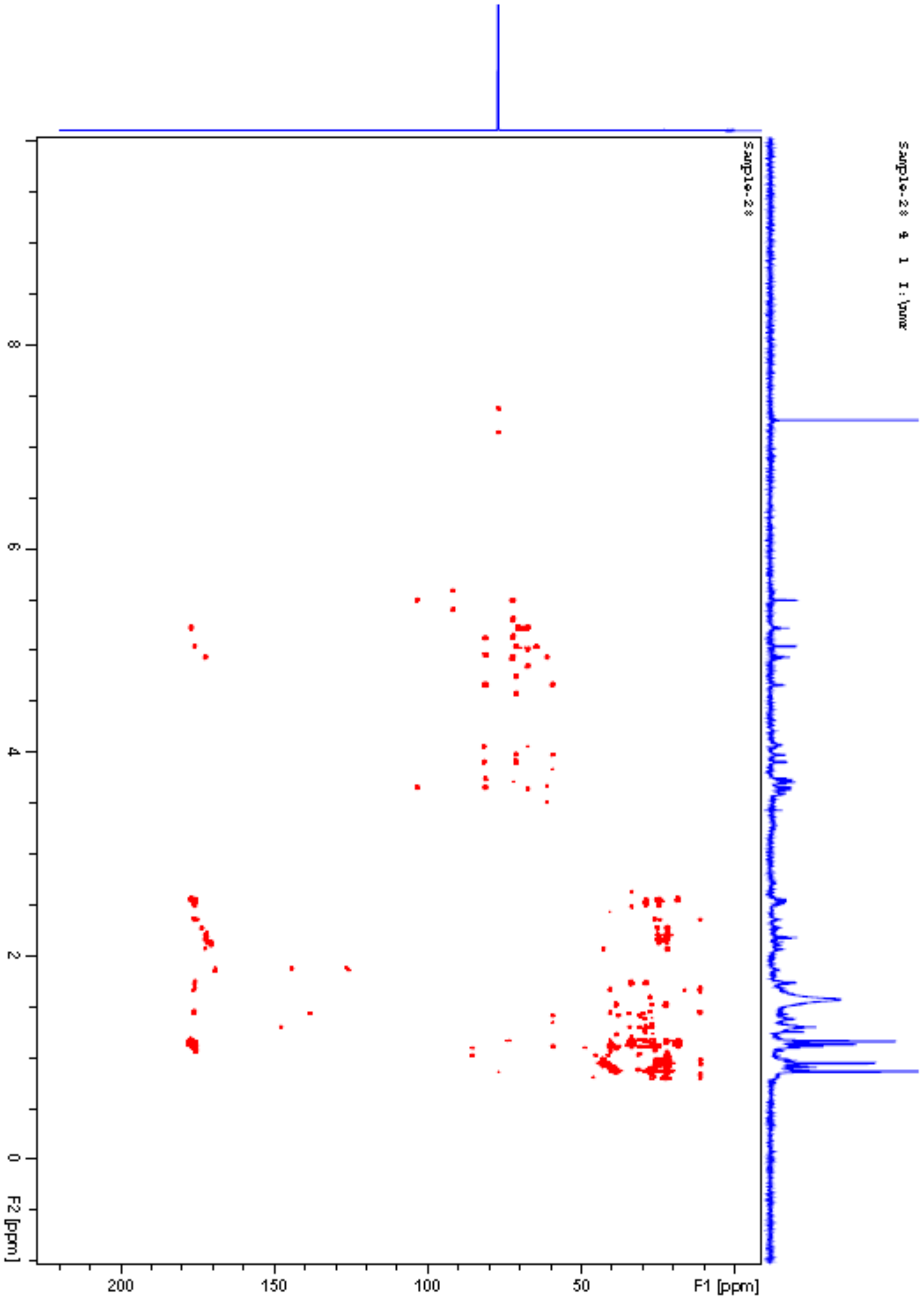

200
150
100
50
F1 [ppm]
8
6
4
2
0
F2 [ppm]

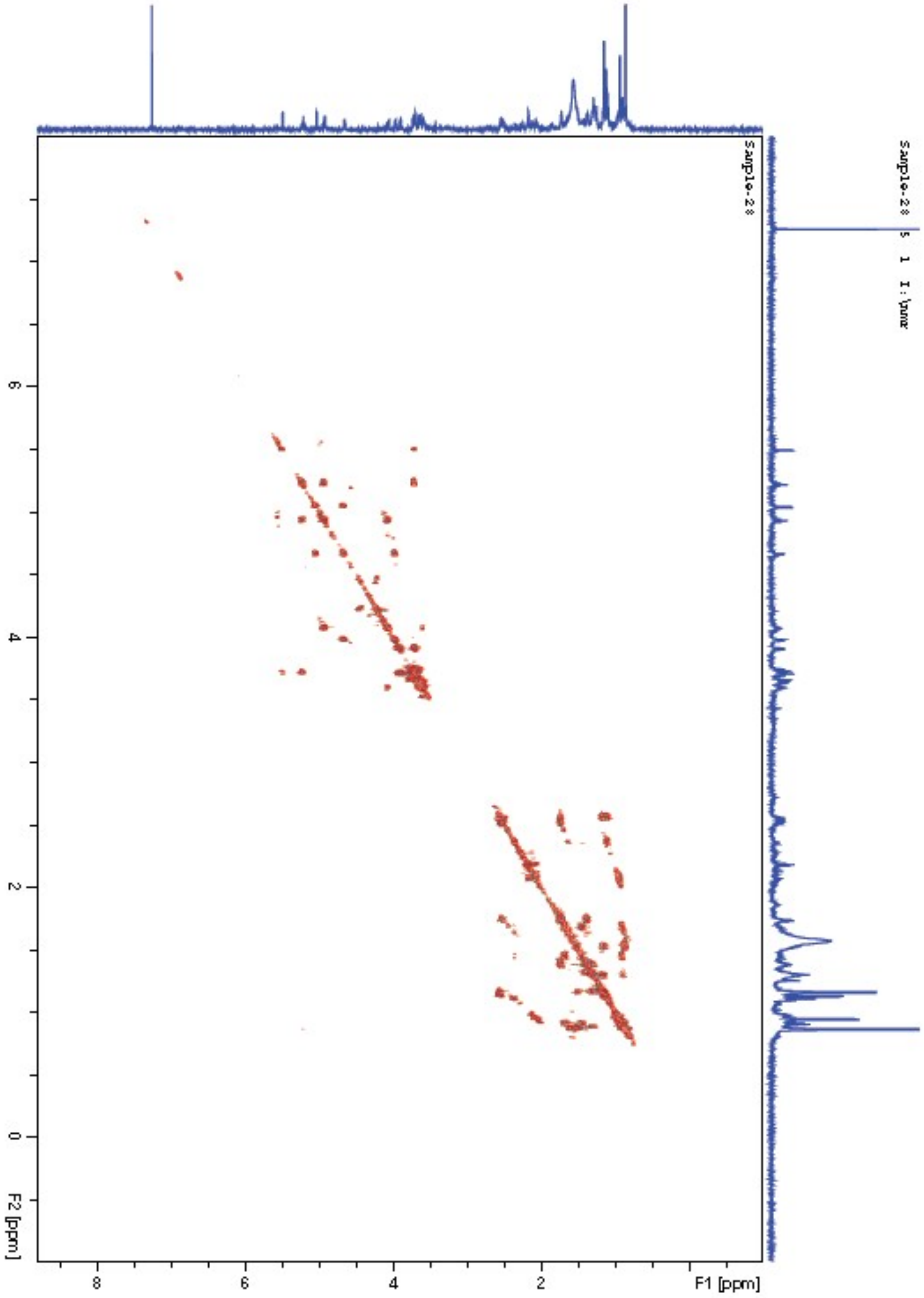

Sample-28
F2 [ppm]
F1 [ppm]

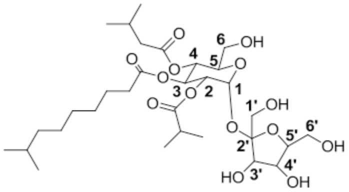

S3:19[9] (4,5,10)

Purified from *S. habrochaites* LA1392

HRMS: (ESI) *m/z* calcd for $C_{32}H_{55}O_{16}^{-}$ ([M+HCOO$^-$]): 695.3496, found: 695.3550

Material recovered: 0.5 - 1 mg

NMR solvent: $CDCl_3$

InChI Key: JMZWUVCPRQAEOU-JUAZSGTLSA-N

Carbon # (group)	^{1}H (ppm)	^{13}C (ppm)
1(CH)	5.73 (d, J = 3.8 Hz)	89.2 ($^1J_{CH}$ = 177.5 Hz)
2(CH)	4.87 (dd, J = 10.2, 3.9 Hz)	70.7
2-O-		
-1(CO)		177.3
-2(CH)	2.55 (m)	33.8
-3(CH_3) x 2	1.13 (d, J = 7.0 Hz), 1.15 (d, J = 7.0 Hz)	18.4, 18.4
3(CH)	5.55 (t, J = 9.9 Hz)	68.9
3-O-		
-1(CO)		173.1
-2(CH_2)	2.22 (m)	34.1
-3(CH_2)	1.53[a]	24.8
-4(CH_2)	1.22-1.29 (m)	29.2
-5(CH_2)	1.22-1.29 (m)	29.5
-6(CH_2)	1.22-1.29 (m)	27.1
-7(CH_2)	1.14[a]	38.9
-8(CH)	1.50 (m)	27.9
-9(CH_3) x 2	0.85 (d, J = 6.7 Hz)	22.6, 22.6
4(CH)	4.95 (t, J = 10.0 Hz)	68.4
4-O		

-1(CO)		172.7
-2(CH_2)	2.18 (m)	43.0
-3(CH)	2.05 (m)	25.5
-4(CH_3) x 2	0.94 (d, *J* = 6.6 Hz)	22.3, 22.3
5(CH)	4.14 (m)	72.0
6(CH_2)	3.62 (d, *J* = 12.1), 3.66 (m)	61.4
1' (CH_2)	3.53 (d, *J* = 12.0 Hz), 3.61[b]	64.8
2' (C)		104.7
3' (CH)	4.22 (d, J = 8.2 Hz)	79.0
4' (CH)	4.32 (t, J = 8.3 Hz)	73.7
5' (CH)	3.78 (m)	82.0
6' (CH_2)	3.71 (d, *J* = 13.3 Hz), 3.88 (d, *J* = 13.3 Hz)	59.8

[a]Determined by COSY

[b]Determined by COSY and HSQC

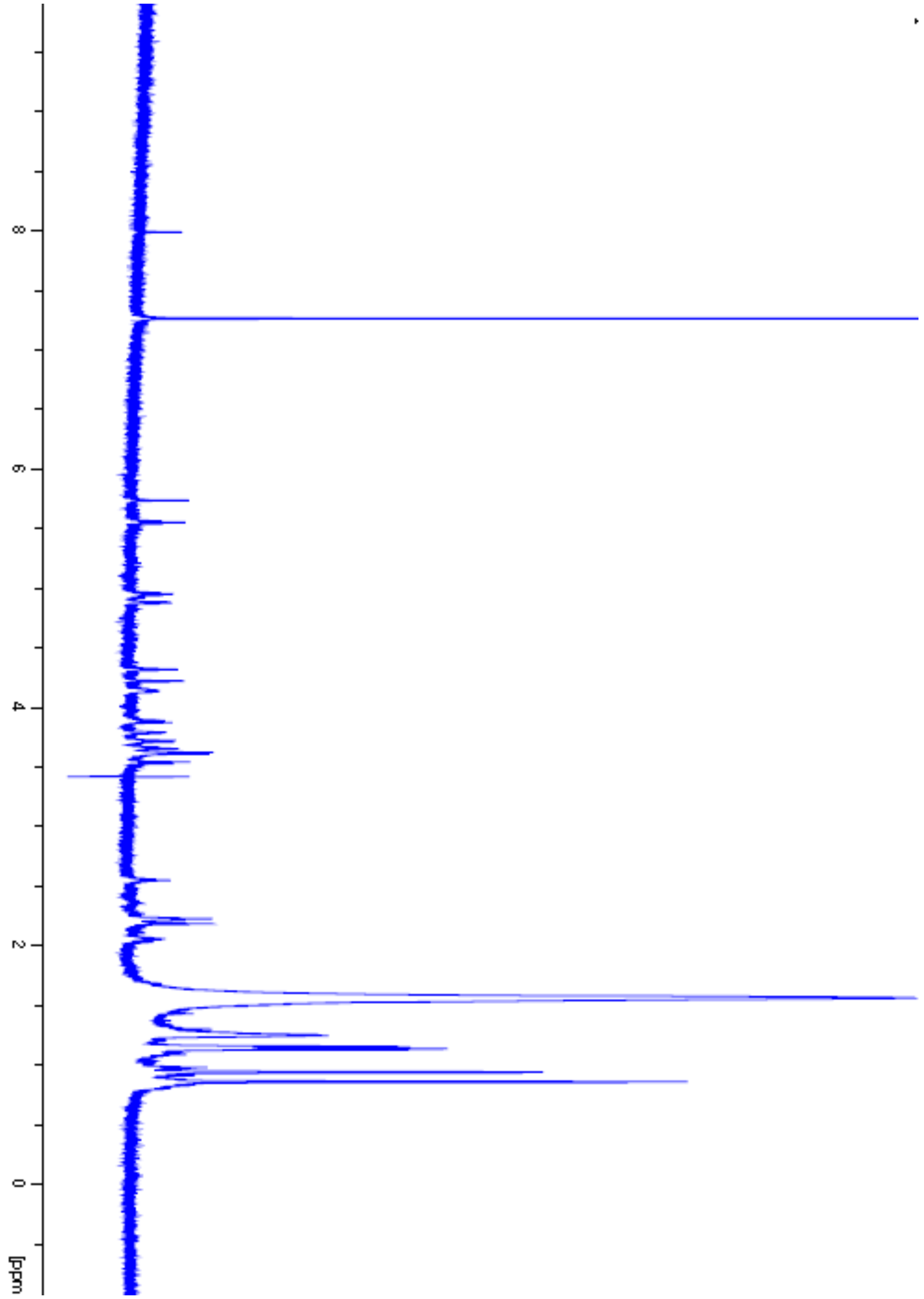
8
6
4
2
0
[ppm]

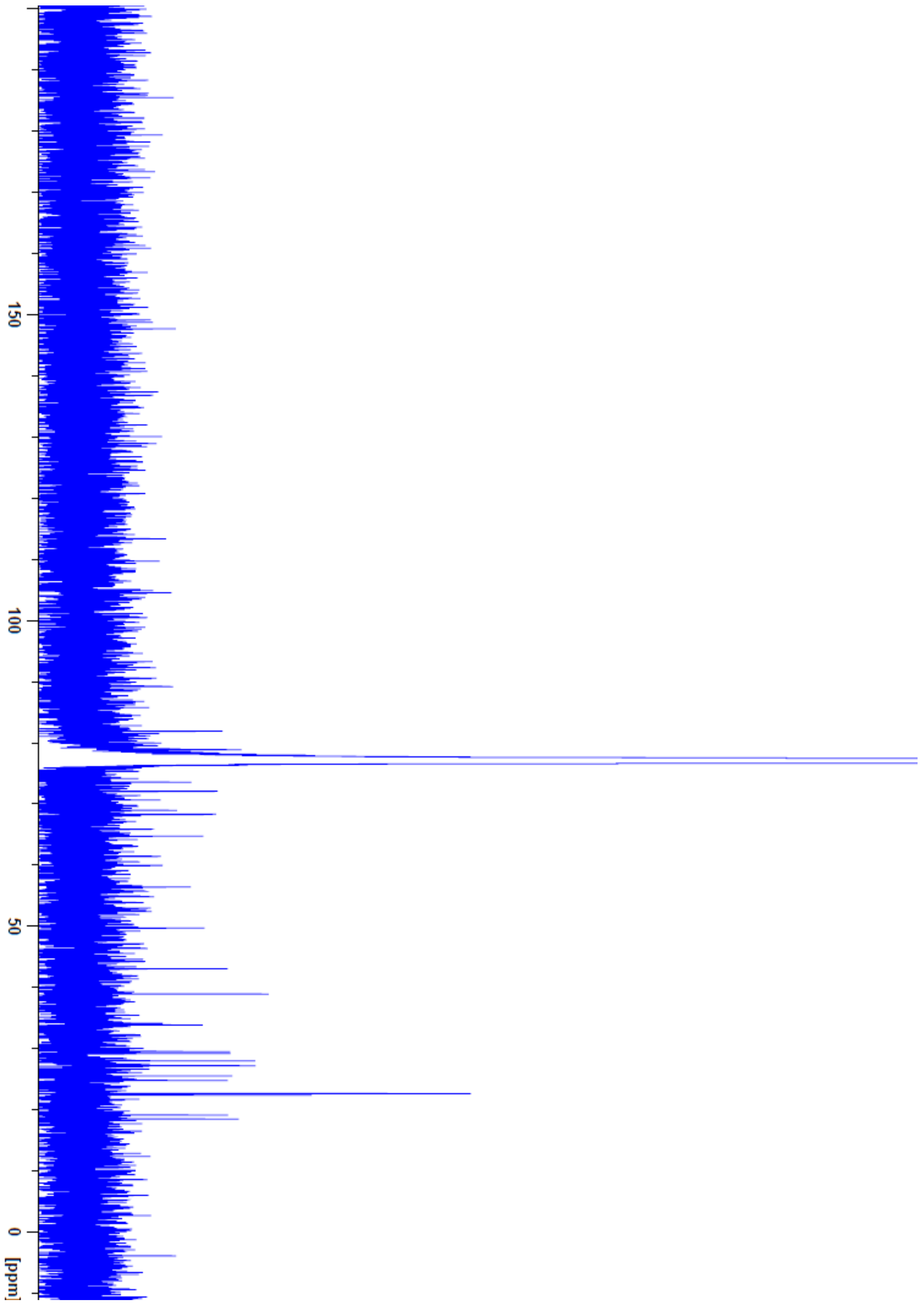
150
100
50
0
[ppm]

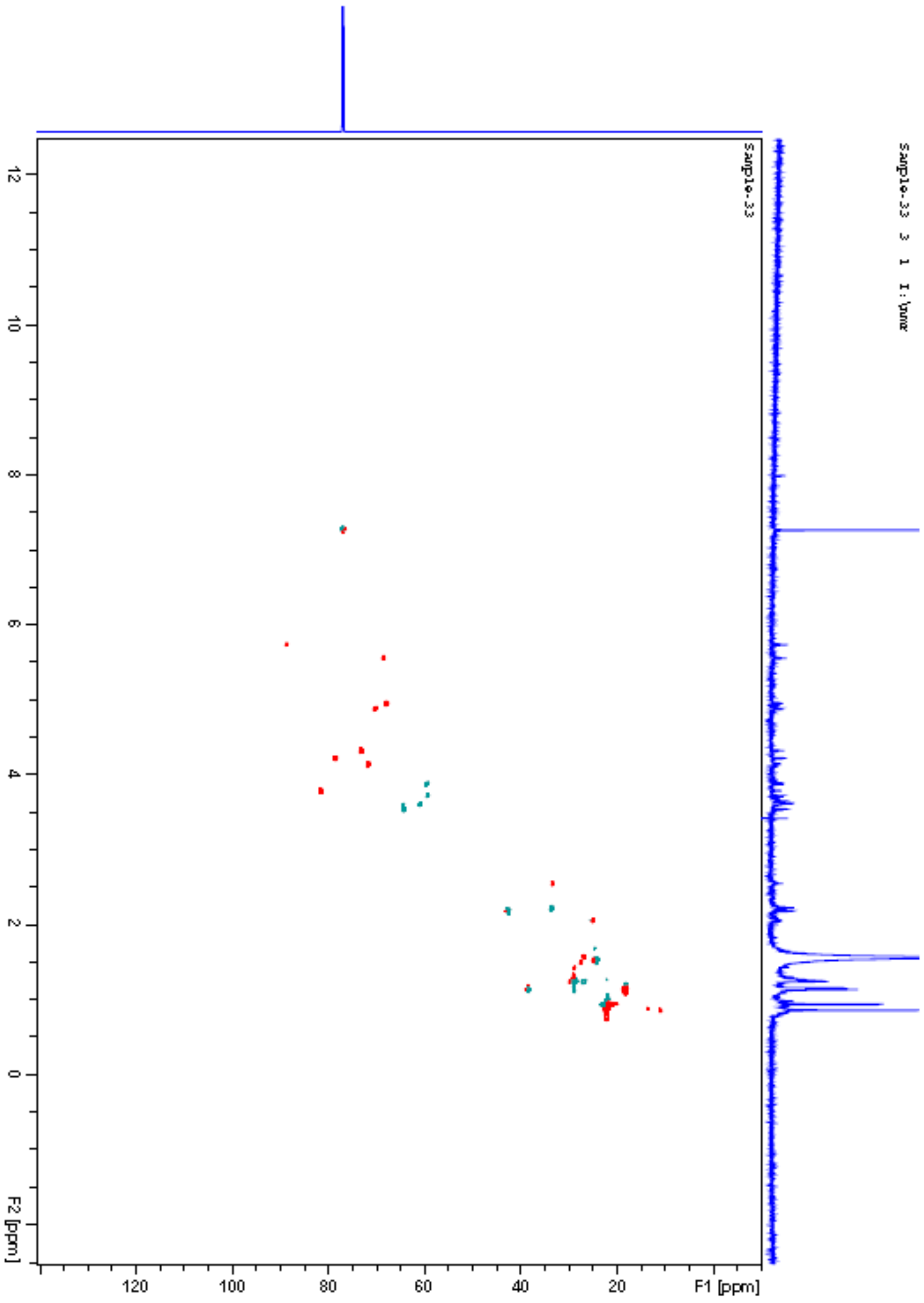
Sample-33
Sample-33 3 1 I:\nmr
F1 [ppm]
F2 [ppm]

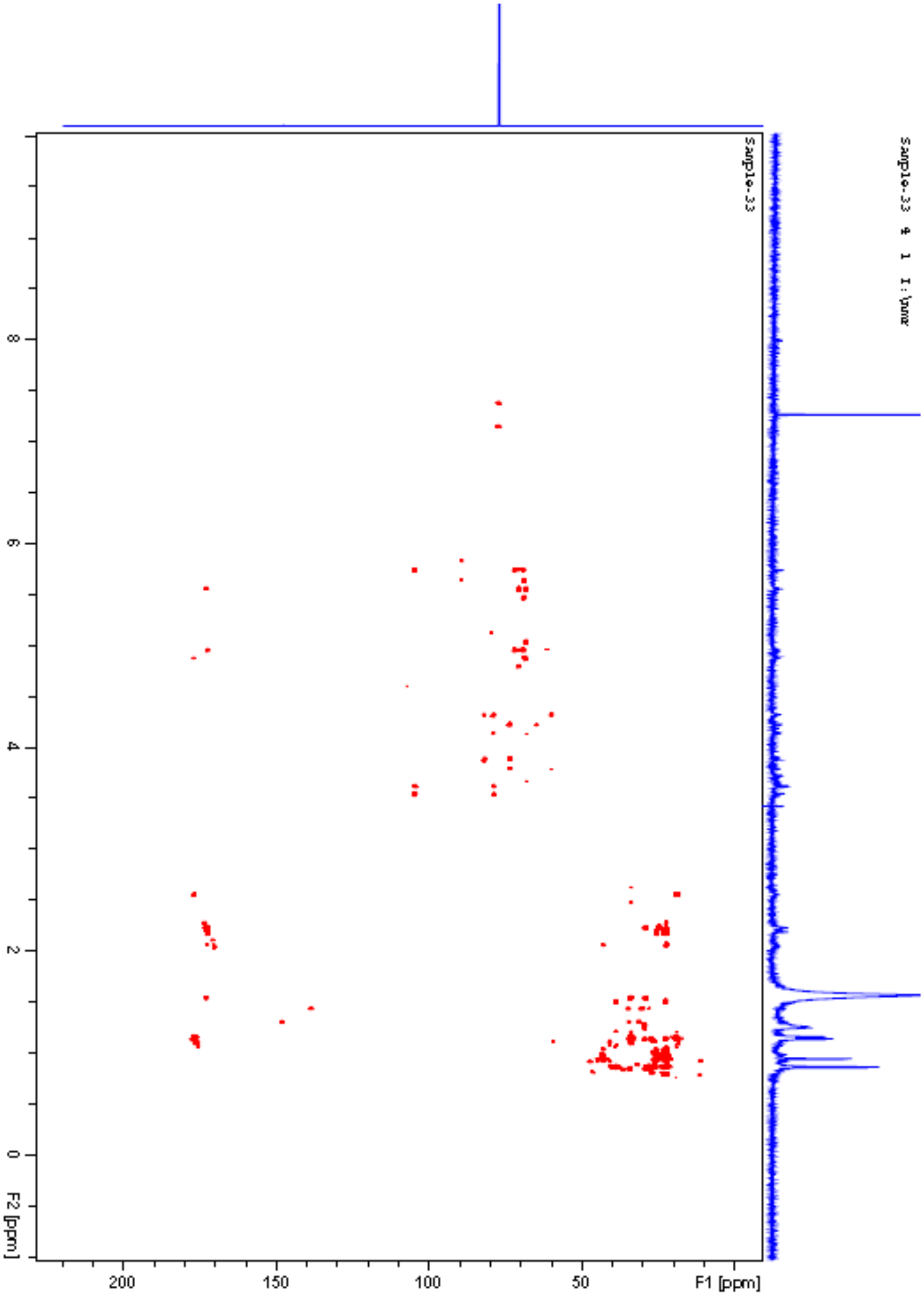

Sample-33
F2 [ppm]
F1 [ppm]

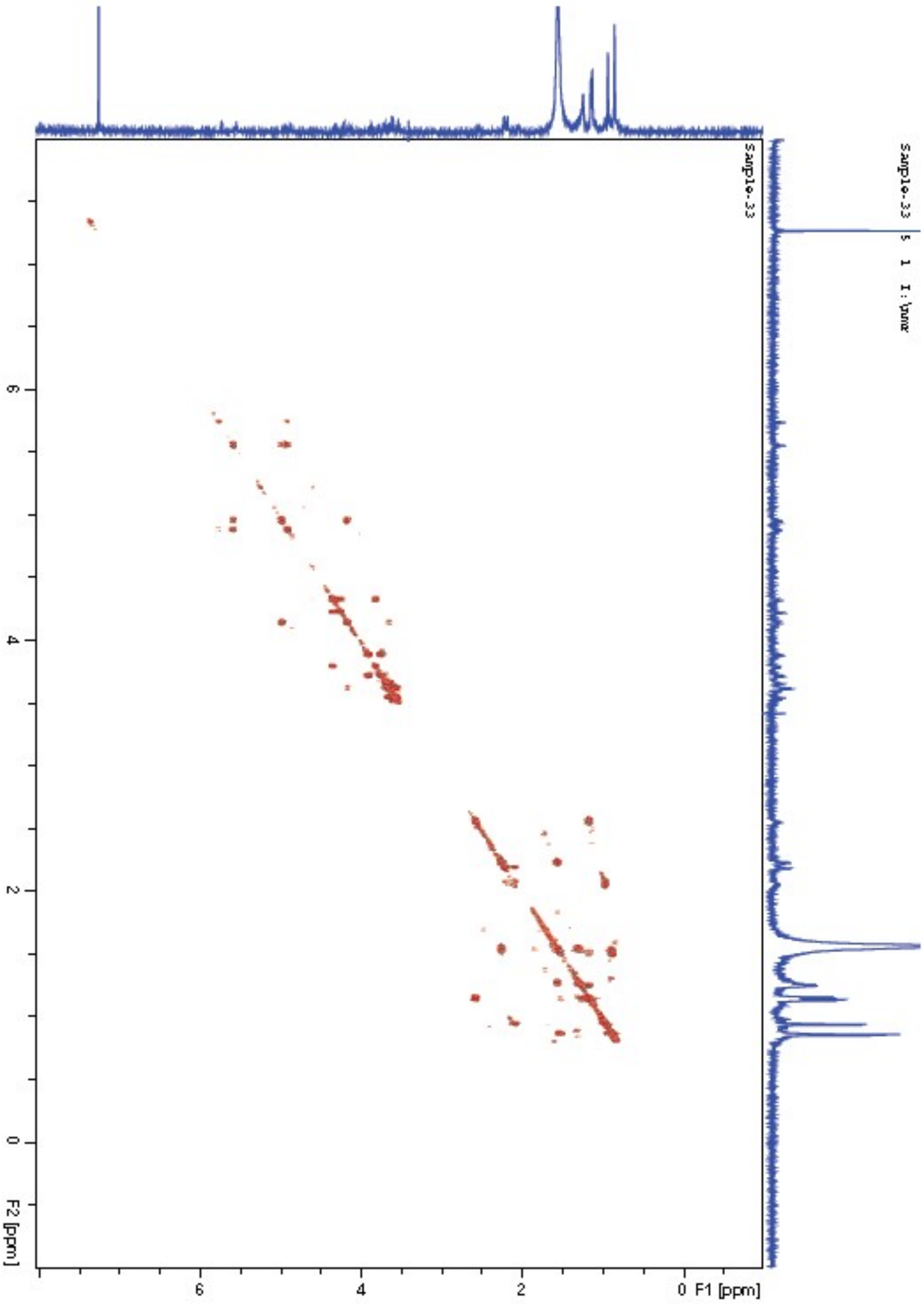
Sample-33
F1 [ppm]
F2 [ppm]

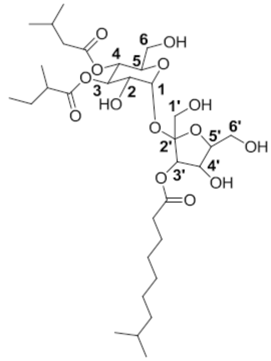

S3:20[4] (5,5,10)

Purified from *S. habrochaites* LA1392

HRMS: (ESI) *m/z* calcd for $C_{33}H_{57}O_{16}^-$ ([M+HCOO$^-$]): 709.3652, found: 709.3716

Material recovered: 2-3 mg

NMR solvent: $CDCl_3$

InChI Key: OWJQWPJHYPGVFC-DHFKECNASA-N

Carbon # (group)	^{1}H (ppm)	^{13}C (ppm)
1(CH)	5.49 (d, J = 3.3 Hz)	92.0 ($^1J_{CH}$ = 175.5 Hz)
2(CH)	3.71[c]	70.9
3(CH)	5.23 (t, J = 10.0)	72.4
3-O-		
-1(CO)		177.2
-2(CH)	2.34 (quin, J = 7.0 Hz)	41.0
-2'(CH_3)	1.10 (d, J = 7.0)	16.4
-3(CH_2)	1.47 (m), 1.66 (m)	26.7
-4(CH_3)	0.90 (t, J = 7.6 Hz)	11.4
4(CH)	4.93 (t, J = 9.9 Hz)	67.7
4-O		
-1(CO)		172.8
-2(CH_2)	2.17 (dd, J = 15.0, 7.1 Hz), 2.19 (dd, J = 15.0, 7.1 Hz)[a]	43.0
-3(CH)	2.06 (m)	25.4
-4(CH_3) x 2	0.95 (d, J = 6.1 Hz)	22.4, 22.4
5(CH)	4.06 (m, J = 5.2 Hz)	72.9
6(CH_2)	3.59 (m), 3.63 (m)	61.4
1' (CH_2)	3.65[b], 3.73 (d, J = 13.0)	64.9
2' (C)		103.6

3' (CH)	5.04 (d, J = 8.0 Hz)	81.4
3'-O		
-1(CO)		176.3
-2(CH_2)	2.52 (m)	34.2
-3(CH_2)	1.73 (quin, J = 7.7 Hz)	24.9
-4(CH_2)	1.38 (m)	29.2
-5(CH_2)	1.30 (m)	29.5
-6(CH_2)	1.30 (m)	27.3
-7(CH_2)	1.16[c]	39.0
-8(CH)	1.52 (m)	28.0
-9(CH_3) x 2	0.86 (d, J = 6.6 Hz)	22.7, 22.7
4' (CH)	4.65 (t, J = 8.2 Hz)	71.4
5' (CH)	3.97 (d, J = 8.1 Hz)	81.9
6' (CH_2)	3.70[b], 3.90 (d, J = 12.8 Hz)	59.6

[a]Higher order multiplet derived from the constants using gNMR

[b]Determined by COSY and HSQC

[c]Determined by COSY.

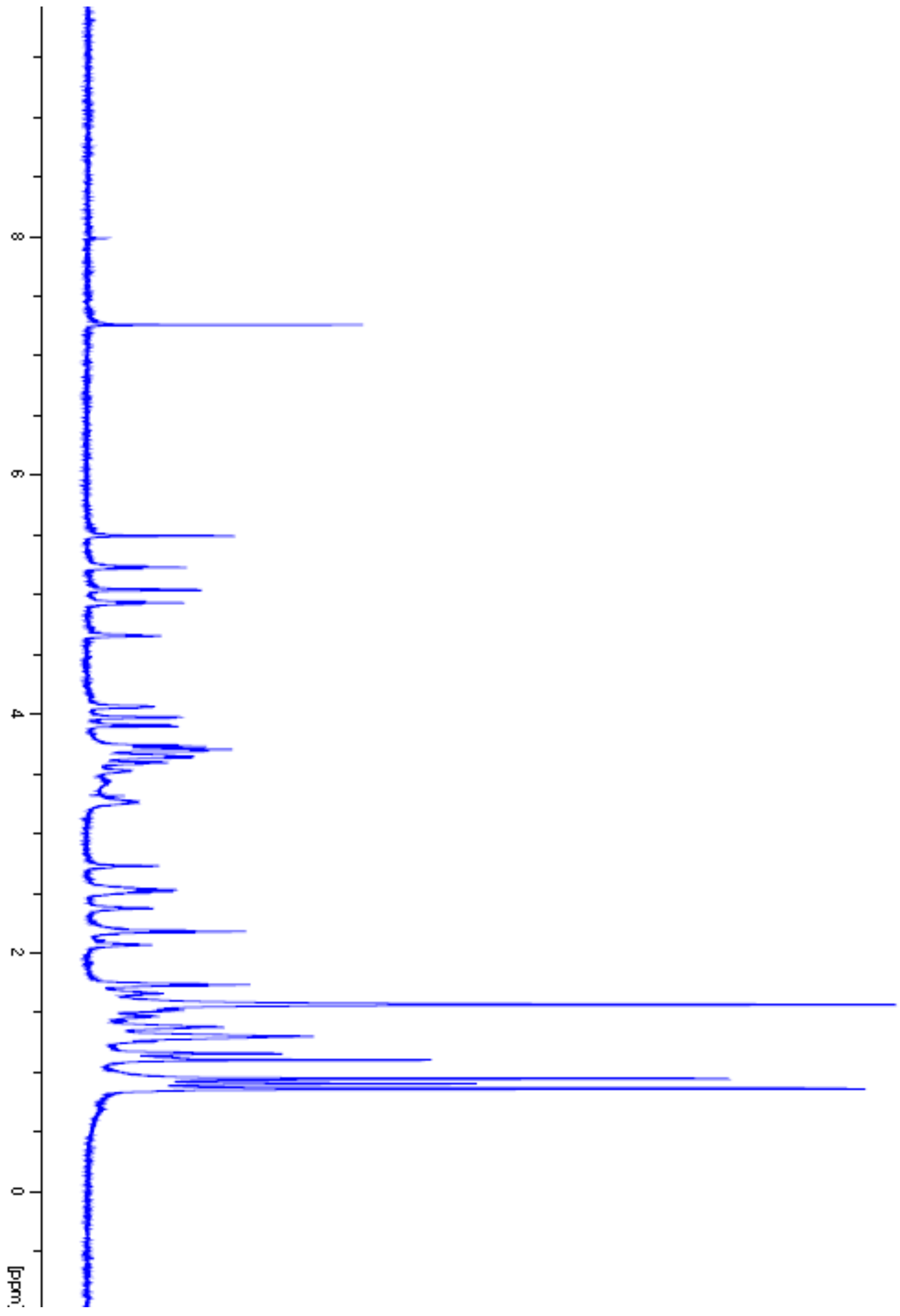
8
6
4
2
0
[ppm]

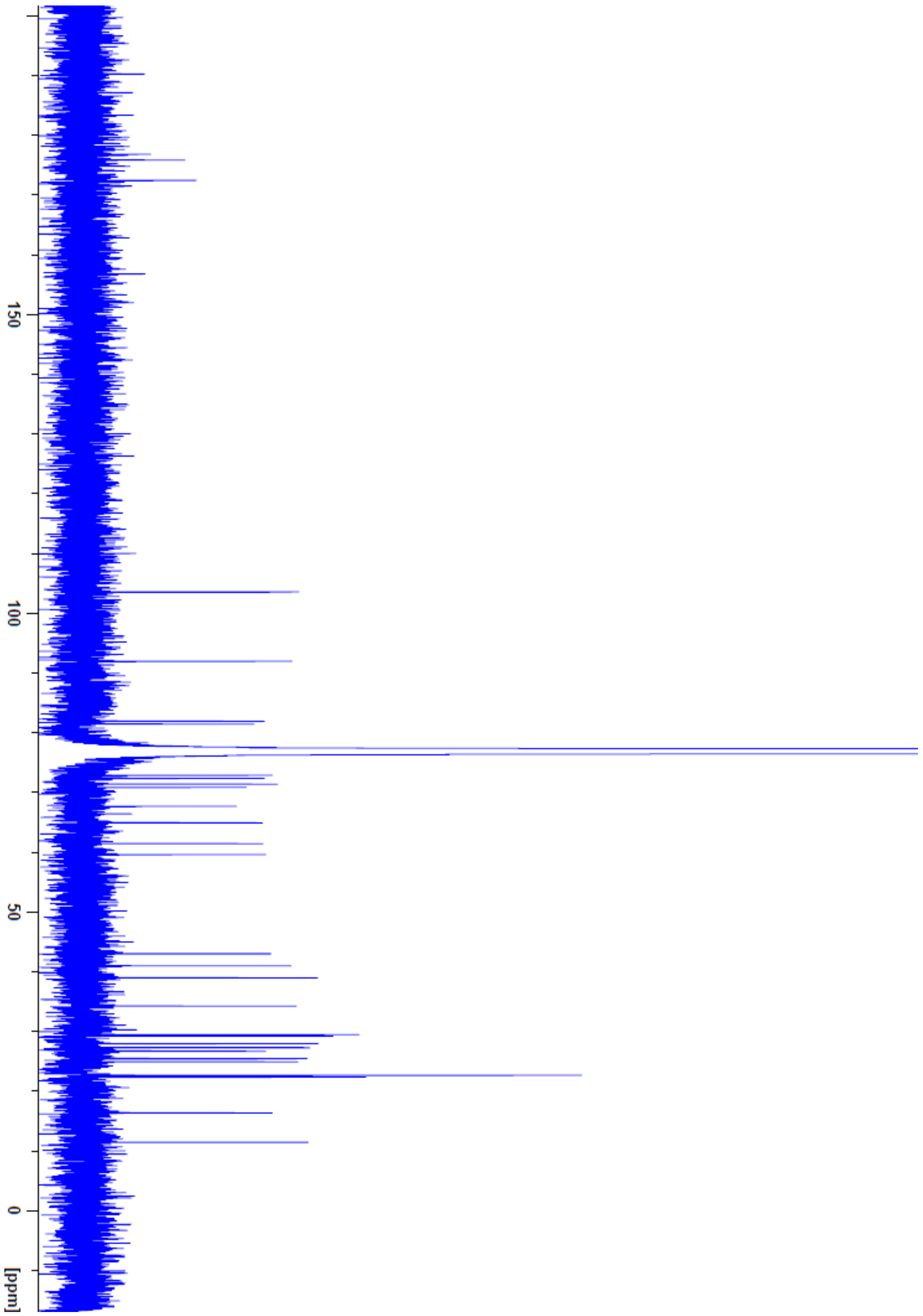
150
100
50
0
[ppm]

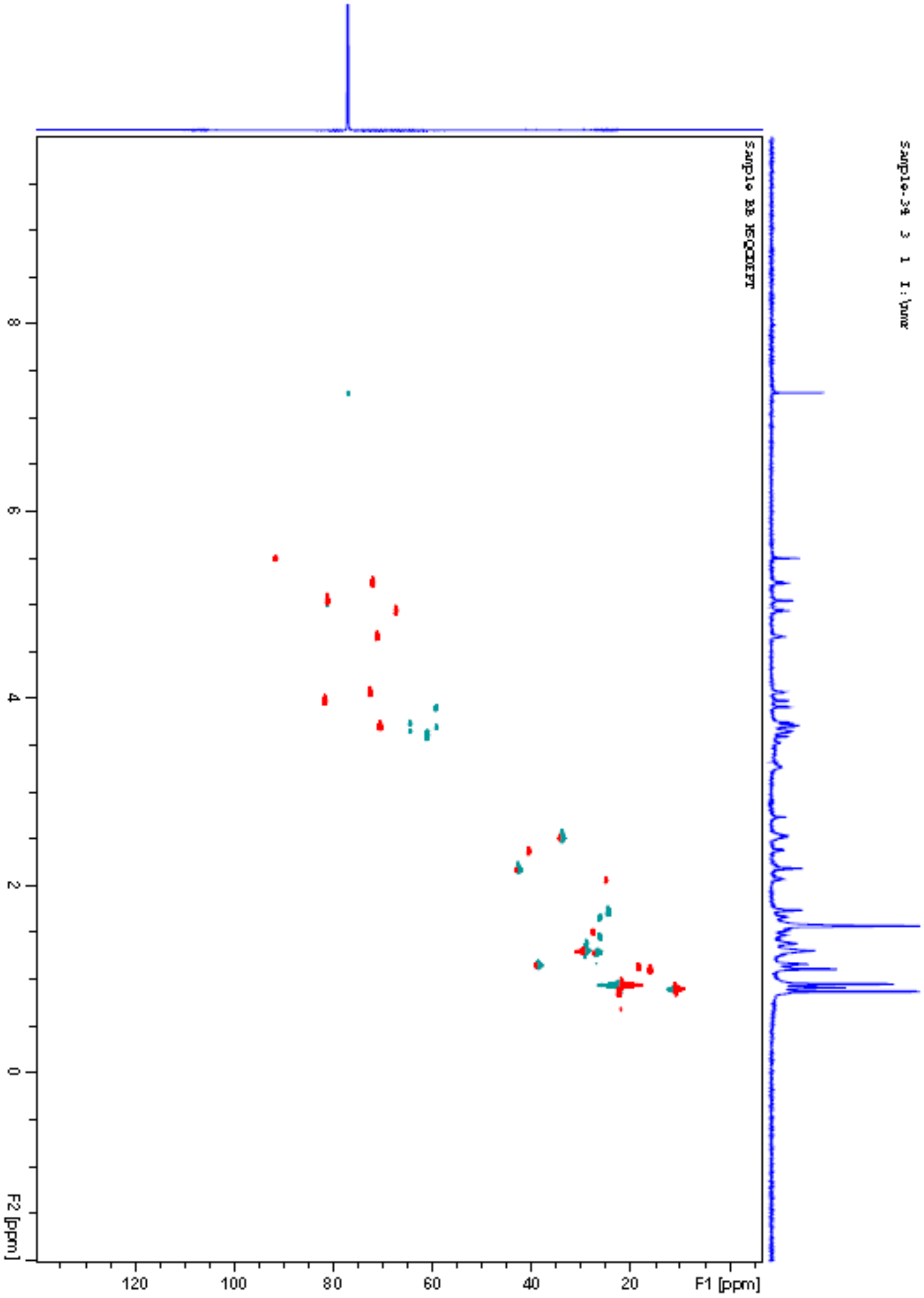
F1 [ppm]
F2 [ppm]

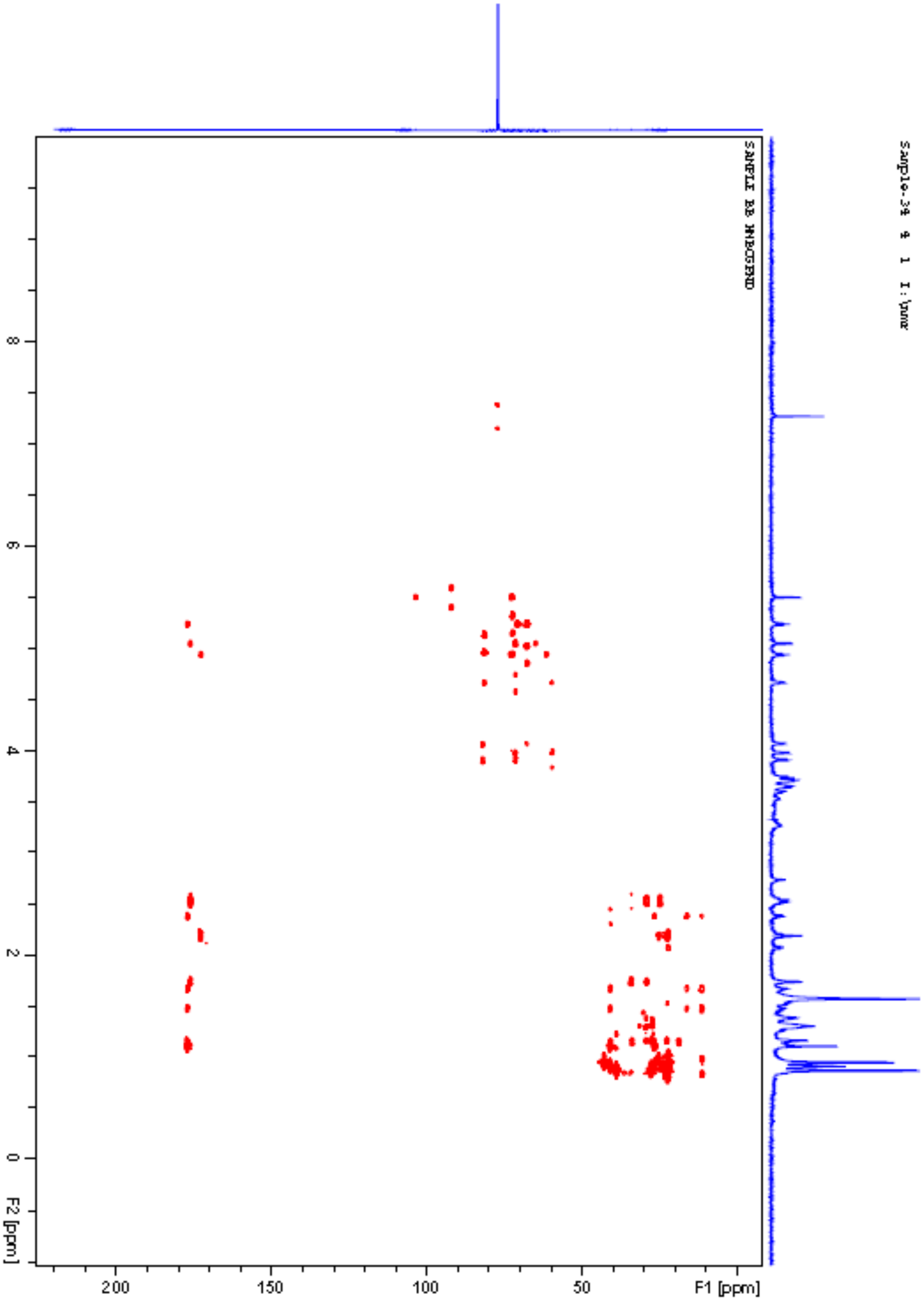
F1 [ppm]
F2 [ppm]

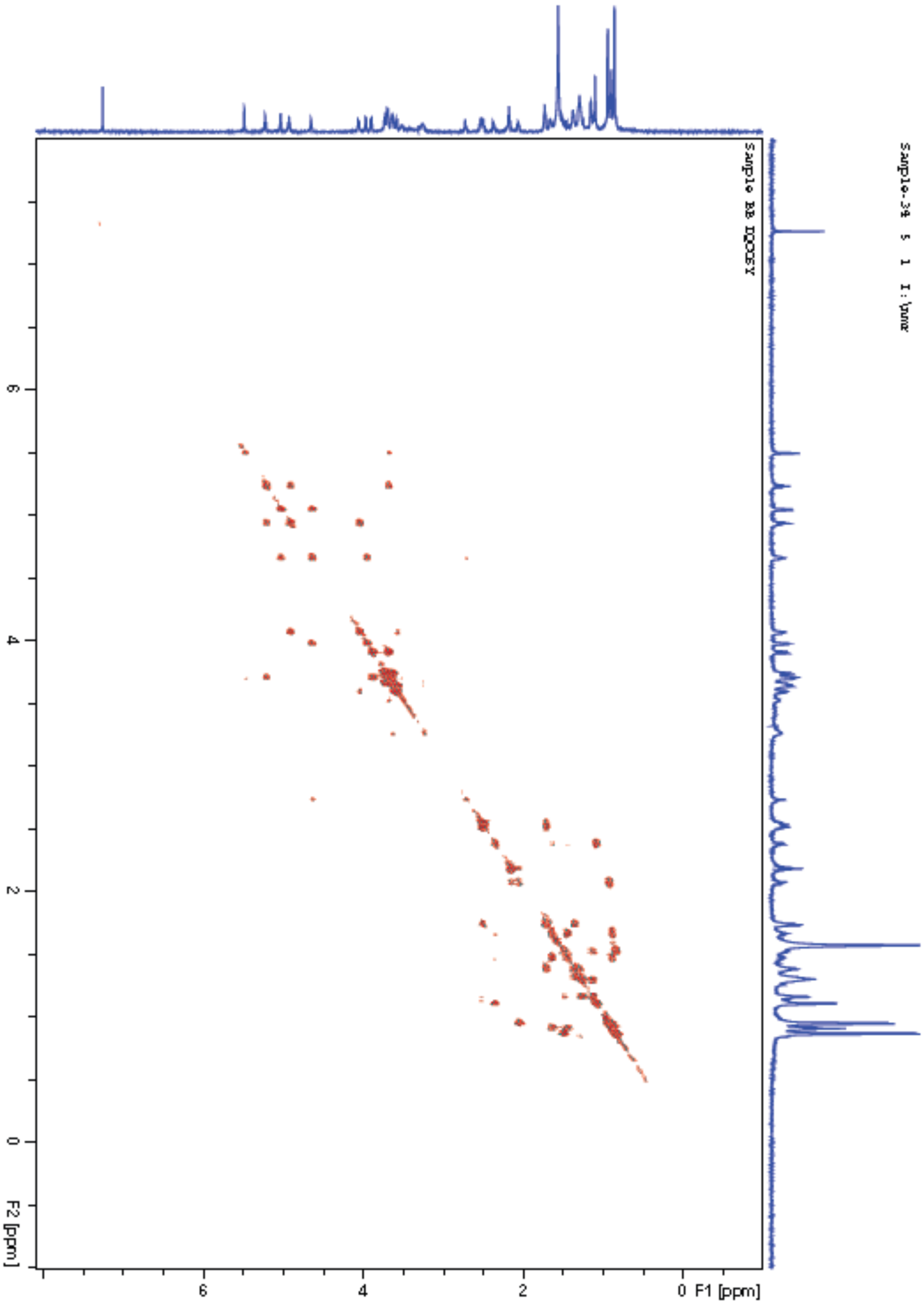
F2 [ppm]
F1 [ppm]

S3:21[1] (5,5,11)

Purified from *S. habrochaites* LA1392

HRMS: (ESI) *m/z* calcd for $C_{34}H_{59}O_{17}^-$ ([M+HCOO$^-$]): 723.3809, found: 723.3868

Material recovered: ~1 mg

NMR solvent: $CDCl_3$

InChI Key: UJJUTTYKTJCHGB-FSKBNNAPSA-N

Carbon # (group)	^{1}H (ppm)	^{13}C (ppm)
1(CH)	5.49 (br. s)	92.0 ($^1J_{CH}$ = 176.8 Hz)
2(CH)	3.70[a]	70.9
3(CH)	5.22 (t, *J* = 9.9 Hz)	72.4
3-O-		
-1(CO)		177.0
-2(CH)	2.37 (m)	40.9
-2'(CH_3)	1.10 (d, *J* = 6.9 Hz)	16.4
-3(CH_2)	1.47 (m), 1.67 (*m*)	26.7
-4(CH_3)	0.91 (t, *J* = 7.2 Hz)	11.4
4(CH)	4.93 (t, *J* = 10.0 Hz)	67.7
4-O		
-1(CO)		172.4
-2(CH_2)	2.18 (m)	43.0
-3(CH)	2.07 (m)	25.4
-4(CH_3) x 2	0.95 (br. m)	22.4
5(CH)	4.06 (m)	72.9
6(CH_2)	3.59 (m), 3.64 (m)	61.4
1' (CH_2)	3.65 (m)[b], 3.73 (d, *J* = 13.0 Hz)	64.9
2' (C)		103.6

3' (CH)	5.04 (d, J = 8.3 Hz)	81.4
3'-O		
-1(CO)		175.9
-2(CH_2)	2.52 (m)	34.2
-3(CH_2)	1.74 (m)	24.9
-4-5(CH_2-CH_2)	1.38 (br. s)	29.2, 29.5
-6(CH_2)	1.25[b], 1.31[b]	27.0
-7(CH_2)	1.08[b], 1.28[b]	36.6
-8(CH)	1.31[a]	34.4
-8'(CH_3)	0.85 (m)	19.2
-9(CH_2)	1.11 (m), 1.31(br. s)	29.6
-10(CH_3)	0.84 (m)	11.4
4' (CH)	4.66 (m)	71.4
5' (CH)	3.97 (m)	81.9
6' (CH_2)	3.70 (d, J = 13.5 Hz), 3.90 (d, J = 13.5 Hz)	59.6

[a]Determined by COSY

[b]Determined by COSY and HSQC.

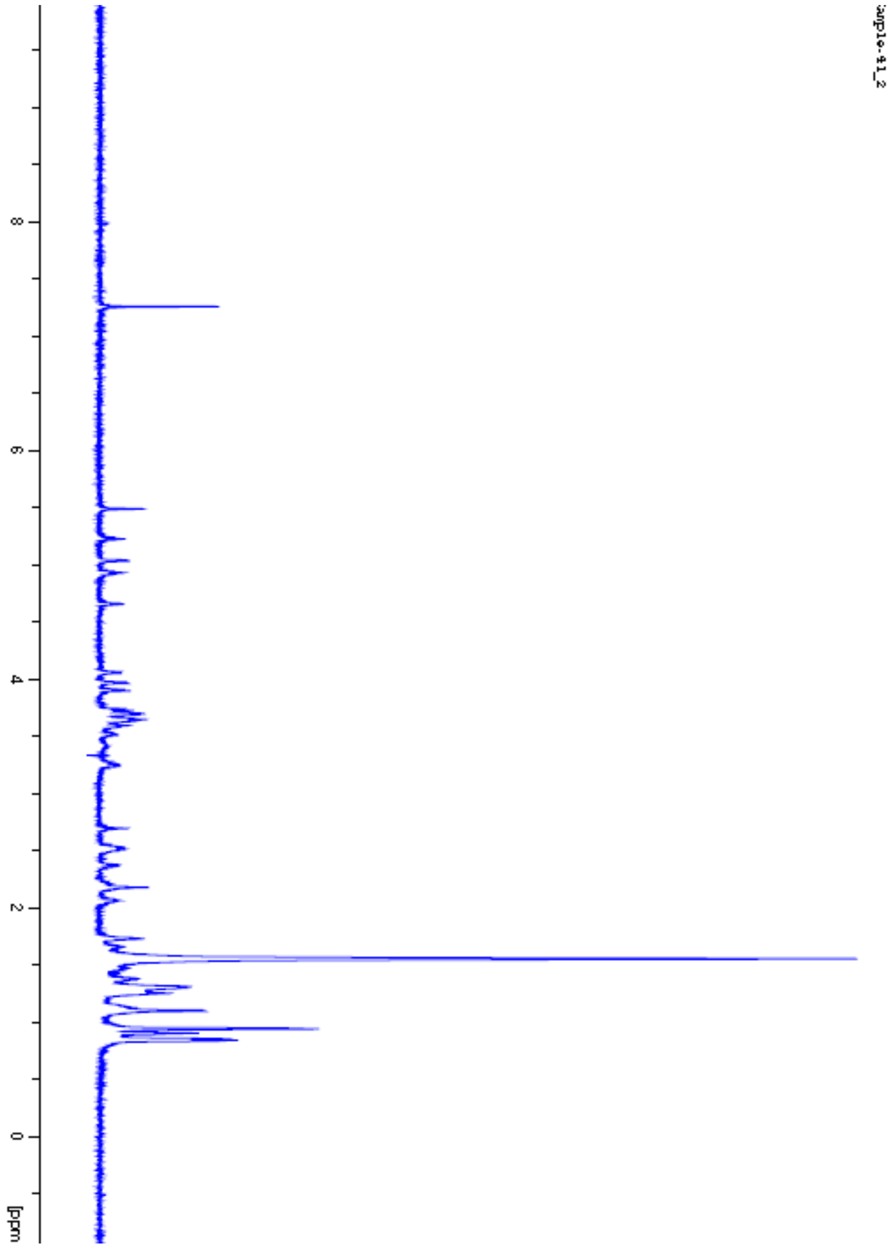
sample-41_2
8
6
4
2
0
[ppm

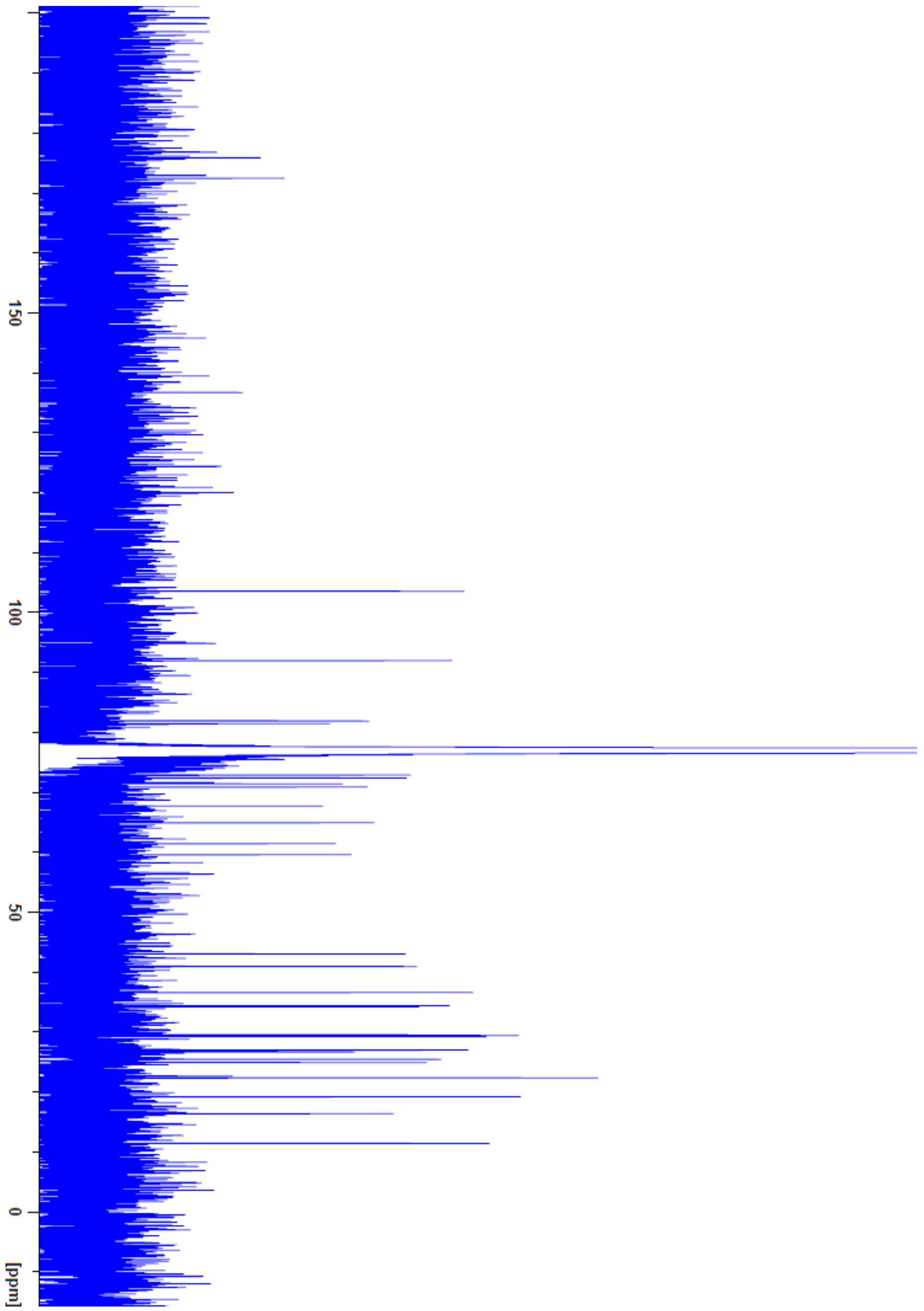
150
100
50
0
[ppm]

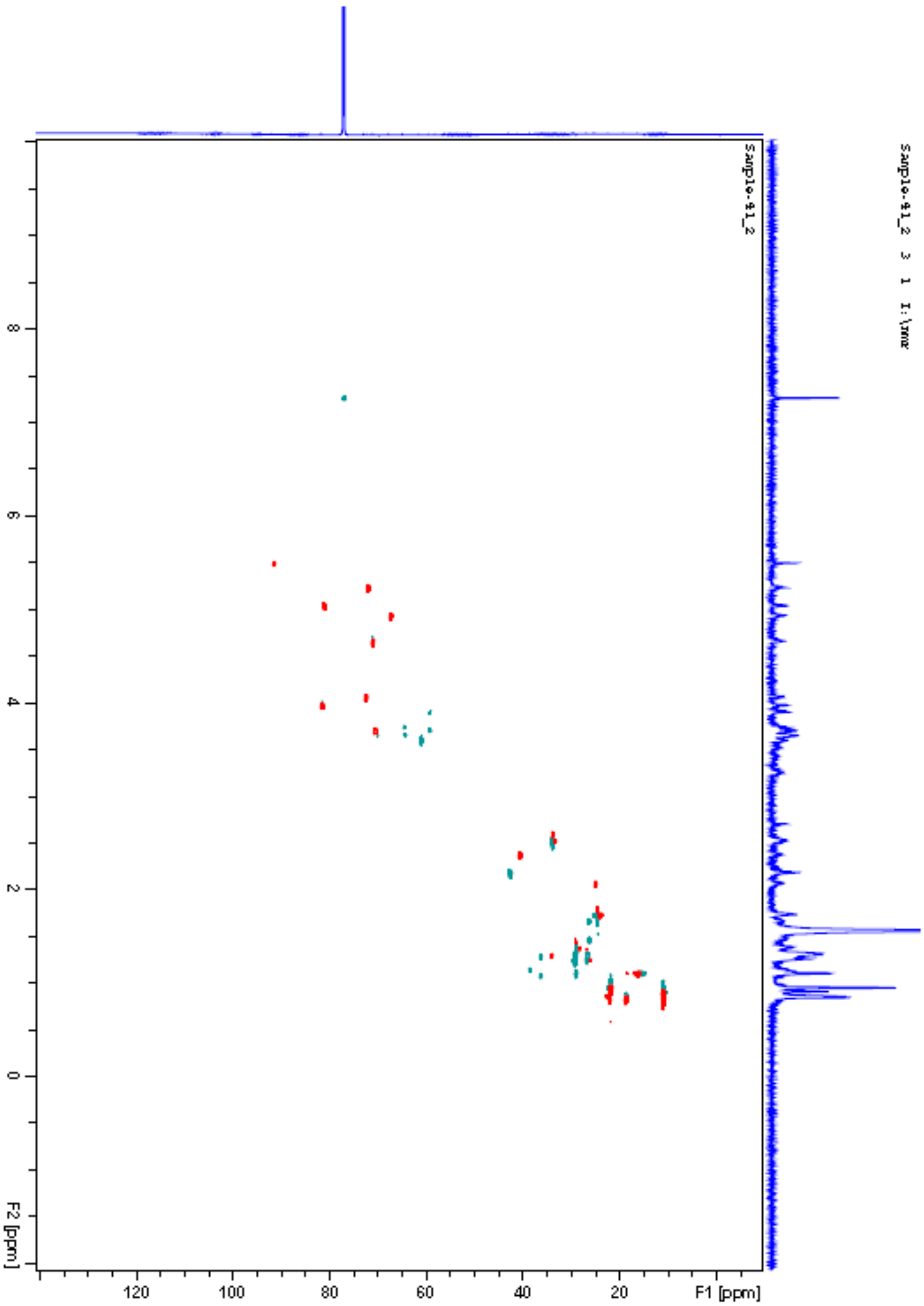
Sample-41_2
Sample-41_2 3 1 I:\nmr
F2 [ppm]
F1 [ppm]

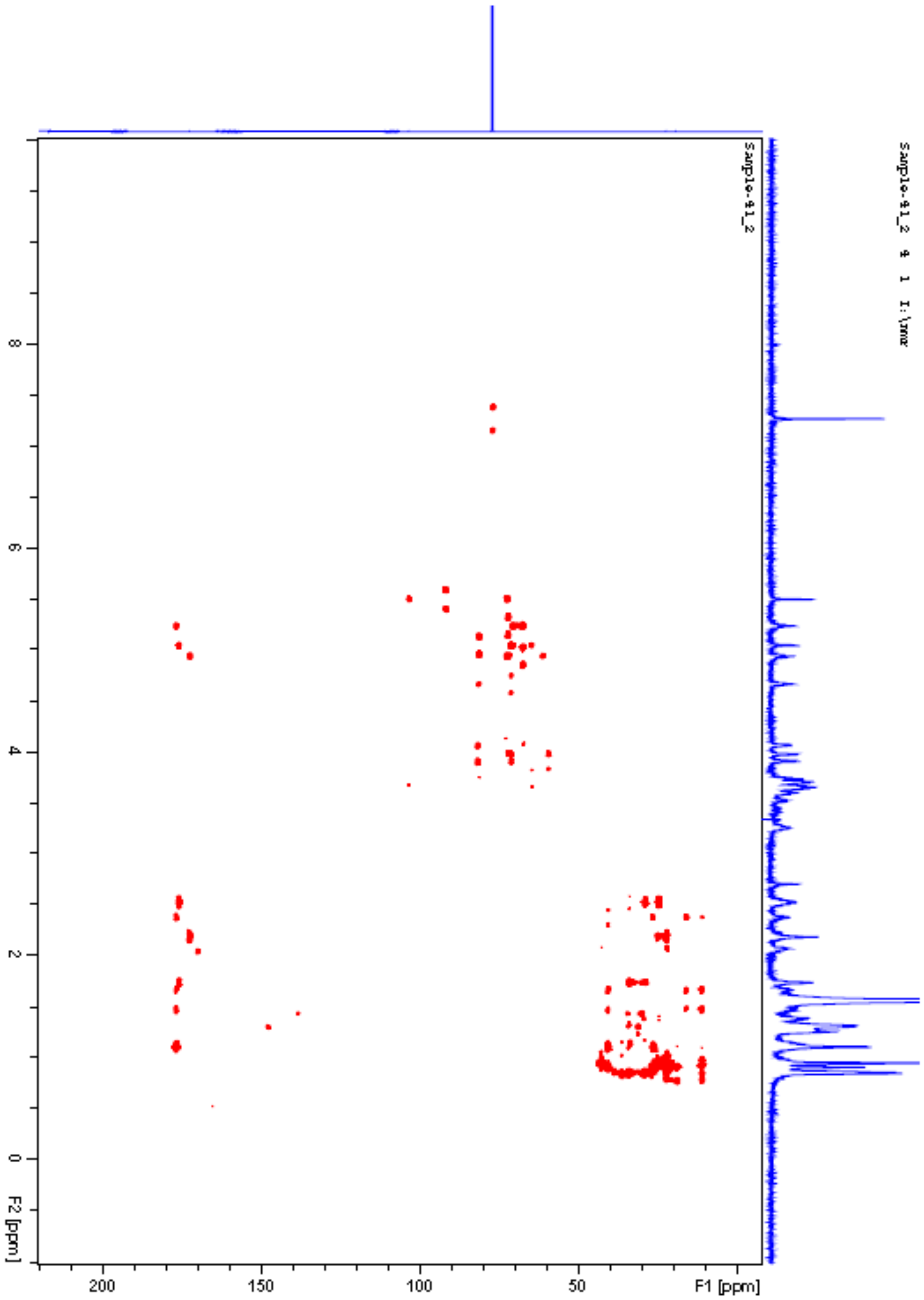
Sample-41_2
Sample-41_2 4 1 I:\nmr
F1 [ppm]
F2 [ppm]

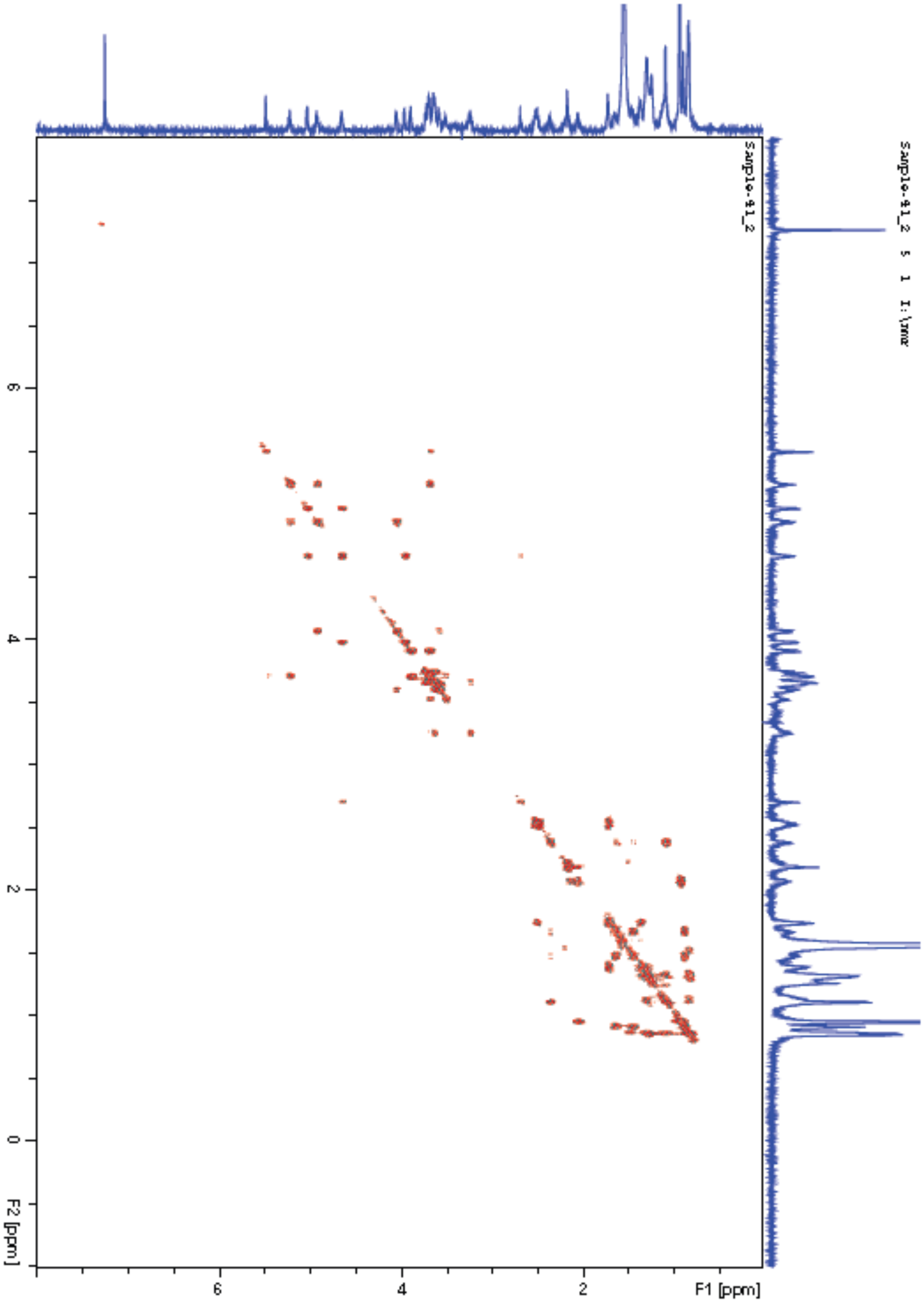
Sample-41_2
F1 [ppm]
F2 [ppm]

S3:21[5] (5,5,11)

Purified from *S. habrochaites* LA1392

HRMS: (ESI) *m/z* calcd for $C_{34}H_{59}O_{17}^{-}$ ([M+HCOO^{-}]): 723.3809, found: 723.3917

Material recovered: 1-2 mg

NMR solvent: $CDCl_3$

InChI Key: NOKSZAJXUWWURN-MGUFFPLASA-N

Carbon # (group)	^{1}H (ppm)	^{13}C (ppm)
1(CH)	5.75 (d, J = 3.7 Hz)	89.1 ($^{1}J_{CH}$ = 177.1 Hz)
2(CH)	4.87 (dd, J = 10.2, 3.8 Hz)	70.6
2-O-		
-1(CO)		176.6
-2(CH)	2.40 (m)	40.5
-2'(CH_3)	1.13 (d, J = 6.5 Hz)	15.9
-3(CH_2)	1.45 (m), 1.63[a]	26.9
-4(CH_3)	0.87 (m)	11.4
3(CH)	5.55 (t, J = 10.0 Hz)	68.9
3-O-		
-1(CO)		172.7
-2(CH_2)	2.21 (m)	34.3
-3(CH_2)	1.53 (m)	24.7
-4-5(CH_2)	1.26 (m)[a]	29.2, 29.4
-6(CH_2)	1.21[b], 1.26[b]	26.9
-7(CH_2)	1.05[b], 1.25[b]	36.5
-8(CH)	1.27[a]	34.2
-8'(CH_3)	0.83 (m)	19.2

-9(CH_2)	1.06 (m), 1.25[b]	36.5
-10(CH_3)	0.85 (m)	11.4
4(CH)	4.94 (t, *J* = 10.0 Hz)	68.4
4-O		
-1(CO)		172.3
-2(CH_2)	2.20 (m)	43.0
-3(CH)	2.05 (m)	25.5
-4(CH_3) x 2	0.94 (d, *J* = 6.4 Hz)	22.3
5(CH)	4.16 (m)	71.9
6(CH_2)	3.61 (m), 3.65 (d, *J* = 12.6 Hz)	61.4
1' (CH_2)	3.53 (d, *J* = 11.8 Hz), 3.61 (m)	64.6
2' (C)		104.5
3' (CH)	4.22 (d, *J* = 8.0 Hz)	78.8
4' (CH)	4.32 (t, *J* = 8.2 Hz)	73.4
5' (CH)	3.77 (d, *J* = 7.5 Hz)	81.7
6' (CH_2)	3.71 (d, *J* = 13.0 Hz), 3.88 (d, *J* = 13.0 Hz)	59.9

[a]Determined by COSY

[b]Determined by COSY and HSQC

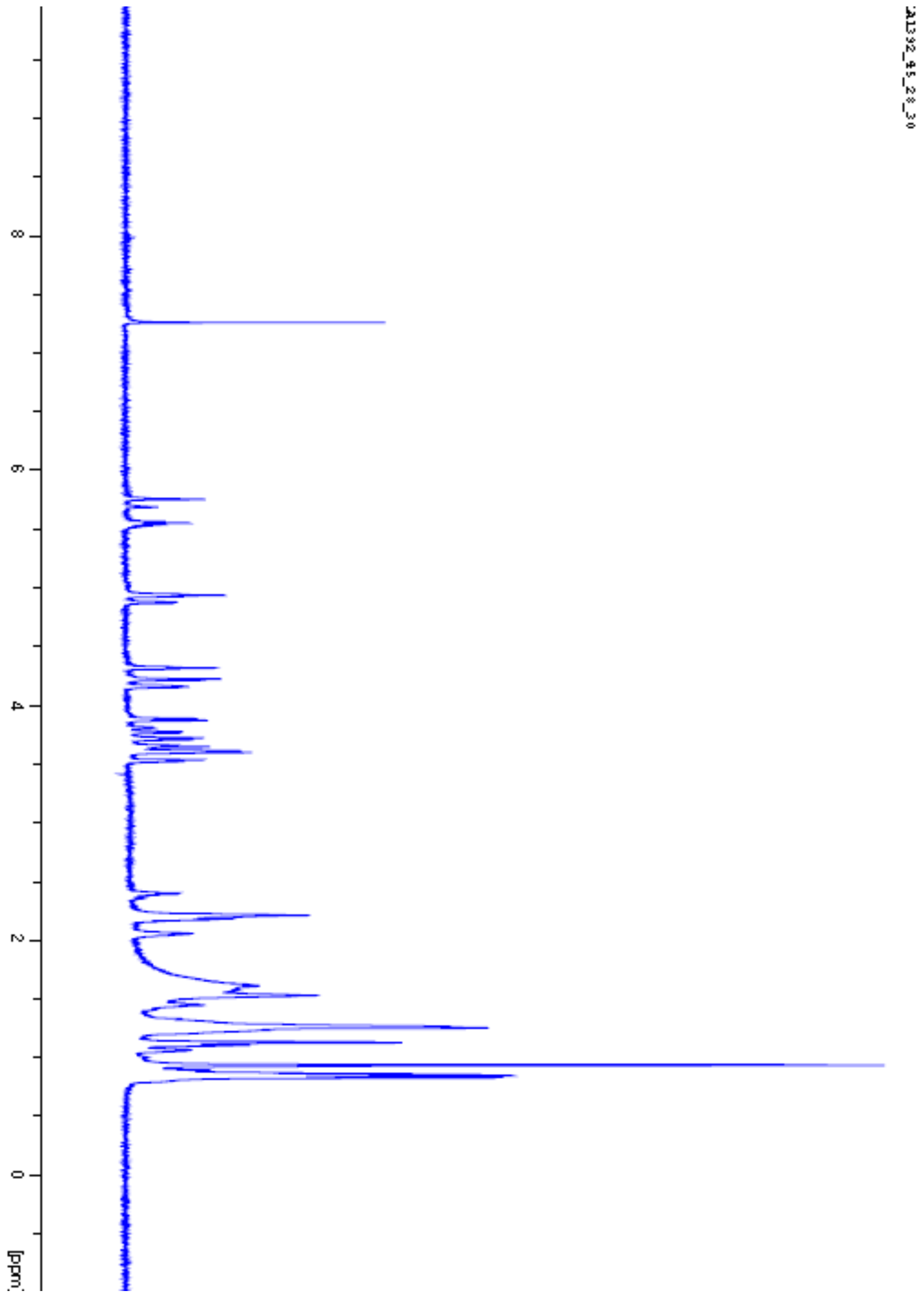
A1392_45_28_30
8
6
4
2
0
[ppm]

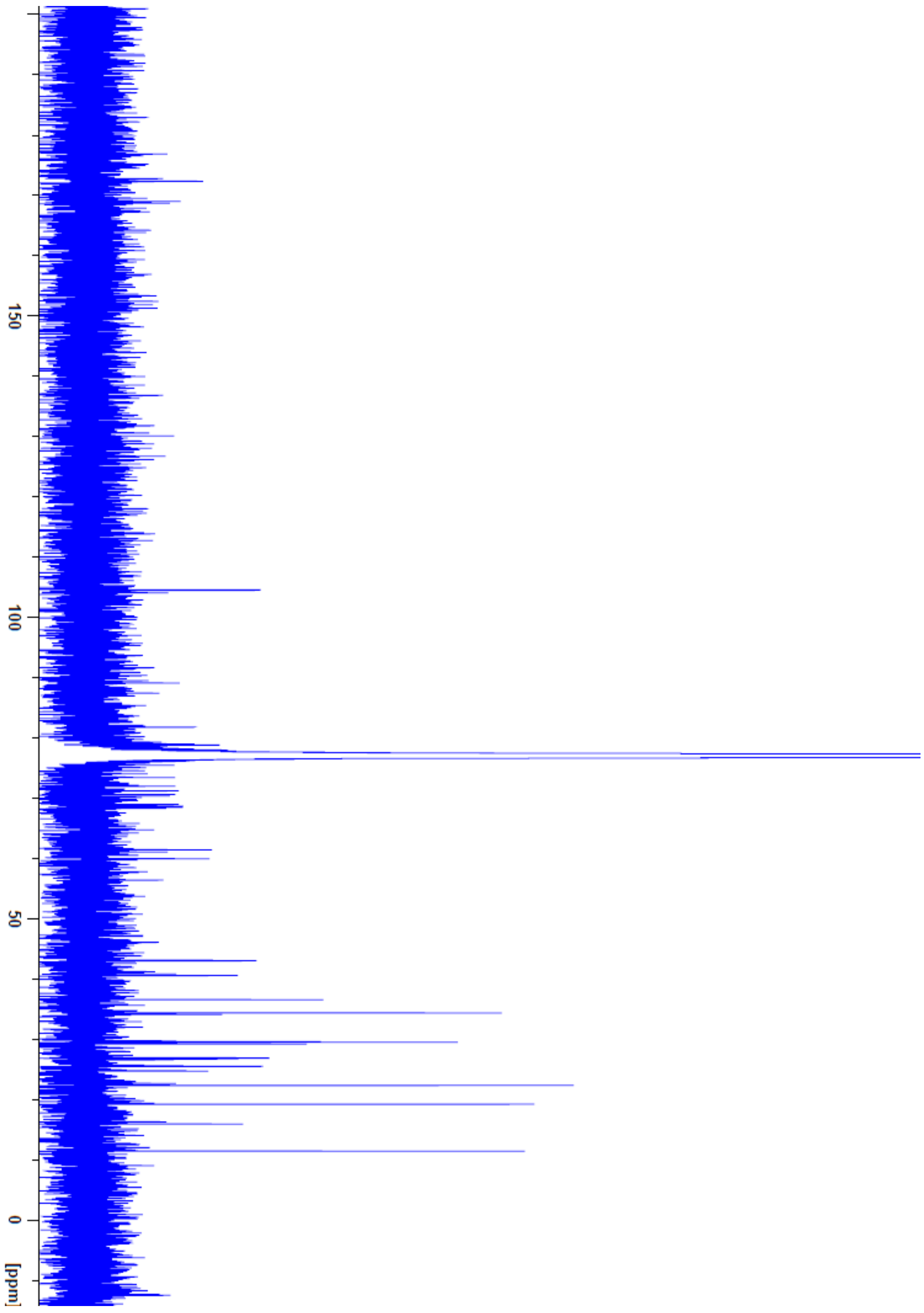
150
100
50
0
[ppm]

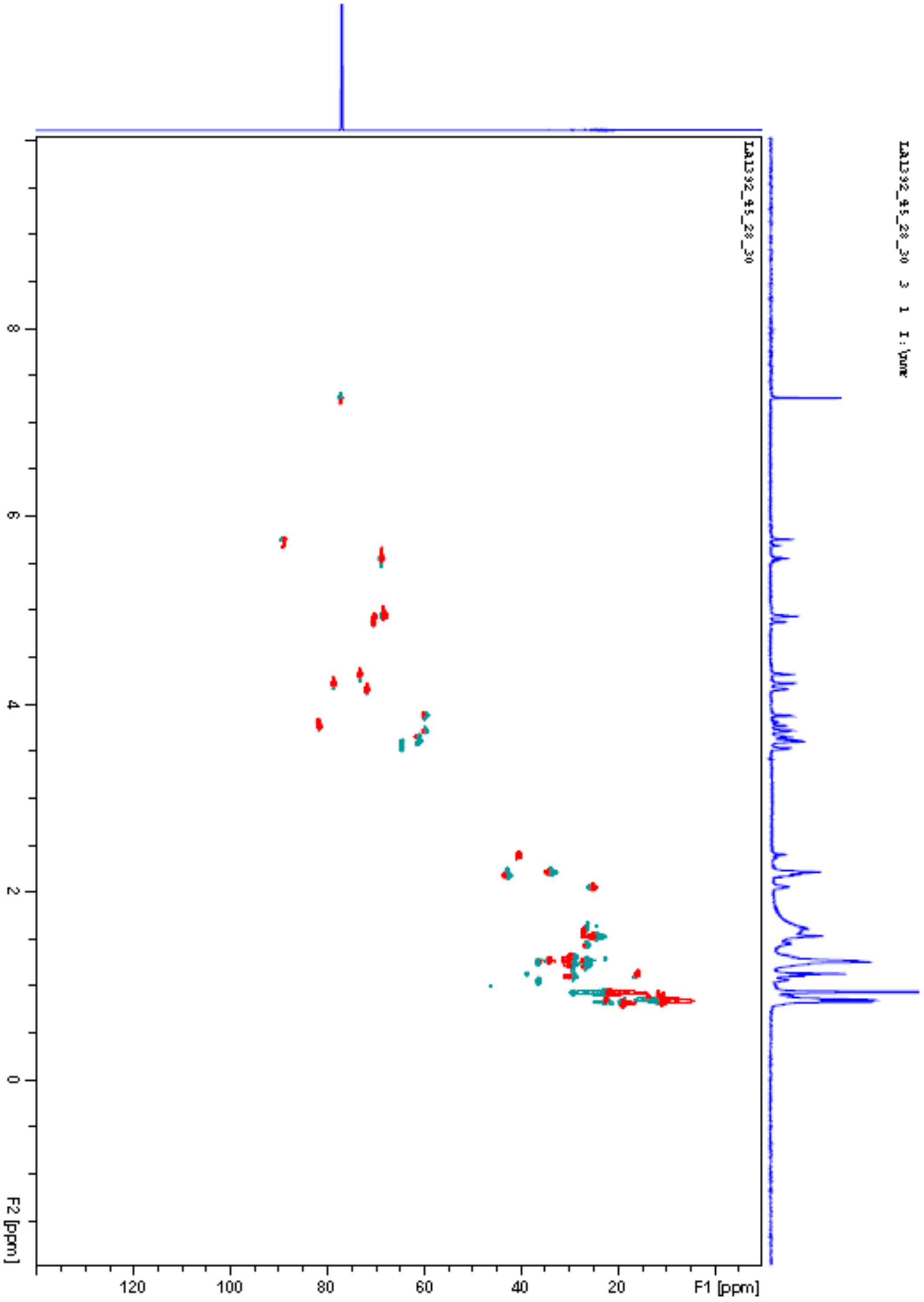
F1 [ppm]
F2 [ppm]
LA1392_45_28_30

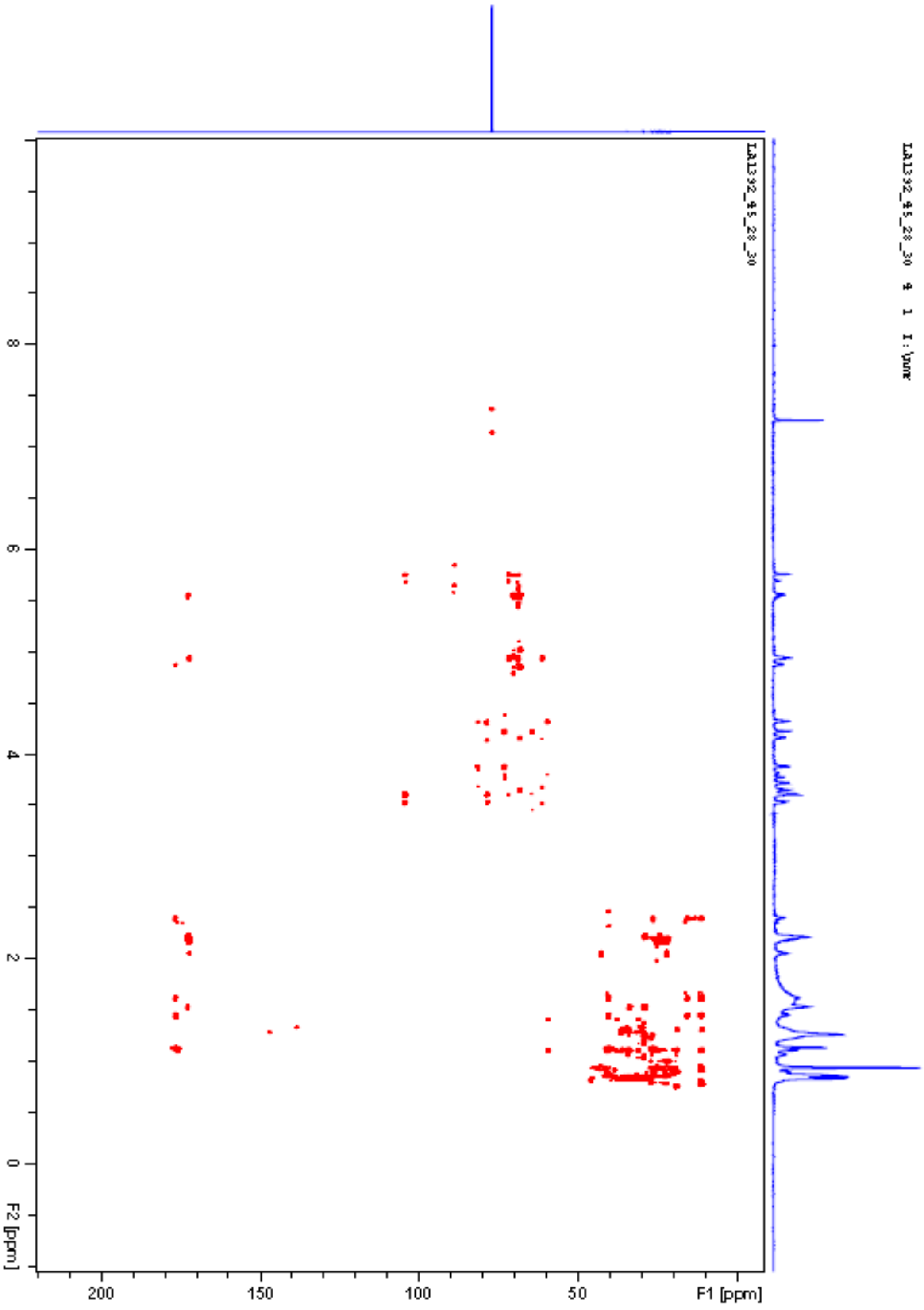
F1 [ppm]
F2 [ppm]
LA1392_45_28_30

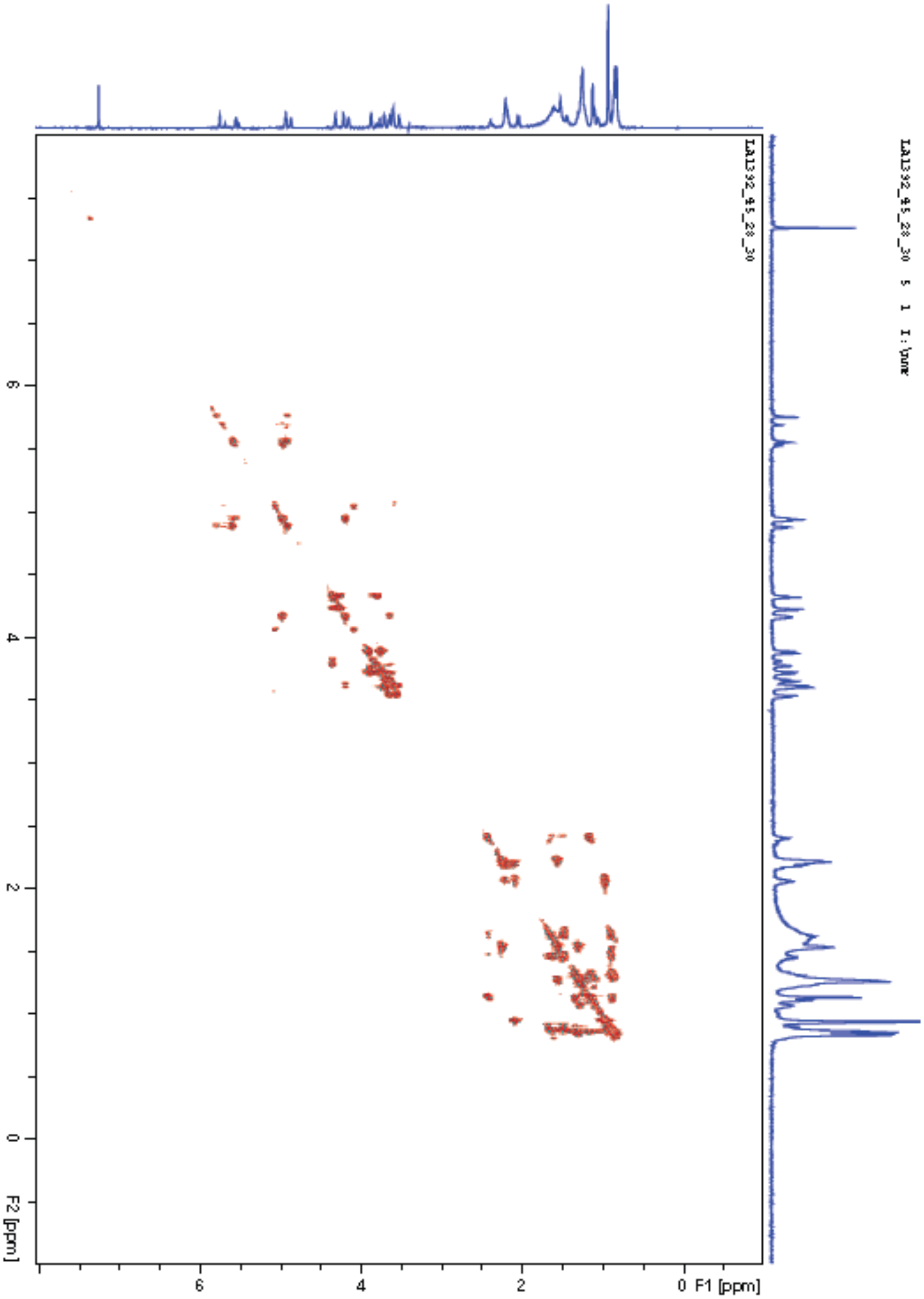
F2 [ppm]
F1 [ppm]
0
2
4
6

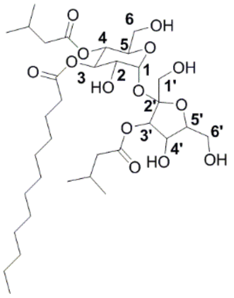

S3:22[4] (5,5,12)

Purified from *S. lycopersicum* M82

HRMS: (ESI) *m/z* calcd for $C_{35}H_{61}O_{16}^-$ ([M+HCOO$^-$]): 737.3965, found: 737.3903

Material recovered: ~ 1 mg

NMR solvent: $CDCl_3$

InChi Key: HZYKVDFNYAKASX-XBKHNYQHSA-N

Carbon # (group)	^{1}H (ppm)	^{13}C (ppm)
1(CH)	5.51 (d, J = 3.8 Hz)	91.8 ($^1J_{CH}$ = 179.7 Hz)
2(CH)	3.71 (m)[a]	70.7
3(CH)	5.22 (t, J = 9.7 Hz)	72.7
3-O-		
-1(CO)		173.8
-2(CH_2)	2.32(m)	34.2
-3(CH_2)	1.57 (br. s)[a]	24.8
-4-10(CH_2-CH_2-CH_2-CH_2-CH_2-CH_2-CH_2-)	1.24-1.28 (br. s)[a]	29.1, 29.2, 29.3, 29.4, 29.6, 29.6, 31.9
-11(CH_2)	1.28[a]	22.7
-12(CH_3)	0.88 (t, J = 7.2 Hz)	14.1
4(CH)	4.90 (t, J = 9.9 Hz)	67.8
4-O		
-1(CO)		172.5
-2(CH_2)	2.19 (m)[a]	43.1
-3(CH)	2.06 (m)	25.5
-4(CH_3) x 2	0.94 (d, J = 6.6 Hz)	22.1

5(CH)	4.06 (m)	72.8
6(CH_2)	3.59 (dd, J = 13.3, 5.5 Hz), 3.63 (m)[a]	61.4
1' (CH_2)	3.65 (m), 3.74 (m)[a]	65.0
2' (C)		103.5
3' (CH)	5.05 (d, J = 8.1 Hz)	81.4
3'-O		
-1(CO)		175.2
-2(CH_2)	2.41 (m)	43.2
-3(CH)	2.20 (m)	25.9
-4(CH_3) x 2	1.04 (d, J = 6.6 Hz), 1.05 (d, J = 6.6 Hz)	22.6
4' (CH)	4.65 (ddd, J = 7.7, 3.7, 3.7 Hz)	71.3
5' (CH)	3.97 (m)	81.9
6' (CH_2)	3.72 (m), 3.91 (dd, J = 12.8, 1.9 Hz)[a]	59.6
[a]Determined by COSY and HSQC		

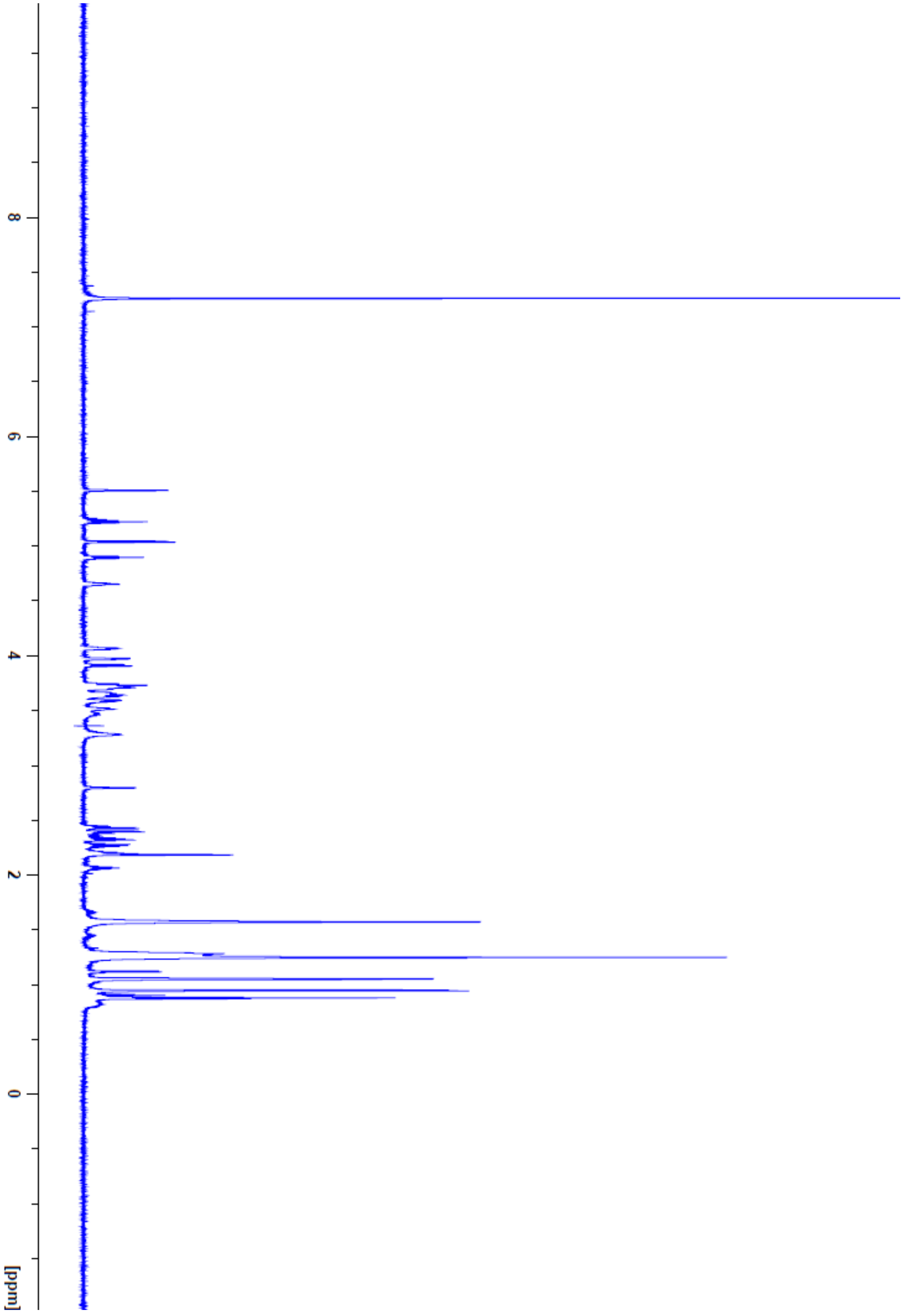
8
6
4
2
0
[ppm]

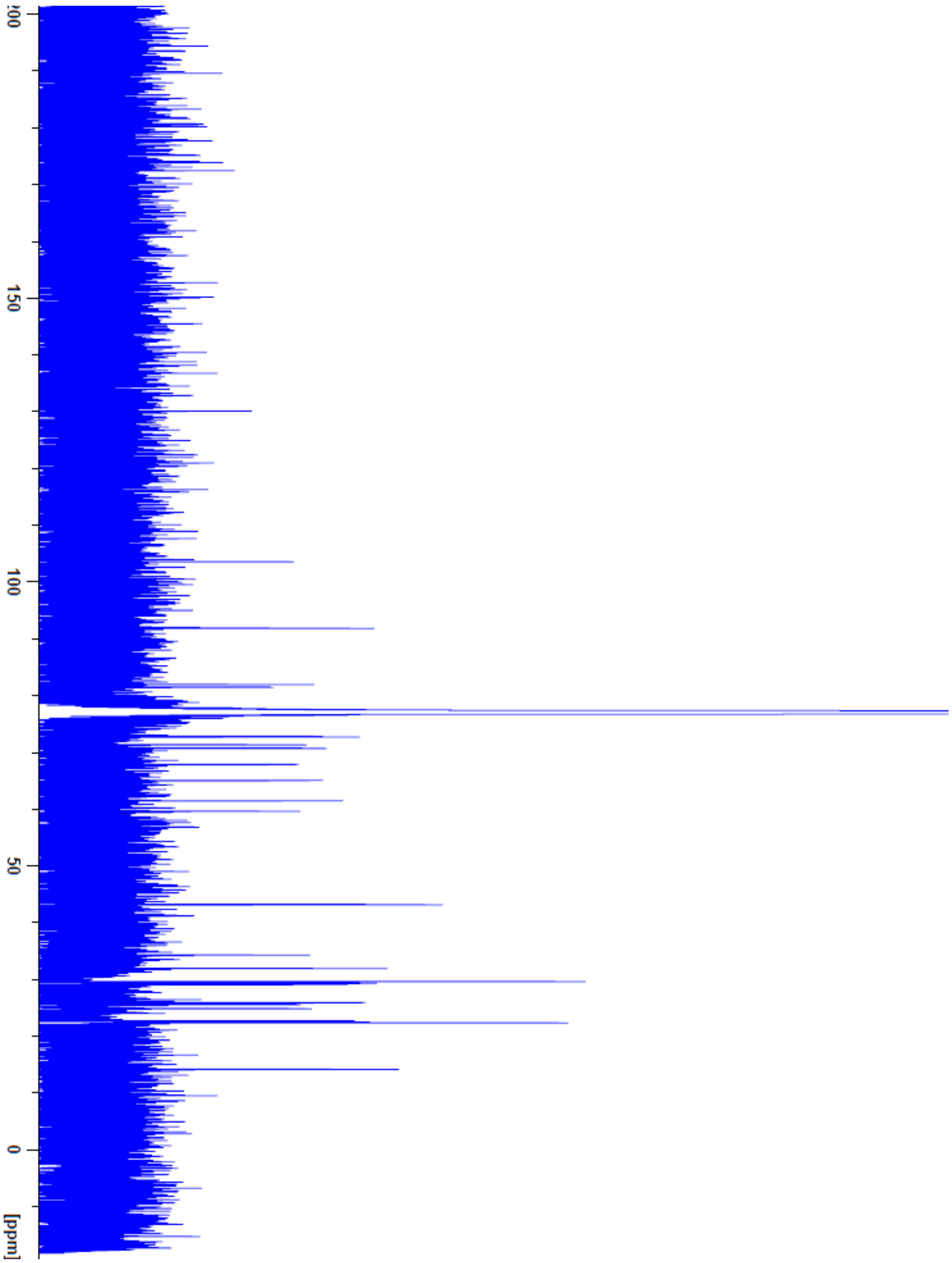
150
100
50
0
[ppm]

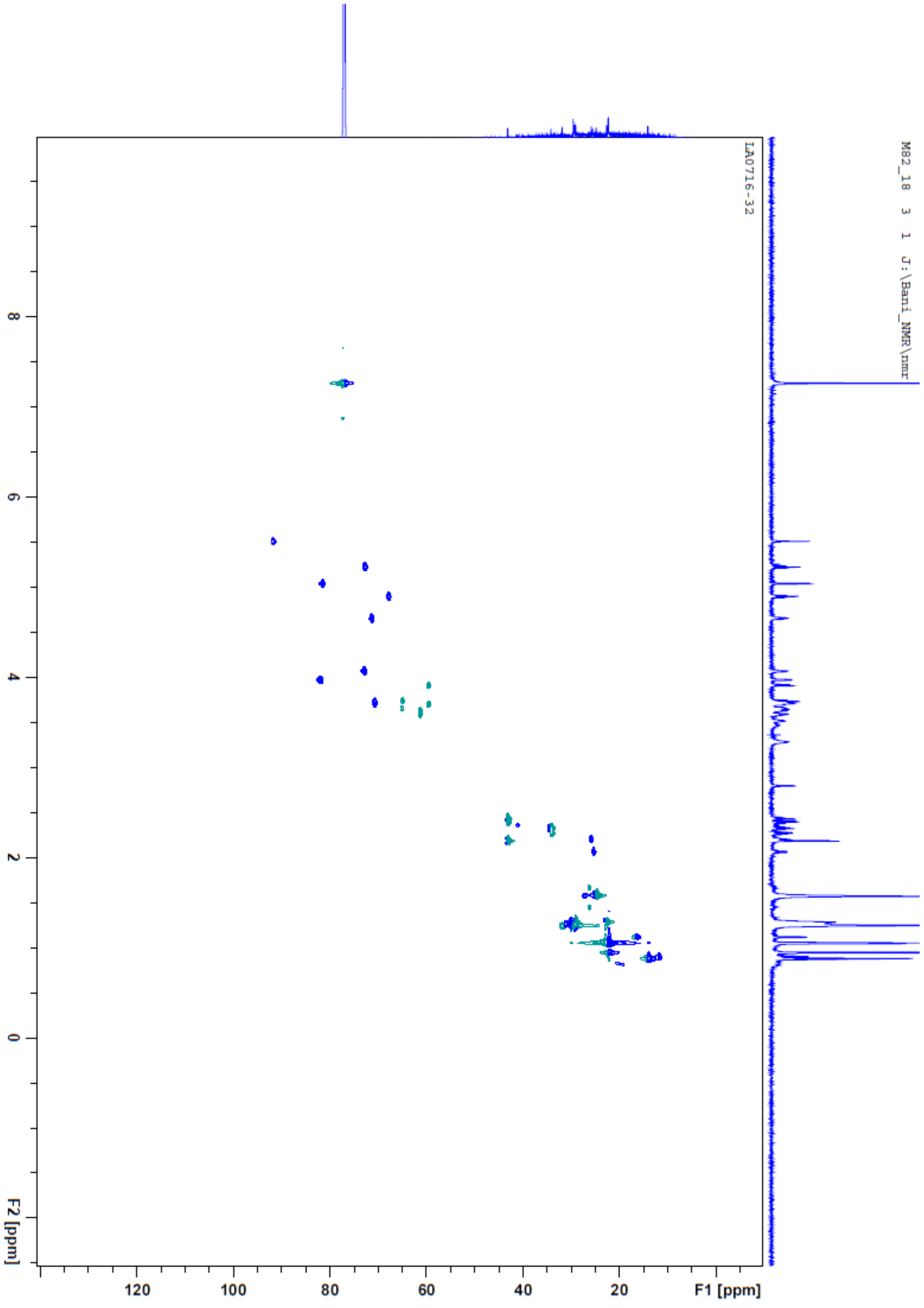
LA0716-32
M82_18 3 1 J:\Bani_NMR\nmr
F2 [ppm]
F1 [ppm]
120
100
80
60
40
20
8
6
4
2
0

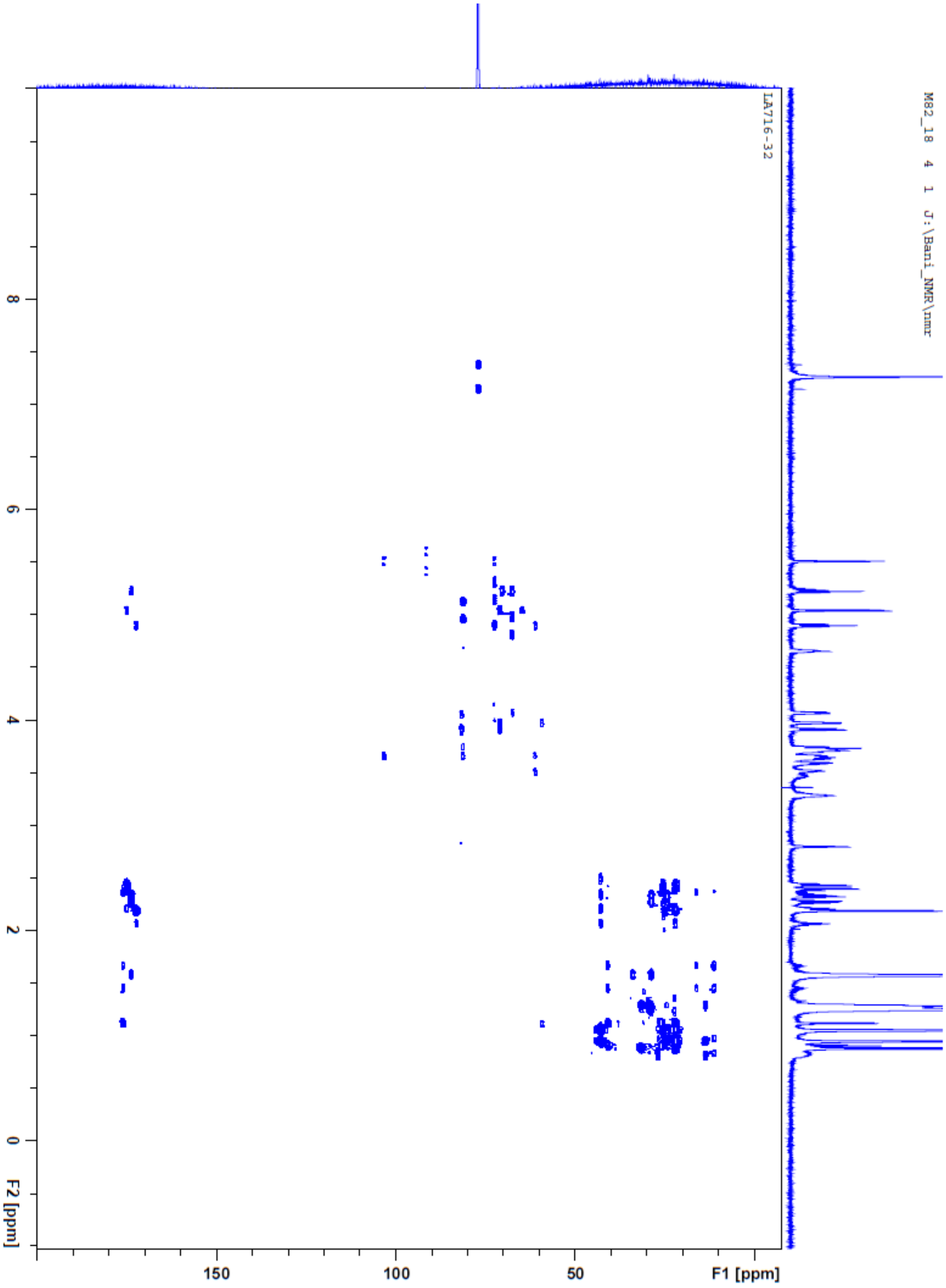

LA716-32
M82_18 4 1 J:\Bani_NMR\nmr
F2 [ppm]
F1 [ppm]

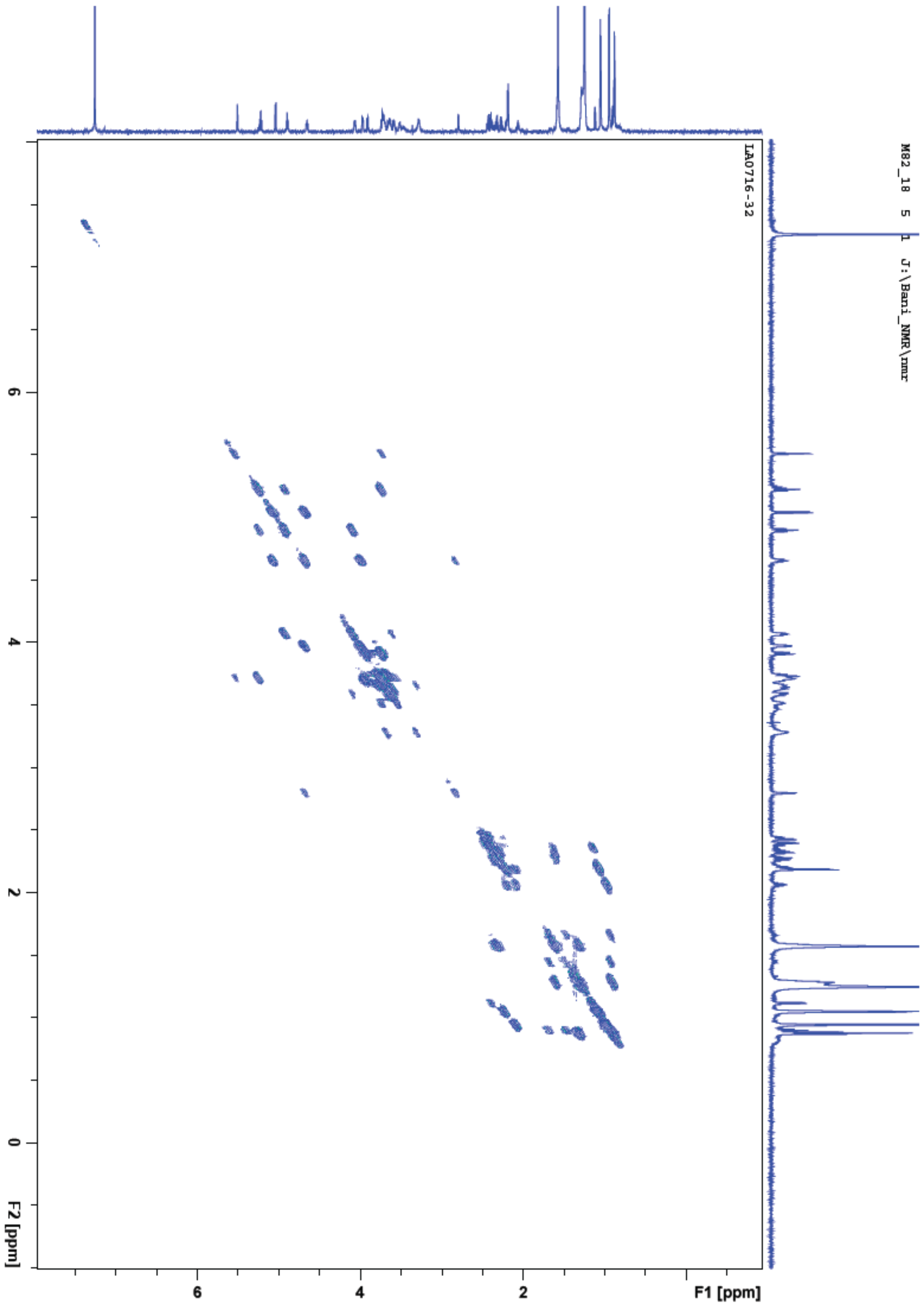
LA0716-32
M82_18 5 1 J:\Bani_NMR\nmr
F1 [ppm]
F2 [ppm]

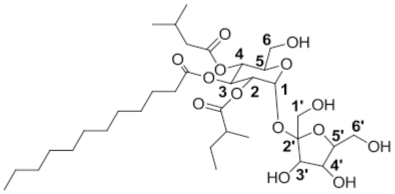

S3:22[5] (5,5,12)

Purified from *S. habrochaites* LA1392

HRMS: (ESI) *m/z* calcd for $C_{35}H_{61}O_{16}$ ([M+HCOO$^-$]): 737.3965 , found: 737.4048

Material recovered: 0.5 - 1 mg

NMR solvent: $CDCl_3$

InChI Key: VWFSSUCTNGJWMR-GIMDXHJVSA-N

Carbon # (group)	^{1}H (ppm)	^{13}C (ppm)
1(CH)	5.76 (*d*, *J* = 3.9 Hz)	88.9 ($^1J_{CH}$ = 177.6 Hz)
2(CH)	4.87 (*dd*, *J* = 10.5, 3.9)	70.7
2-O-		
-1(CO)		176.8
-2(CH)	2.39 (m)	40.5
-2'(CH_3)	1.13 (*d*, *J* = 6.9 Hz)	15.9
-3(CH_2)	1.45 (m), 1.63[a]	26.7
-4(CH_3)	0.87 (m)	11.4
3(CH)	5.55 (t, *J* = 10.1 Hz)	68.9
3-O-		
-1(CO)		173.0
-2(CH_2)	2.21 (m)	34.0
-3(CH_2)	1.53[b]	24.6
-4-9(CH_2-CH_2-CH_2-CH_2-CH_2-CH_2)	1.28 (br. s)	29.1, 29.2, 29.3, 29.4, 29.4, 29.6
-10(CH_2)	1.28 (br. s)	31.9
-11(CH_2)	1.29[b]	22.7
-12(CH_3)	0.88 (t, *J* = 7.1 Hz)	14.1
4(CH)	4.93 (m)	68.4
4-O		

-1(CO)		172.6
-2(CH_2)	2.18 (m)	43.0
-3(CH)	2.05 (m)	25.4
-4(CH_3) x 2	0.94 (d, *J* = 6.8 Hz)	22.3
5(CH)	4.16 (m)	72.0
6(CH_2)	3.60 (m), 3.65 (d, *J* = 12.6 Hz)	61.3
1' (CH_2)	3.52 (dd, *J* = 12.0, 4.1 Hz), 3.60 (m)	64.7
2' (C)		104.5
3' (CH)	4.22 (d, *J* = 8.2 Hz)	78.8
4' (CH)	4.32 (t, *J* = 8.3 Hz)	73.3
5' (CH)	3.77 (d, *J* = 8.2 Hz)	81.7
6' (CH_2)	3.71 (d, *J* = 13.1 Hz), 3.88 (d, *J* = 13.1 Hz)	59.9

[a]Determined by COSY and HSQC

[b]Determined by COSY

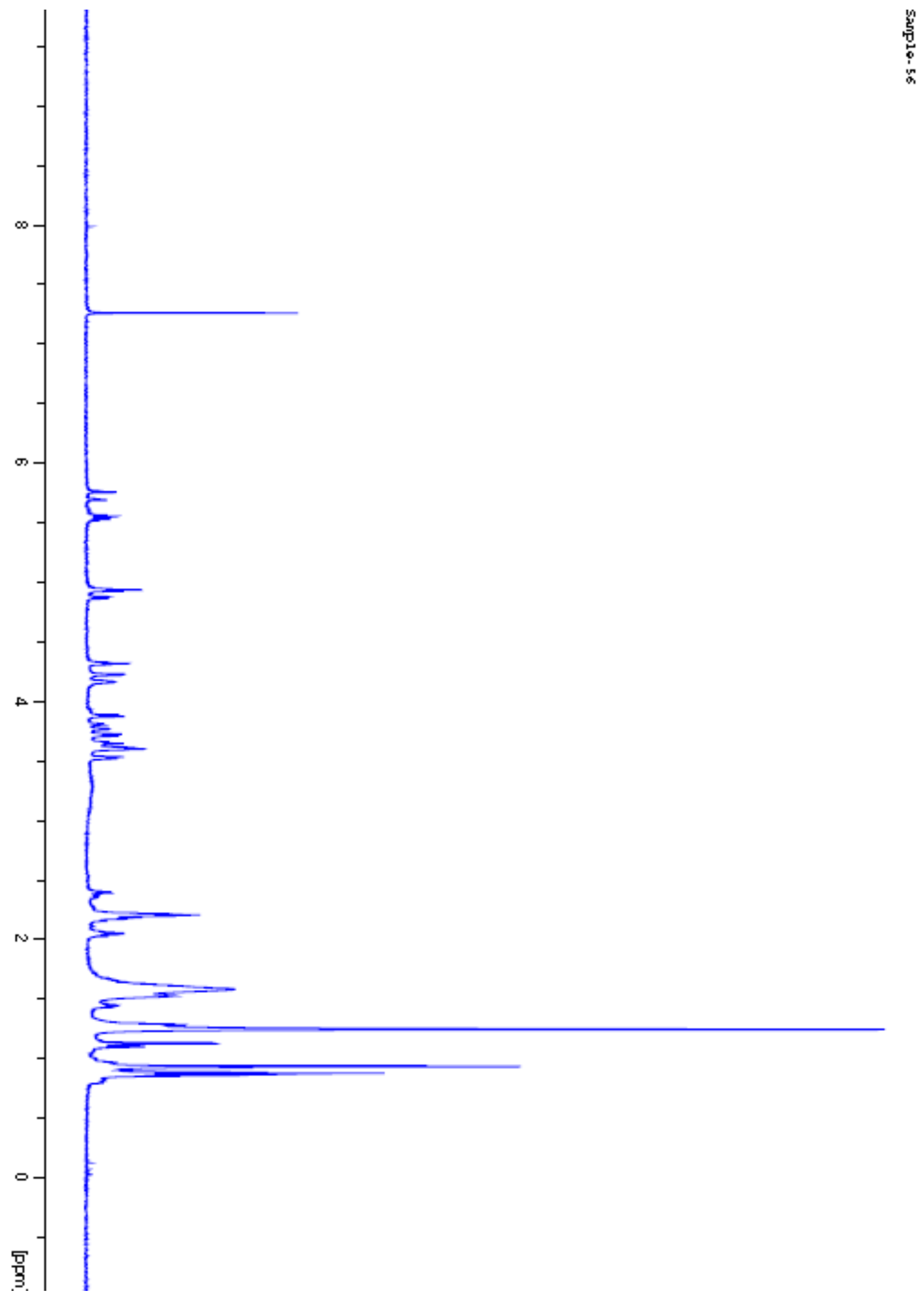
Sample-56
8
6
4
2
0
[ppm]

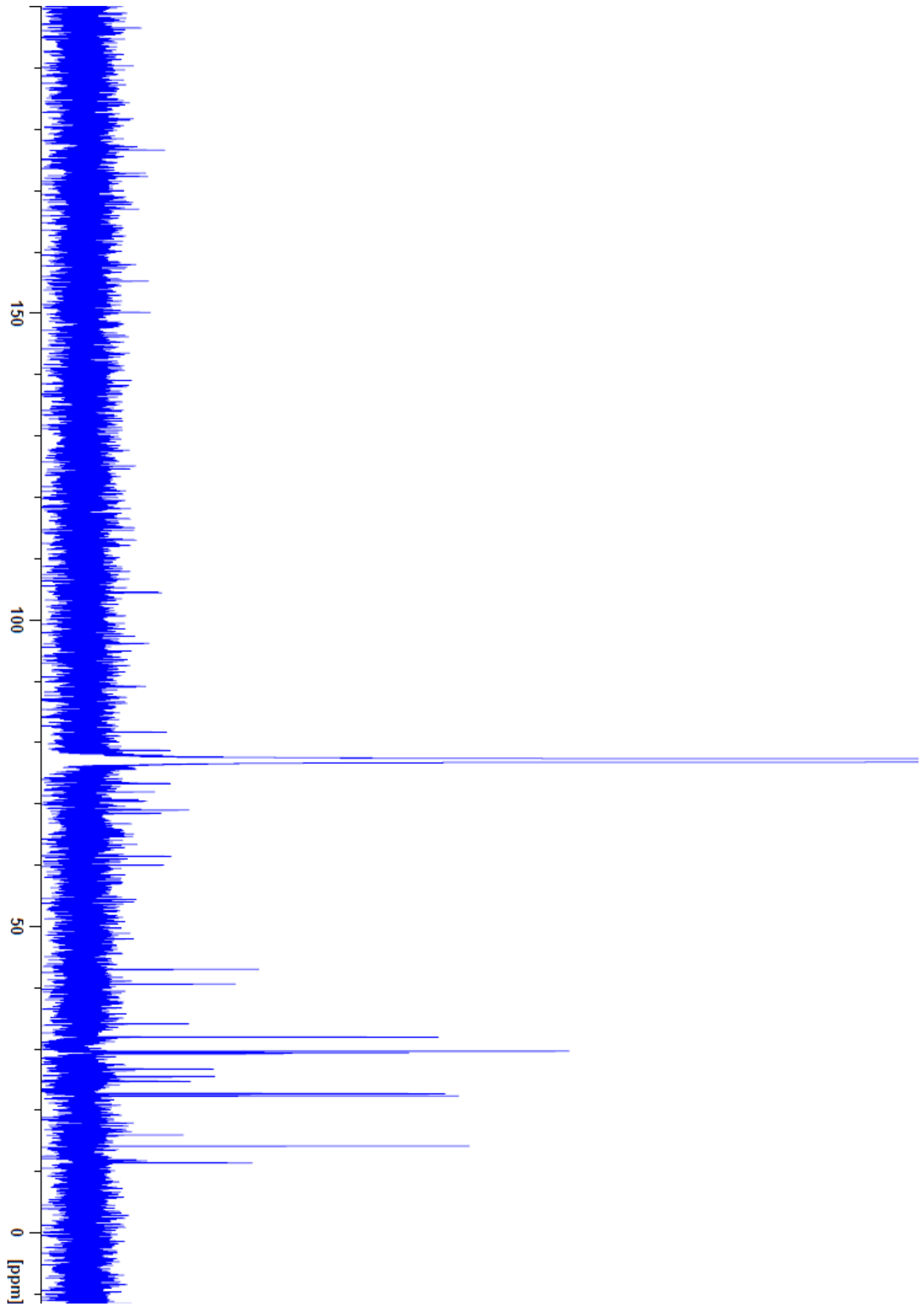
150
100
50
0
[ppm]

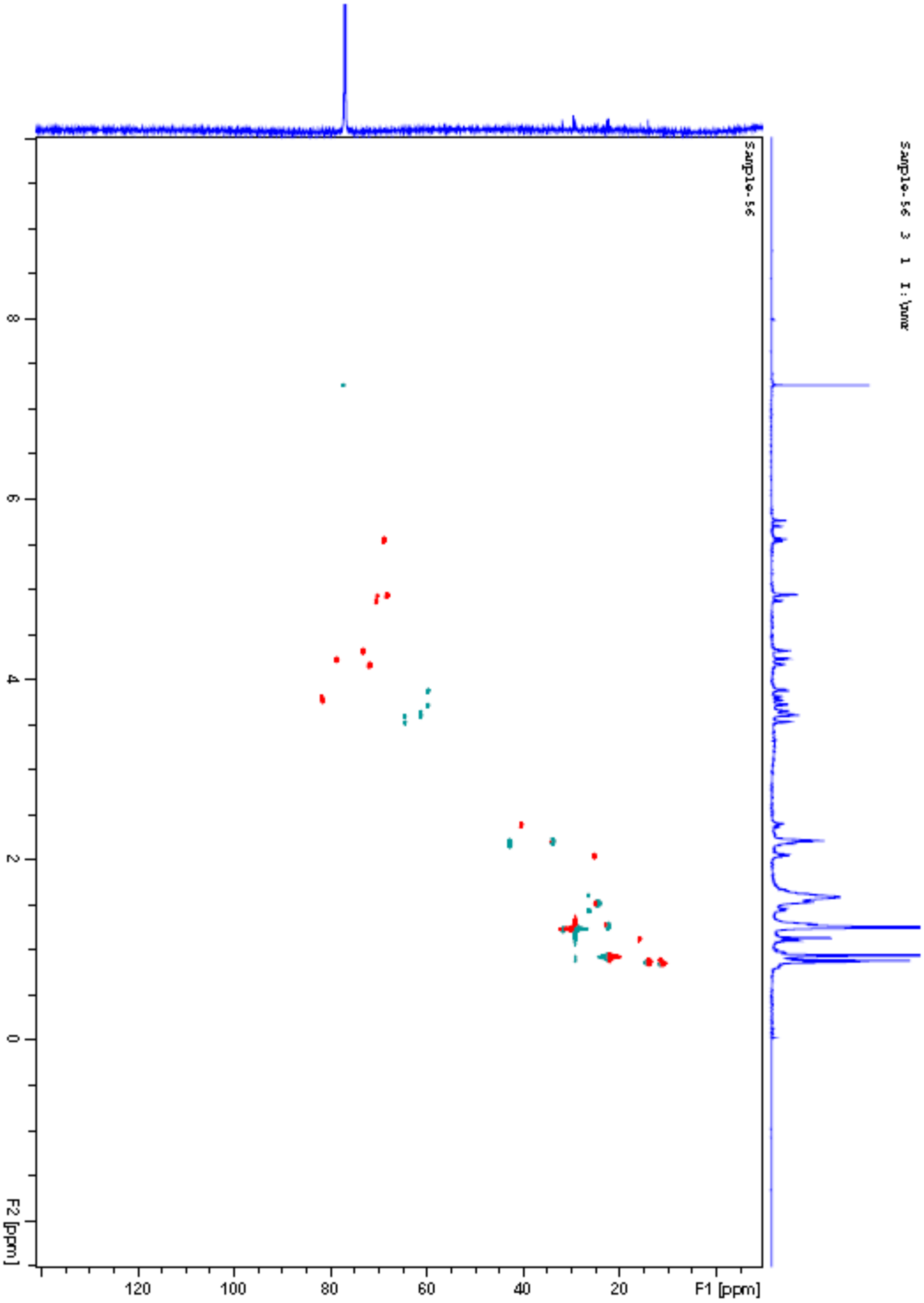

F1 [ppm]
F2 [ppm]

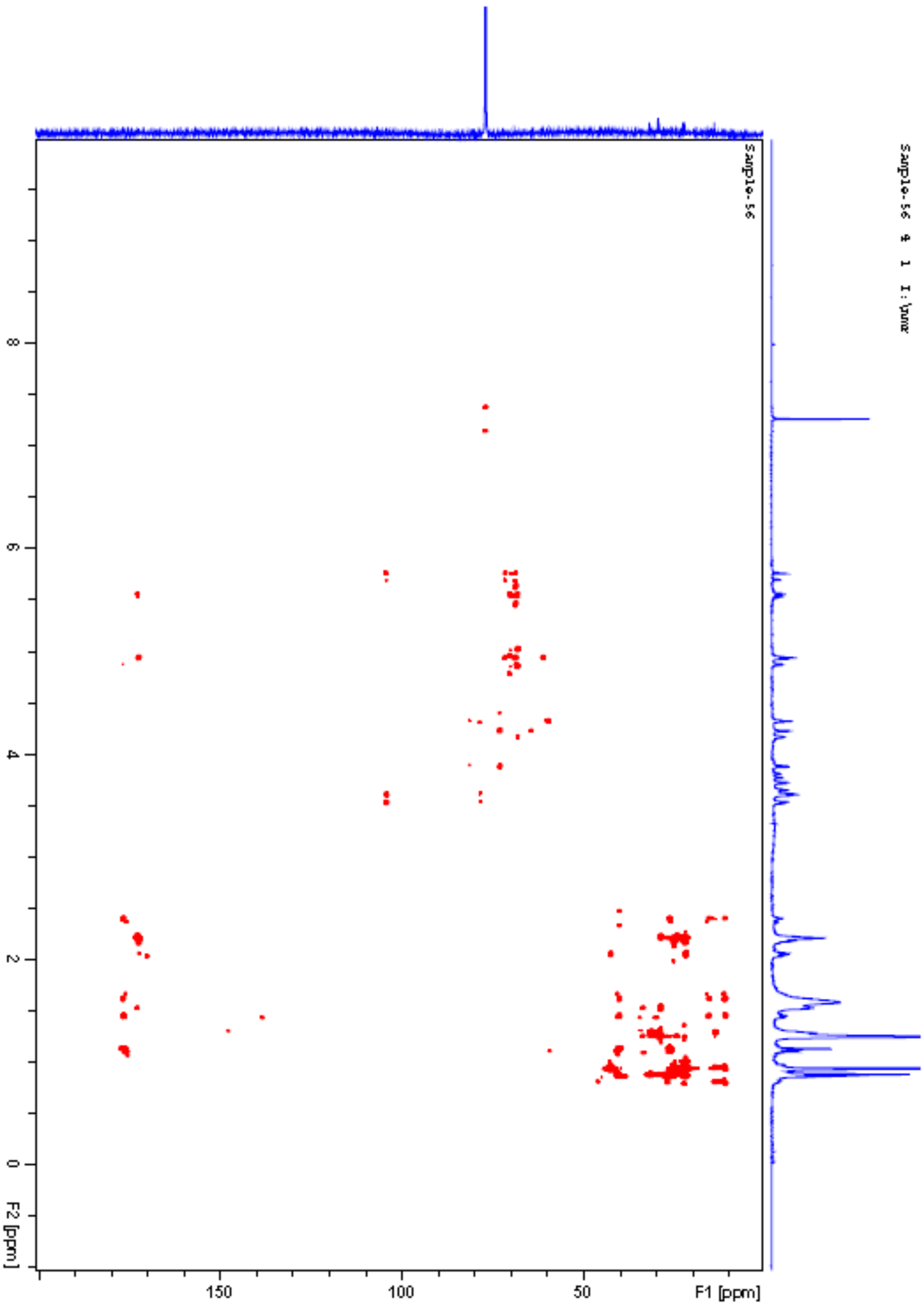
Sample-56
Sample-56 4 1 I:\nmr
F2 [ppm]
F1 [ppm]
150
100
50
8
6
4
2
0

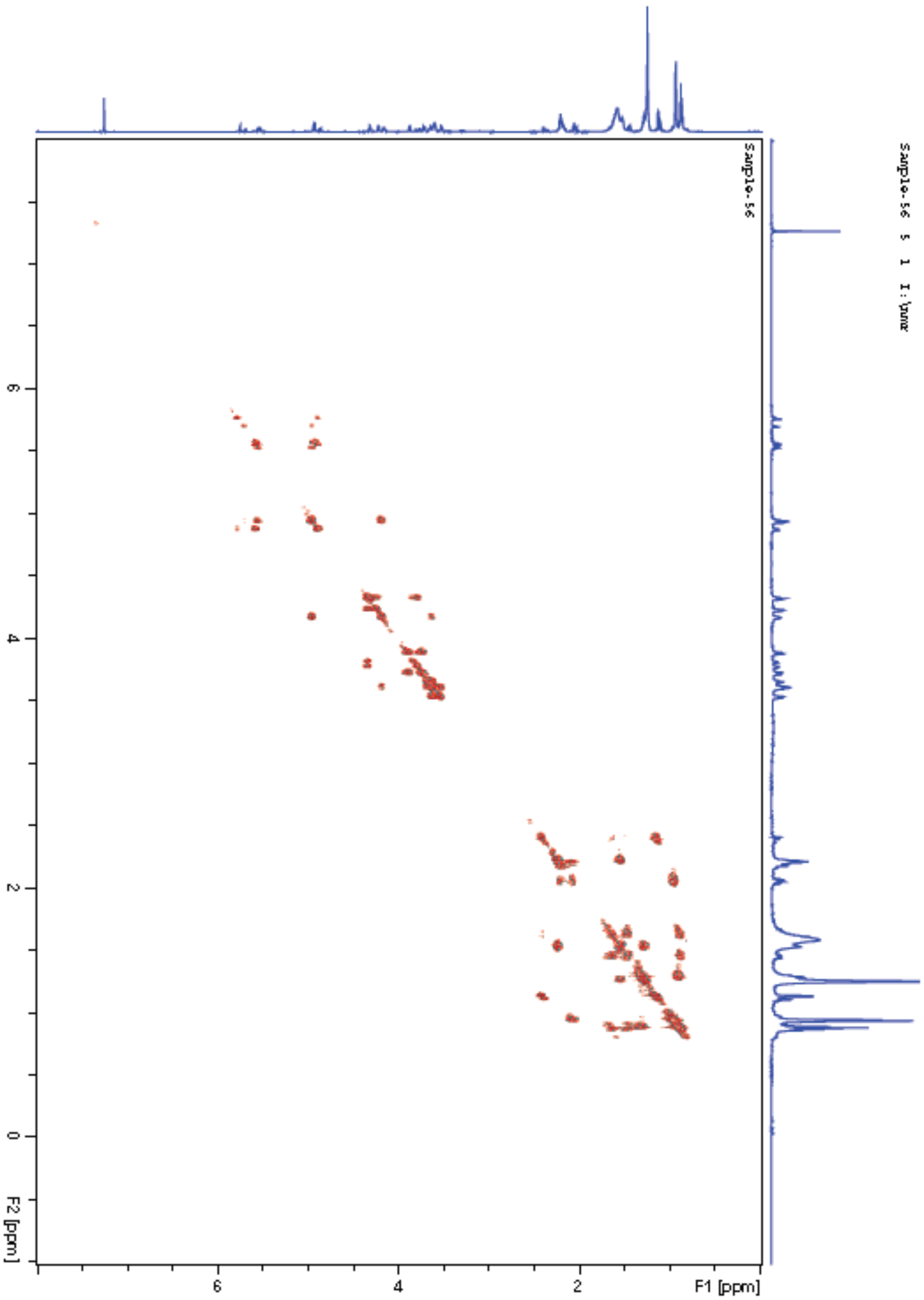
Sample-56
Sample-56 5 1 I:\nmr
F2 [ppm]
F1 [ppm]
6
4
2
0

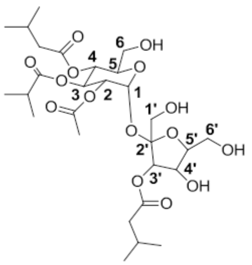

S4:16[3] (2,4,5,5)

Purified from *S. habrochaites* LA1392

HRMS: (ESI) *m/z* calcd for $C_{29}H_{47}O_{17}^{-}$ ([M+HCOO^{-}]): 667.2819, found: 667.2884

Material recovered: 2-3 mg

NMR solvent: $CDCl_3$

InChI Key: AKWKWIHXNGCVIN-GWPJAQCUSA-N

Carbon # (group)	^{1}H (ppm)	^{13}C (ppm)
1(CH)	5.60 (d, J = 3.8 Hz)	89.4 ($^1J_{CH}$ = 178.2 Hz)
2(CH)	4.93 (dd, J = 10.4, 3.8 Hz)	70.3
2-O-		
-1(CO)		170.0
-2(CH_3)	2.03 (s)	20.6
3(CH)	5.46 (t, J = 10.0 Hz)	69.0
3-O-		
-1(CO)		175.8
-2(CH)	2.48 (m)	33.9
-3(CH_3) x 2	1.08 (d, J = 4.7 Hz), 1.09 (d, J = 4.7 Hz)	18.6
4(CH)	4.95 (t, J = 10.0 Hz)	68.2
4-O		
-1(CO)		172.2
-2(CH_2)	2.18 (dd, J = 15.0, 7.1 Hz), 2.20 (dd, J = 15.0, 7.1 Hz) [a]	43.0
-3(CH)	2.05 (m)	25.4
-4(CH_3) x 2	0.93 (d, J = 6.6 Hz)	22.3
5(CH)	4.13 (m)	72.1
6(CH_2)	3.61 (m)	61.6

1' (CH_2)	3.50 (d, *J* = 12.4 Hz), 3.61 (m)	64.5
2' (C)		103.9
3' (CH)	5.20 (d, *J* = 8.1 Hz)	79.5
3'-O		
-1(CO)		174.3
-2(CH_2)	2.41 (dd, *J* = 15.0, 7.1 Hz), 2.45 (dd, *J* = 15.0, 7.1 Hz)[a]	43.1
-3(CH)	2.20 (m)	25.8
-4(CH_2) x 2	1.04 (d, *J* = 2.3 Hz), 1.05 (d, *J* = 2.3 Hz)	22.4
4' (CH)	4.58 (d, *J* = 8.2 Hz)	71.2
5' (CH)	3.94 (ddd, J = 8.5, 2.3, 2.3 Hz)	82.5
6' (CH_2)	3.72 (d, *J* = 13.2, 2.1 Hz), 3.91 (dd, *J* = 13.2, 2.5 Hz)	59.7

[a]Higher order multiplet derived from the constants using gNMR

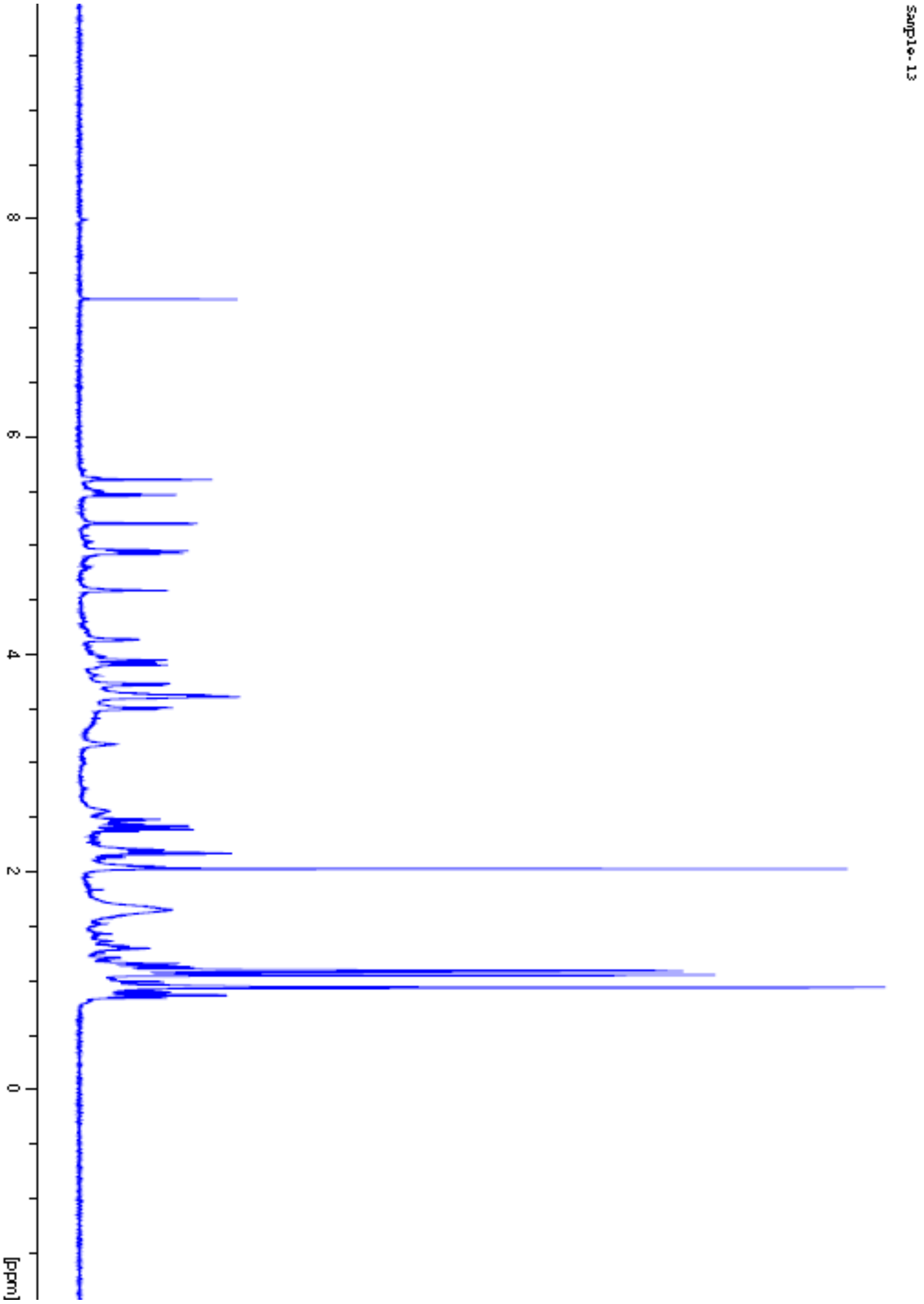
Sample-13
8
6
4
2
0
[ppm]

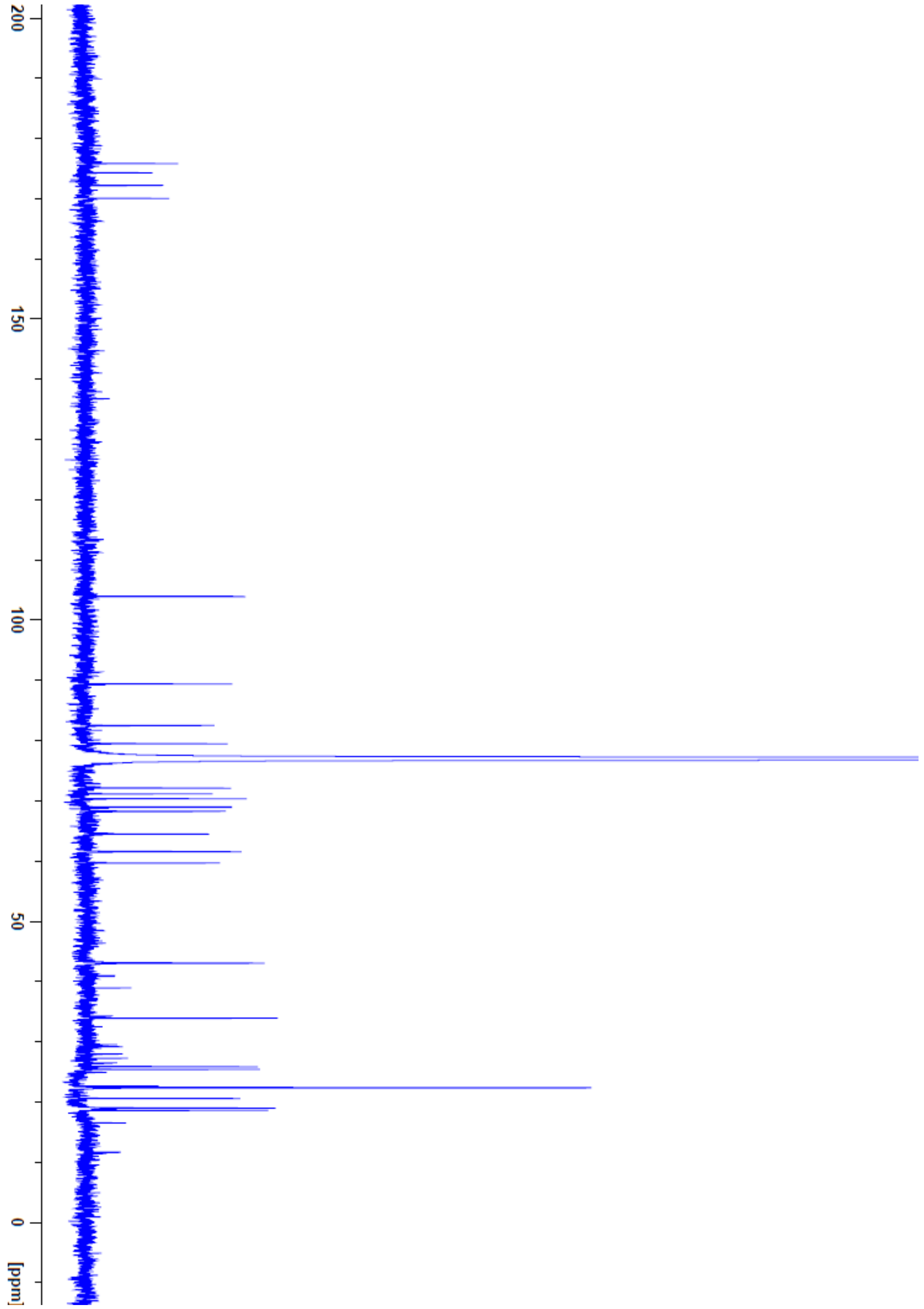
200
150
100
50
0
[ppm]

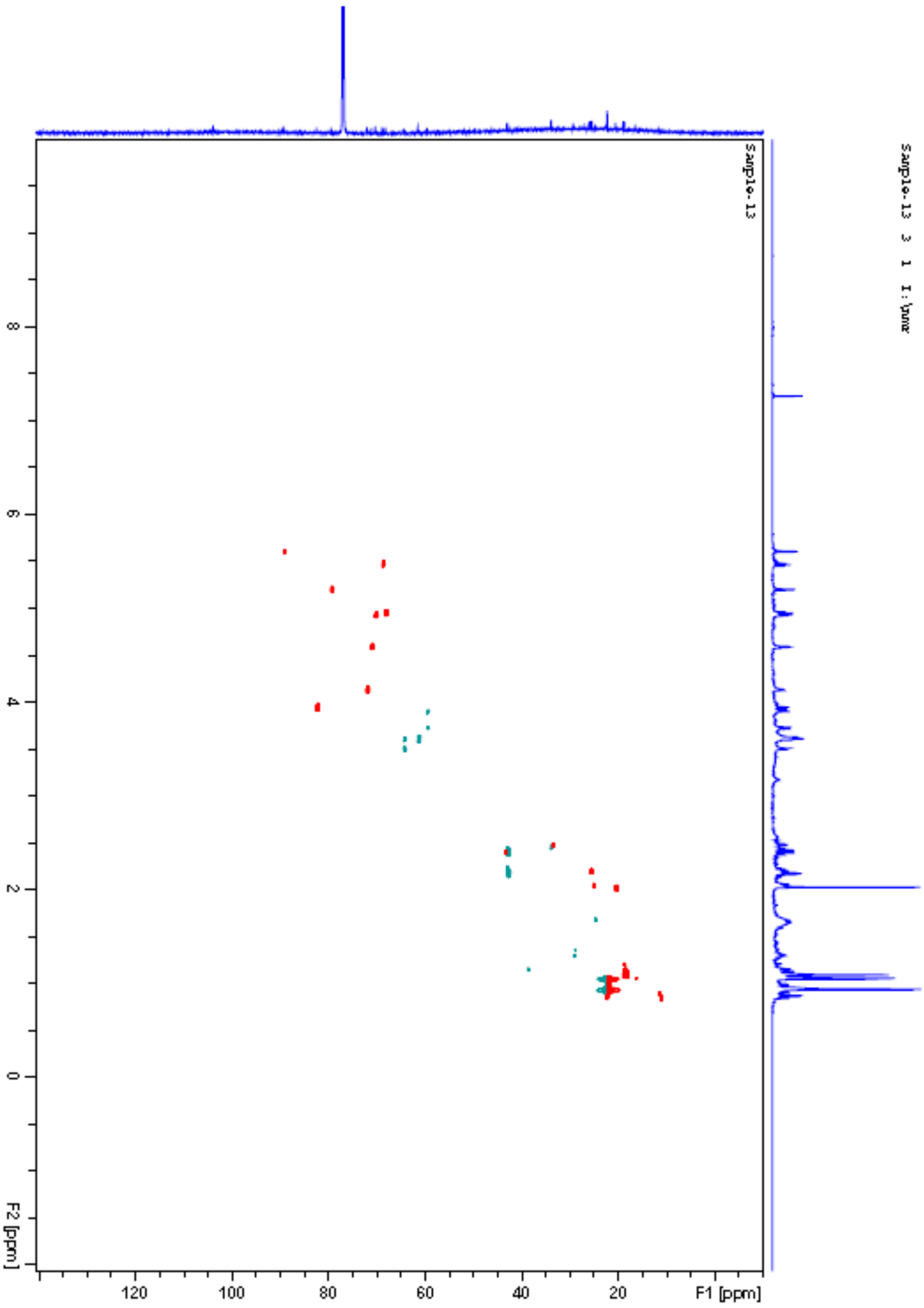
Sample-13
Sample-13 3 1 I:\nmr
F2 [ppm]
F1 [ppm]
120
100
80
60
40
20
8
6
4
2
0

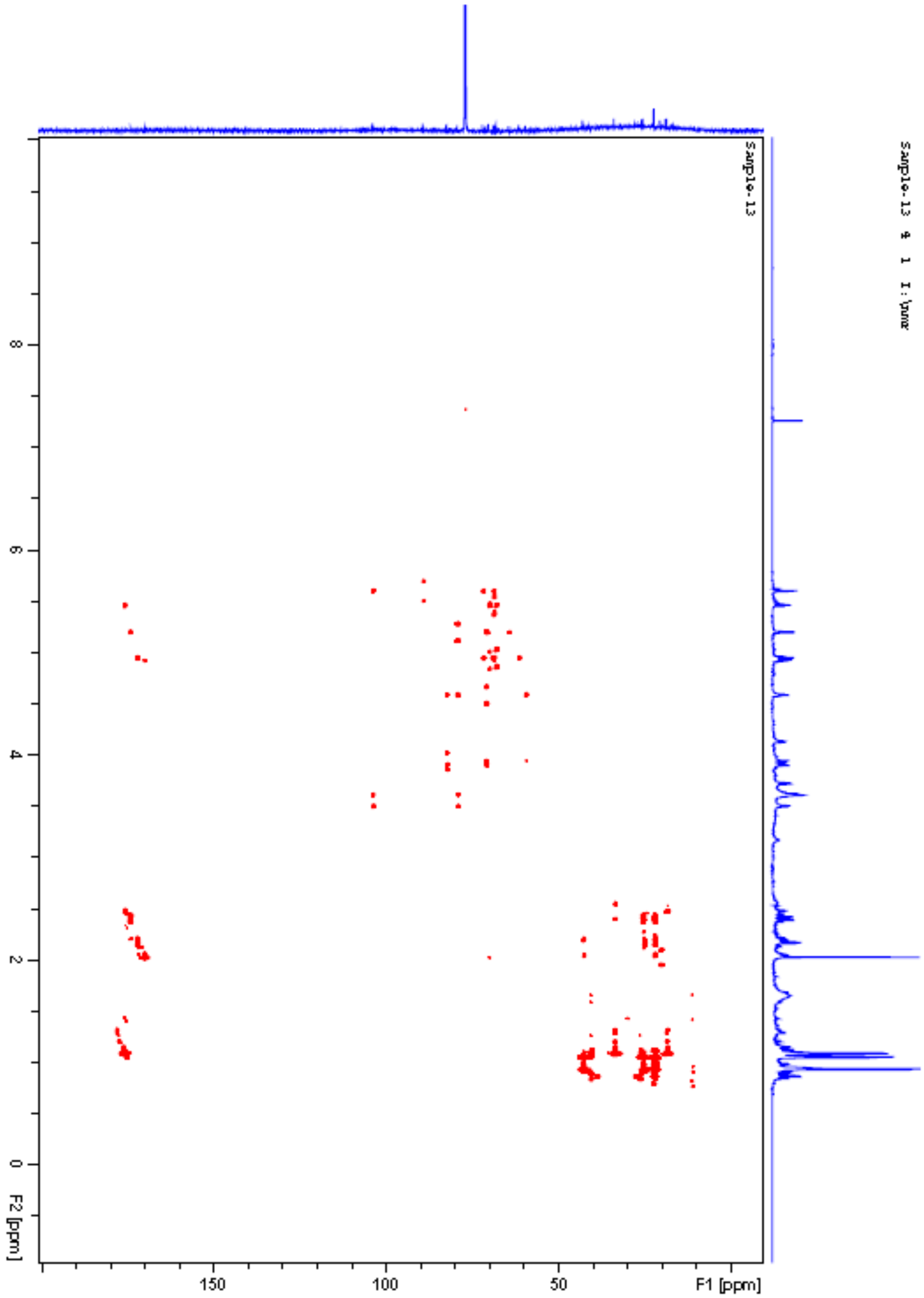
Sample-13
F1 [ppm]
F2 [ppm]

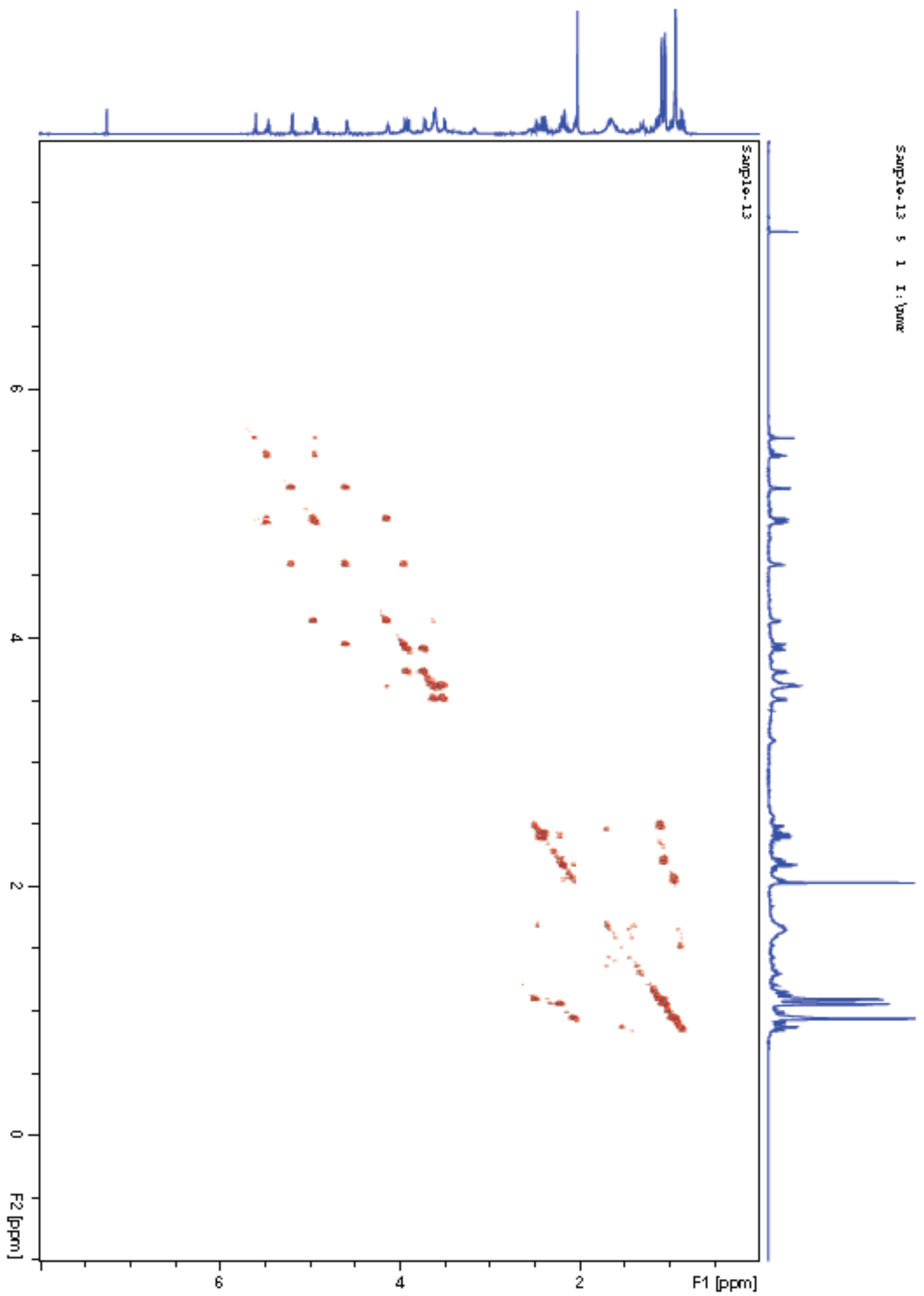
Sample-13
Sample-13 5 1 I:\nmr
F2 [ppm]
F1 [ppm]
6
4
2
0

**S4:17[2]** (2,5,5,5)<br><br>**Purified from *S. habrochaites* LA1392**<br><br>**HRMS:** (ESI) *m/z* calcd for $C_{30}H_{49}O_{17}^{-}$ ([M+HCOO^{-}]): 681.2975, found: 681.3039<br><br>**Material recovered:** 2-3 mg<br><br>**NMR solvent**: $CDCl_3$<br><br>**InChI Key**: JTIAMKZBSXQQDB-QAFHIVESSA-N		
Carbon # (group)	^{1}H (ppm)	^{13}C (ppm)
1(CH)	5.61 (d, J = 3.8 Hz)	89.4 ($^{1}J_{CH}$ = 177.5 Hz)
2(CH)	4.91 (dd, J = 10.4, 3.8 Hz)	70.4
2-O-		
-1(CO)		170.0
-2(CH_3)	2.02 (s)	20.6
3(CH)	5.49 (t, J = 10.0 Hz)	68.8
3-O-		
-1(CO)		175.4
-2(CH)	2.32 (m)	40.9
-2'(CH_3)	1.06 (d, J = 7.0 Hz)	16.4
-3(CH_2)	1.41 (m), 1.59[a]	26.5
-4(CH_3)	0.85 (t, J = 7.4 Hz)	11.5
4(CH)	4.94 (t, J = 10.0 Hz)	68.4
4-O		
-1(CO)		172.2
-2(CH_2)	2.18 (dd, J = 14.9, 7.2 Hz), 2.21 (dd, J = 14.9, 7.2 Hz) [b]	42.9
-3(CH)	2.05 (m)	25.4
-4(CH_3) x 2	0.93 (d, J = 1.7 Hz), 0.94 (d, J = 1.7 Hz)	22.3

5(CH)	4.13 (m)	72.1
6(CH_2)	3.61 (m)	61.6
1' (CH_2)	3.50 (d, J = 12.0 Hz), 3.62[c]	64.5
2' (C)		103.9
3' (CH)	5.18 (d, J = 8.1 Hz)	79.6
3'-O		
-1(CO)		174.3
-2(CH_2)	2.41 (dd, J = 15.0, 7.1 Hz), 2.46 (dd, J = 15.0, 7.1 Hz) [b]	43.1
-3(CH)	2.20 (m)	25.8
-4(CH_2) x 2	1.05 (d, J = 2.0 Hz), 1.05 (d, J = 2.1 Hz)	22.4
4' (CH)	4.60 (t, J = 8.2 Hz)	71.2
5' (CH)	3.93 (ddd, J = 8.5, 2.3, 2.3 Hz)	82.4
6' (CH_2)	3.72 (dd, J = 12.9, 2.0 Hz), 3.91 (dd, J = 13.0, 2.3 Hz)	59.6

[a]Determined by COSY

[b]Higher order multiplet derived from the constants using gNMR

[c]Determined by COSY and HSQC

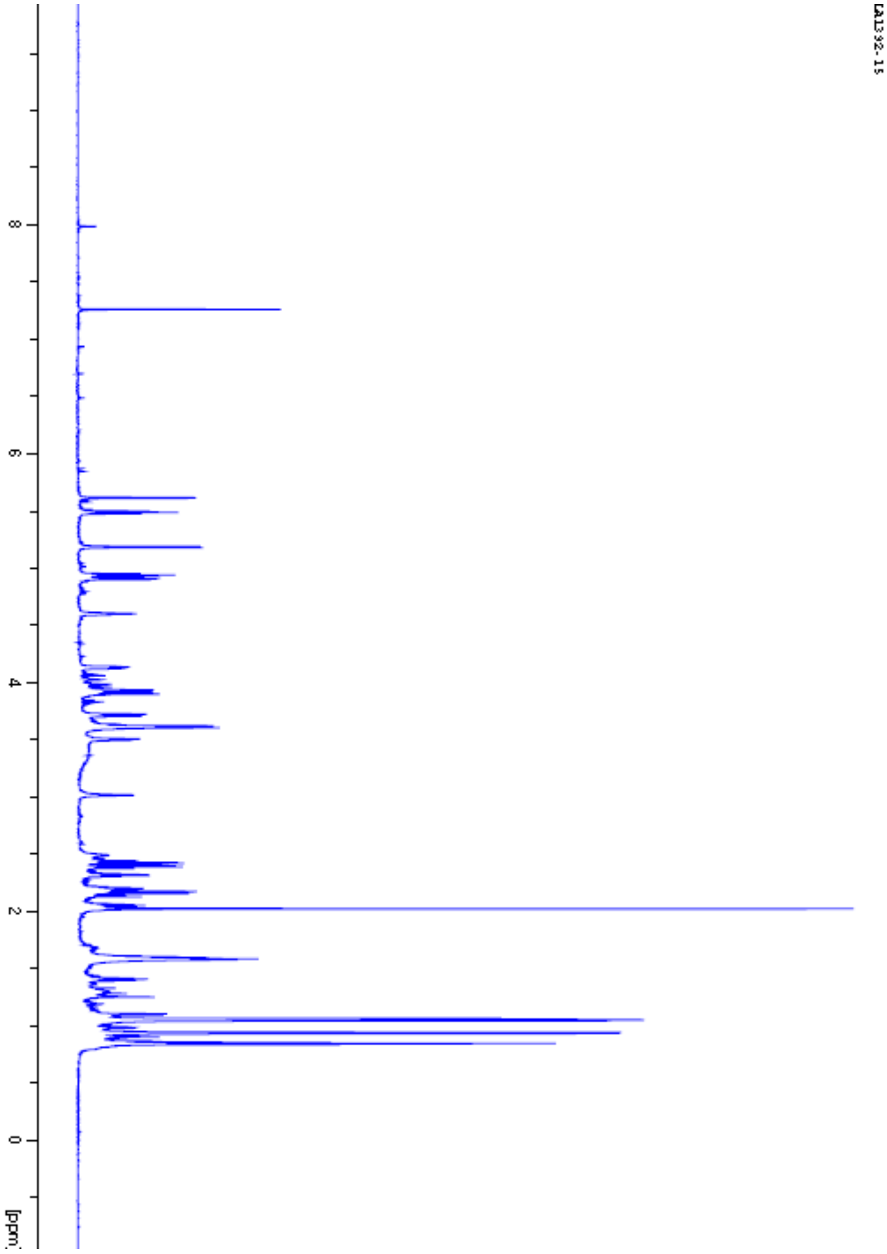
LA1392-15
8
6
4
2
0
[ppm]

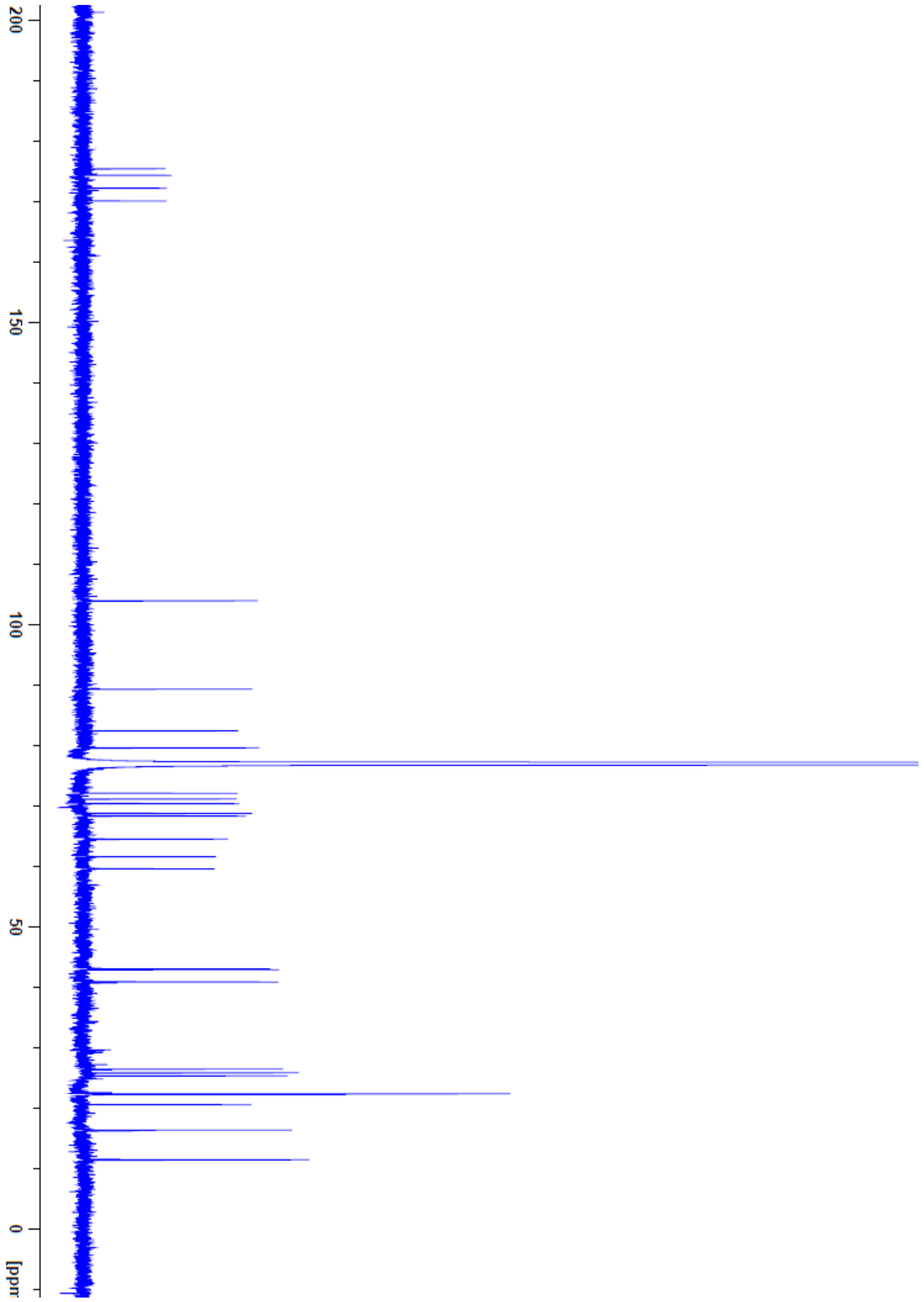
200
150
100
50
0
[ppm

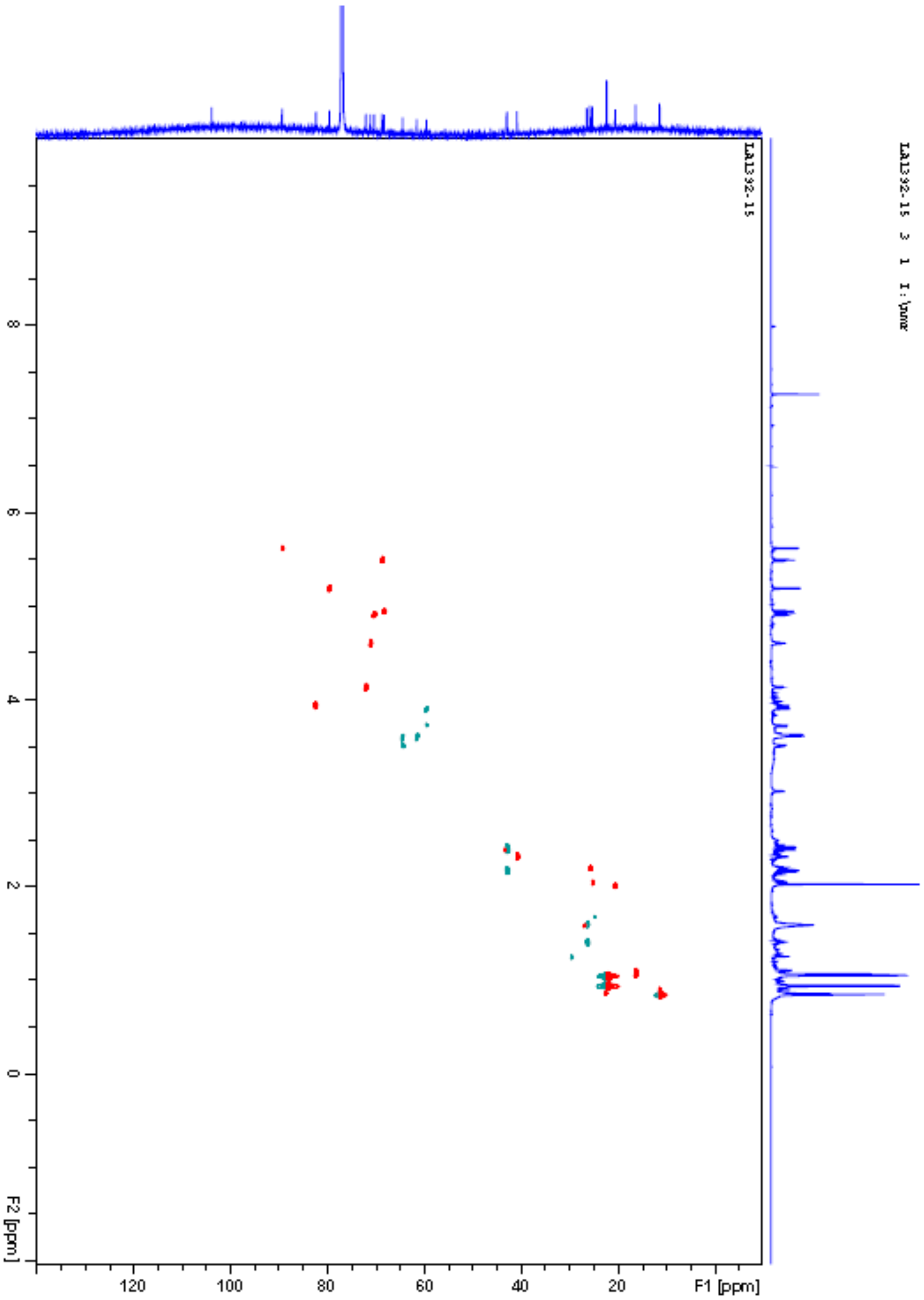

LA1392-15
LA1392-15 3 1 I:\nmr
F1 [ppm]
F2 [ppm]

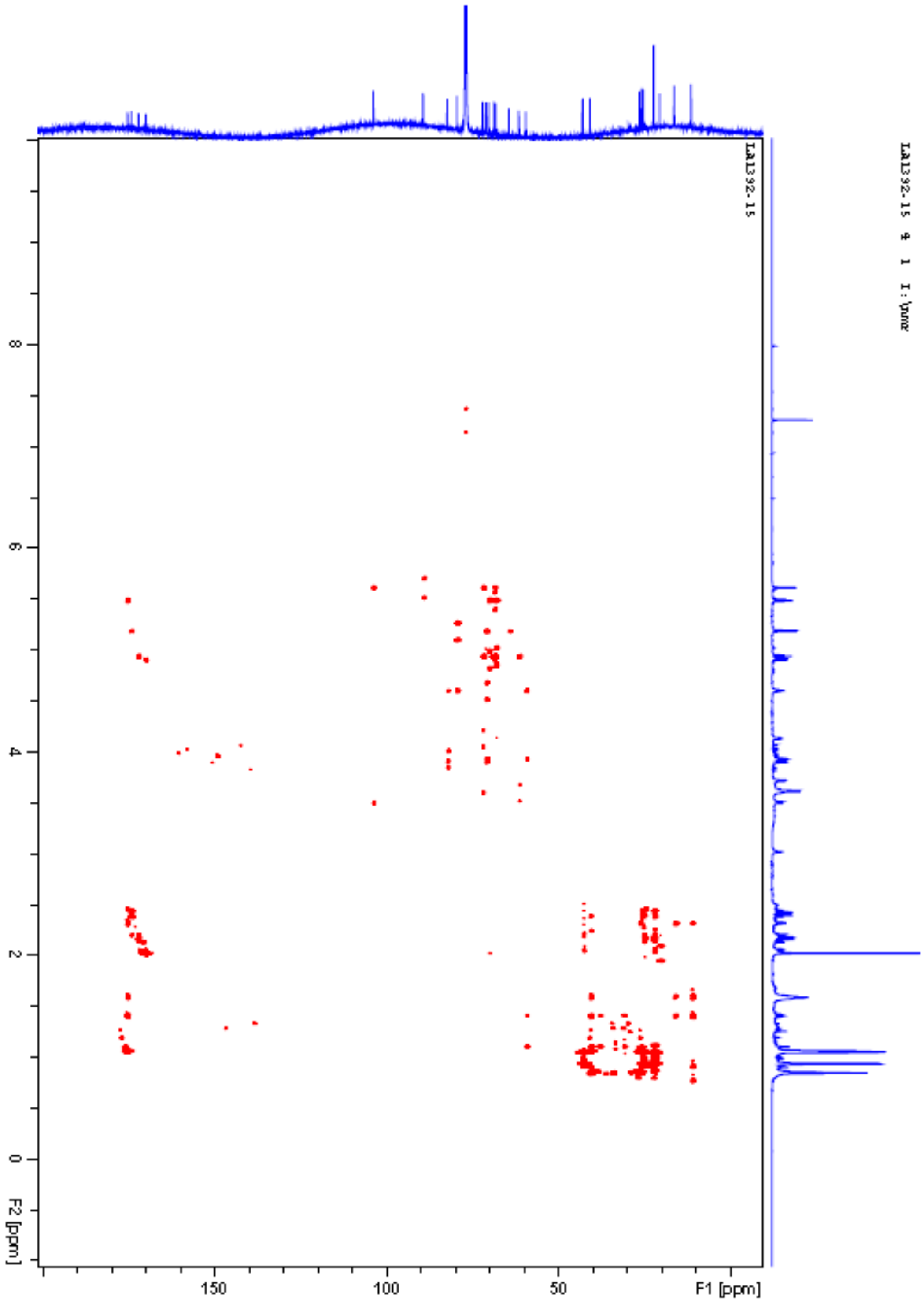
LA1392-15
F2 [ppm]
F1 [ppm]

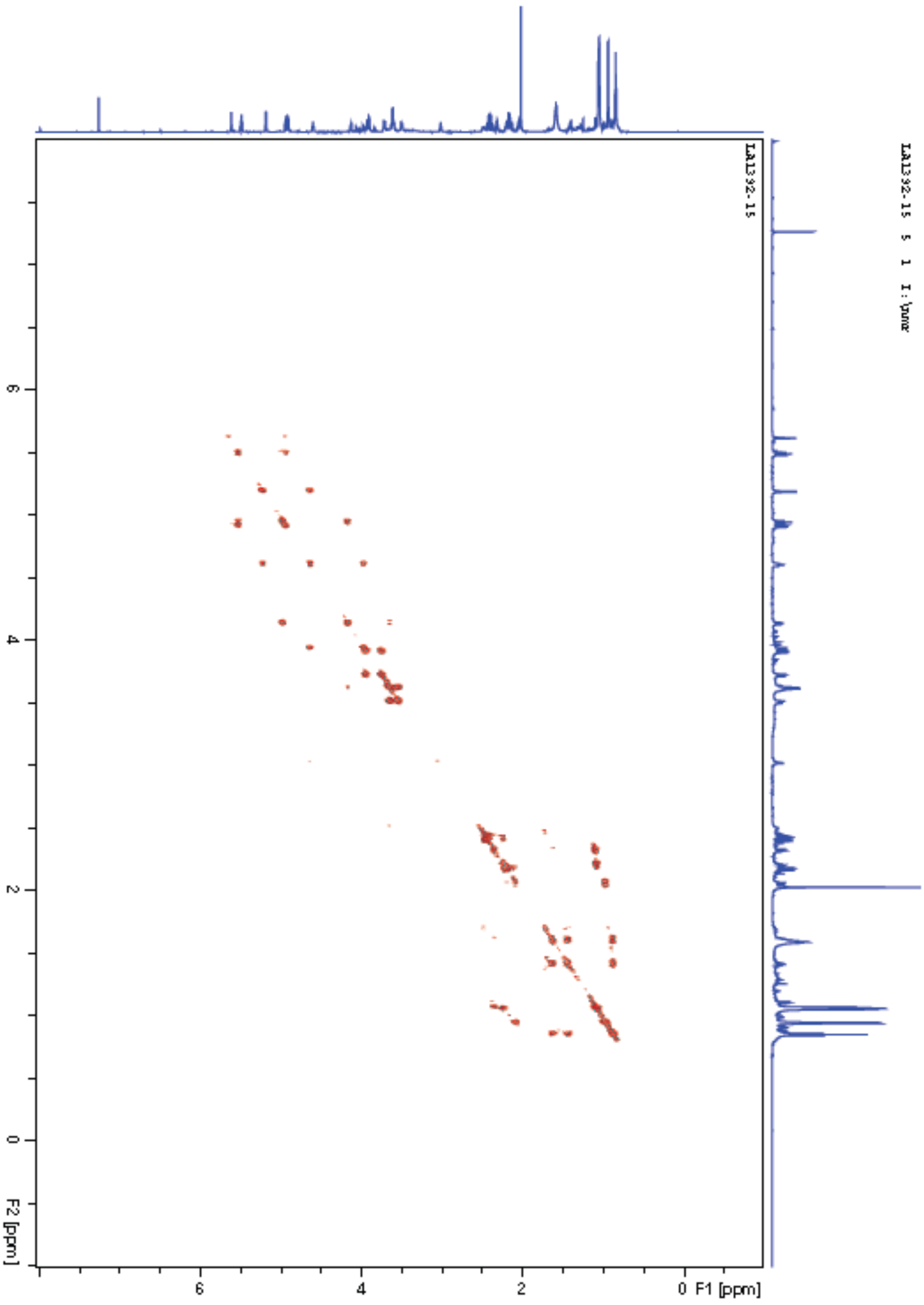

LA1392-15
F2 [ppm]
F1 [ppm]

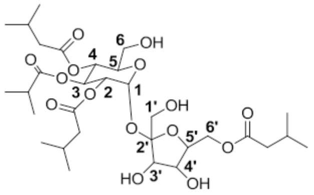

S4:19[7] (4,5,5,5)

Purified from *S. habrochaites* LA1392

HRMS: (ESI) *m/z* calcd for $C_{32}H_{53}O_{17}^{-}$ ([M+HCOO^{-}]): 709.3288, found: 709.3358

Material recovered: ~ 1 mg

NMR solvent: $CDCl_3$;

InChI Key: JTIAMKZBSXQQDB-QAFHIVESSA-N

Carbon # (group)	^{1}H (ppm)	^{13}C (ppm)
1(CH)	5.57 (d, J = 3.7 Hz)	89.3 ($^{1}J_{CH}$ = 176.9 Hz)
2(CH)	5.00 (dd, J = 10.5, 3.8 Hz)	70.1
2-O-		
-1(CO)		171.8
-2(CH_2)	2.17 (m)	42.9
-3(CH)	2.05 (m)	25.4
-4(CH_3) x 2	0.93 (d, J = 3.3 Hz), 0.94 (d, J = 3.1 Hz)	22.3
3(CH)	5.52 (t, J = 9.9 Hz)	69.2
3-O-		
-1(CO)		176.3
-2(CH)	2.47 (m)	33.9
-3(CH_3) x 2	1.09 (d, J = 7.0 Hz)	18.8
4(CH)	4.98 (t, J = 10.0 Hz)	68.6
4-O		
-1(CO)		172.2
-2(CH_2)	2.17 (m)	42.7
-3(CH)	2.05 (m)	25.3
-4(CH_3) x 2	0.92 (d, J = 6.7 Hz), 0.93 (d, J = 6.7 Hz)	22.3

5(CH)	4.11 (m)	72.2
6(CH_2)	3.61 (m), 3.67 (d, *J* = 12.4 Hz)	61.6
1' (CH_2)	3.58 (d, *J* = 12.0 Hz), 3.61 (m)	64.3
2' (C)		104.4
3' (CH)	4.21 (m)	78.7
4' (CH)	4.11 (m)	75.9
5' (CH)	3.99 (m)	79.4
6' (CH_2)	4.21 (m), 4.48 (dd, *J* = 12.0, 6.6 Hz)	64.2
3'-O		
-1(CO)		173.7
-2(CH_2)	2.27 (m)	43.0
-3(CH)	2.12 (m)	25.6
-4(CH_2) x 2	0.96 (d, *J* = 3.5 Hz), 0.97 (d, *J* = 3.5 Hz)	22.4

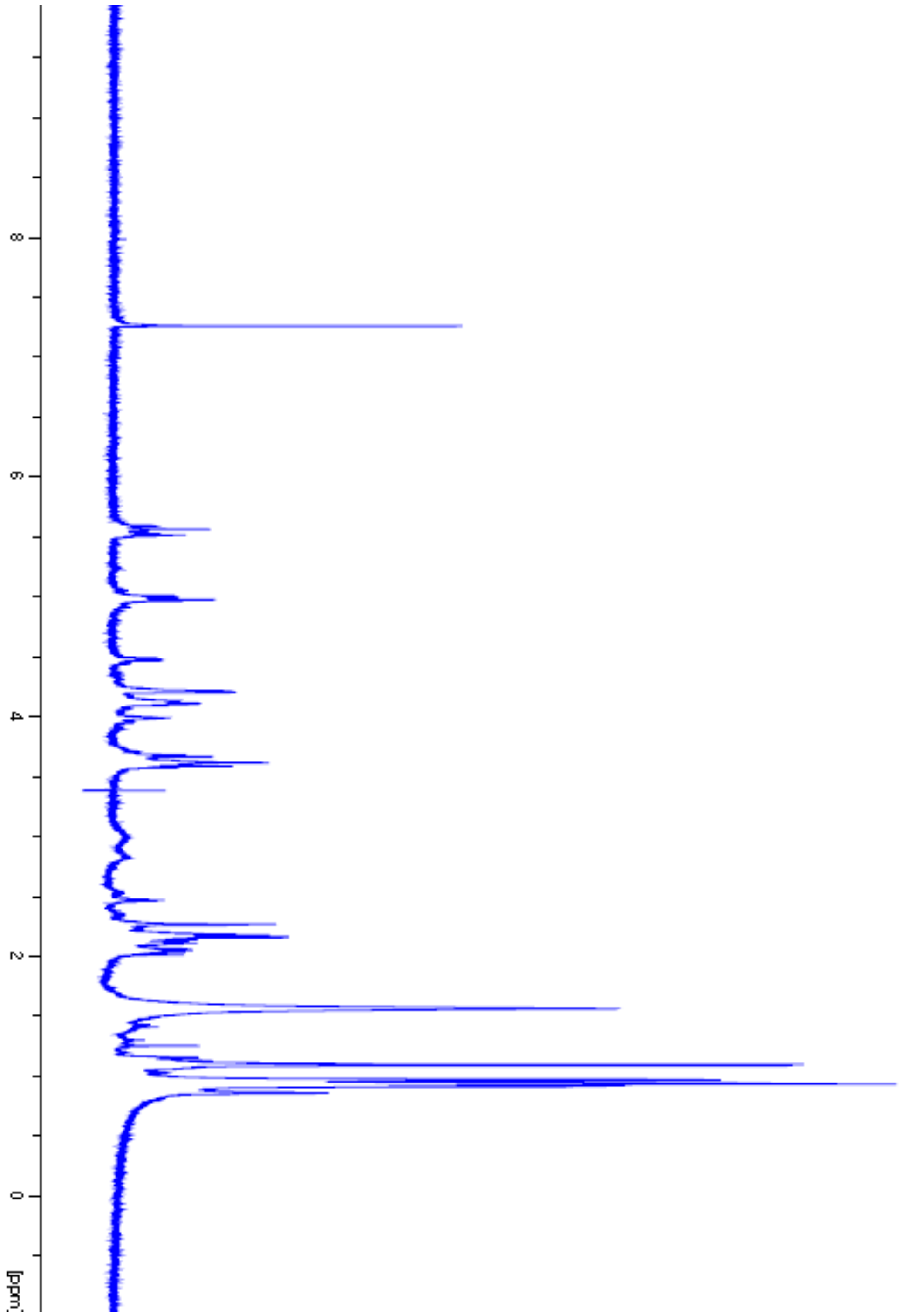
8
6
4
2
0
[ppm]

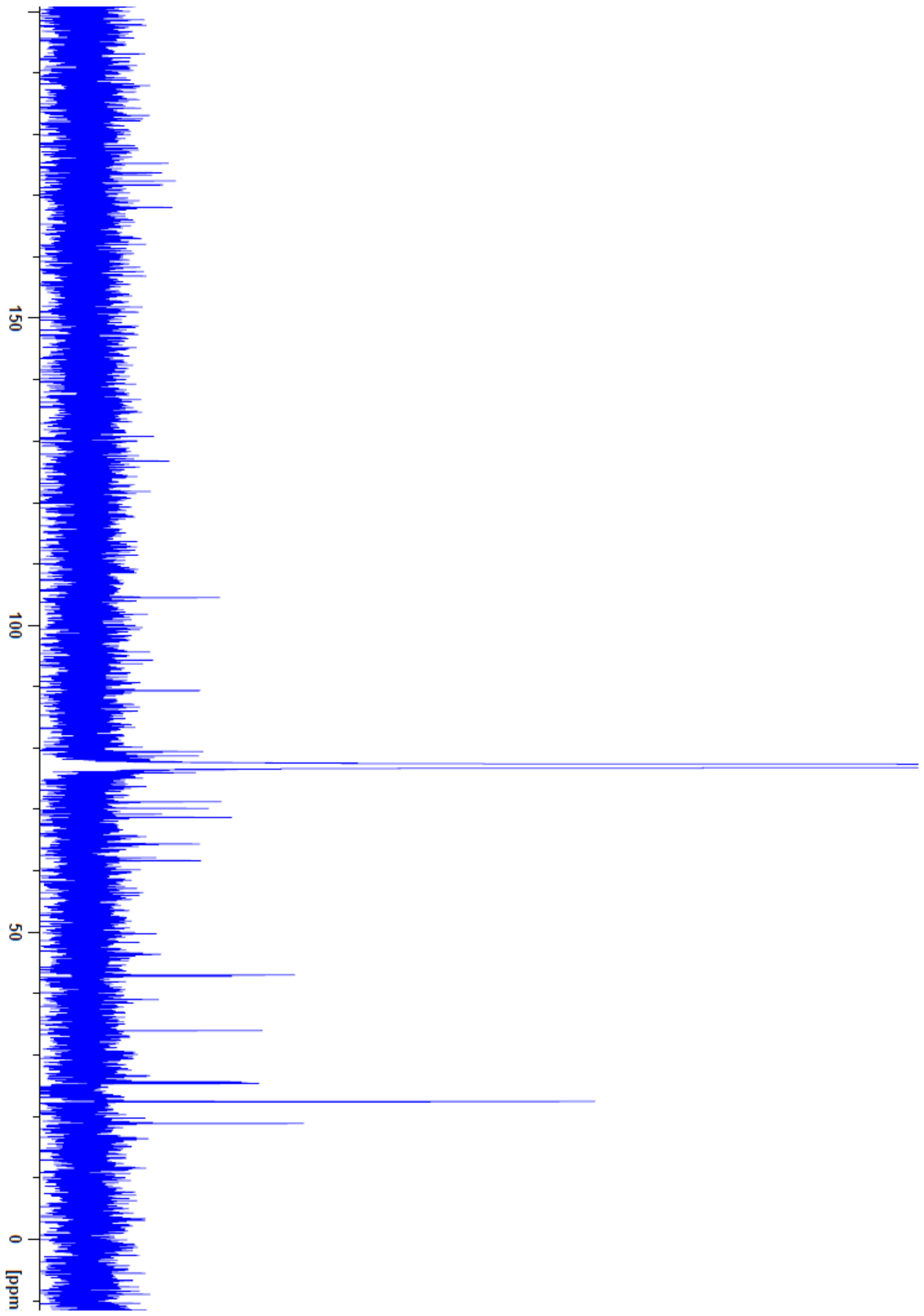

150
100
50
0
[ppm]

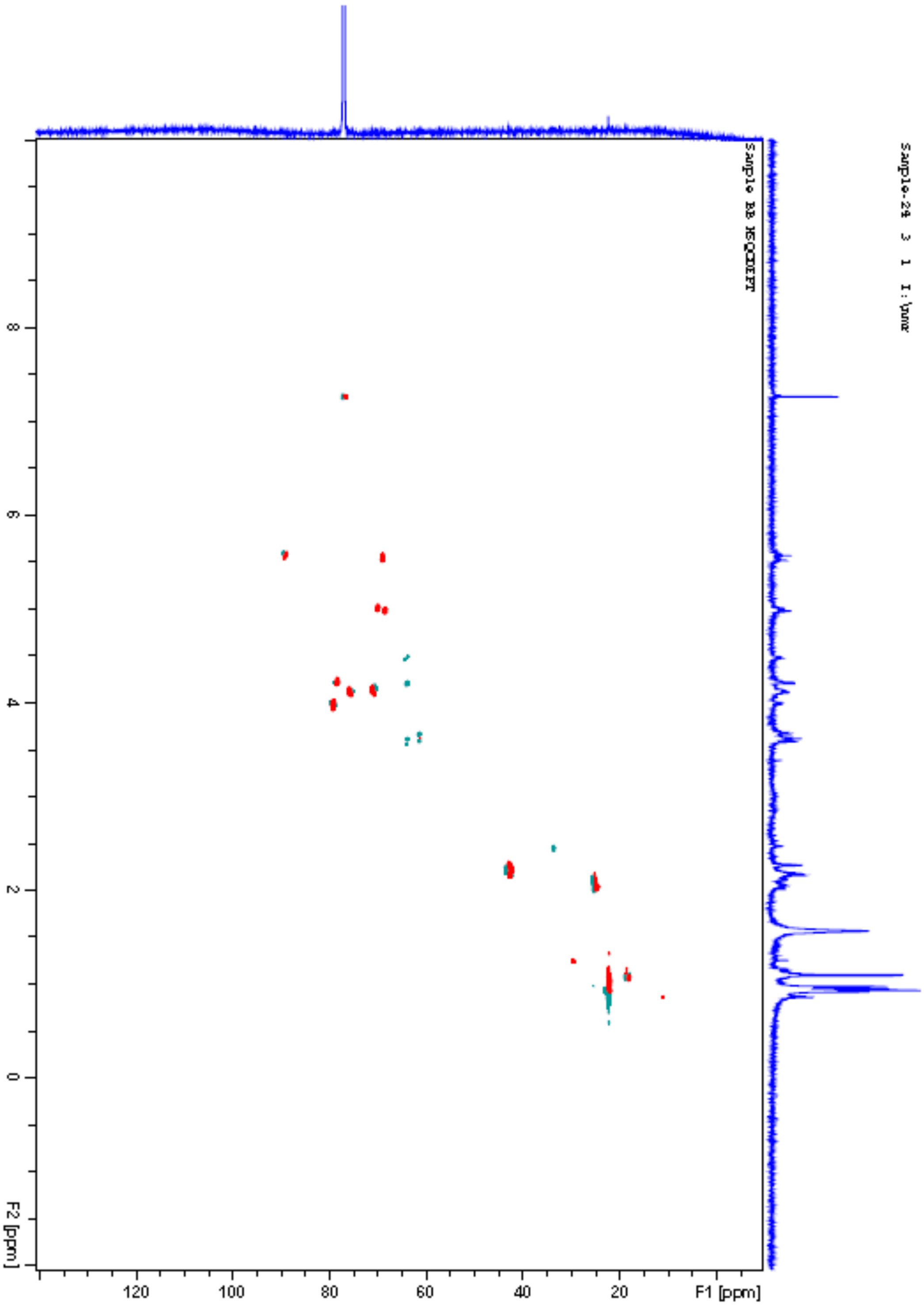
Sample BB HSQCDEPT
Sample-24 3 1 I:\nmr
F1 [ppm]
F2 [ppm]
120
100
80
60
40
20
8
6
4
2
0

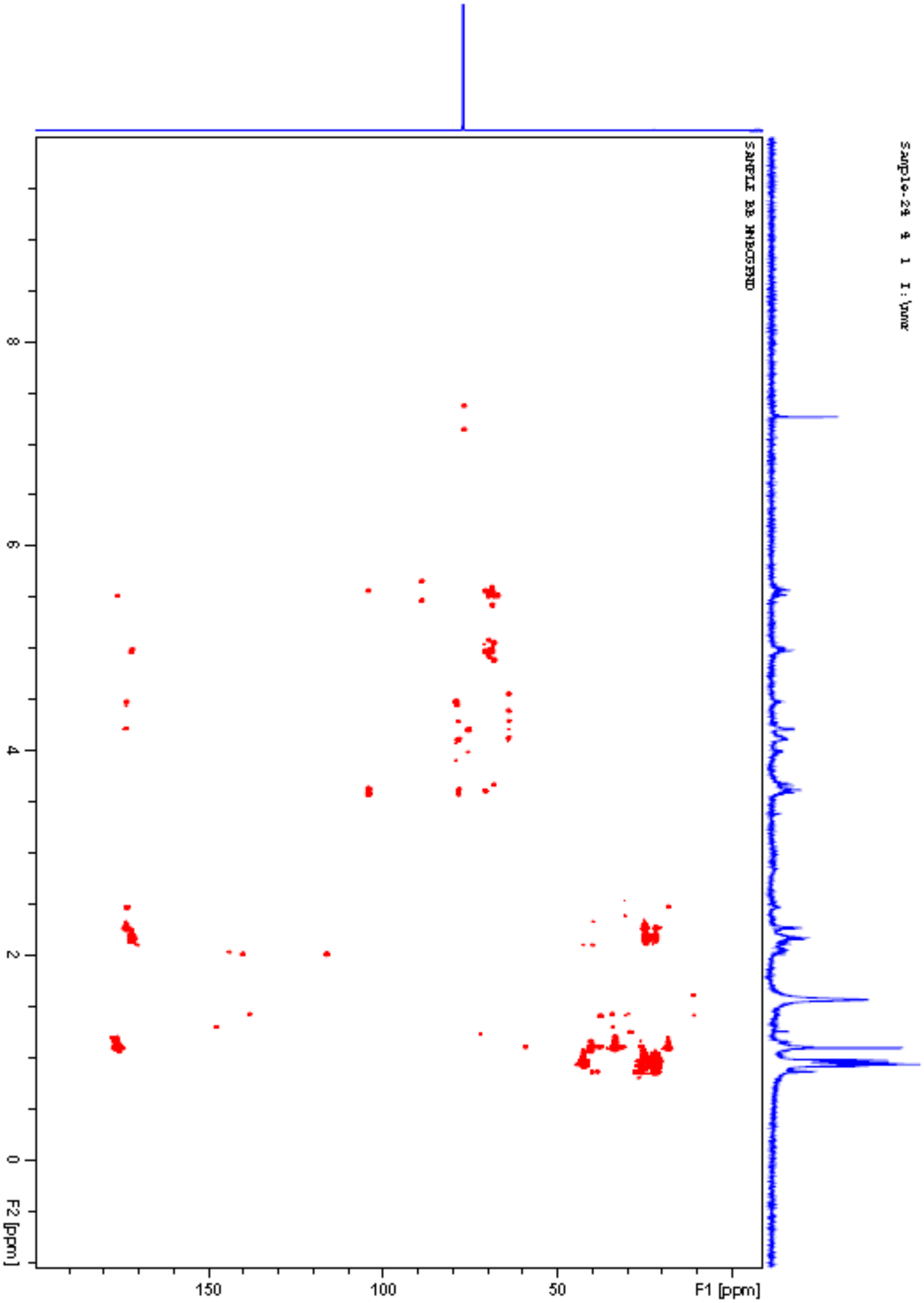
F1 [ppm]
F2 [ppm]

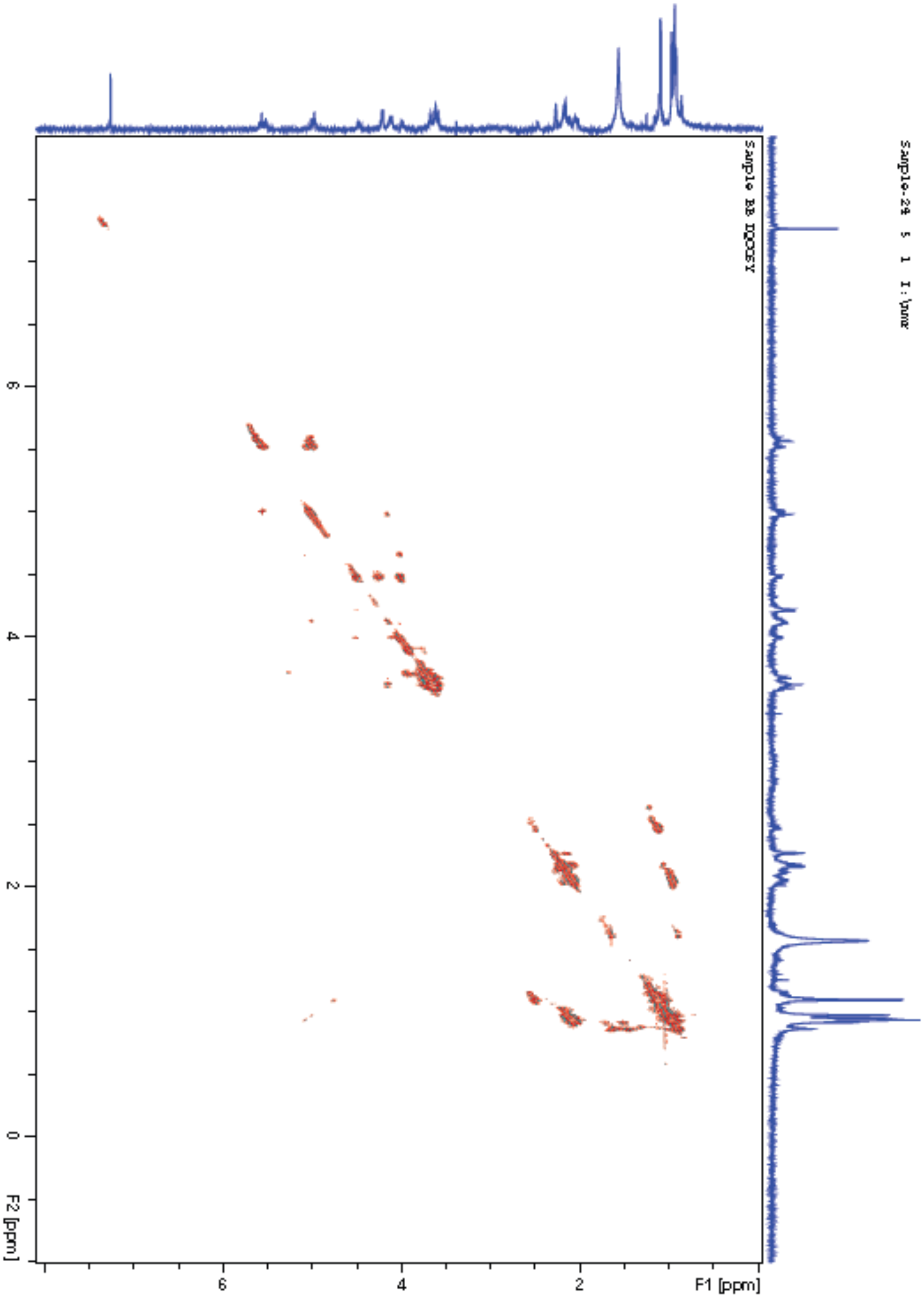

F1 [ppm]
F2 [ppm]

S4:20[6] (5,5,5,5)

Purified from *S. habrochaites* LA1392

HRMS: (ESI) *m/z* calcd for $C_{33}H_{55}O_{17}^{-}$ ([M+HCOO^{-}]): 723.3445, found: 723.3523

Material recovered: ~0.5 mg

NMR solvent: $CDCl_3$

InChI Key: DOOYSSJNUQYOON-FLPSWKLKSA-N

Carbon # (group)	^{1}H (ppm)	^{13}C (ppm)
1(CH)	5.58 (d, *J* = 3.9 Hz)	89.2 ($^{1}J_{CH}$ = 176.1 Hz)
2(CH)	4.99 (dd, *J* = 10.3, 3.8 Hz)	70.1
2-O-		
-1(CO)		172.1
-2(CH_2)	2.18 (m)	42.8
-3(CH)	2.06 (m)	25.2
-4(CH_3) x 2	0.94 (m)	22.3
3(CH)	5.52 (m)	69.3
3-O-		
-1(CO)		175.8
-2(CH)	2.32 (m)	40.5
-2'(CH_3)	1.07 (d, *J* = 6.9 Hz)	16.4
-3(CH_2)	1.40 (m), 1.62 (m)	26.2
-4(CH_3)	0.86 (m)	11.1
4(CH)	4.97 (m)	68.7
4-O		
-1(CO)		172.3
-2(CH_2)	2.18 (m)	42.8

-3(CH)	2.06 (m)	25.2
-4(CH_3) x 2	0.94 (m)	22.3
5(CH)	4.15 (m)	72.0
6(CH_2)	3.61 (m), 3.67 (d, J = 12.7 Hz)	61.5
1' (CH_2)	3.57 (d, J = 12.2 Hz), 3.61 (m)	64.2
2' (C)		104.3
3' (CH)	4.21 (m)	78.6
4' (CH)	4.12 (m)	75.8
5' (CH)	3.98 (m)	79.4
6' (CH_2)	4.20 (m), 4.47 (m)	64.3
3'-O		
-1(CO)		173.5
-2(CH_2)	2.27 (m)	43.0
-3(CH)	2.12 (m)	25.5
-4(CH_2) x 2	0.97 (d, J = 3.7 Hz), 0.97 (d, J = 3.7 Hz)	22.4

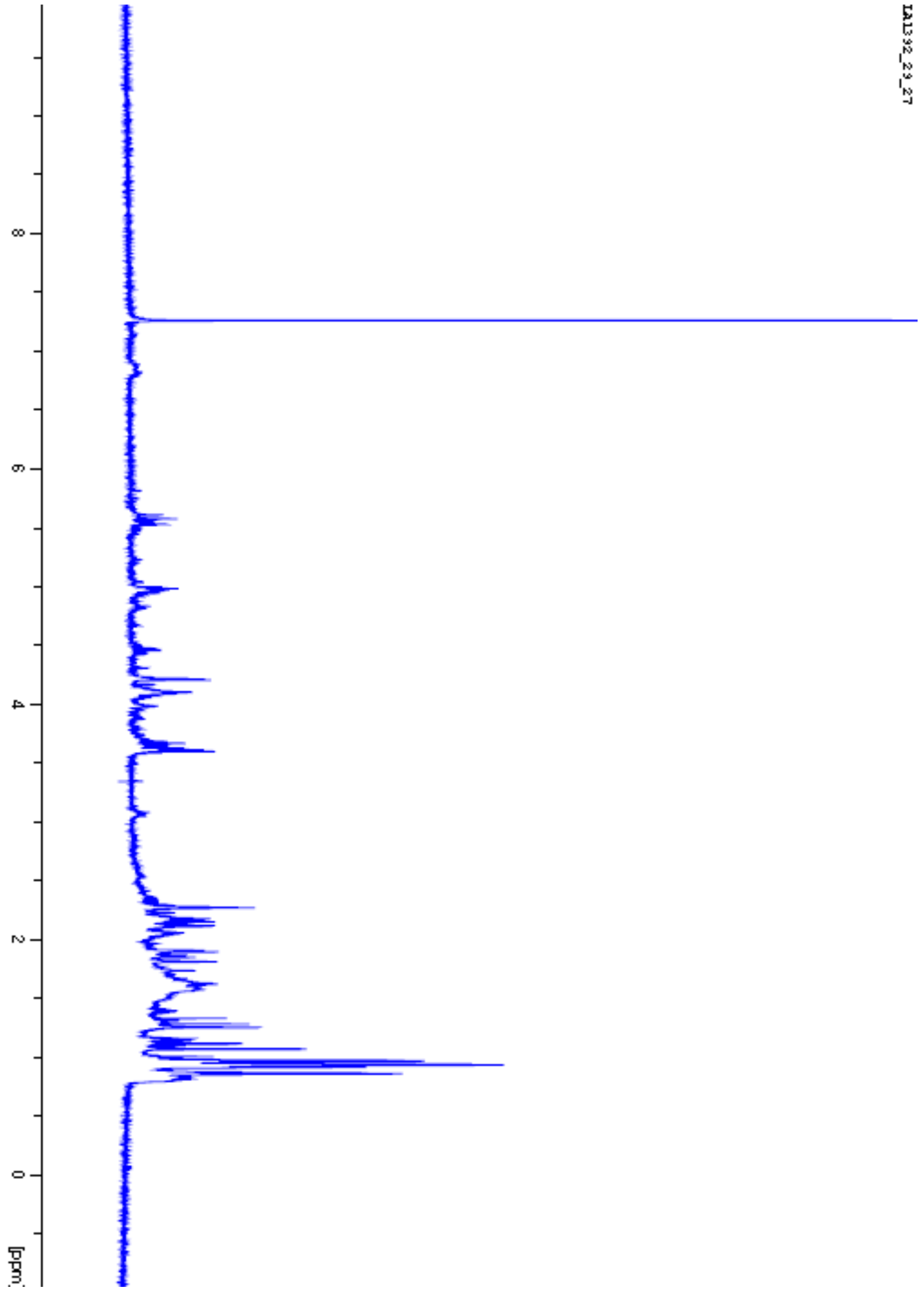
IA1332_29_27
8
6
4
2
0
[ppm]

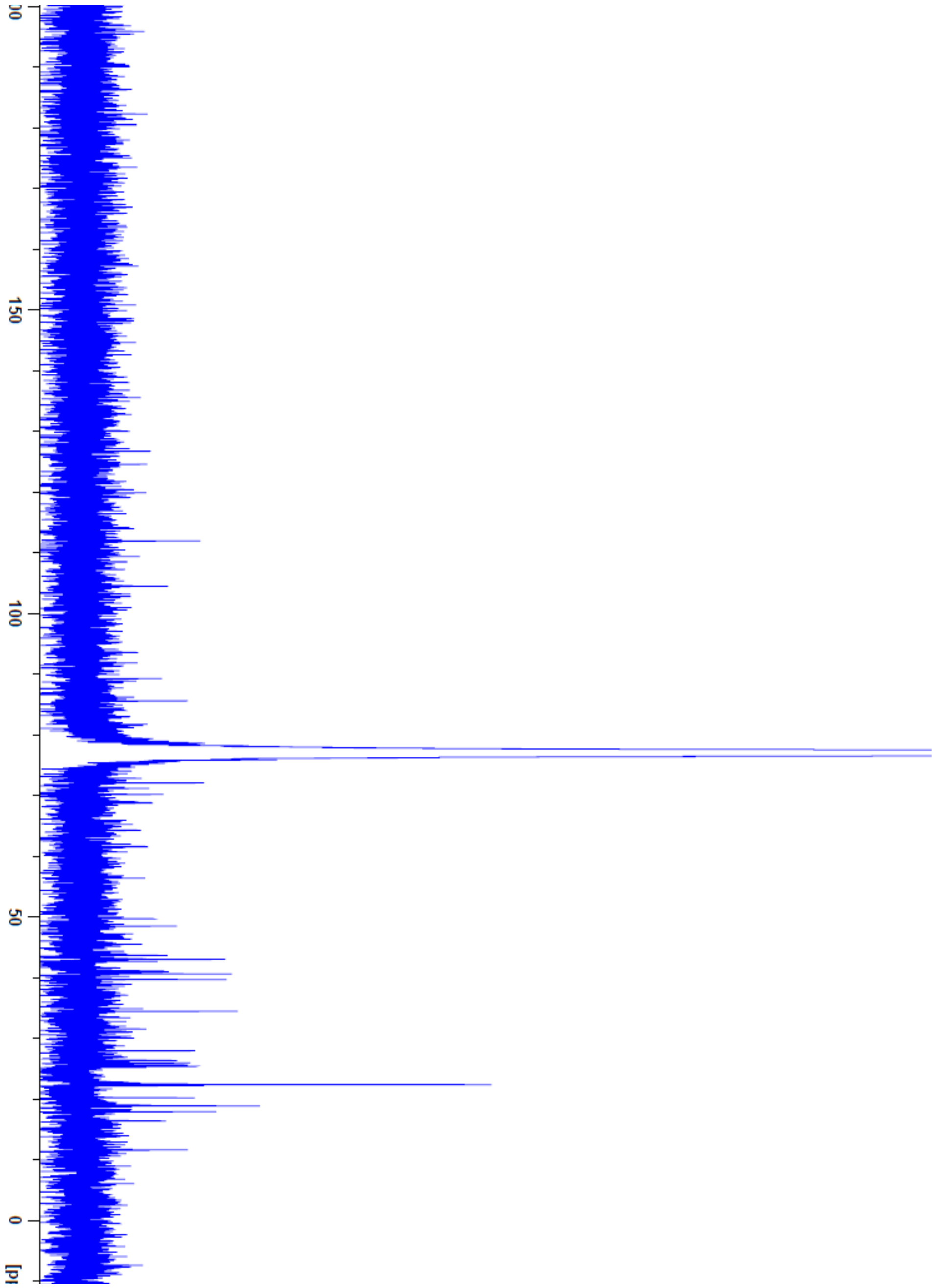
150
100
50
0

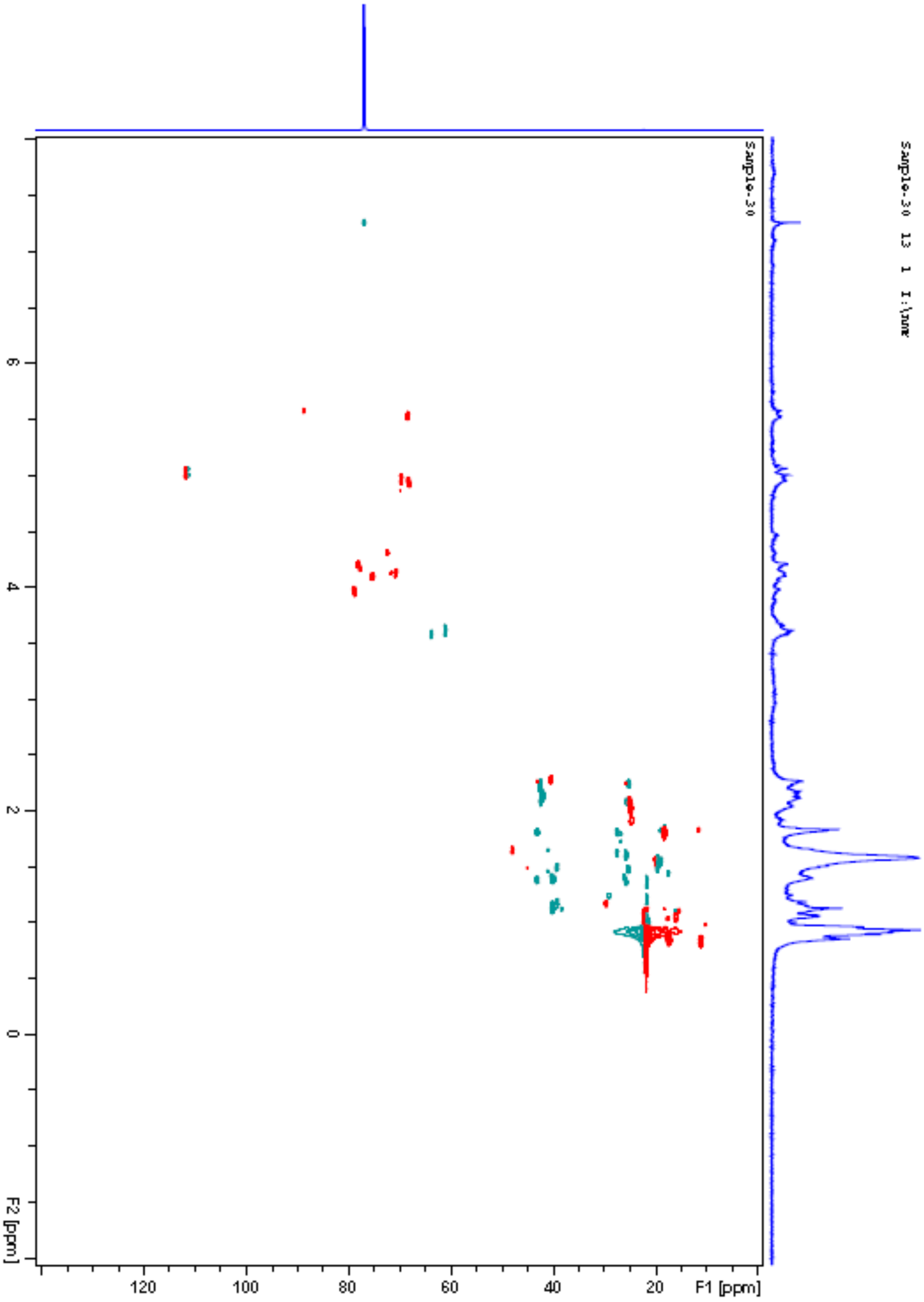
Sample-30
Sample-30 13 1 I:\nmr
F1 [ppm]
F2 [ppm]

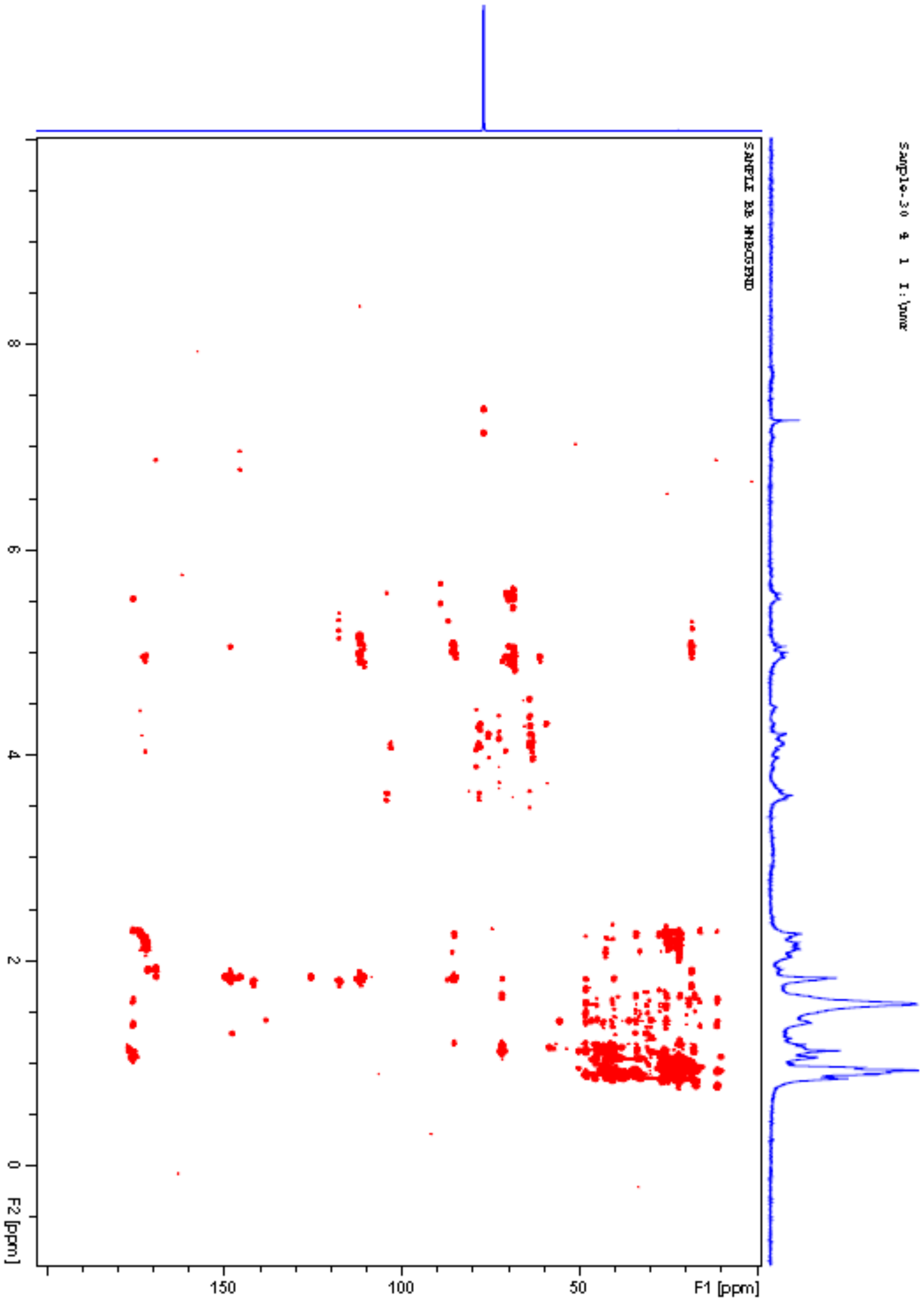

F1 [ppm]
150
100
50
F2 [ppm]
0
2
4
6
8

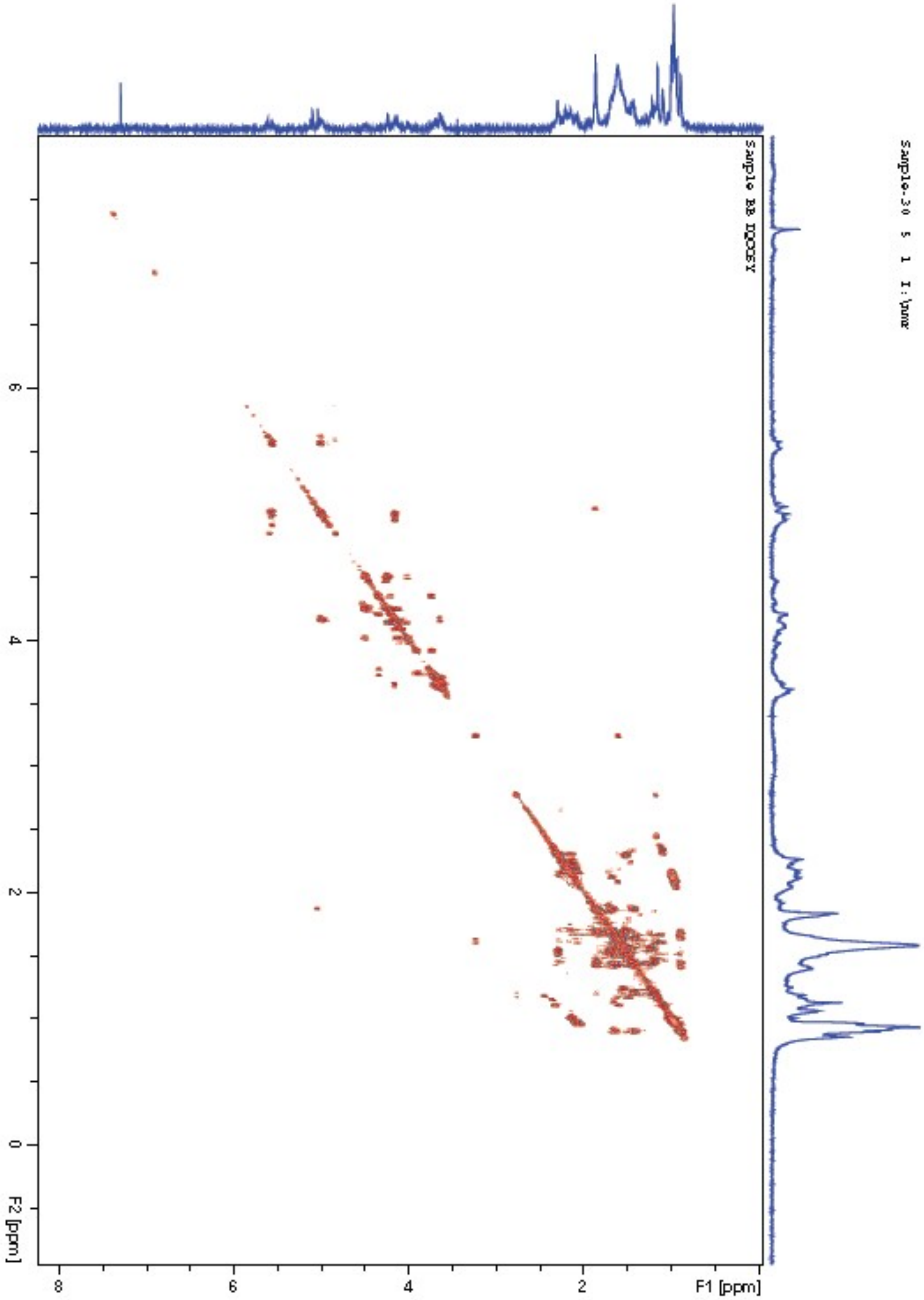
F1 [ppm]
F2 [ppm]

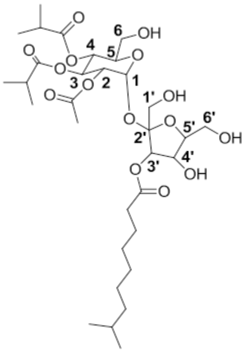

S4:20[7] (2,4,4,10)

Purified from *S. habrochaites* LA1777

HRMS: (ESI) *m/z* calcd for $C_{33}H_{55}O_{17}^{-}$ ([M+HCOO^{-}]): 723.3445, found: 723.3598

Material recovered: 1-2 mg

NMR solvent: $CDCl_3$

InChI Key: SZESHEMALQYQTA-HHWIILEKSA-N

Carbon # (group)	^{1}H (ppm)	^{13}C (ppm)
1(CH)	5.60 (d, J = 3.4 Hz)	89.5 ($^{1}J_{CH}$ = 177.3 Hz)
2(CH)	4.93 (m)	70.4
2-O-		
-1(CO)		170.0
-2(CH_3)	2.03(s)	20.6
3(CH)	5.45 (t, J = 9.9 Hz)	68.8
3-O-		
-1(CO)		175.8
-2(CH)	2.48 (m)	33.9
-3(CH_3) x 2	1.12 (d, J = 7.0 Hz), 1.15 (d, J = 7.0 Hz)	18.7, 18.8
4(CH)	4.93 (m)	68.2
4-O		
-1(CO)		176.0
-2(CH_2)	2.52 (m)	33.5
-3(CH) x 2	1.08 (d, J = 6.7 Hz), 1.09 (d, J = 6.7 Hz)	18.9, 19.0
5(CH)	4.14 (m)	72.2
6(CH_2)	3.60 (m)	61.6
1' (CH_2)	3.52 (d, J = 12.2 Hz), 3.63 (d, J = 12.3 Hz)	64.6

2' (C)		104.0
3' (CH)	5.16 (d, J = 8.1 Hz)	80.0
3'-O		
-1(CO)		175.1
-2(CH_2)	2.52 (m)	34.0
-3(CH_2)	1.72 (quin, J = 7.4 Hz)	24.8
-4(CH_2)	1.38 (m)	29.1
-5(CH_2)	1.30 (m)	29.5
-6(CH_2)	1.30 (m)	27.3
-7(CH_2)	1.16[a]	39.0
-8(CH_2)	1.52 (m)	27.9
-9(CH_3) x 2	0.86 (d, J = 6.6 Hz)	22.6
4' (CH)	4.61 (t, J = 8.2 Hz)	71.3
5' (CH)	3.93 (d, J = 8.1 Hz)	82.4
6' (CH_2)	3.71 (d, J = 12.8 Hz), 3.90 (*d*, J = 12.8 Hz)	59.6

[a]Determined by COSY.

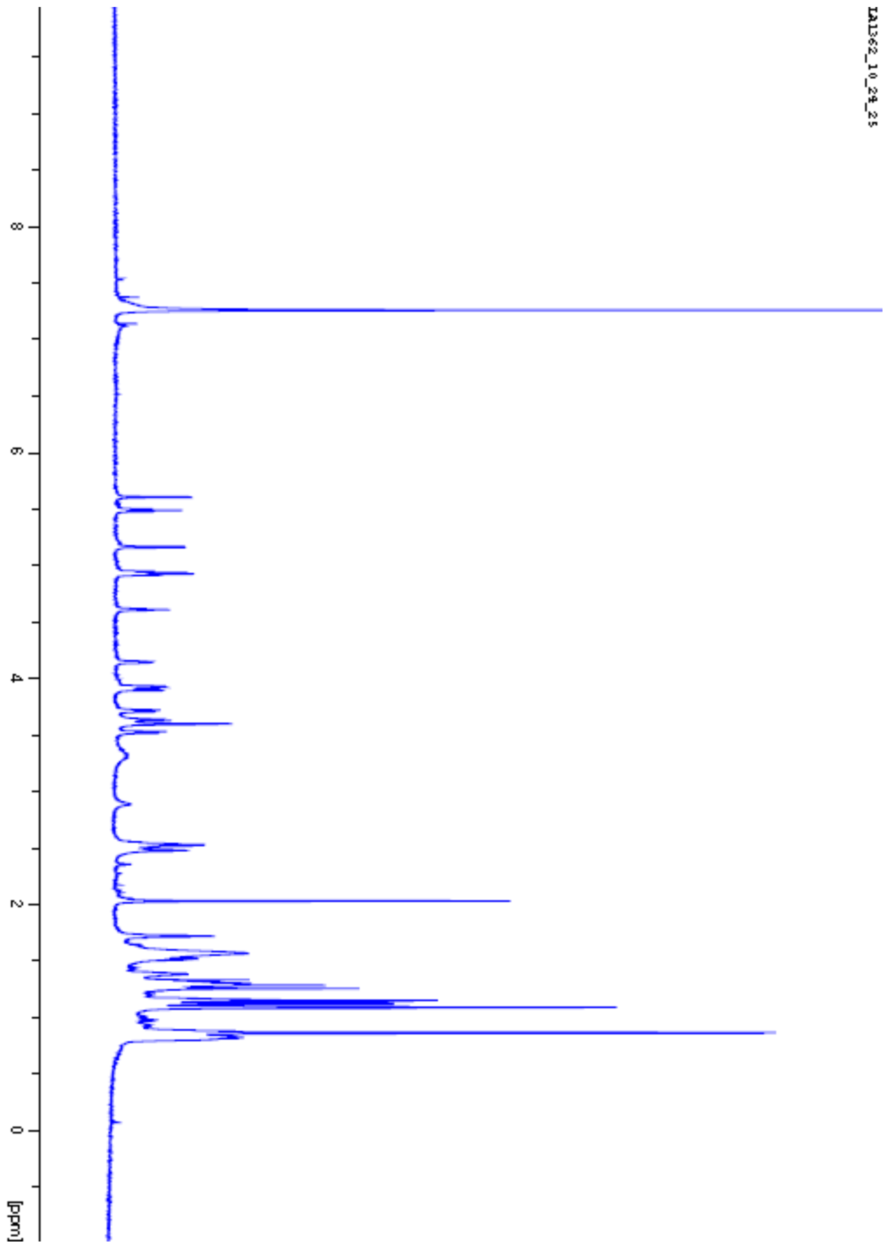
IA1362_10_24_25
8
6
4
2
0
[ppm]

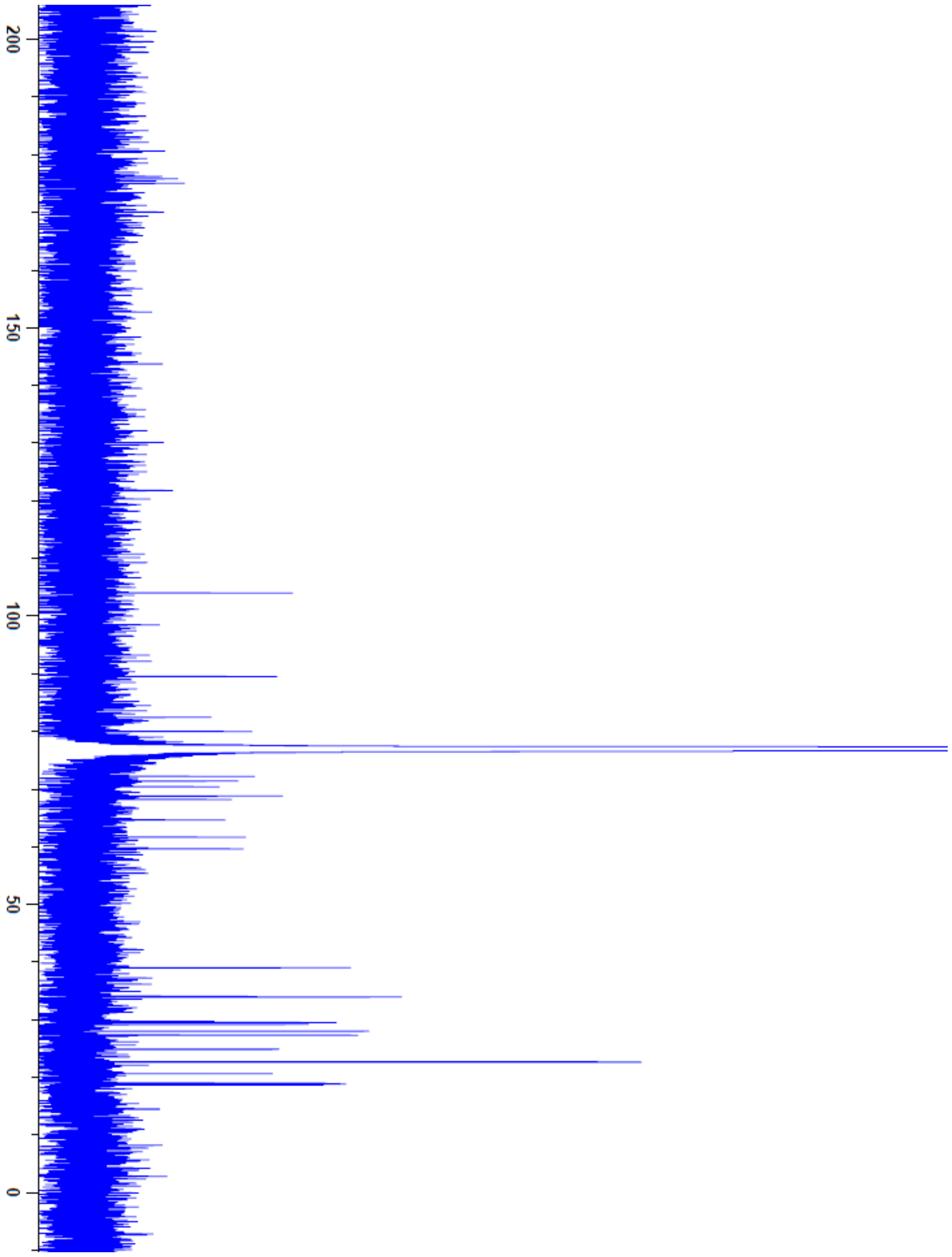
200
150
100
50
0

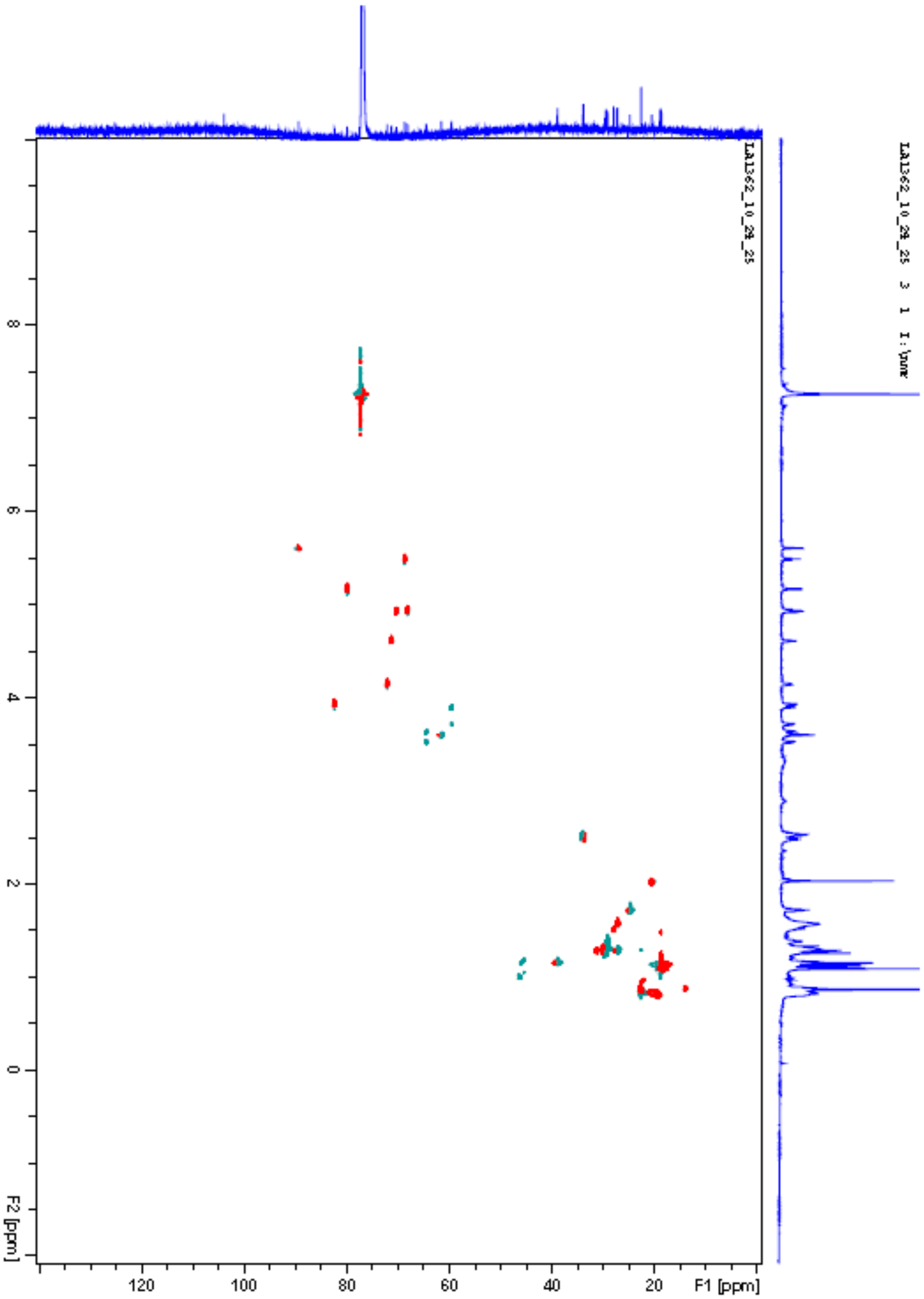

F1 [ppm]
F2 [ppm]

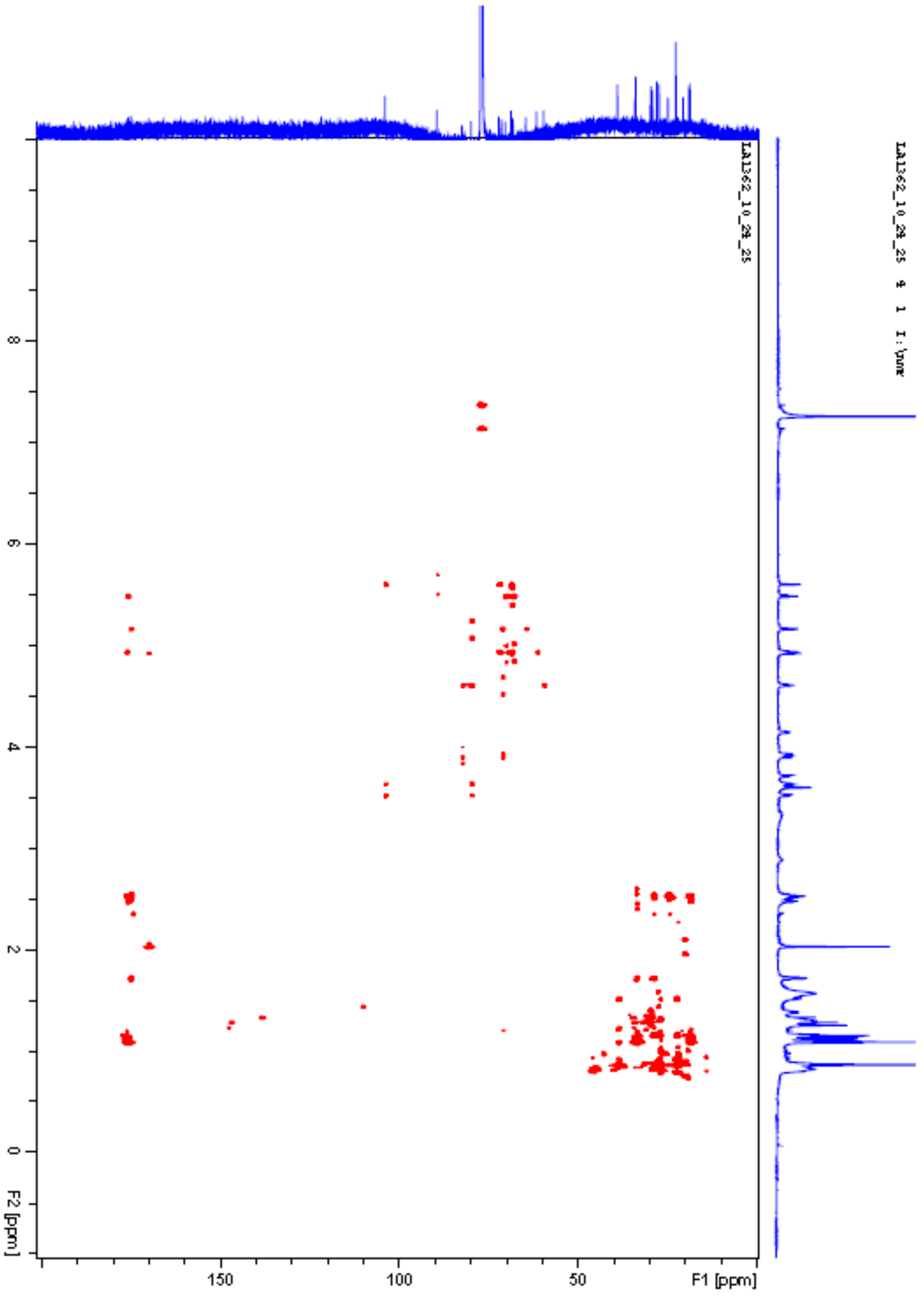

LA1362_10_24_25
LA1362_10_24_25 4 1 I:\nmr
F2 [ppm]
F1 [ppm]

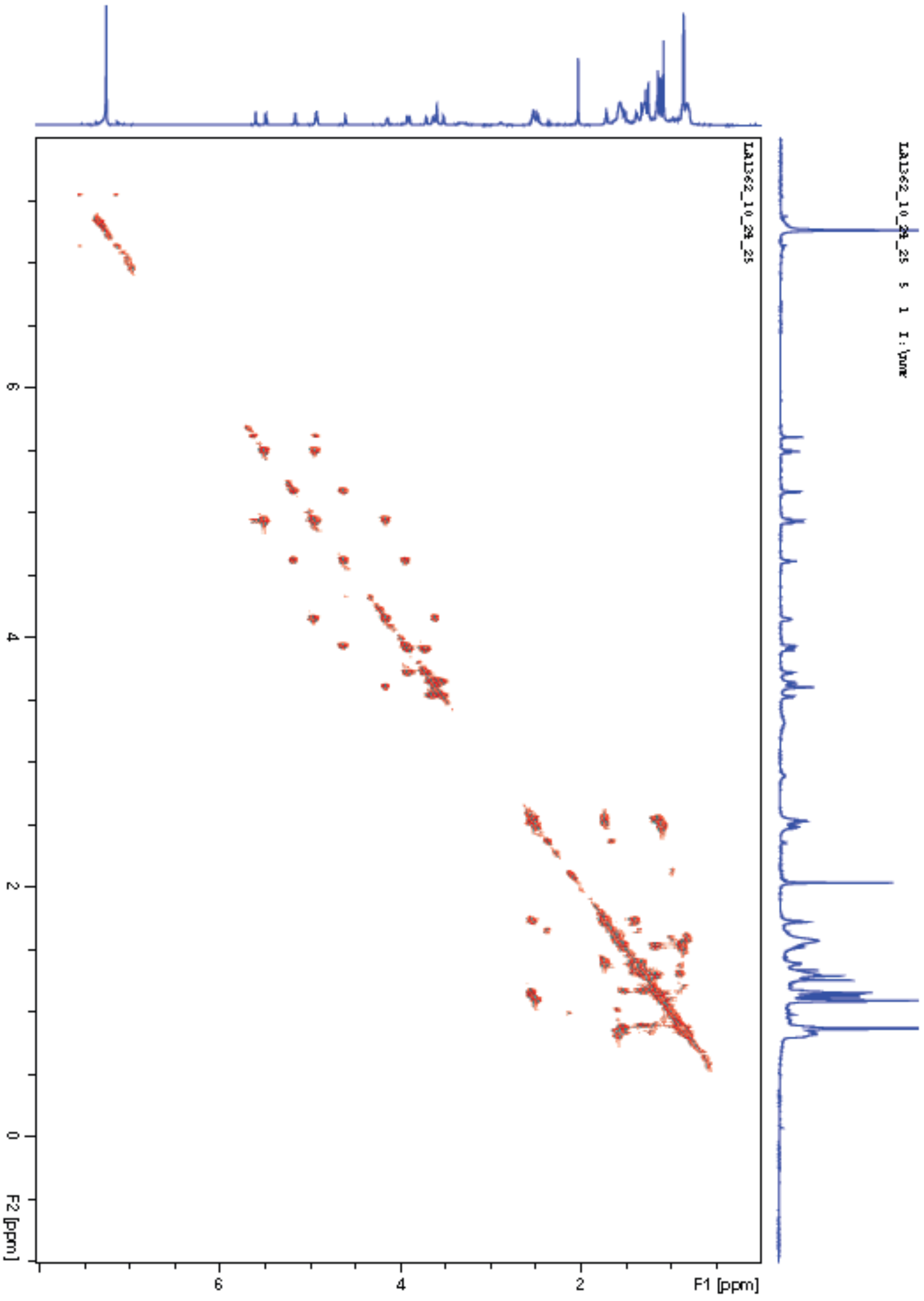
LA1362_10_24_25
F1 [ppm]
F2 [ppm]

S4:21[2] (2,4,5,10)

Purified from *S. habrochaites* LA1777

HRMS: (ESI) *m/z* calcd for $C_{34}H_{57}O_{17}^{-}$ [M+HCOO^{-}]: 737.3601, found: 737.3712

Material recovered: 2-3 mg

NMR solvent: CD_3OD

InChI Key: POOTYPRTOUAUAE-PKUDYEBGSA-N

Carbon # (group)	^{1}H (ppm)	^{13}C (ppm)
1(CH)	5.64 (d, J = 3.4)	90.6 ($^{1}J_{CH}$ = 172.0 Hz)
2(CH)	4.88[a]	72.0
2-O-		
-1(CO)		172.0
-2(CH_3)	2.00 (s)	20.9
3(CH)	5.43 (t, J = 10.0 Hz)	71.4
3-O-		
-1(CO)		177.9
-2(CH)	2.48 (m)	35.3
-3(CH_3) x 2	1.08 (d, J = 7.1 Hz), 1.10 (d, J = 7.1 Hz)	19.2, 19.2
4(CH)	5.12 (t, J = 9.7 Hz)	69.7
4-O		
-1(CO)		173.6
-2(CH_2)	2.15 (dd, J = 15.0, 7.1 Hz), 2.21 (dd, J = 15.0, 7.1 Hz) [b]	44.2
-3(CH)	2.03 (m)	26.7
-4(CH_3) x 2	0.95 (d, J = 6.2 Hz), 0.95 (d, J = 6.2 Hz)	22.9, 22.9
5(CH)	4.12 (m)	72.1

6(CH_2)	3.56[c], 3.67 (d, J = 12.2 Hz)	61.7
1' (CH_2)	3.44 (d, J = 12.2 Hz), 3.58 (d, J = 12.2 Hz)	64.8
2' (C)		105.1
3' (CH)	5.38 (d, J = 8.3 Hz)	79.0
3'-O		
-1(CO)		175.3
-2(CH_2)	2.50 (m)	35.1
-3(CH_2)	1.72 (m)	26.3
-4(CH_2)	1.41 (br. m)	30.5
-5-6(CH_2-CH_2)	1.34 (br. m)	28.7, 30.8
-7(CH_2)	1.20 (br. m)	40.4
-8(CH)	1.54 (m)	29.3
-9(CH_3) x 2	0.89 (d, J = 6.6 Hz), 0.89 (d, J = 6.6 Hz)	23.2, 23.2
4' (CH)	4.28 (t, J = 8.3 Hz)	73.6
5' (CH)	3.88 (br. m)	82.3
6' (CH_2)	3.78 (m)	63.2

[a]Determined by COSY

[b]Higher order multiplet derived from the constants using gNMR

[c]Determined by COSY and HSQC.

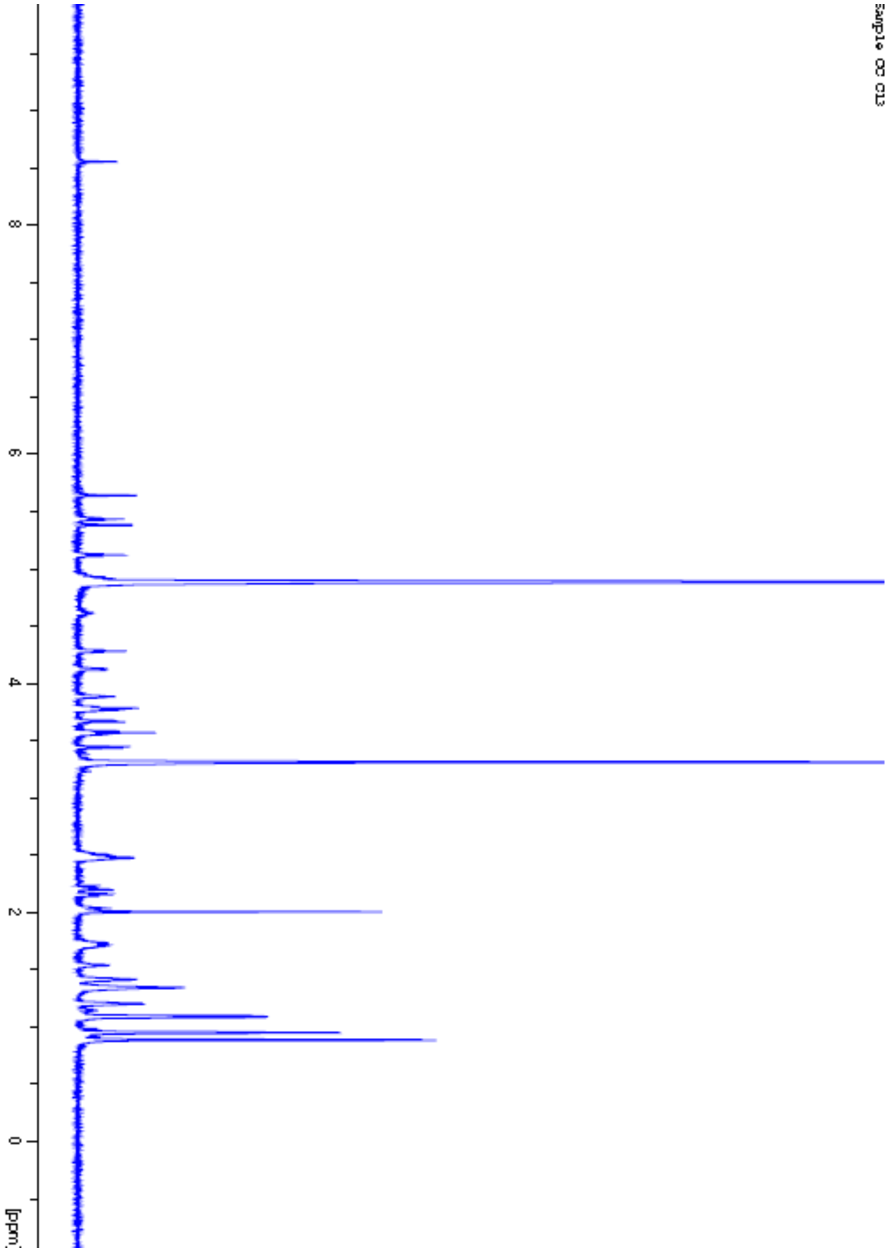
8
6
4
2
0
[ppm]

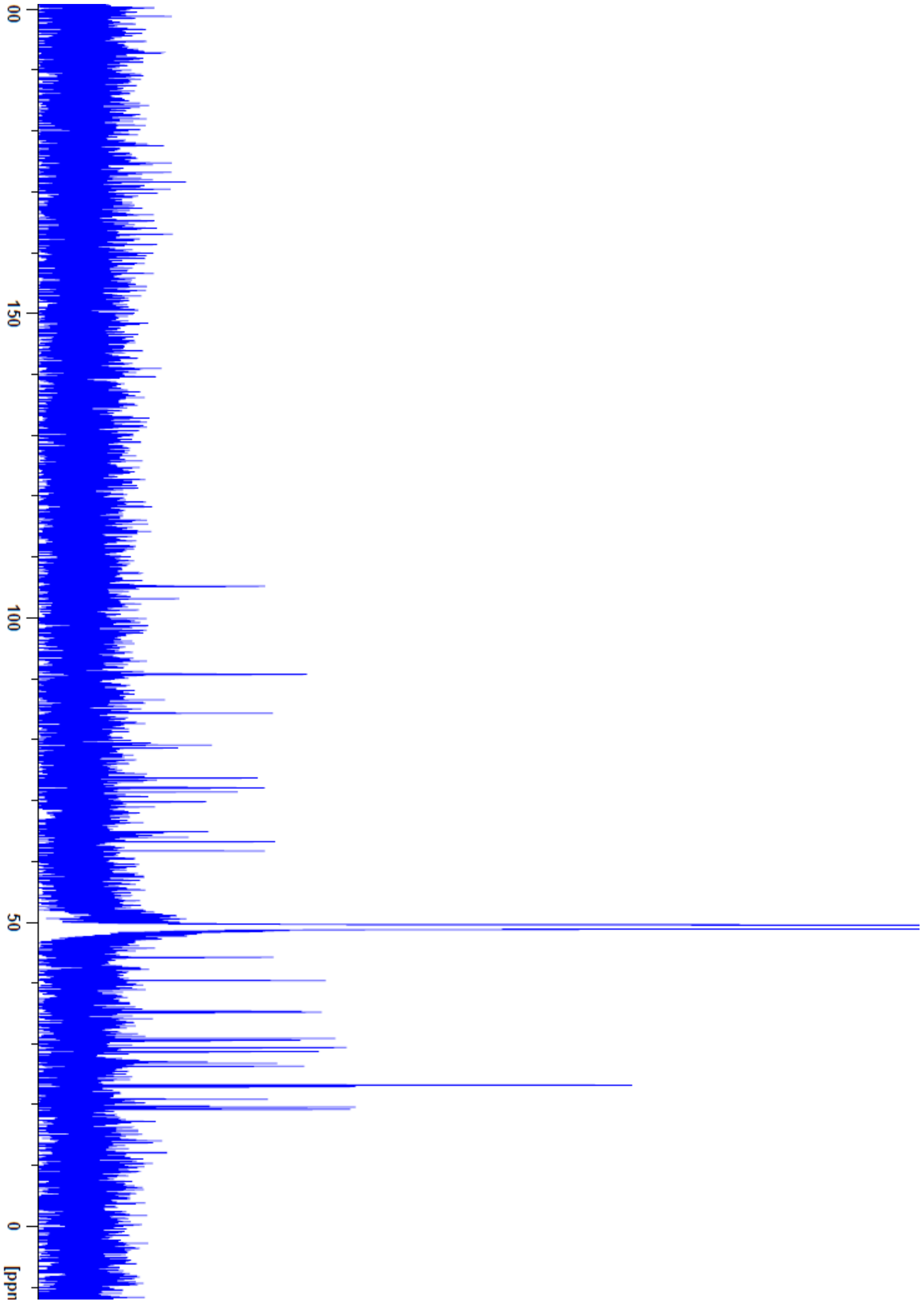
150
100
50
0

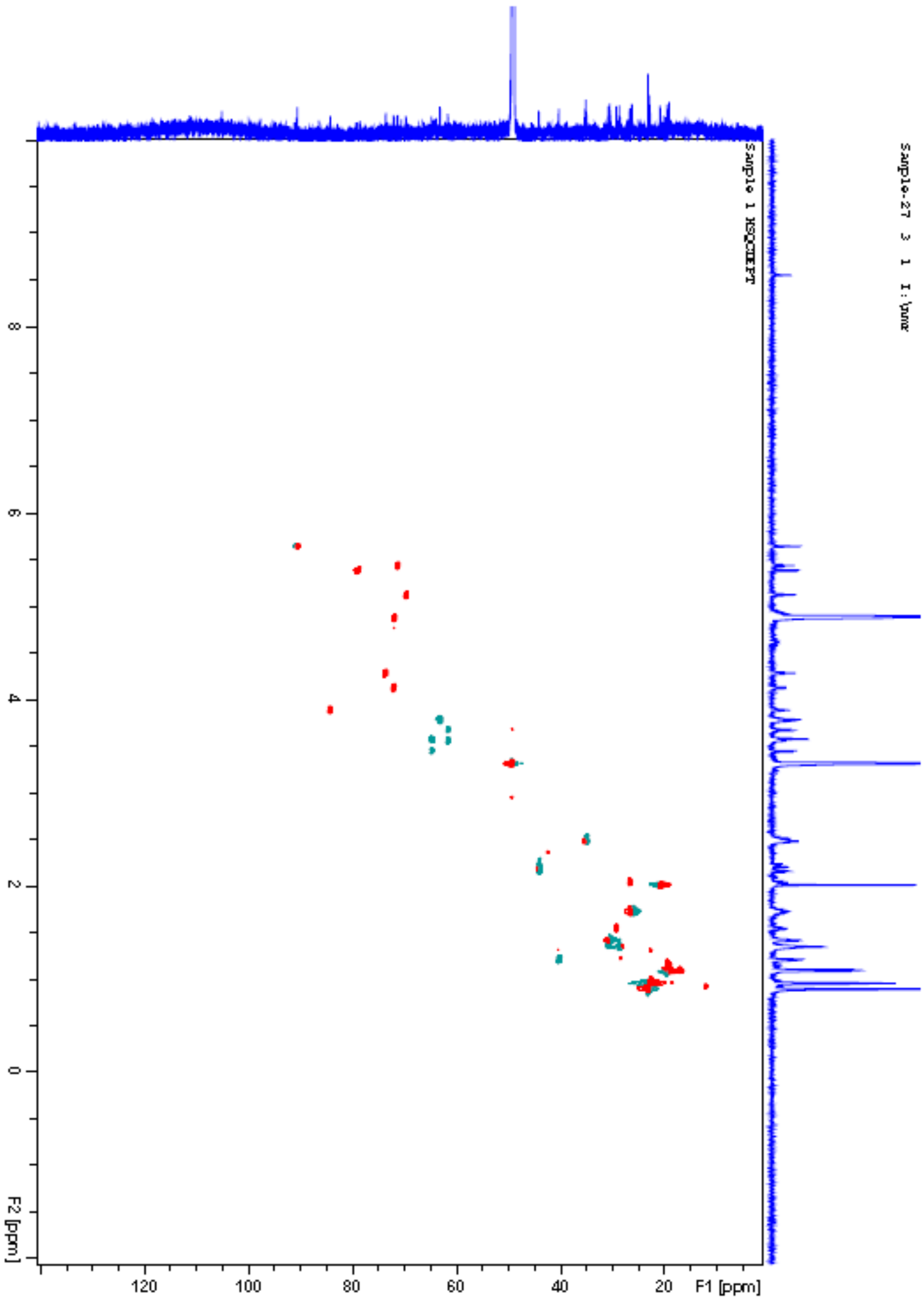
Sample 1 HSQCDEPT
F1 [ppm]
F2 [ppm]

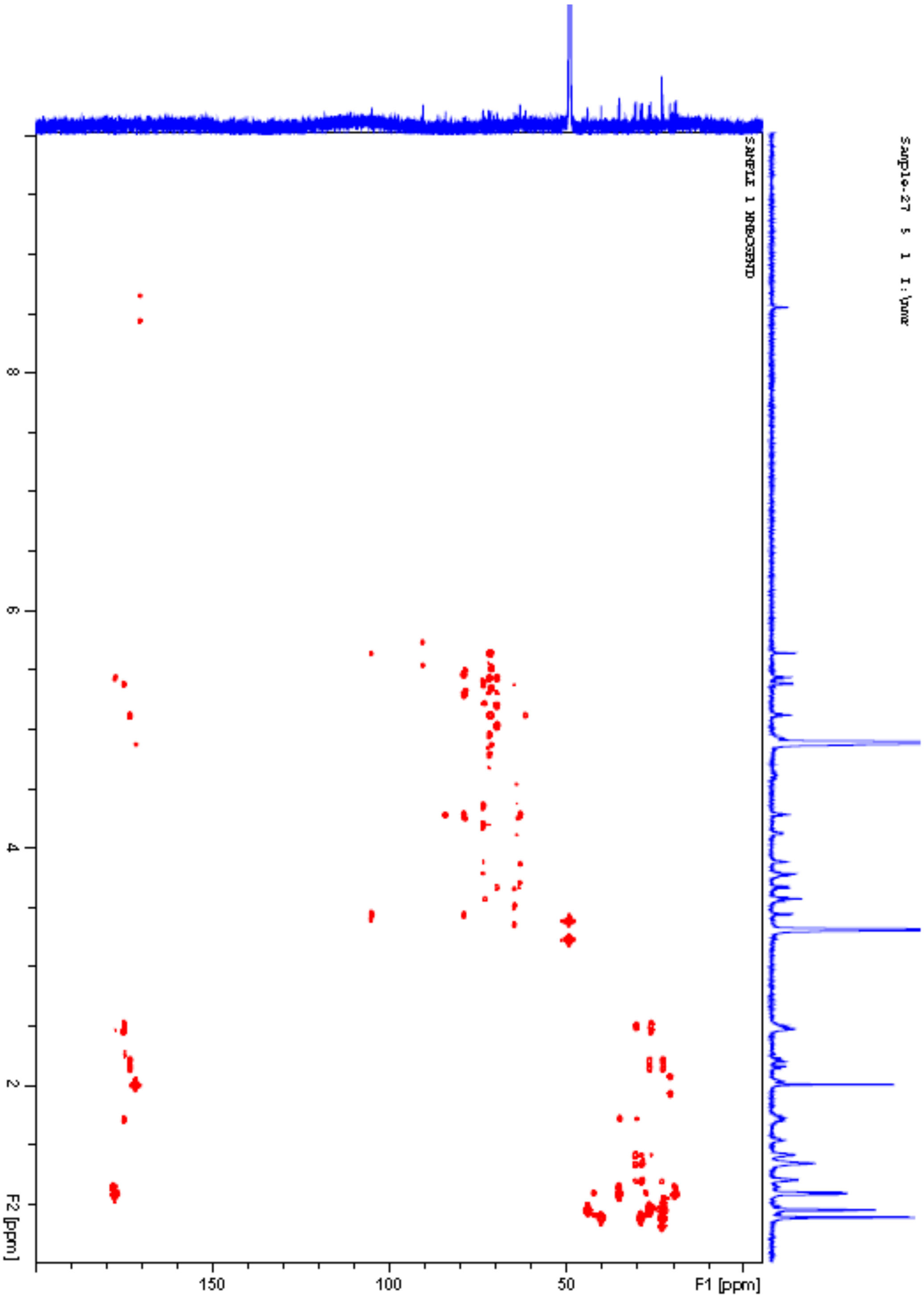
8
6
4
2
F2 [ppm]
150
100
50
F1 [ppm]

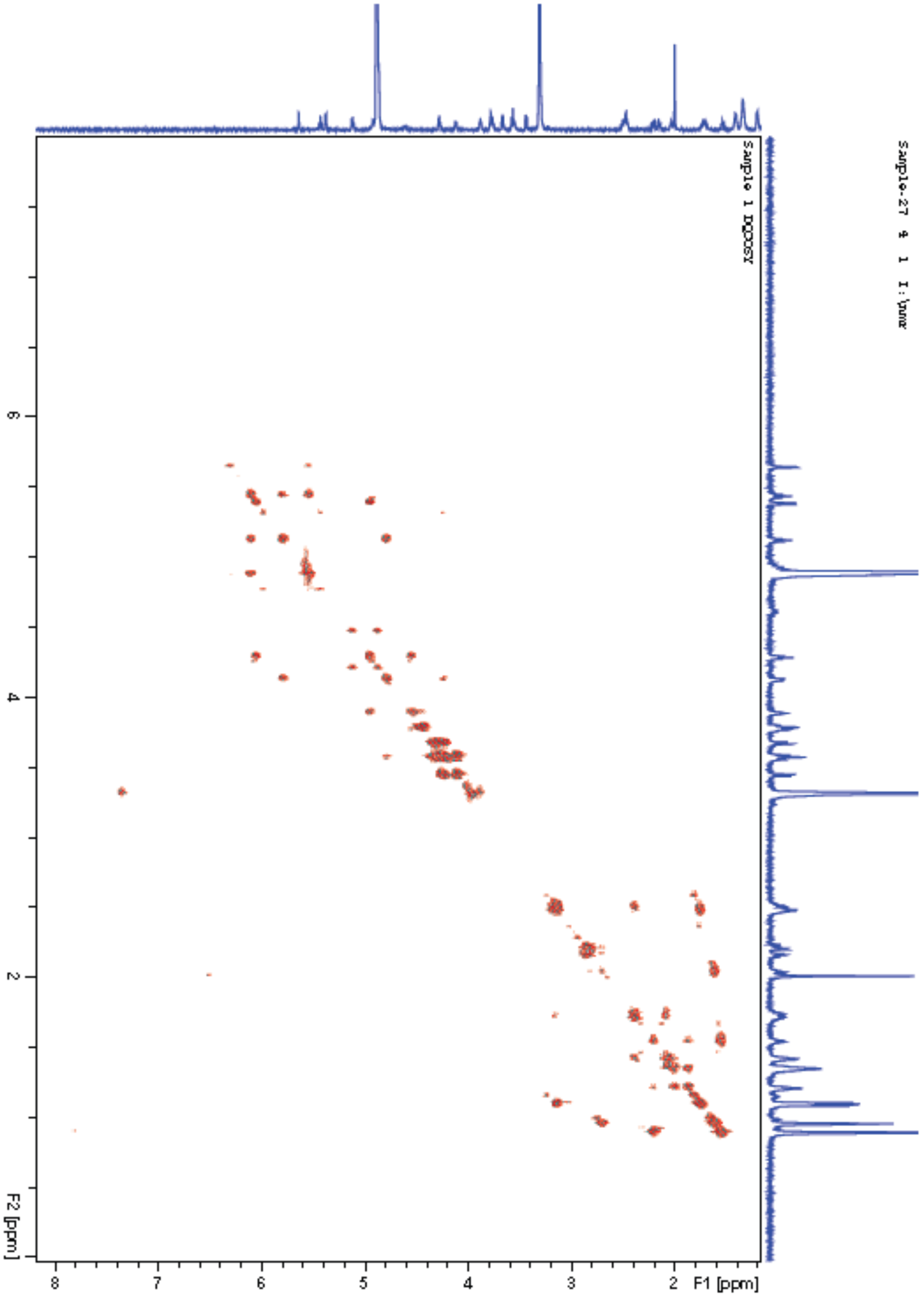
Sample 1 DQCOSY
F1 [ppm]
F2 [ppm]

**S4:22[2]** (2,5,5,10)<br>**Purified from *S. habrochaites* LA1777**<br>**HRMS:** (ESI) *m/z* calcd for $C_{35}H_{59}O_{17}^{-}$ (M+HCOO^{-}]): 751.3758, found: 751.3755<br>**Material recovered:** 3-5 mg<br>**NMR solvent**: $CDCl_3$<br>**InChI Key**: WSPCQIBMNUBQFE-DHWUKAANSA-N		
Carbon # (group)	^{1}H (ppm)	^{13}C (ppm)
1(CH)	5.61 (d, *J* = 3.6 Hz)	89.4
2(CH)	4.86 (dd, *J* = 10.4, 3.7 Hz)	70.5
2-O-		
-1(CO)		170.1
-2(CH_3)	2.00 (s)	20.6
3(CH)	5.47 (t, *J* = 9.8 Hz)	68.6
3-O-		
-1(CO)		175.9
-2(CH)	2.29 (m)	40.9
-2(CH_3)	1.04 (d, *J* = 7.0 Hz)	16.4
-3(CH_2)	1.39 (m), 1.57 (m)	26.5
-4(CH_3)	0.83(m)[a]	11.5
4(CH)	4.91 (m)	68.4
4-O		
-1(CO)		172.2
-2(CH_2)	2.16 (m)	42.9
-3(CH)	2.03 (m)	25.3
-4(CH_3) x 2	0.92 (*m*), 0.92 (m)	22.3, 22.3
5(CH)	4.11 (m)	71.9

6(CH_2)	3.59 (m)	61.6
1' (CH_2)	3.51[b], 3.63[b]	64.5
2' (C)		104.0
3' (CH)	5.13 (d, *J* = 7.5 Hz)	79.8
3'-O		
-1(CO)		175.4
-2(CH_2)	2.50 (m)	34.2
-3(CH_2)	1.68[a]	24.9
-4(CH_2)	1.35 (m)[a]	29.2
-5(CH_2)	1.28 (m)[a]	29.5
-6(CH_2)	1.28 (m)[a]	27.3
-7(CH_2)	1.13 (m)	39.0
-8(CH)	1.49 (m)	28.0
-9(CH_3) x 2	0.84 (m)[a], 0.84 (m)[a]	22.7, 22.7
4' (CH)	4.55 (t, *J* = 7.4 Hz)	71.4
5' (CH)	3.90 (m)[a]	82.3
6' (CH_2)	3.69 (d, *J* = 11.6 Hz), 3.87 (d, *J* = 13.7 Hz)	59.8

[a]Determined by COSY

[b]Determined by COSY and HSQC.

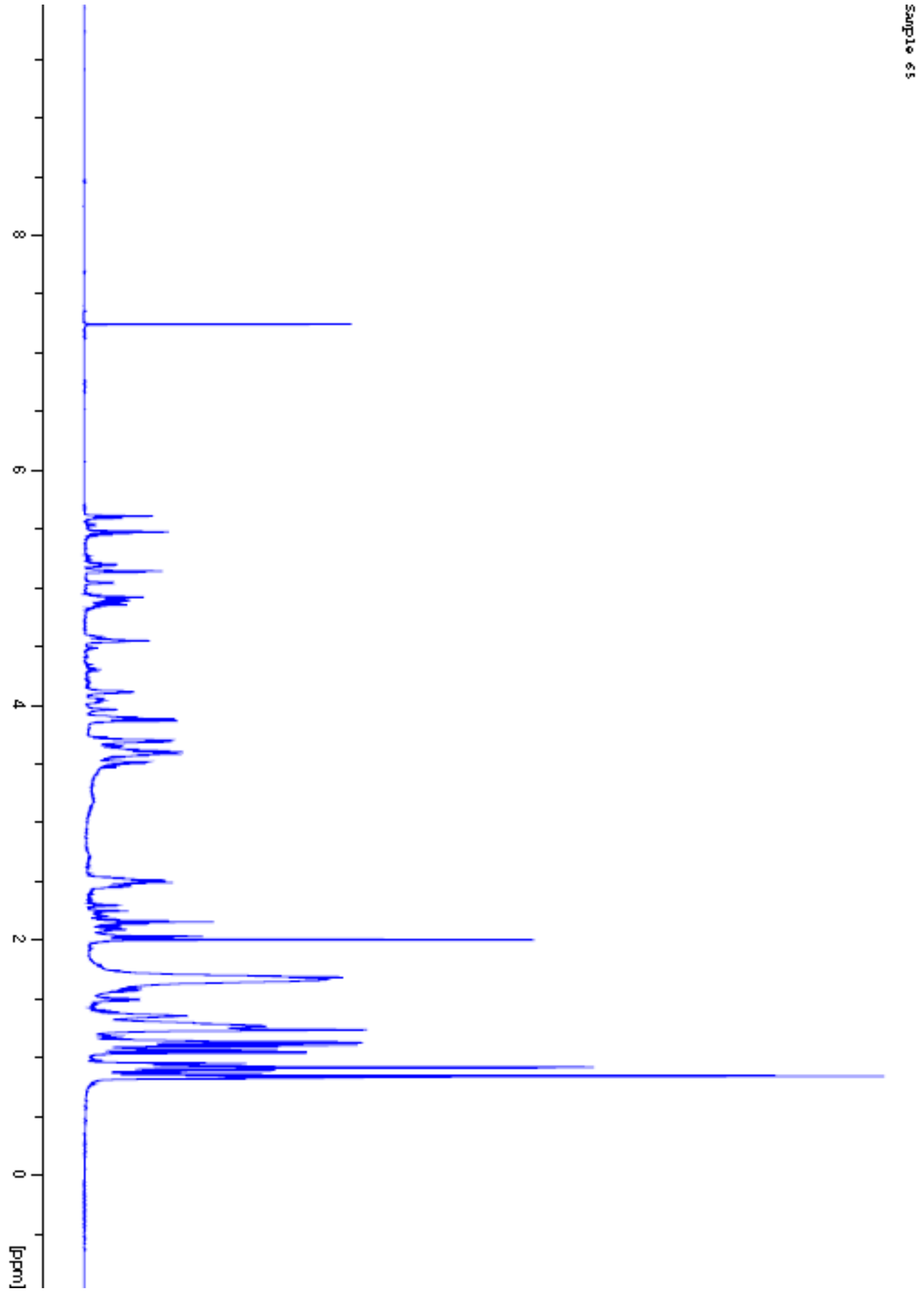
Sample 65
8
6
4
2
0
[ppm]

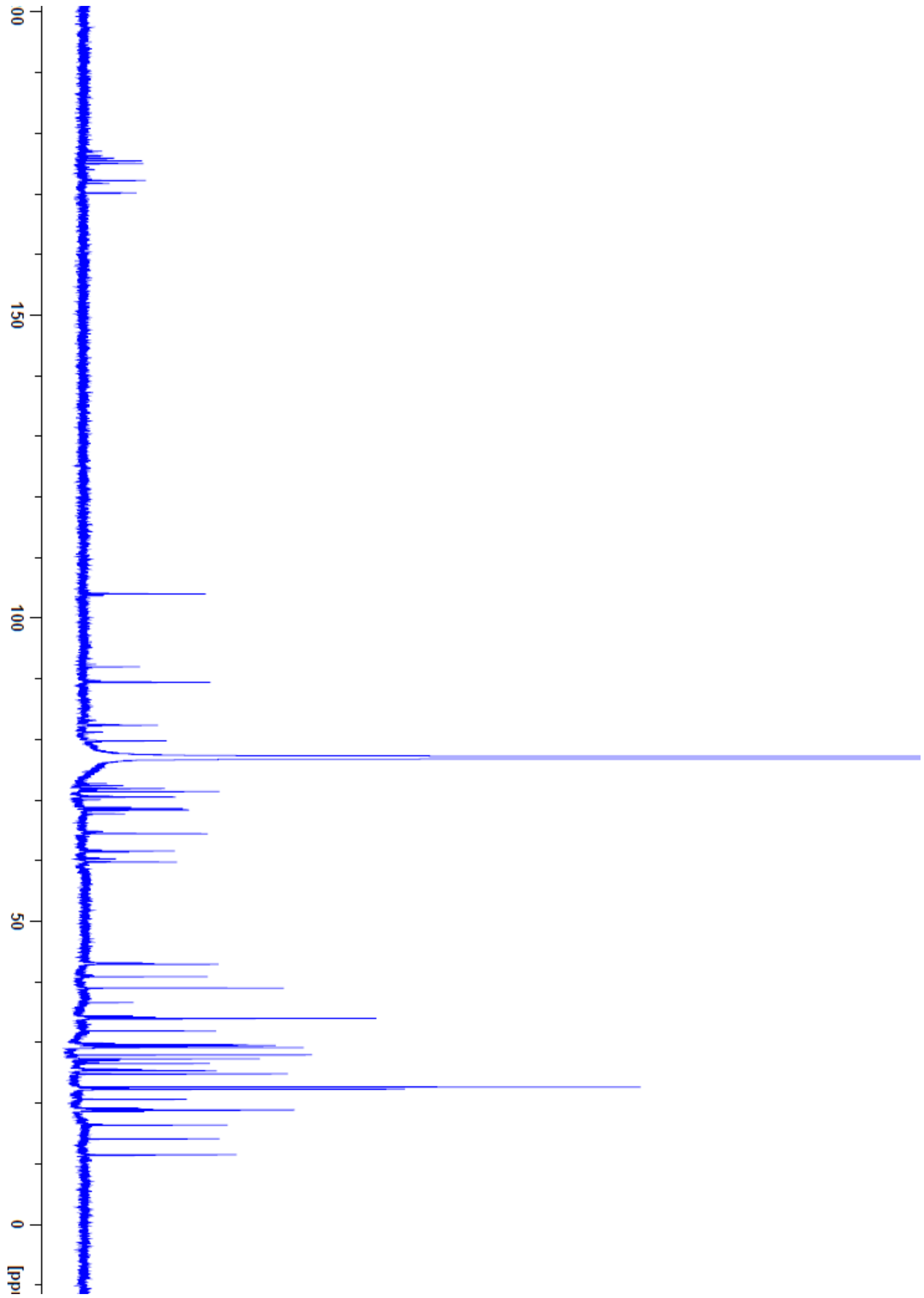
150
100
50
0

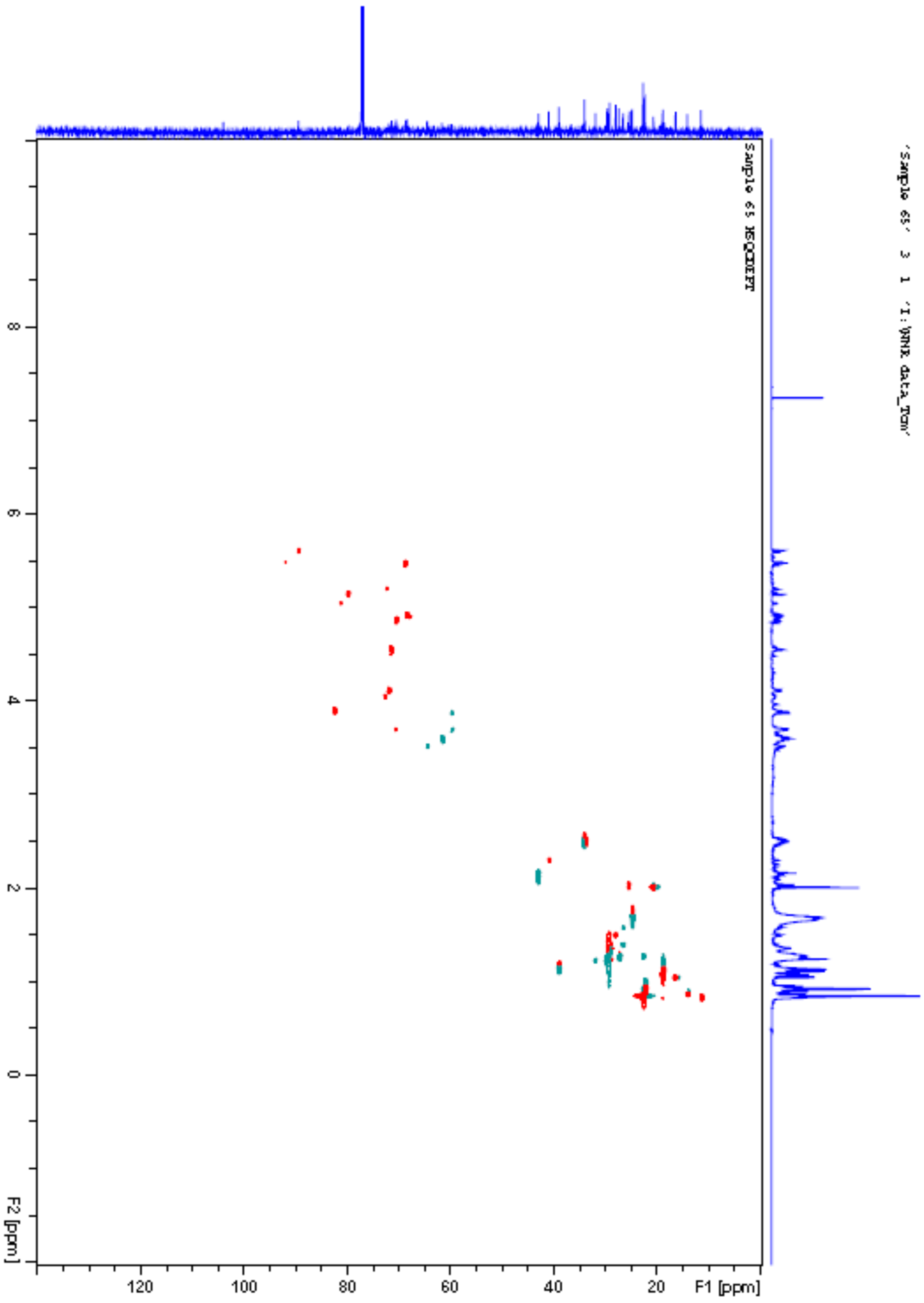
F2 [ppm]
F1 [ppm]

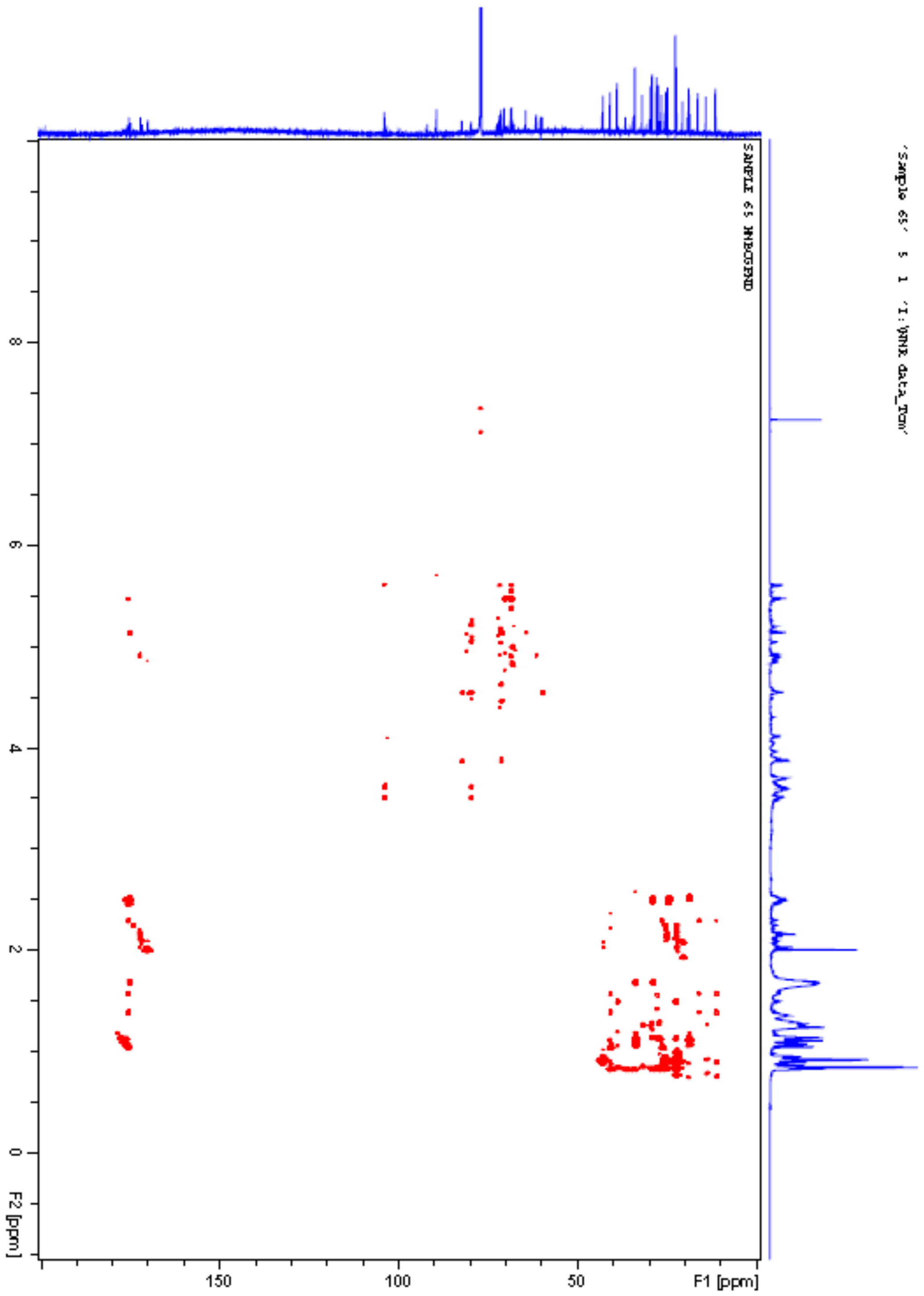

'Sample 65' 5 1 'I:\NMR data_Tom'
SAMPLE 65 HMBCGPND
F1 [ppm]
F2 [ppm]

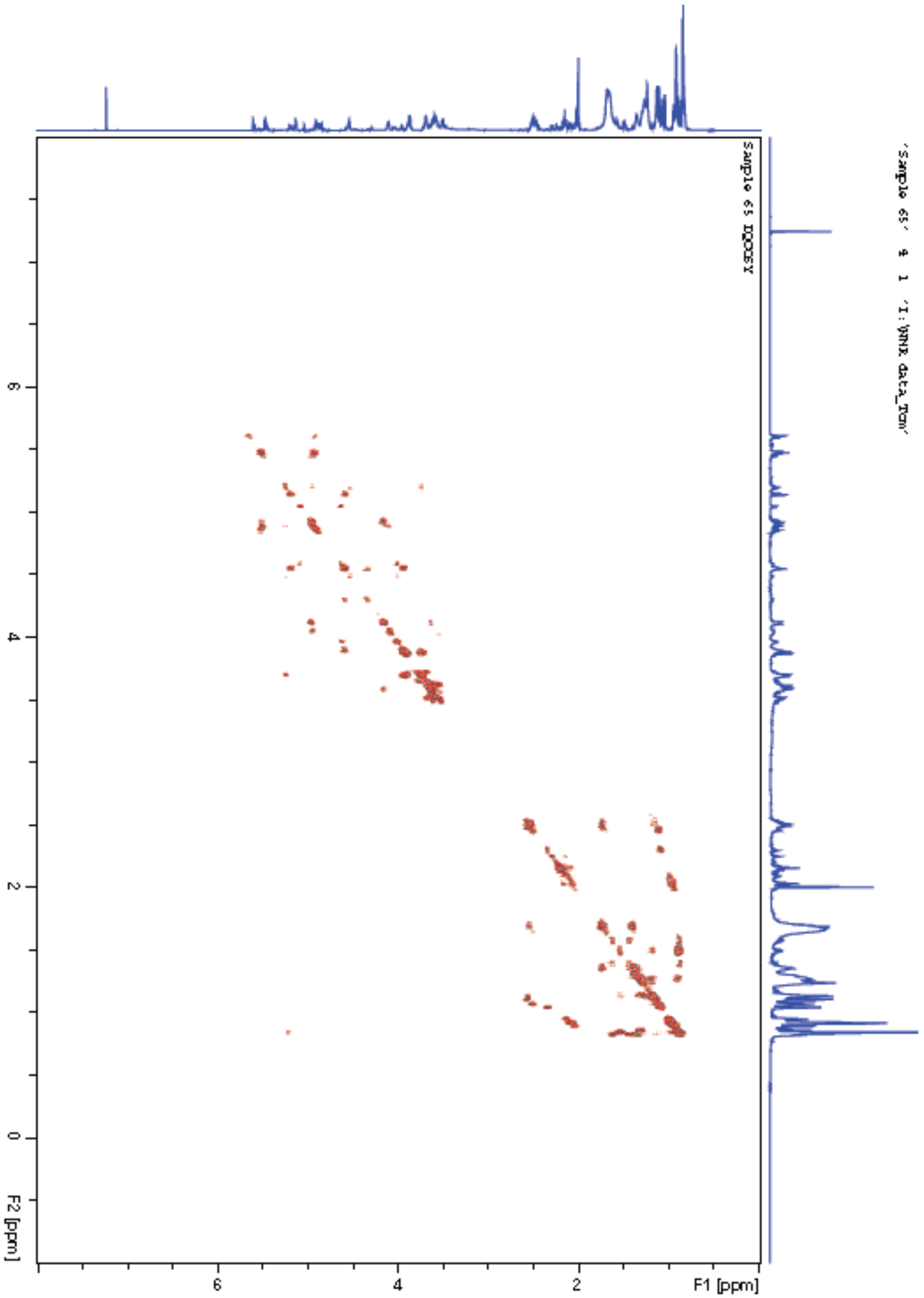
F1 [ppm]
F2 [ppm]

S4:22[3] (2,4,5,11)

Purified from *S. habrochaites* LA1392

HRMS: (ESI) *m/z* calcd for $C_{35}H_{59}O_{17}^{-}$ ([M+HCOO^{-}]): 751.3758, found: 751.3834

Material recovered: 2-3 mg

NMR solvent: $CDCl_3$

InChI Key: CHKUMLUKCBFMMZ-DHWUKAANSA-N

Carbon # (group)	^{1}H (ppm)	^{13}C (ppm)
1(CH)	5.60 (d, *J* = 3.7 Hz)	89.5 ($^{1}J_{CH}$ = 178.0 Hz)
2(CH)	4.91(dd, *J* = 10.4, 3.7 Hz)	70.4
2-O-		
-1(CO)		170.0
-2(CH_3)	2.03 (s)	20.6
3(CH)	5.48 (t, *J* = 10.0 Hz)	68.8
3-O-		
-1(CO)		176.0
-2(CH)	2.48 (m)	33.9
-3(CH_3) x 2	1.09 (d, *J* = 5.8 Hz), 1.09 (d, *J* = 5.8 Hz)	19.2
4(CH)	4.95 (t, *J* = 10.0 Hz)	68.2
4-O		
-1(CO)		172.2
-2(CH_2)	2.16 (dd, *J* = 15.0, 7.1 Hz), 2.18 (dd, *J* = 15.0, 7.1 Hz) [a]	42.9
-3(CH)	2.05 (m)	25.4
-4(CH_3) x 2	0.93 (d, *J* = 2.3 Hz), 0.94 (d, *J* = 2.3 Hz)	22.3, 22.6
5(CH)	4.13 (m)	71.1
6(CH_2)	3.62 (m)	61.6

1' (CH_2)	3.52 (d, *J* = 12.4 Hz), 3.62 (m)	64.5
2' (C)		104.0
3' (CH)	5.16 (d, *J* = 8.0 Hz)	80.0
3'-O		
-1(CO)		175.1
-2(CH_2)	2.52 (m)	34.0
-3(CH_2)	1.71 (quin, *J* = 7.7 Hz)	24.8
-4-5(CH_2)	1.38 (m)	29.2, 29.5
-6(CH_2)	1.26[b], 1.31[b]	27.0
-7(CH_2)	1.09[b], 1.29[b]	36.6
-8(CH)	1.30[b]	34.4
-8'(CH_3)	0.84 (d, *J* = 6.6 Hz)	19.2
-9(CH_2)	1.12 (m), 1.31 (m)	29.6
-10(CH_3)	0.86 (m)	11.4
4' (CH)	4.60 (t, *J* = 8.2 Hz)	71.3
5' (CH)	3.92 (m)	82.4
6' (CH_2)	3.71 (dd, *J* = 13.1, 2.0 Hz), 3.90 (dd, *J* = 13.1, 2.4 Hz)	59.6

[a]Higher order multiplet derived from the constants using gNMR

[b]Determined by COSY and HSQC.

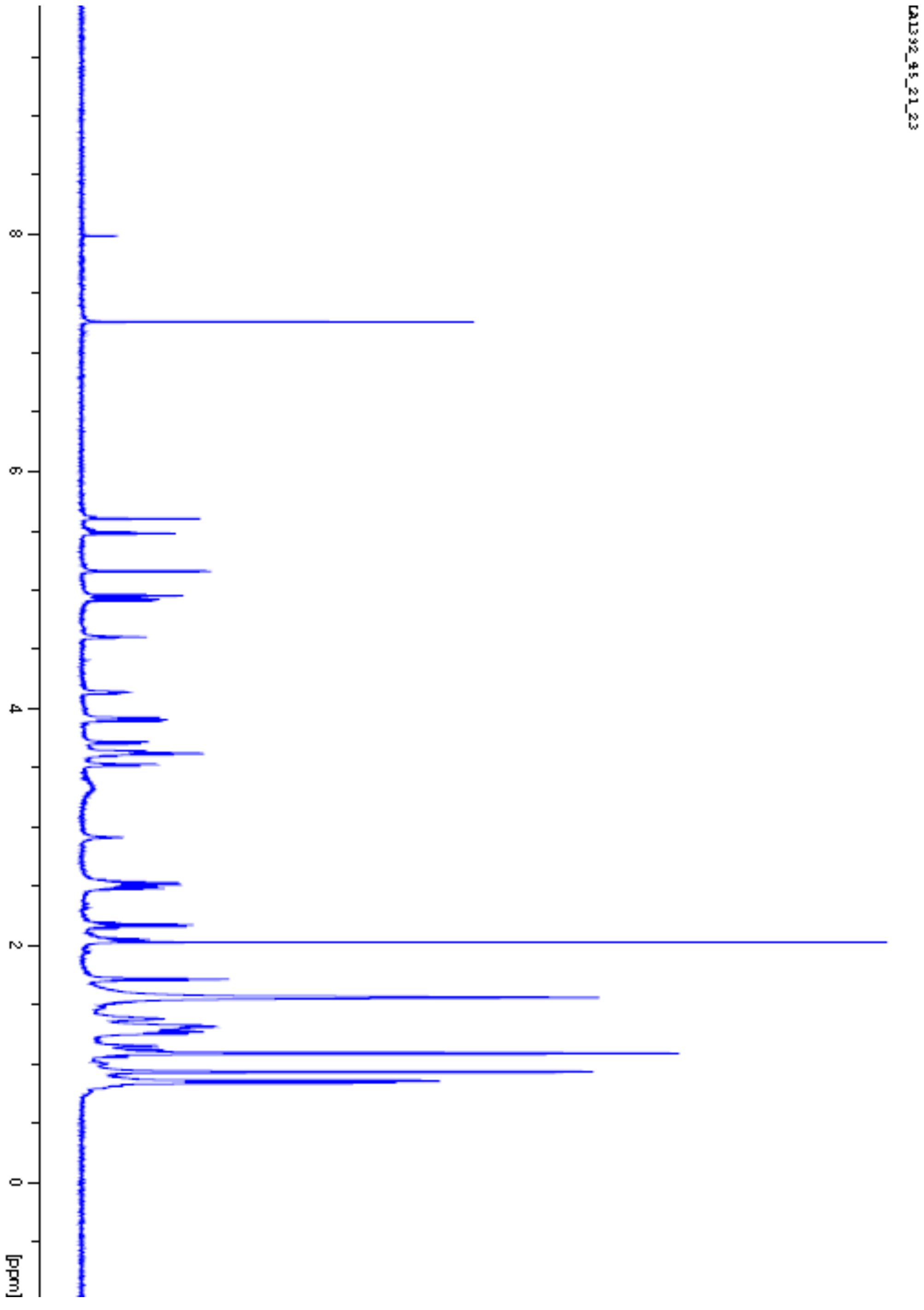
8
6
4
2
0
[ppm]

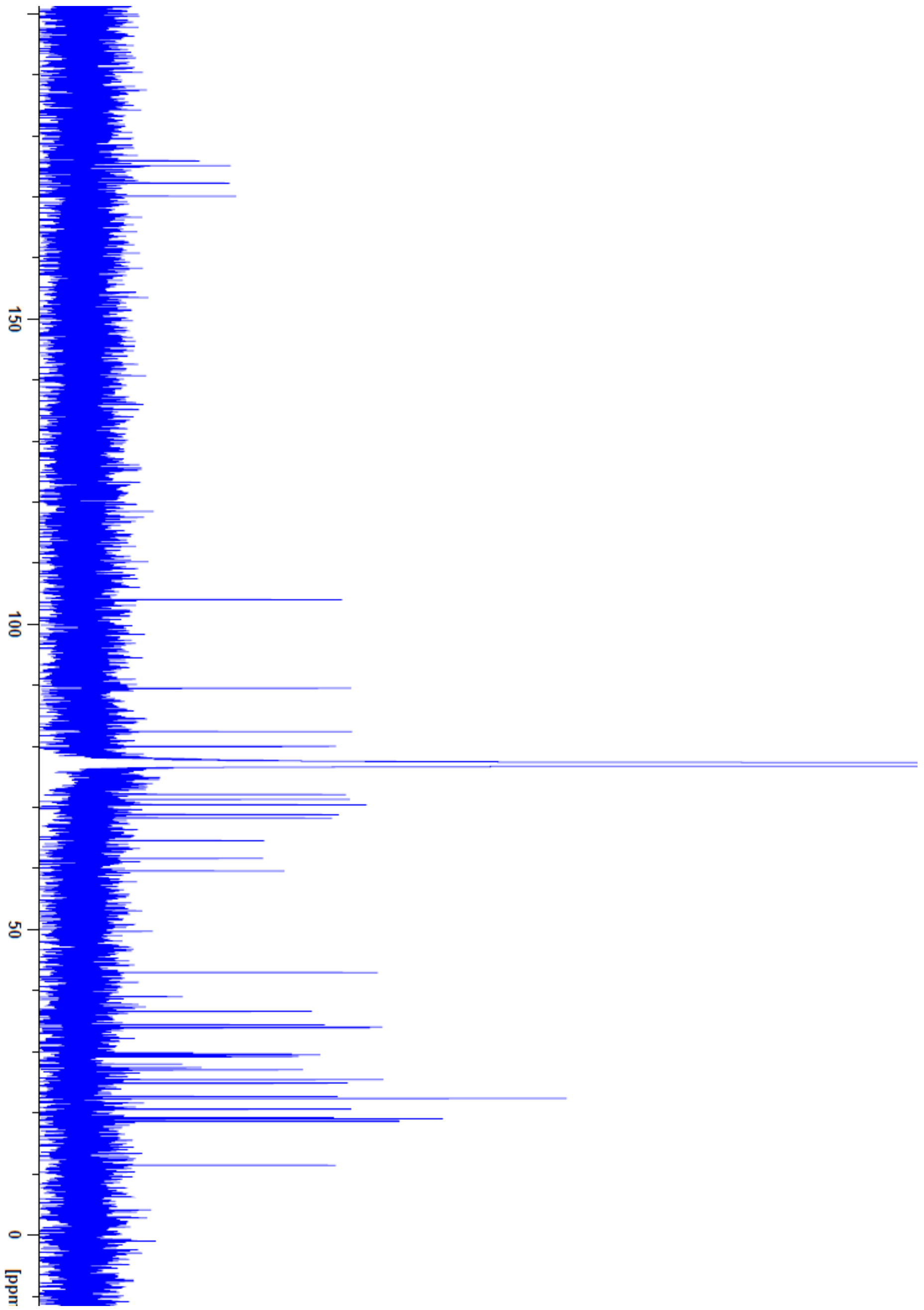
150
100
50
0

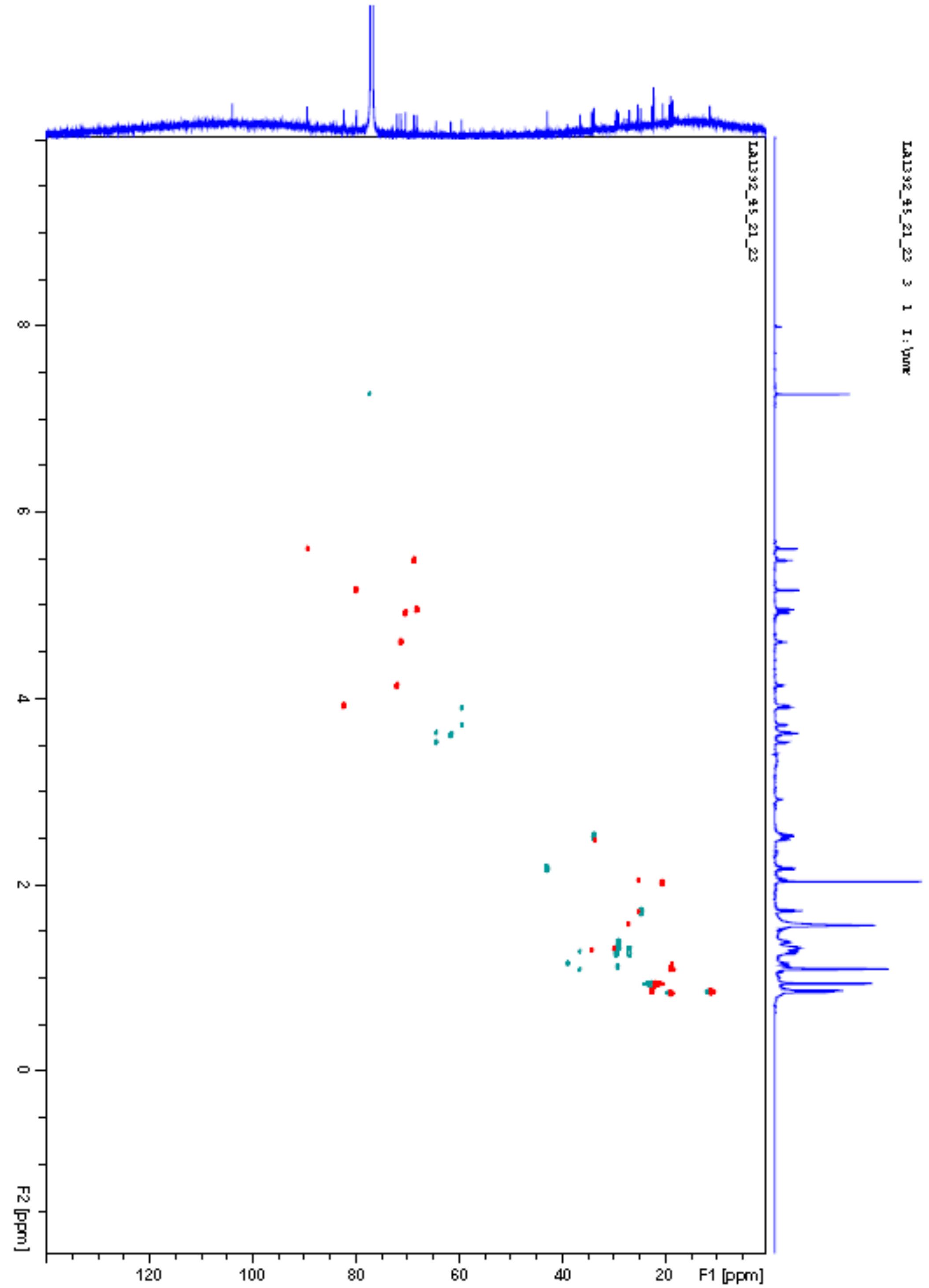
F1 [ppm]
F2 [ppm]

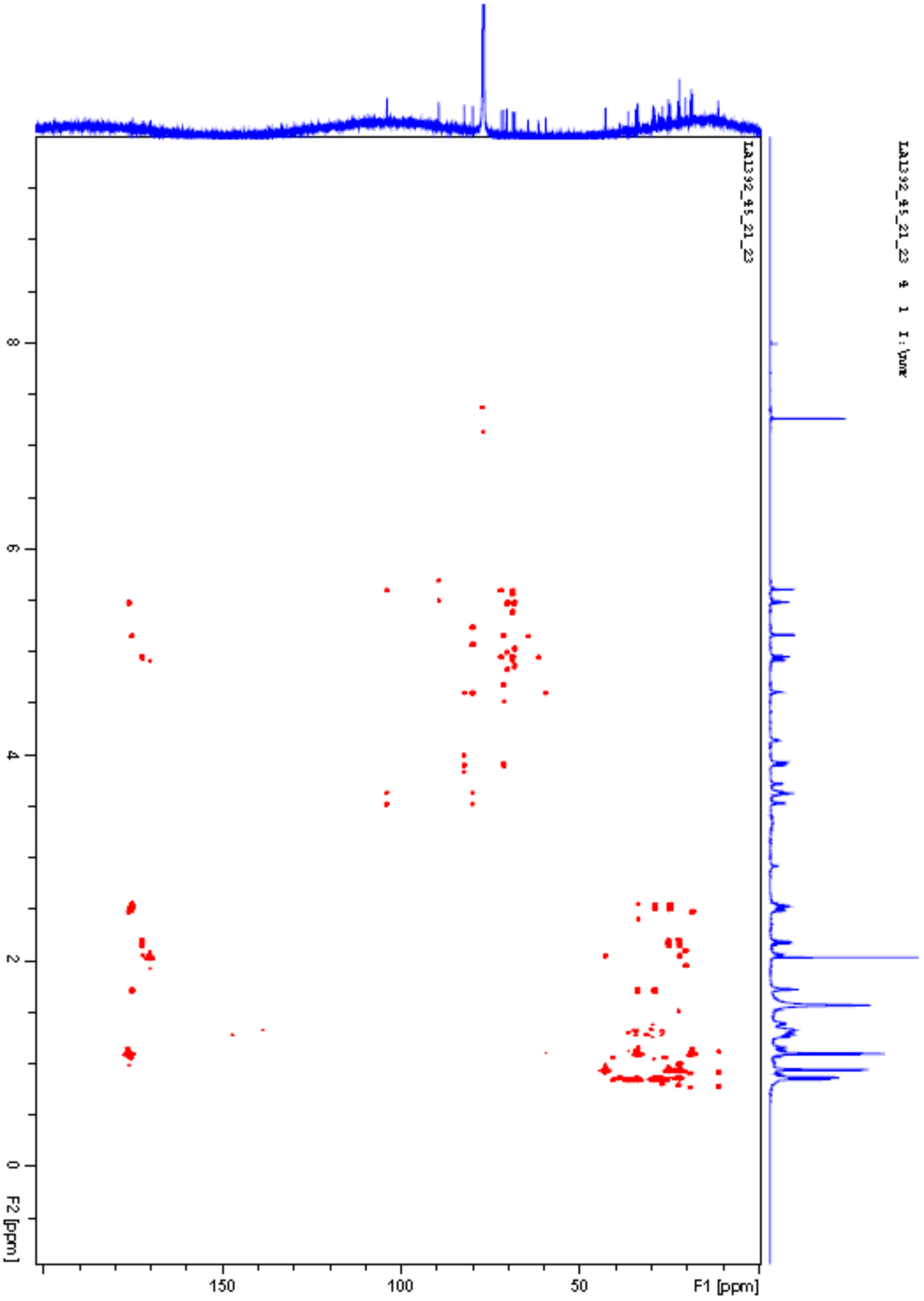

F1 [ppm]
150
100
50
F2 [ppm]
8
6
4
2
0

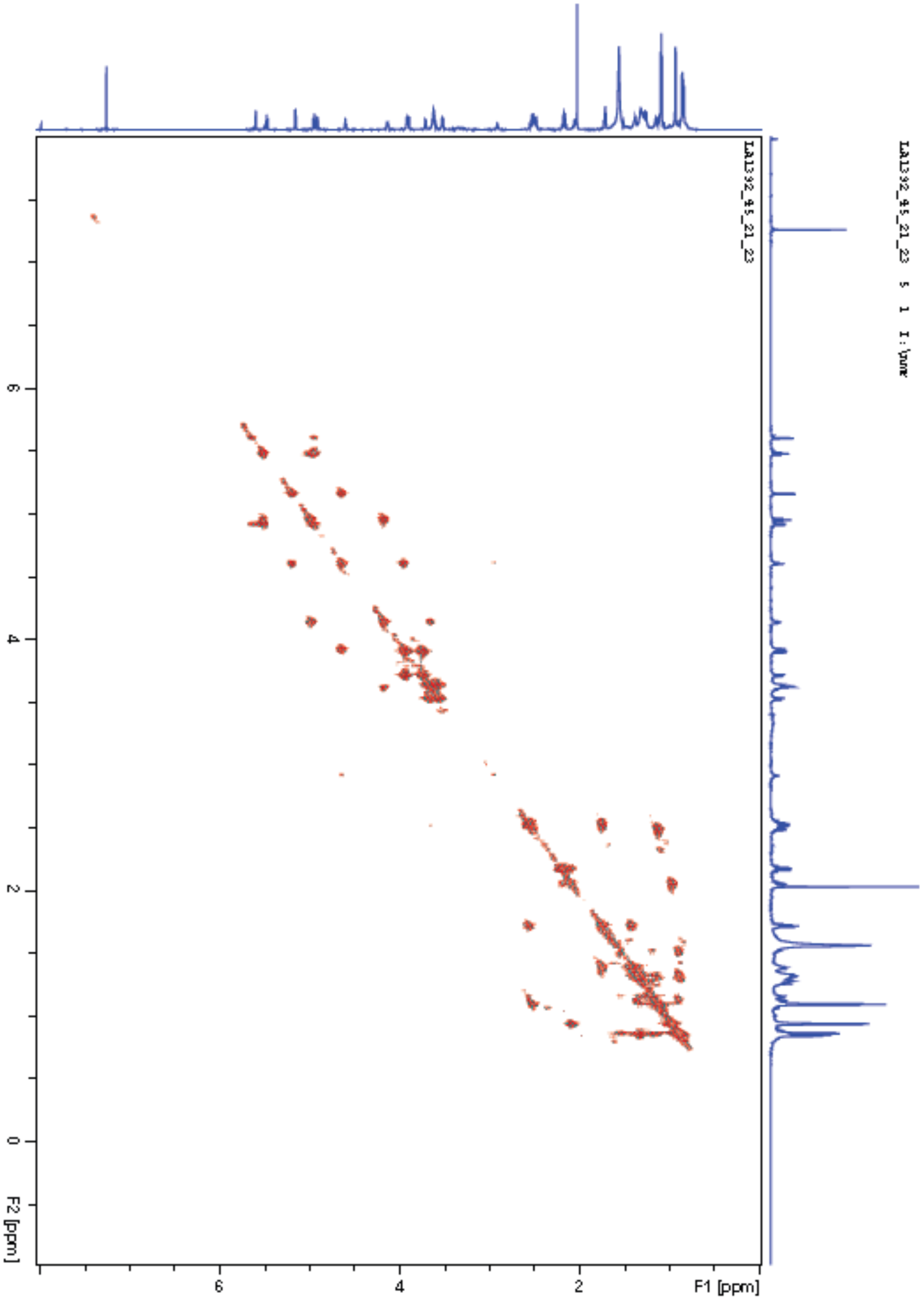
LA1392_45_21_23
LA1392_45_21_23 5 1 I:\nmr
F1 [ppm]
F2 [ppm]

**S4:22[6]** (2,4,4,12)<br><br>**Purified from *S. habrochaites* LA1777**<br><br>**HRMS:** (ESI) *m/z* calcd for $C_{35}H_{59}O_{17}^-$ ([M+HCOO$^-$]): 751.3758, found: 751.3832<br><br>**Material recovered:** 0.5-1 mg<br><br>**NMR solvent**: CD_3OD<br><br>**InChI Key**: MQFRJLURXZHVOT-JUAKHFSCSA-N		
Carbon # (group)	^{1}H (ppm)	^{13}C (ppm)
1(CH)	5.64 (d, *J* = 3.6 Hz)	90.6 ($^1J_{CH}$ = 177.0 Hz)
2(CH)	4.88[a]	72.0
2-O-		
-1(CO)		171.7
-2(CH_3)	2.01 (s)	20.8
3(CH)	5.45 (t, *J* = 9.9 Hz)	71.3
3-O-		
-1(CO)		177.5
-2(CH)	2.52 (m)	35.3
-3(CH_3) x 2	1.12 (d, *J* = 7.0 Hz), 1.14 (d, *J* = 7.0 Hz)	b
4(CH)	5.11 (t, *J* = 9.9 Hz)	69.7
4-O		
-1(CO)		177.0
-3(CH)	2.52 (m)	35.3
-4(CH_3) x 2	1.08 (d, *J* = 7.0 Hz), 1.08 (d, *J* = 7.0 Hz)	See below[b]
5(CH)	4.14 (m)	72.0
6(CH_2)	3.55 (dd, *J* = 12.3, 4.6 Hz), 3.66 (dd, *J* = 12.3, 2.4 Hz)	61.6

1' (CH_2)	3.44 (d, *J* = 11.8 Hz), 3.58 (d, *J* = 11.8 Hz)	64.8
2' (C)		105.1
3' (CH)	5.39 (d, *J* = 8.2 Hz)	78.8
3'-O		
-1(CO)		175.0
-2(CH_2)	2.48 (m)	35.1
-3(CH_2)	1.72 (m)	26.2
-4(CH_2)	1.40 (m)	30.5
-5(CH_2)	1.36 (m)	30.5
-6-9(CH_2-CH_2-CH_2-CH_2)	1.26-1.34 (br. m)	30.6, 30.9, 30.9, 30.9
-10(CH_2)	1.26-1.34 (br. m)	33.2
-11(CH_2)	1.31[a]	23.9
-12(CH_3)	0.90 (t, *J* = 7.2 Hz)	14.5
4' (CH)	4.29 (t, *J* = 8.4 Hz)	73.6
5' (CH)	3.88 (m)	84.3
6' (CH_2)	3.77 (m)	63.2

[a]Determined by COSY

[b] ^{13}C could not be unequivocally assigned among resonances at 19.2, 19.4, 19.4 and 19.5 ppm.

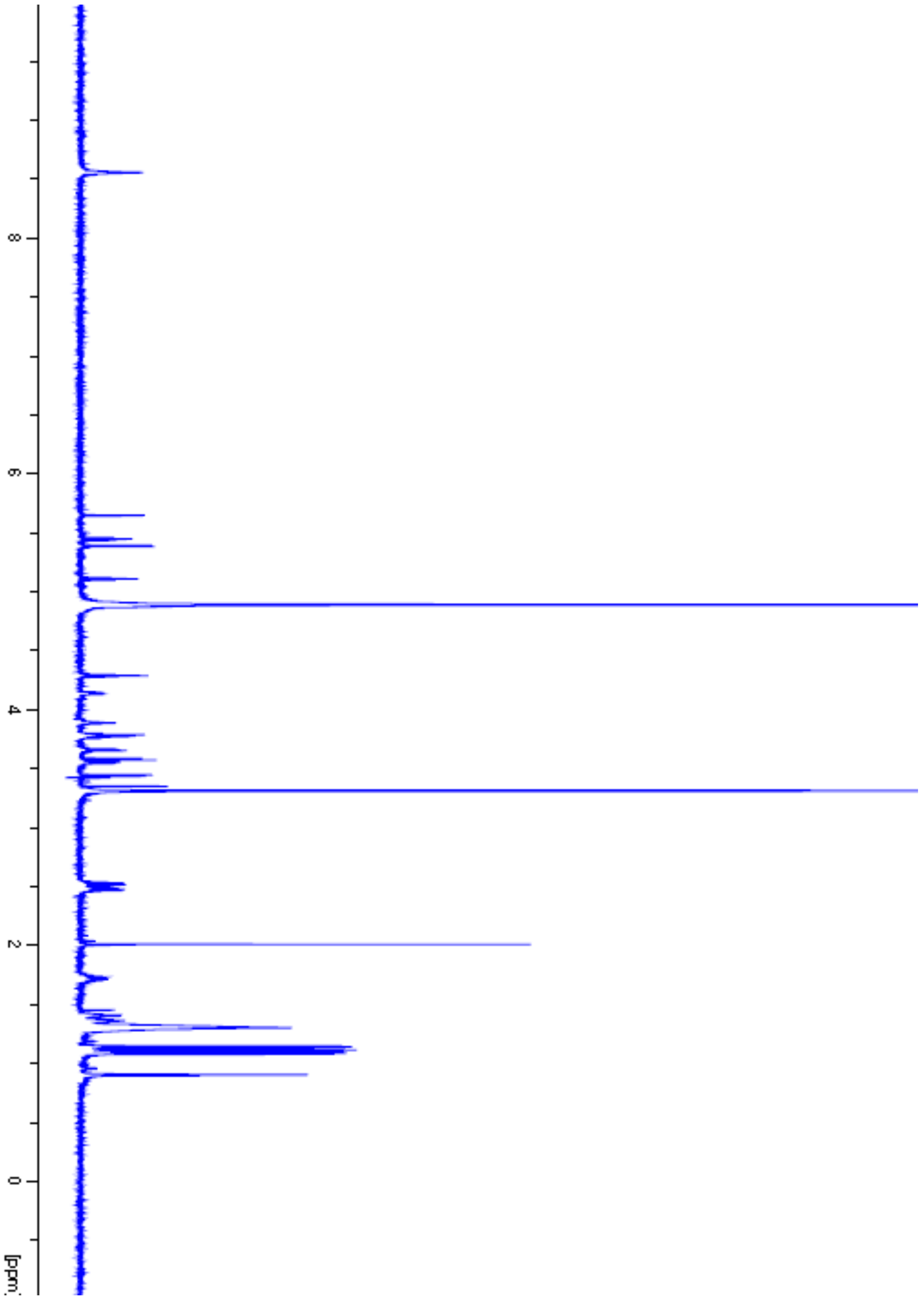
8
6
4
2
0
[ppm]

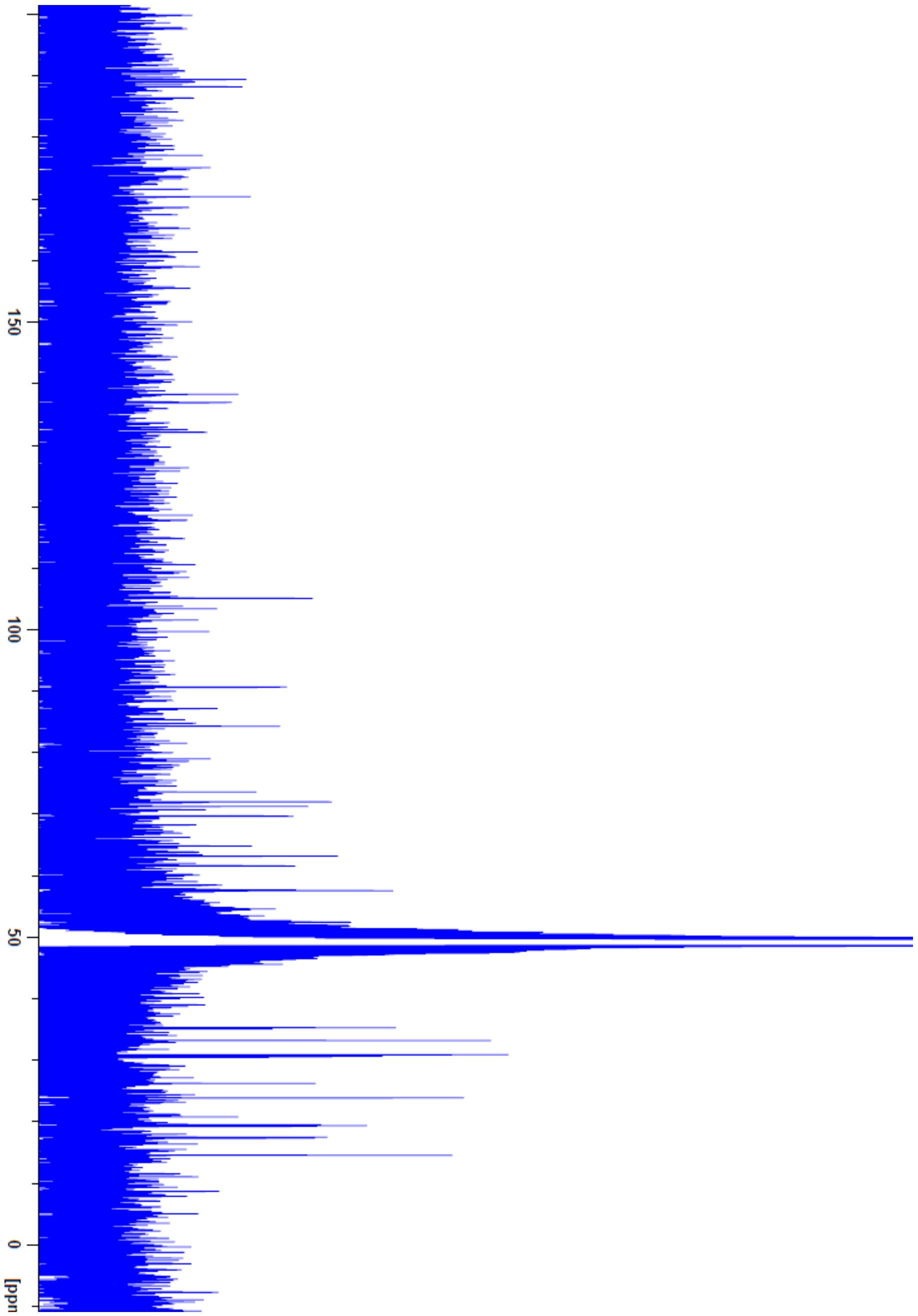
150
100
50
0

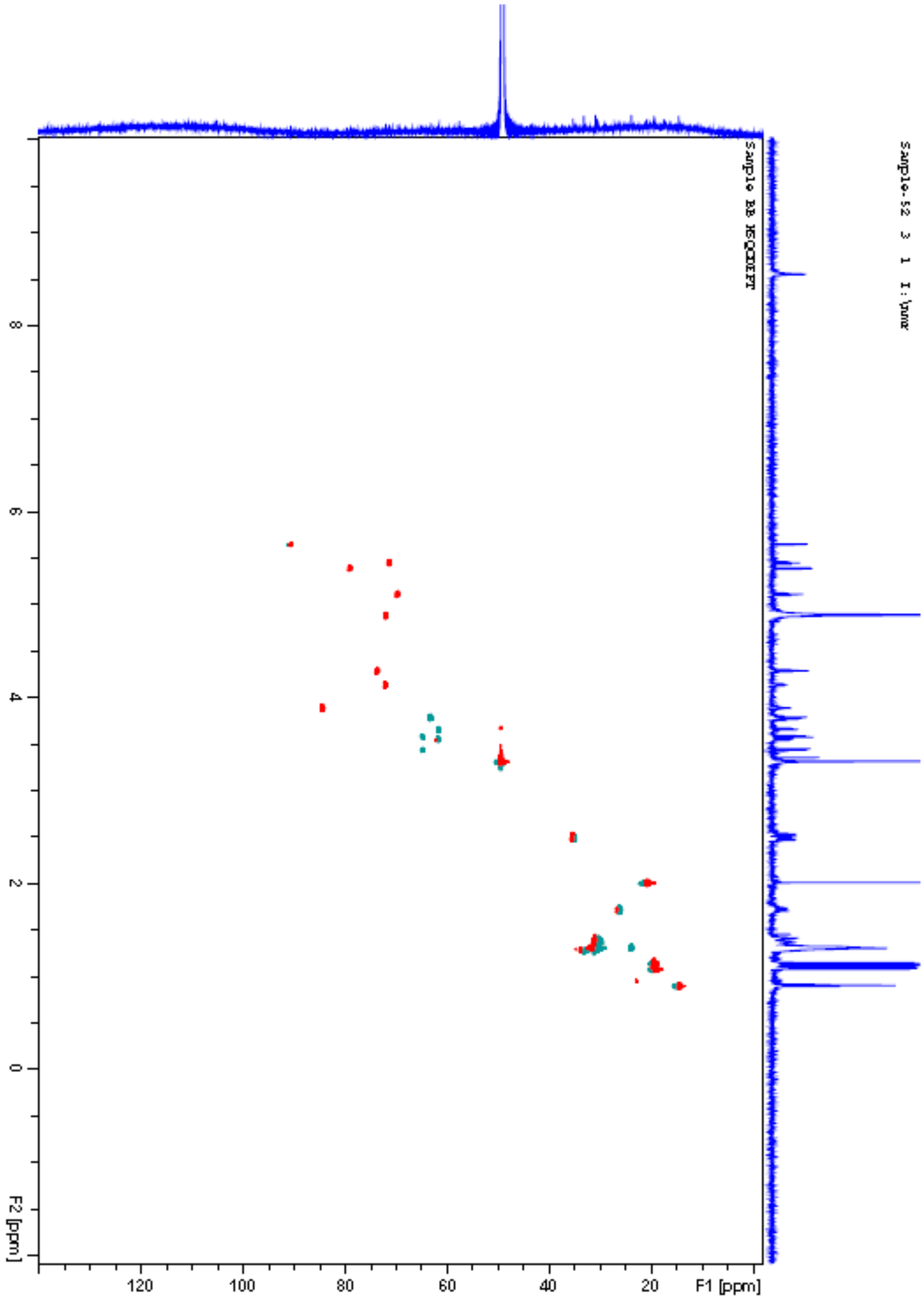

F1 [ppm]
F2 [ppm]

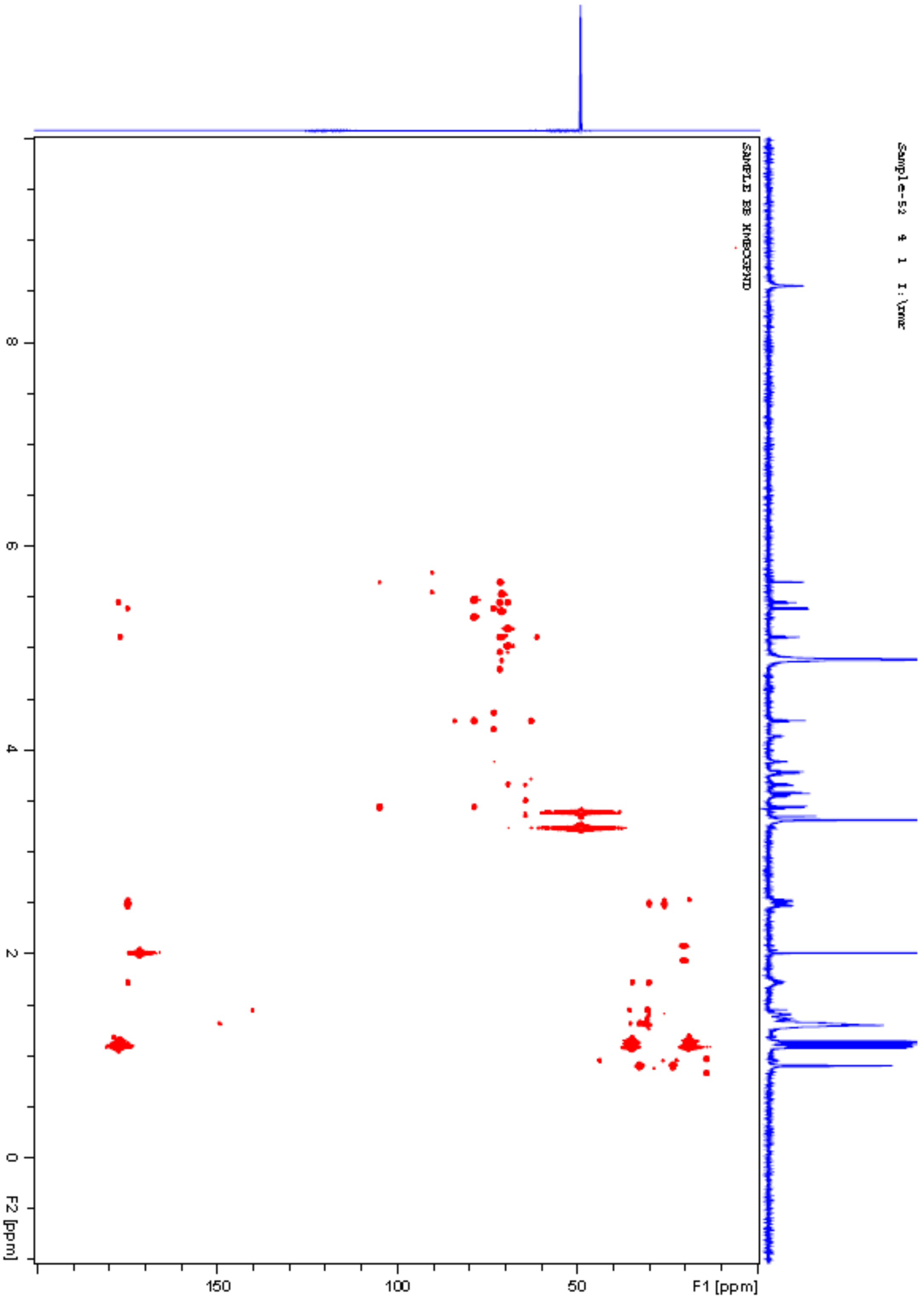
Sample-52 4 1 I:\nmr
SAMPLE EE HMBCGPND
F1 [ppm]
F2 [ppm]
150
100
50
8
6
4
2
0

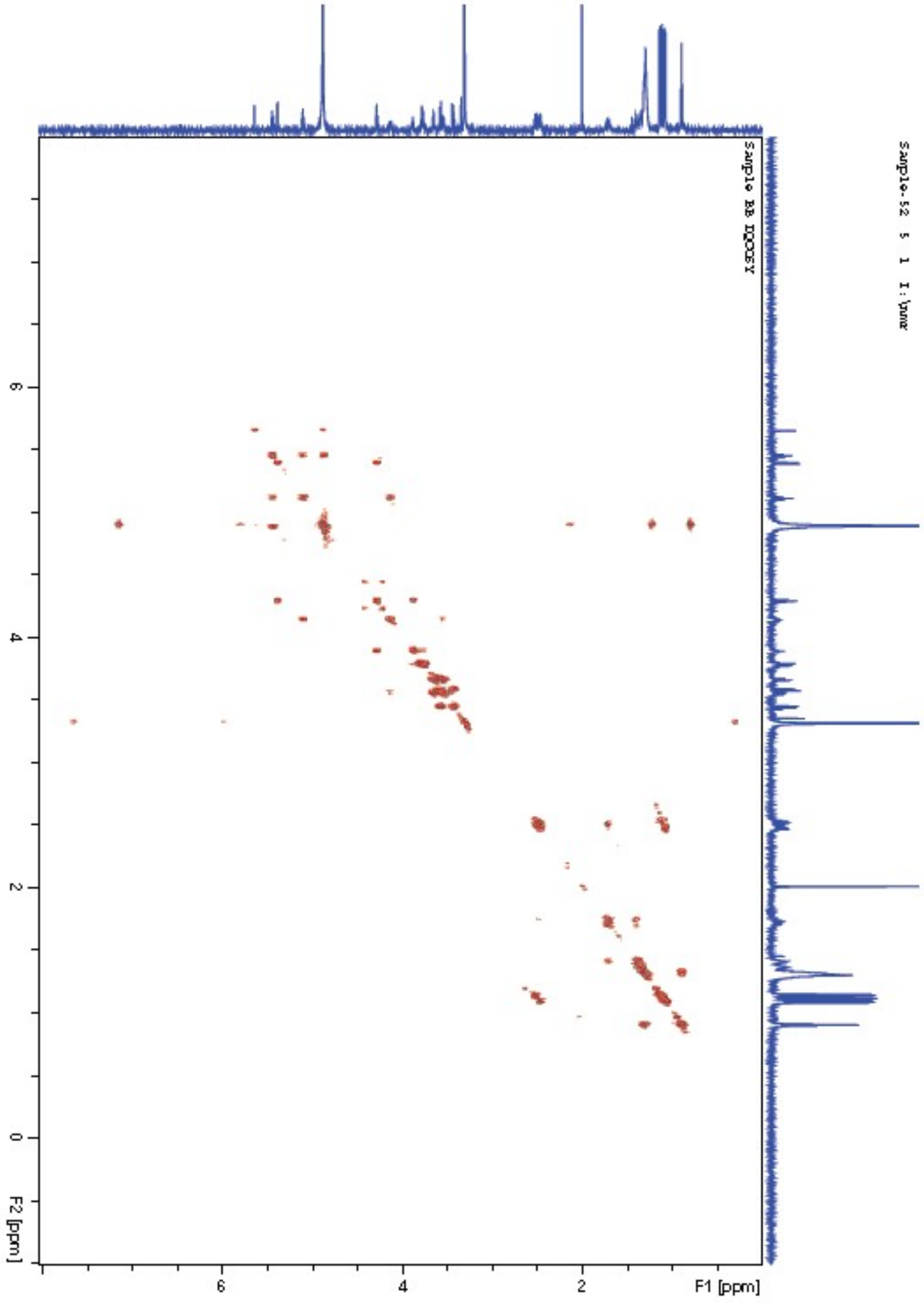
F2 [ppm]
F1 [ppm]
Sample 52 5 1 I:\nmr

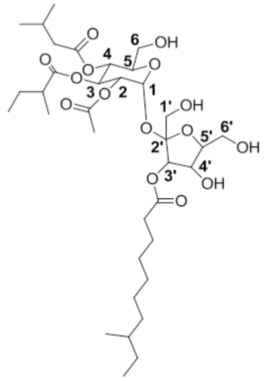

S4:23[3] (2,5,5,11)

Purified from *S. habrochaites* LA1392

HRMS: (ESI) *m/z* calcd for $C_{36}H_{61}O_{17}^{-}$ ([M+HCOO^{-}]): 765.3914, found: 765.4035

Material recovered: 2-3 mg

NMR solvent: $CDCl_3$

InChI Key: RNROCLFHSOXEIV-IZILDJFTSA-N

Carbon # (group)	^{1}H (ppm)	^{13}C (ppm)
1(CH)	5.62 (d, J = 3.7 Hz)	89.3 ($^{1}J_{CH}$ = 177.4 Hz)
2(CH)	4.89 (dd, J = 10.4, 3.7 Hz)	70.4
2-O-		
-1(CO)		170.4
-2(CH_3)	2.02 (s)	20.5
3(CH)	5.50 (t, J = 10.0 Hz)	68.6
3-O-		
-1(CO)		175.6
-2(CH)	2.32 (m)	40.7
-2'(CH_3)	1.06 (d, J = 7.0 Hz)	16.3
-3(CH_2)	1.41 (m), 1.60 (m)	26.3
-4(CH_3)	0.85 (m)	b
4(CH)	4.94 (t, J = 9.9 Hz)	68.2
4-O		
-1(CO)		172.4
-2(CH_2)$_2$	2.15 (dd, J = 15.0, 7.1 Hz), 2.18 (dd, J = 15.0, 7.1 Hz) [a]	42.7
-3(CH)	2.05 (m)	25.2
-4(CH_3) x 2	0.94 (d, J = 6.6 Hz), 0.94 (d, J = 6.6 Hz)	22.0

5(CH)	4.13 (m)	71.9
6(CH_2)	3.62 (m)	61.5
1' (CH_2)	3.53 (d, J = 12.4 Hz, 3.64 (d, J = 12.4 Hz)	64.3
2' (C)		104.0
3' (CH)	5.15 (d, J = 8.0 Hz)	79.9
3'-O		
-1(CO)		175.3
-2(CH_2)	2.53 (m)	34.2
-3(CH_2)	1.72 (quin, J = 7.8 Hz)	25.0
-4-5(CH_2-CH_2)	1.40 (m)	29.4, 29.7
-6(CH_2)	1.25[c], 1.31[c]	27.2
-7(CH_2)	1.09[c], 1.29[c]	36.8
-8(CH)	1.31[d]	34.6
-8'(CH_3)	0.85 (m)	19.4
-9(CH_2)	1.11 (m), 1.31 (m)	29.8
-10(CH_3)	0.85 (m)	See below[b]
4' (CH)	4.60 (t, J = 8.3 Hz)	71.2
5' (CH)	3.91 (m)[d]	82.2
6' (CH_2)	3.70 (d, J = 12.1 Hz), 3.90 (m)[c]	59.4

[a]Higher order multiplet derived from the constants using gNMR

[b] ^{13}C could not be unequivocally assigned among resonances at 11.6 and 11.7 ppm

[c]Determined by COSY and HSQC

[d]Determined by COSY

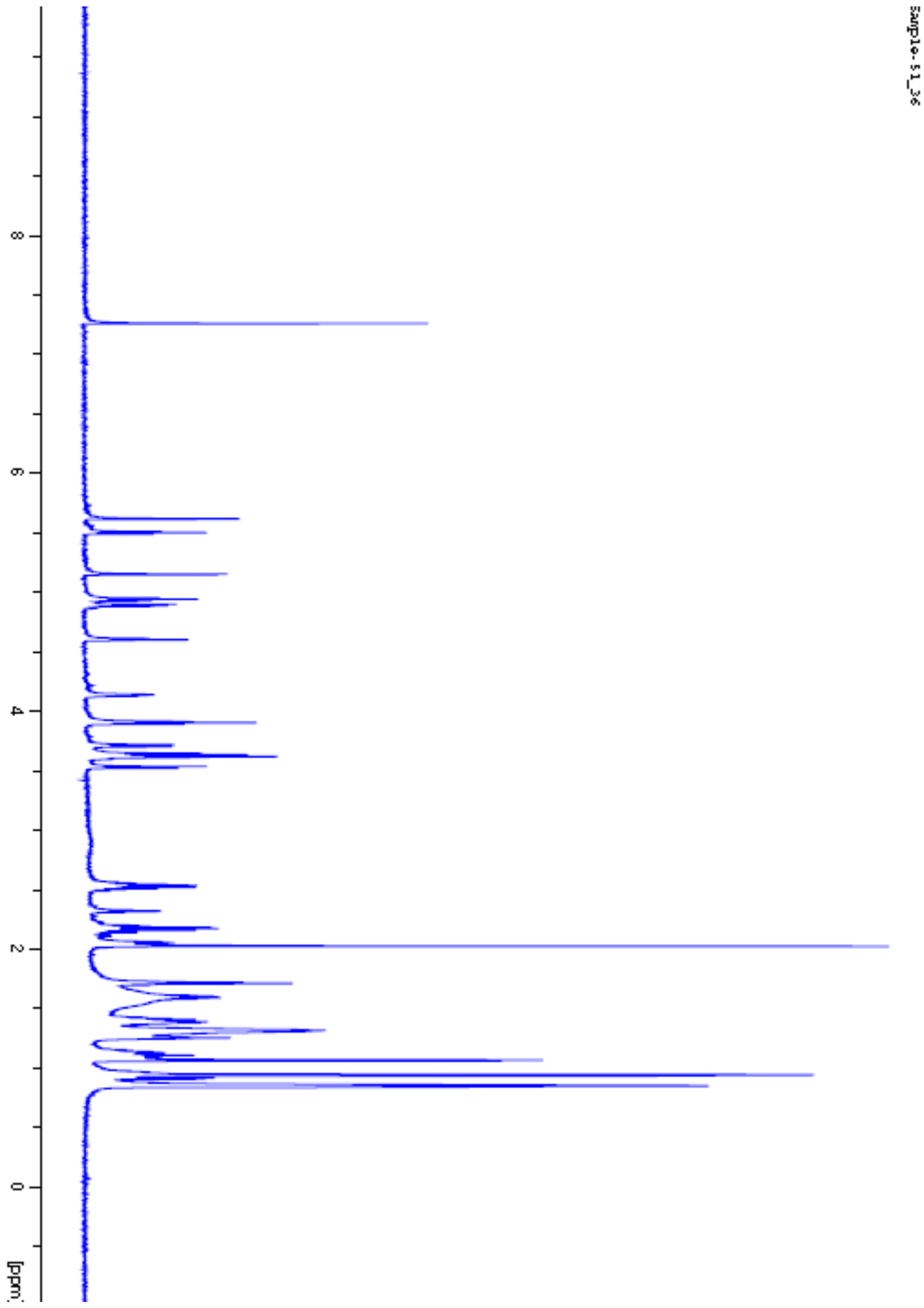
Sample-51_36
8
6
4
2
0
[ppm]

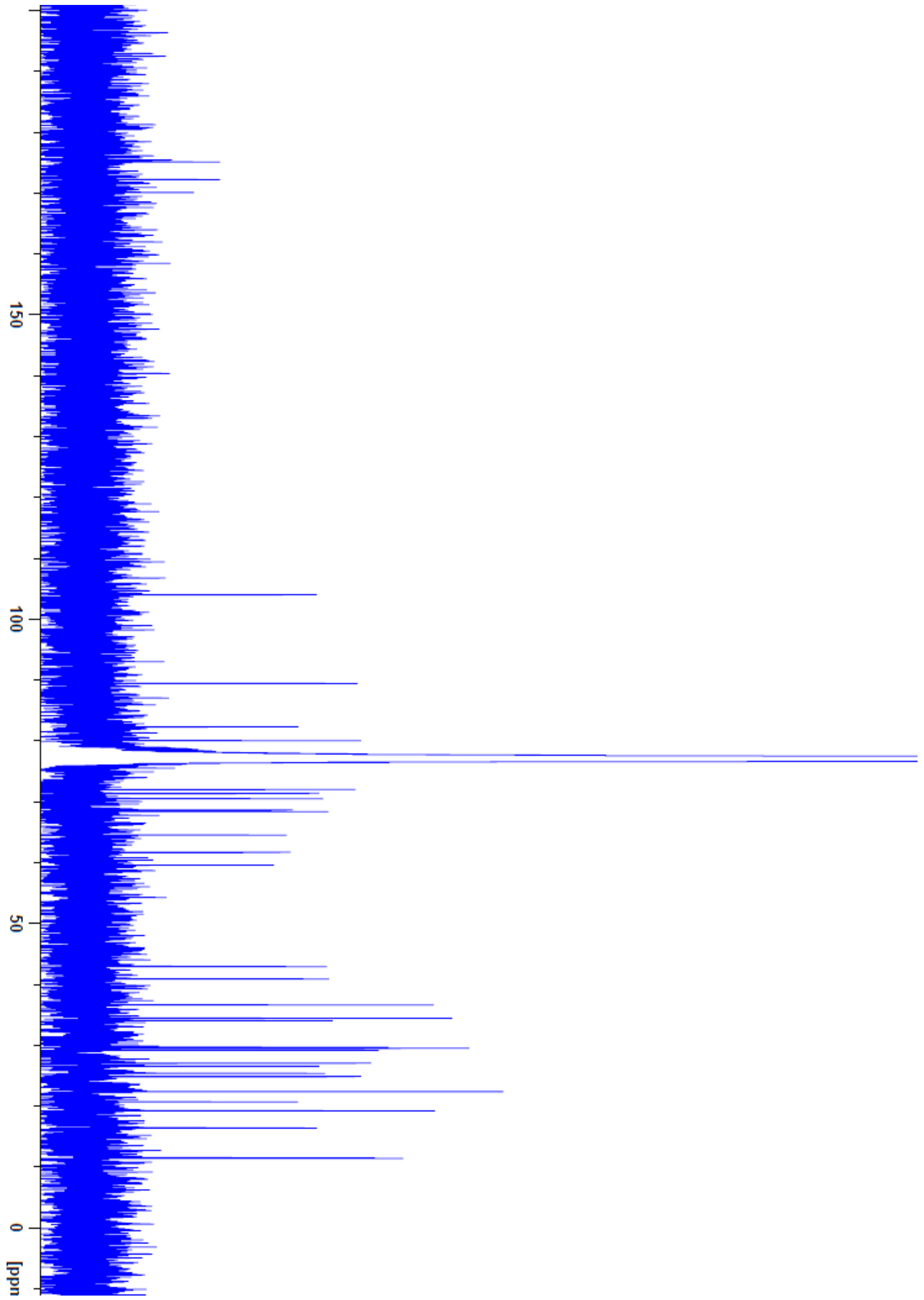
150
100
50
0

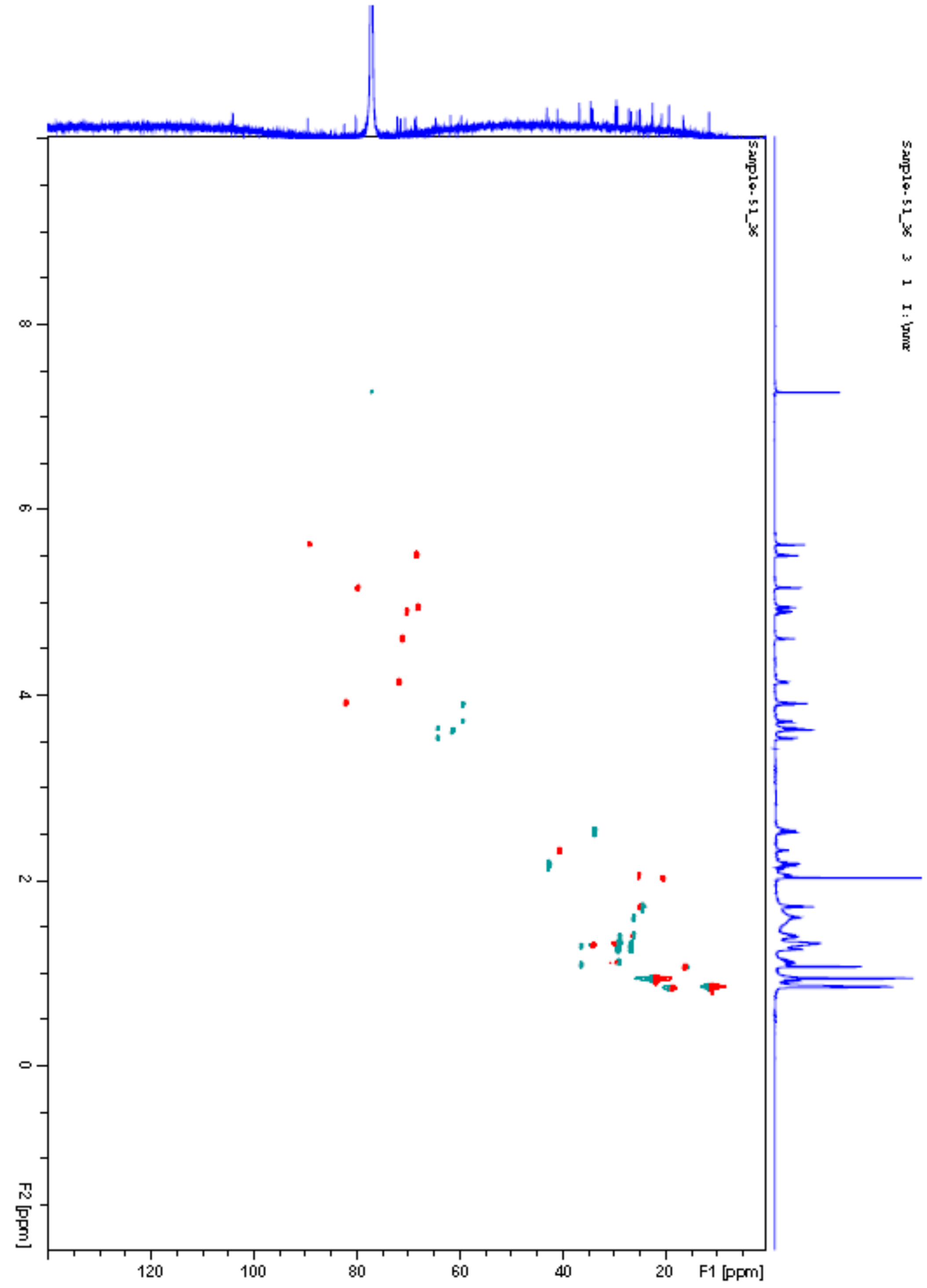
120
100
80
60
40
20
F1 [ppm]

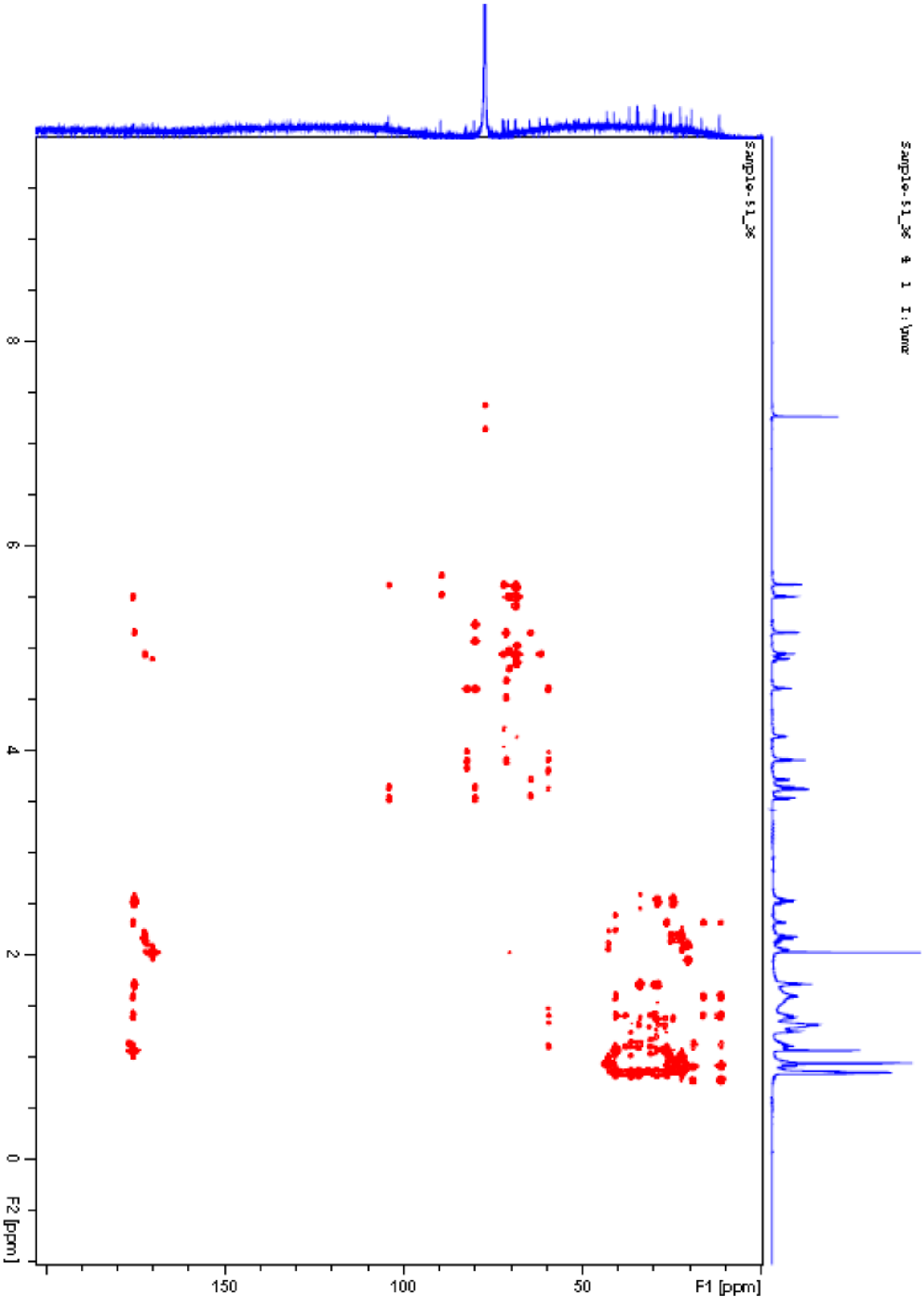

150
100
50
F1 [ppm]
8
6
4
2
0
F2 [ppm]

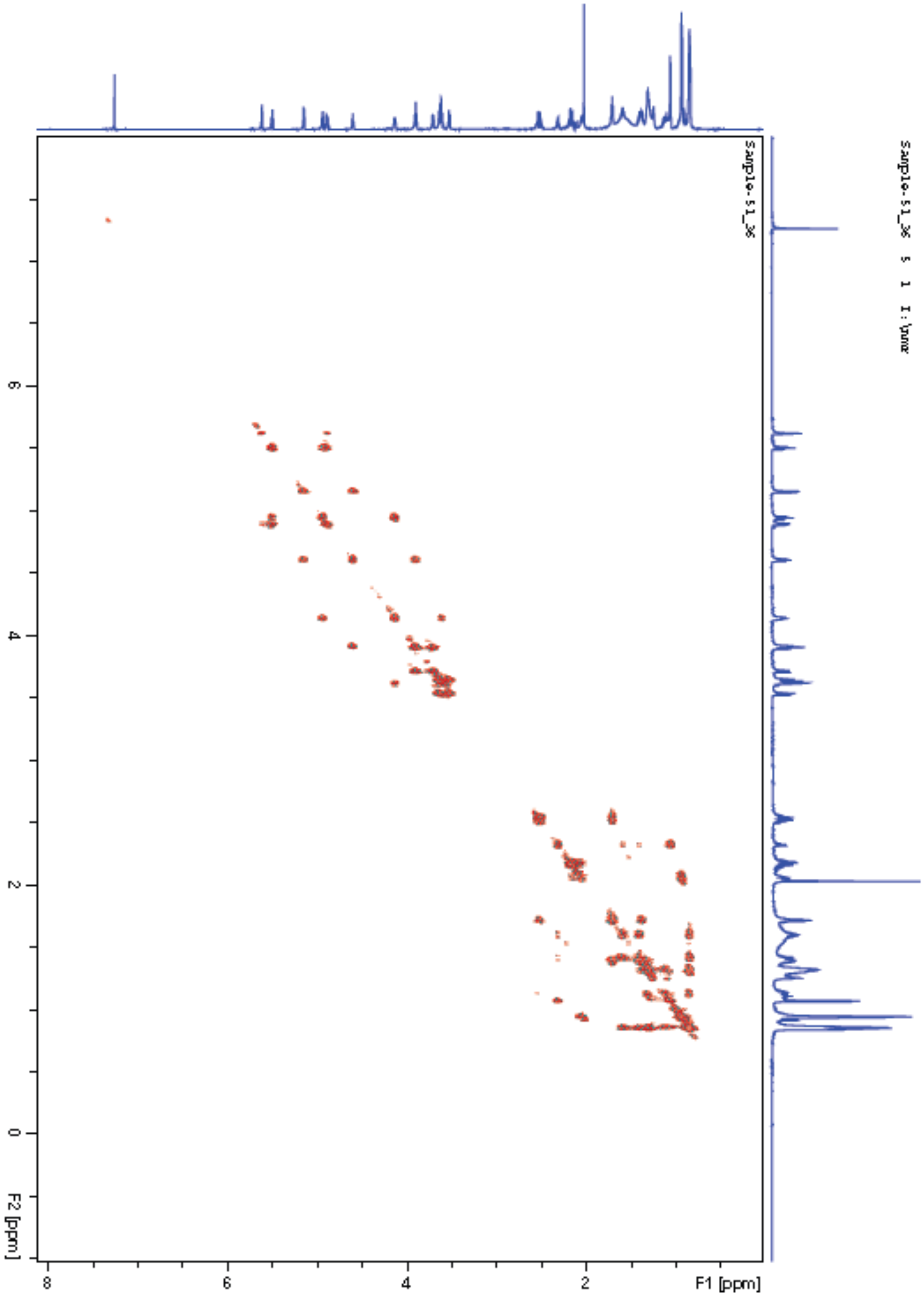
F1 [ppm]
F2 [ppm]

**S4:23[5]** (2,4,5,12)<br><br>**Purified from *S. habrochaites* LA1392**<br><br>**HRMS:** (ESI) *m/z* calcd for $C_{36}H_{61}O_{17}^-$ ([M+HCOO$^-$]): 765.3914, found: 765.4016<br><br>**Material recovered:** 1-2 mg<br><br>**NMR solvent**: $CDCl_3$<br><br>**InChI Key**: PJFIWYSBJRFHSN-KUAWNJLRSA-N		
Carbon # (group)	^{1}H (ppm)	^{13}C (ppm)
1(CH)	5.60 (d, J = 3.8 Hz)	89.5 ($^1J_{CH}$ = 177.8 Hz)
2(CH)	4.91 (dd, J = 10.3, 3.9 Hz)	70.4
2-O-		
-1(CO)		170.0
-2(CH_3)	2.03 (s)	20.6
3(CH)	5.47 (t, J = 10.0 Hz)	68.8
3-O-		
-1(CO)		176.0
-2(CH)	2.48 (m)	33.9
-3(CH_3) x 2	1.09 (d, J = 5.8 Hz), 1.09 (d, J = 5.7 Hz)	18.6
4(CH)	4.95 (t, J = 9.9 Hz)	68.2
4-O		
-1(CO)		172.2
-2(CH_2)$_2$	2.16 (dd, J = 15.0, 7.1 Hz), 2.18 (dd, J = 15.0, 7.1 Hz) [a]	42.9
-3(CH)	2.05 (m)	25.4
-4(CH_3) x 2	0.93 (d, J = 6.7 Hz), 0.93 (d, J = 6.7 Hz)	21.6
5(CH)	4.14 (m)	72.1
6(CH_2)	3.61 (m)	61.6

1' (CH_2)	3.52 (d, *J* = 12.4 Hz, 3.63 (m)[b]	64.5
2' (C)		104.0
3' (CH)	5.16 (d, *J* = 8.1 Hz)	79.9
3'-O		
-1(CO)		175.0
-2(CH_2)	2.52 (m)	34.0
-3(CH_2)	1.71 (quin, *J* = 7.5 Hz)	24.5
-4(CH_2)	1.38 (m)	29.1, 29.3
-5(CH_2)	1.33 (m)	
-6(CH_2)	1.29 (m)	27.4, 29.6, 29.9
-7(CH_2)	1.23-1.27 (br. m)	
-8(CH_2)	1.23-1.27 (br. m)	
-9(CH_2)	1.15 (m)	39.2
-10(CH)	1.51[c]	28.0
-11(CH_3) x 2	0.86 (d, *J* = 6.6 Hz)	22.6
4' (CH)	4.60 (t, *J* = 8.3 Hz)	71.2
5' (CH)	3.92 (ddd, *J* = 8.4, 2.4, 2.4 Hz)	82.4
6' (CH_2)	3.71 (dd, *J* = 13.0, 2.0 Hz), 3.90 (dd, *J* = 13.4, 2.4 Hz)	59.6

[a]Higher order multiplet derived from the constants using gNMR

[b]Determined by COSY and HSQC

[c]Determined by COSY

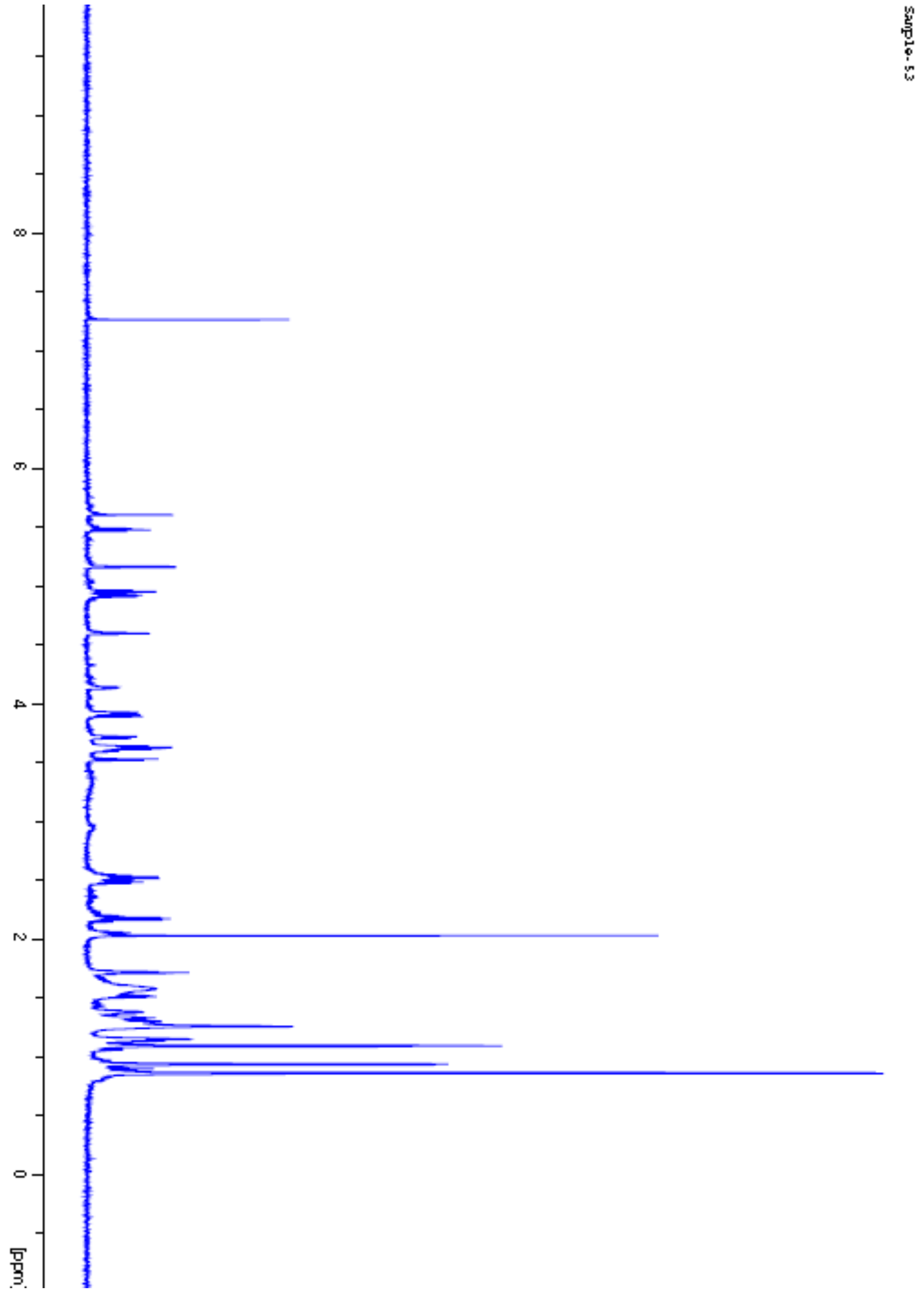
8
6
4
2
0
[ppm]

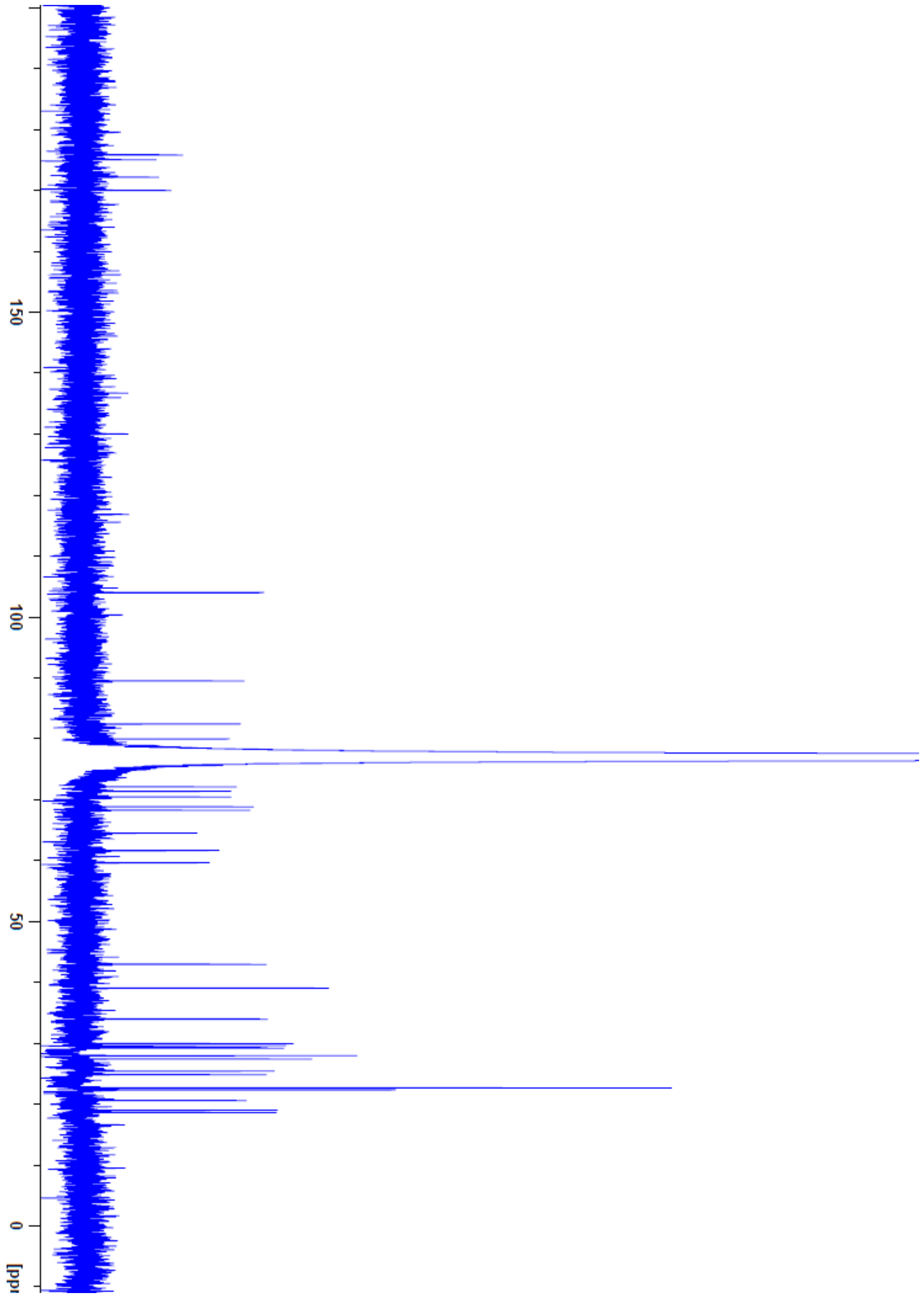
150
100
50
0

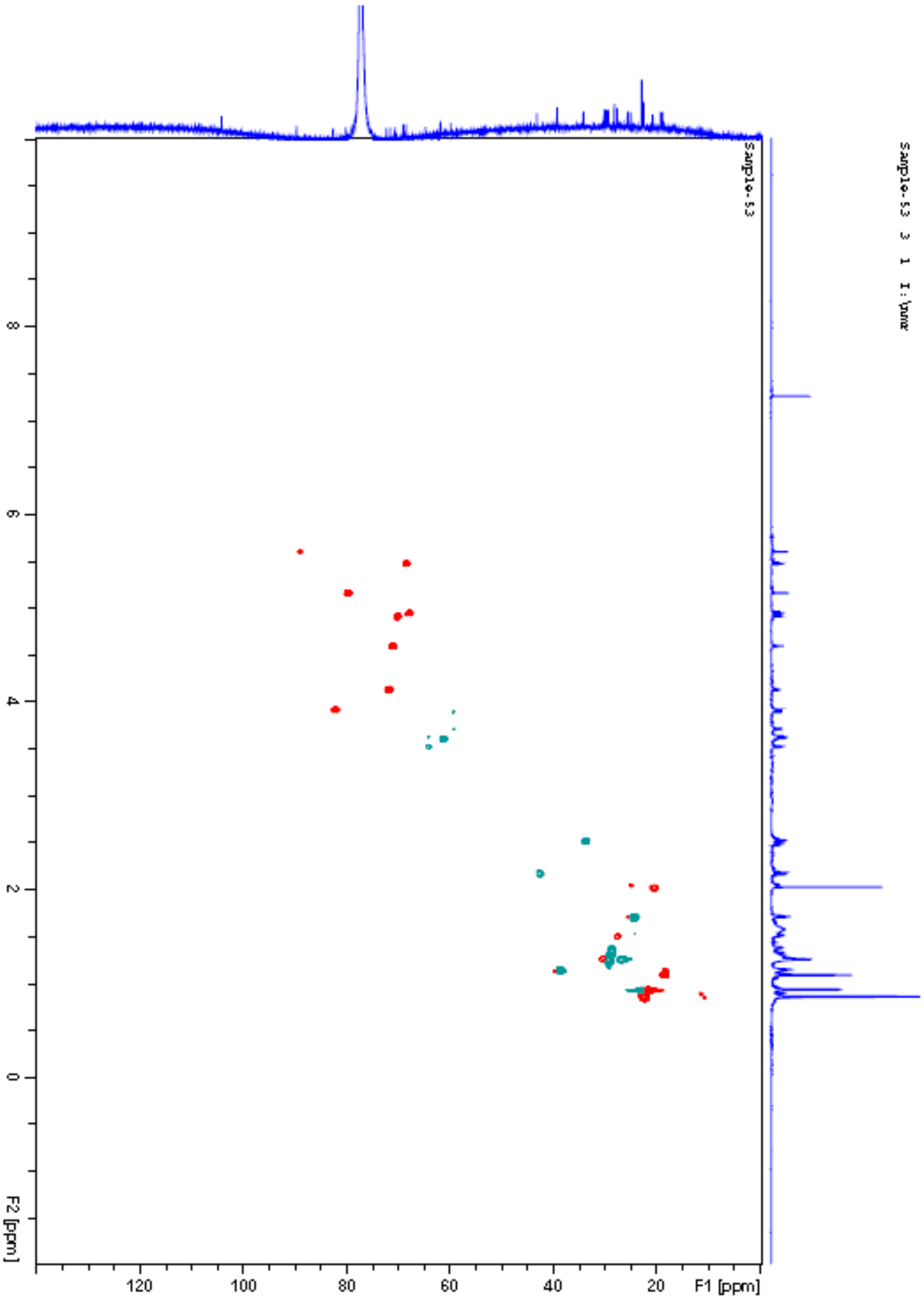
F1 [ppm]
F2 [ppm]
Sample-53
Sample-53 3 1 I:\nmr
120
100
80
60
40
20
8
6
4
2
0

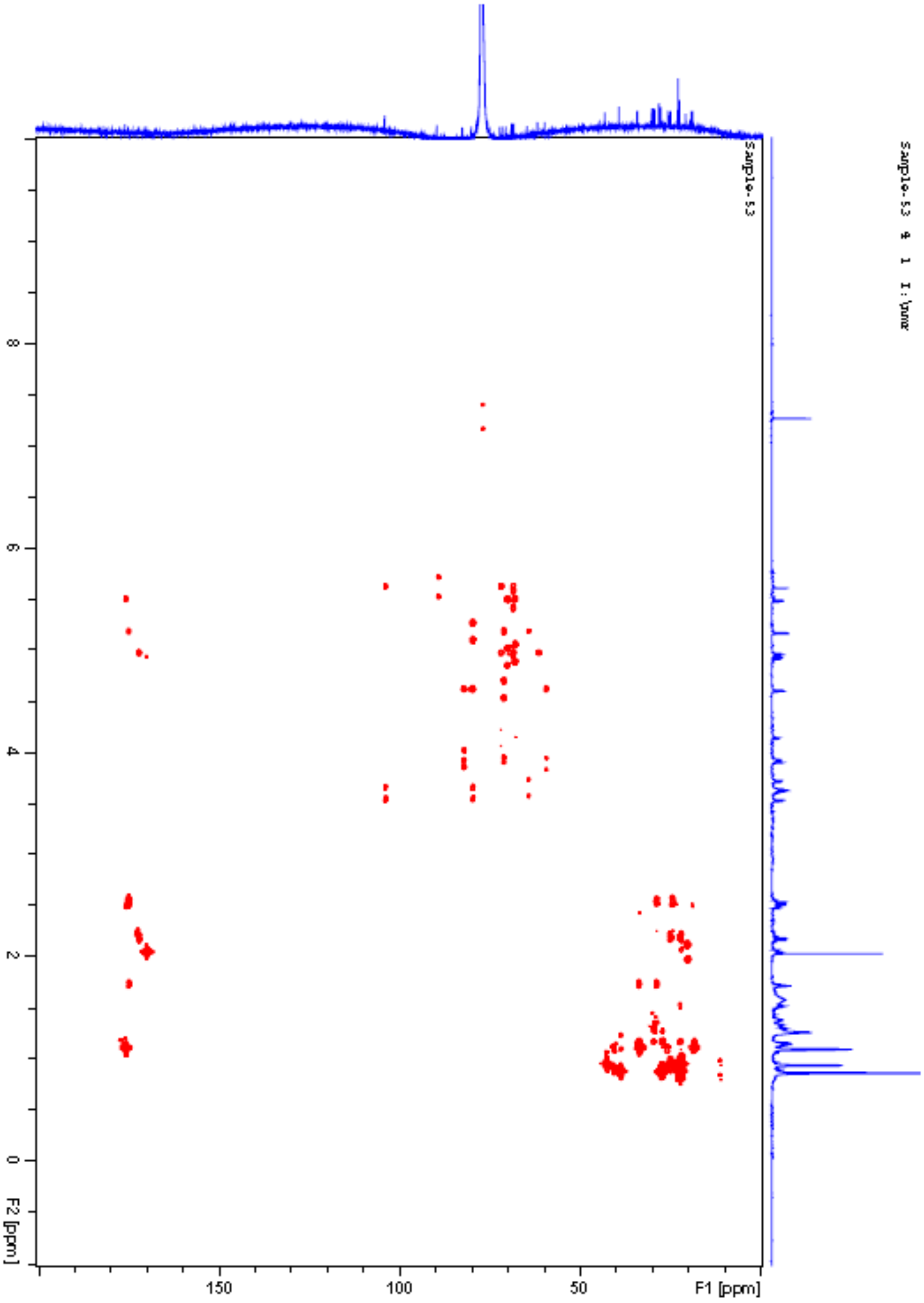

Sample-53
150
100
50
F1 [ppm]
8
6
4
2
0
F2 [ppm]

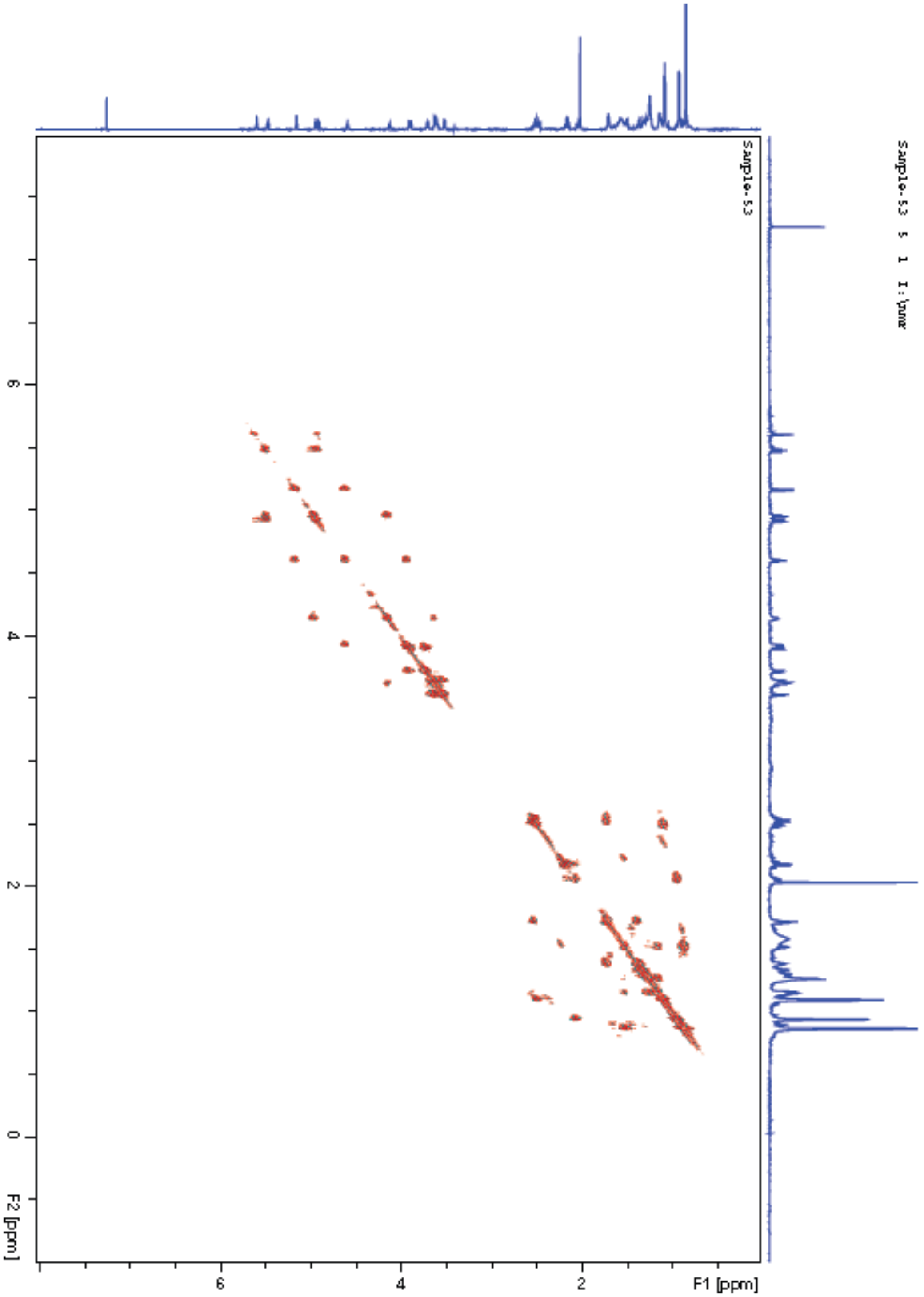
F1 [ppm]
F2 [ppm]
Sample-53

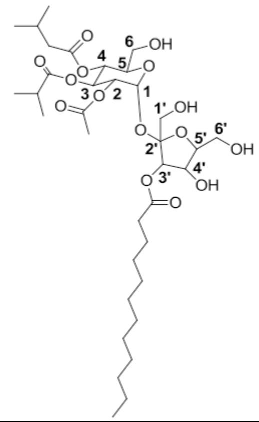

S4:23[6] (2,4,5,12)

Purified from *S. habrochaites* LA1777

HRMS: (ESI) *m/z* calcd for $C_{36}H_{61}O_{17}^{-}$ ([M+HCOO^{-}]): 765.3914, found: 765.3979

Material recovered: 2-3 mg

NMR solvent: $CDCl_3$

InChI Key: QXWXZIRJCBQBBH-KUAWNJLRSA-N

Carbon # (group)	^{1}H (ppm)	^{13}C (ppm)
1(CH)	5.60 (d, *J* = 3.6 Hz)	89.5
2(CH)	4.91 (dd, *J* = 10.3, 3.7 Hz)	70.4
2-O-		
-1(CO)		170.0
-2(CH_3)	2.03 (s)	20.6
3(CH)	5.47 (t, *J* = 10.0 Hz)	68.8
3-O-		
-1(CO)		175.8
-2(CH)	2.48 (m)	33.9
-3(CH_3) x 2	1.09 (d, *J* = 5.8 Hz), 1.09 (d, *J* = 5.9 Hz)	19.0
4(CH)	4.95 (t, *J* = 10.0 Hz)	68.2
4-O		
-1(CO)		172.2
-2(CH_2)	2.16 (dd, *J* = 15.0, 7.1 Hz), 2.18 (dd, *J* = 15.0, 7.1 Hz) [a]	42.9
-3(CH)	2.05 (m)	25.4
-4(CH_3) x 2	0.93 (d, *J* = 6.6 Hz), 0.93 (d, *J* = 6.6 Hz)	22.3
5(CH)	4.13 (m)	72.1

6(CH_2)	3.62 (m)	61.6
1' (CH_2)	3.52 (d, J = 12.3 Hz), 3.62 (m)	64.5
2' (C)		104.0
3' (CH)	5.16 (d, J = 8.0 Hz)	79.9
3'-O		
-1(CO)		175.0
-2(CH_2)	2.52 (m)	34.0
-3(CH_2)	1.71 (quin, J = 7.8 Hz)	24.8
-4(CH_2)	1.37 (m)	29.3
-5(CH_2)	1.33 (m)	29.1
-6-9(CH_2-CH_2-CH_2-CH_2)	1.23-1.30 (br. m)	29.3, 29.6, 29.6, 29.7
-10(CH_2)	1.23-1.30 (br. m)	31.9
-11(CH_2)	1.28[b]	22.7
-12(CH_3)	0.88 (t, J = 7.1 Hz)	14.1
4' (CH)	4.59 (t, J = 8.1 Hz)	71.3
5' (CH)	3.92 (m)	82.4
6' (CH_2)	3.71 (d, J = 12.7 Hz), 3.90 (m)	59.6
[a]Higher order multiplet derived from the constants using gNMR<br>[b]Determined by COSY		

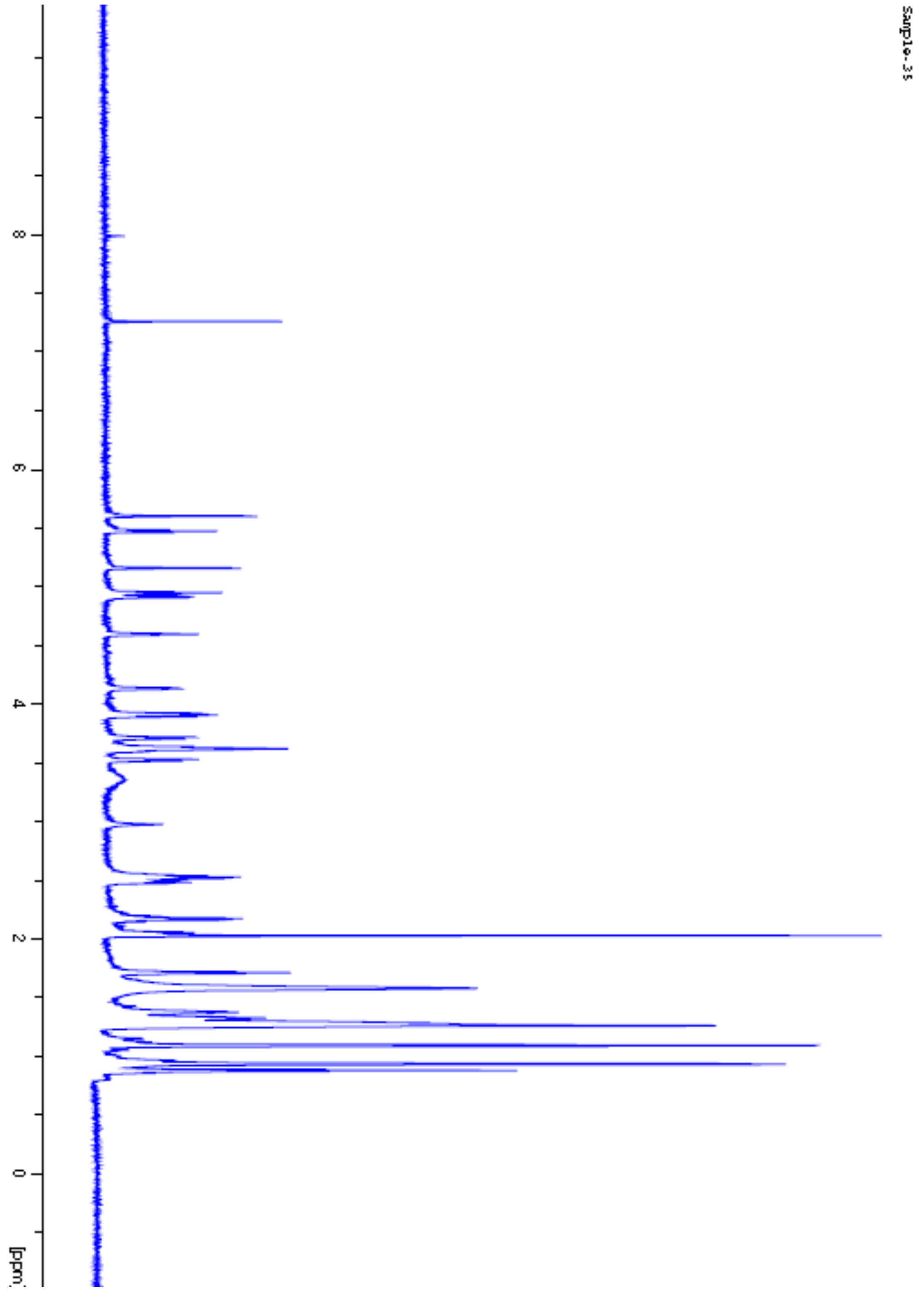
8
6
4
2
0
[ppm]

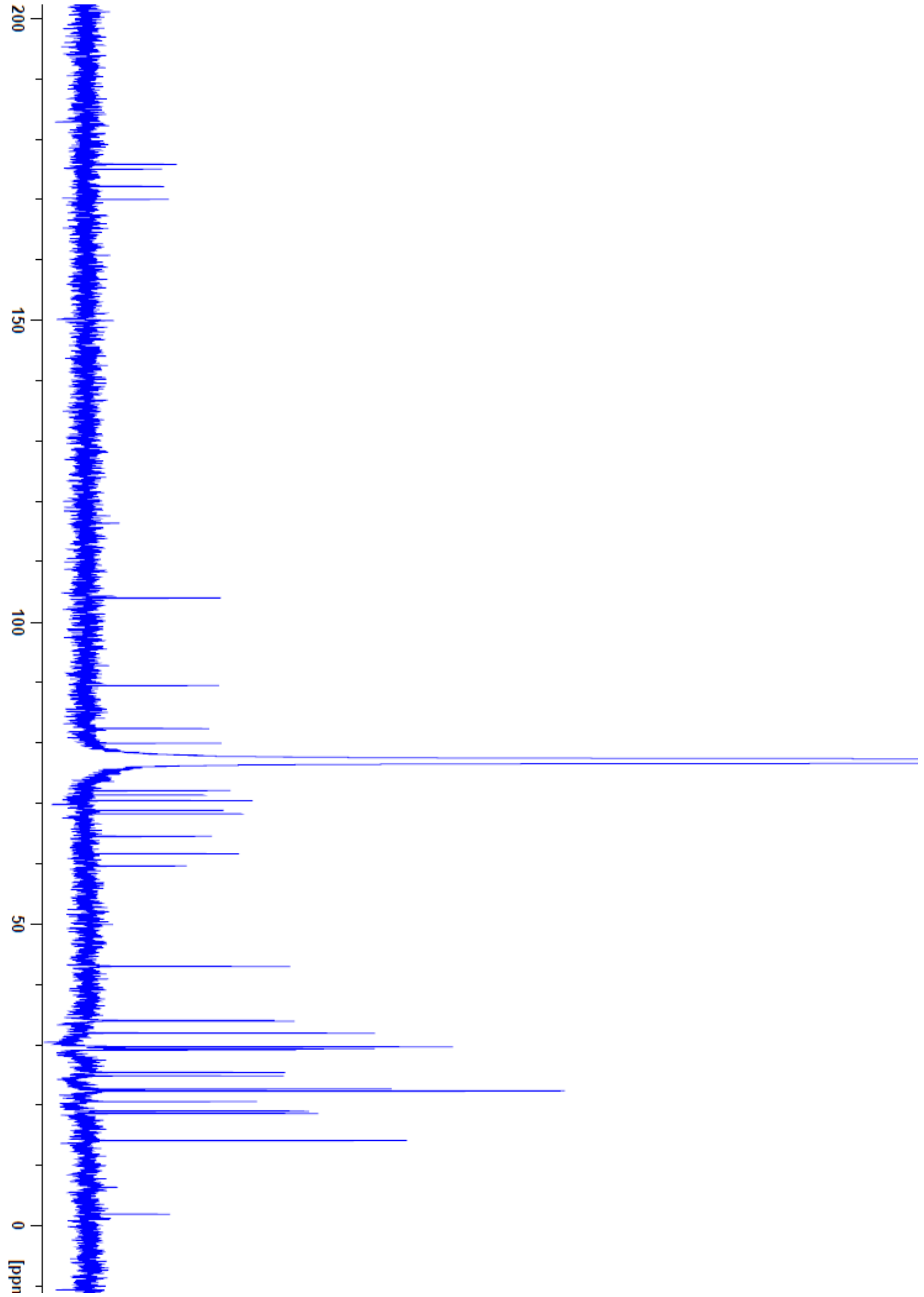
200
150
100
50
0
[ppm

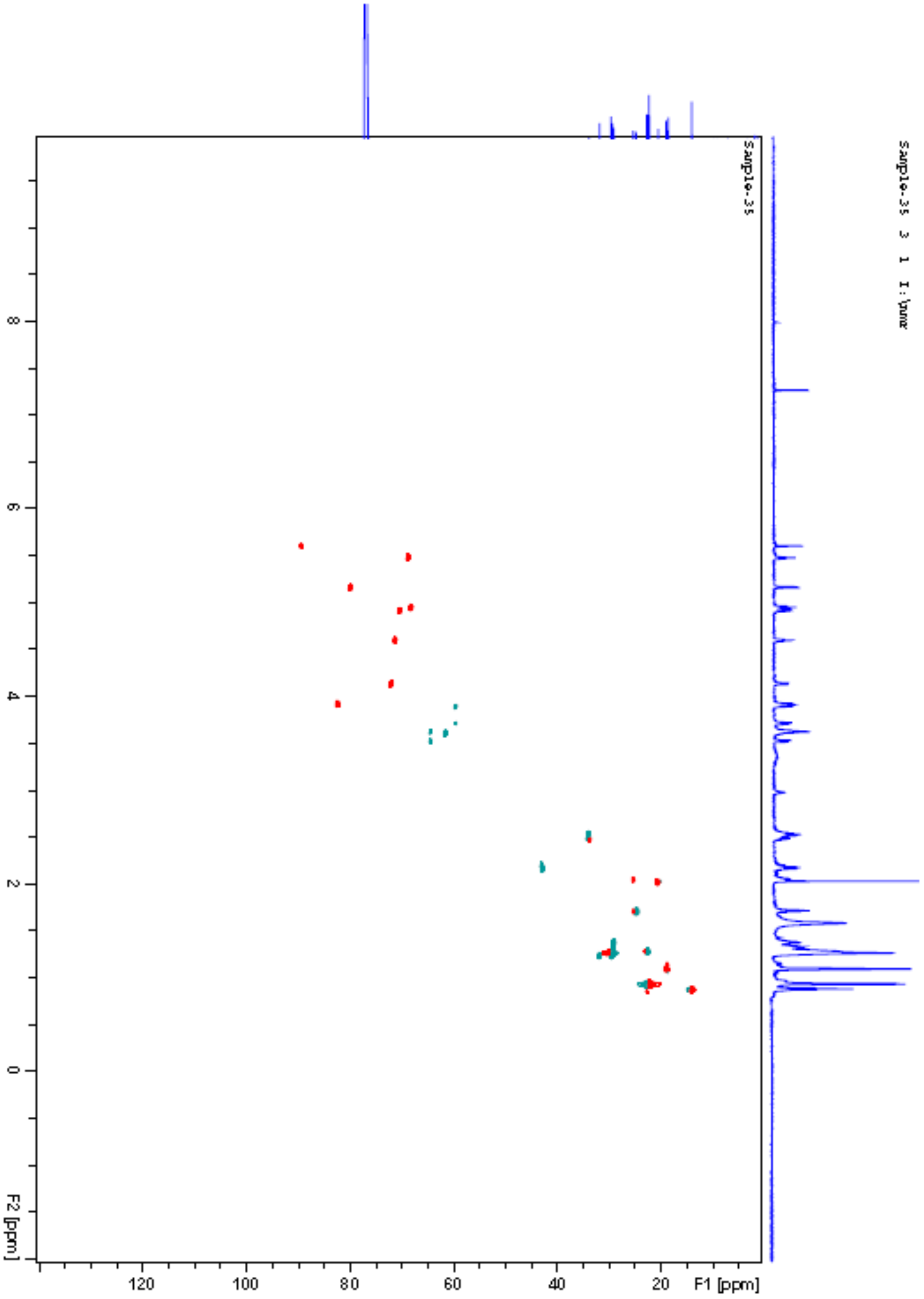

Sample-35
Sample-35 3 1 I:\nmr
F2 [ppm]
F1 [ppm]
120
100
80
60
40
20

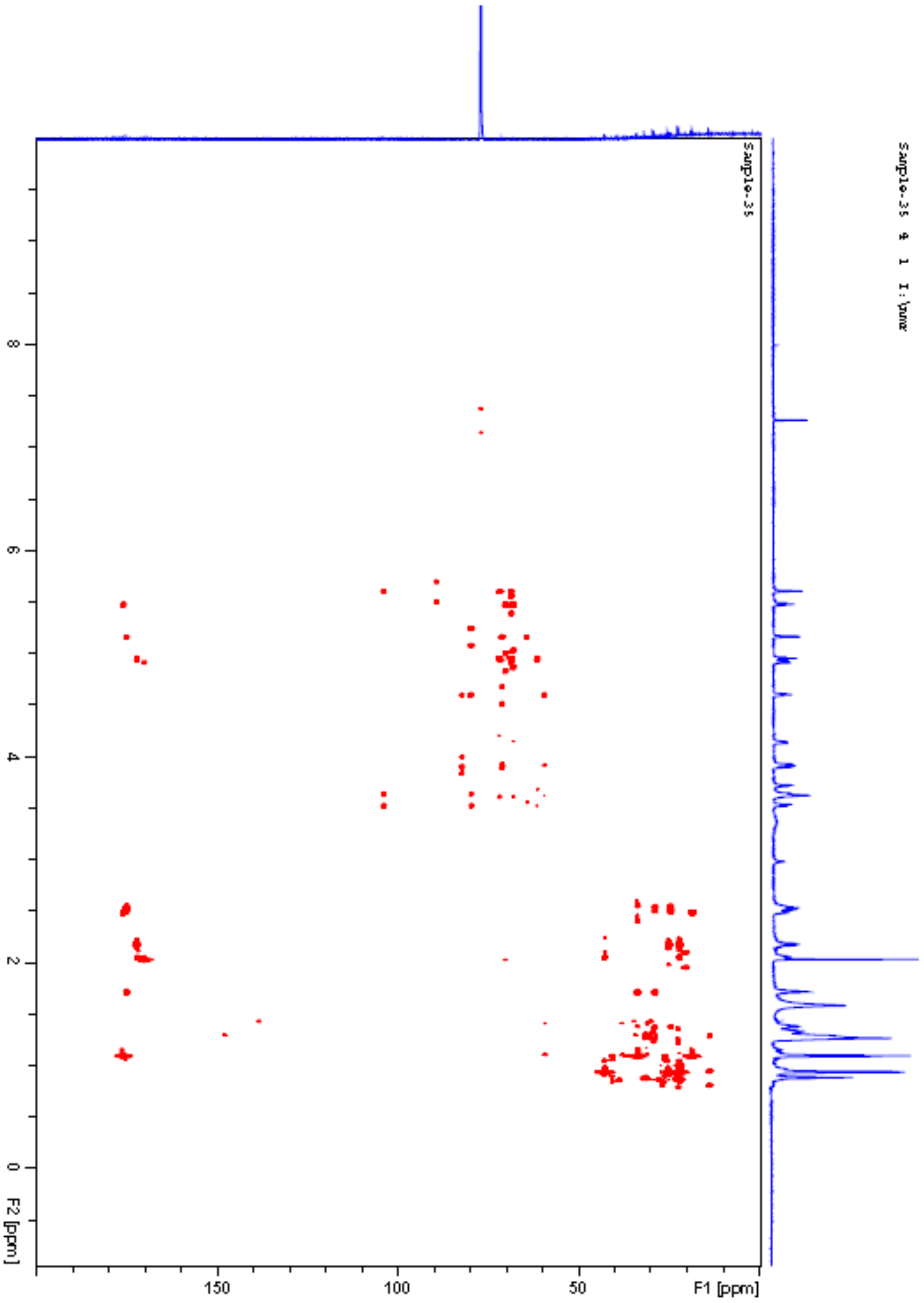
Sample-35
8
6
4
2
0
F2 [ppm]
150
100
50
F1 [ppm]

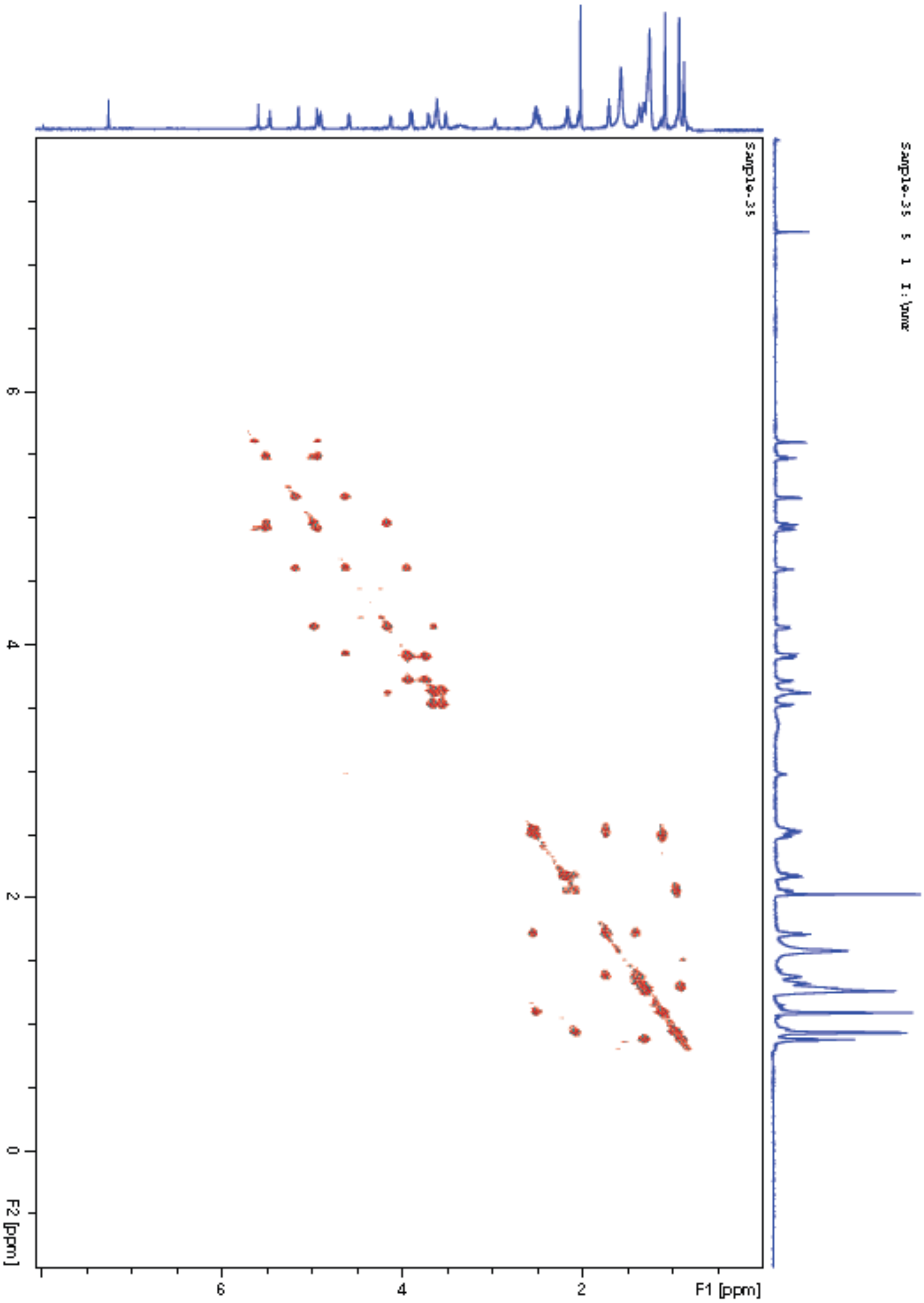
Sample-35
F1 [ppm]
F2 [ppm]
6
4
2
0

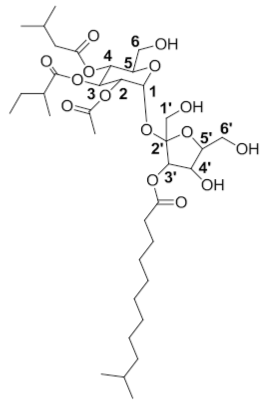

S4:24[5] (2,5,5,12)

Purified from *S. habrochaites* LA1392

HRMS: (ESI) *m/z* calcd for $C_{37}H_{63}O_{17}^{-}$ ([M+HCOO^{-}]): 779.4071, found: 779.4138

Material recovered: 1-2 mg

NMR solvent: $CDCl_3$

InChI Key: FYOAITQAGUKZIH-JBZCSEOXSA-N

Carbon # (group)	^{1}H (ppm)	^{13}C (ppm)
1(CH)	5.61 (d, *J* = 3.7 Hz)	89.3 ($^{1}J_{CH}$ = 177.9 Hz)
2(CH)	4.88 (dd, *J* = 10.4, 3.5 Hz)	70.4
2-O-		
-1(CO)		170.3
-2(CH_3)	2.02 (s)	20.7
3(CH)	5.49 (t, *J* = 10.0 Hz)	68.6
3-O-		
-1(CO)		175.5
-2(CH)	2.31 (m)	40.8
-2'(CH_3)	1.06 (d, *J* = 7.0)	16.4
-3(CH_2)	1.41 (m), 1.61[a]	26.5
-4(CH_3)	0.83-0.86 (m)	11.4
4(CH)	4.93 (t, *J* = 10.0)	68.5
4-O		
-1(CO)		172.3
-2(CH_2)	2.17 (m)[b]	43.0
-3(CH)	2.05 (m)	25.3
-4(CH_3) x 2	0.94 (m)	22.6, 22.7
5(CH)	4.13 (m)	71.9

6(CH_2)	3.61 (m)	61.6
1' (CH_2)	3.52 (d, J = 12.6 Hz), 3.63 (d, J = 12.6 Hz)	64.4
2' (C)		103.8
3' (CH)	5.14 (d, J = 8.0 Hz)	79.8
3'-O		
-1(CO)		175.1
-2(CH_2)	2.53 (m)	34.0
-3(CH_2)	1.70 (quin, J = 7.5 Hz)	24.8
-4(CH_2)	1.36 (m)	29.1, 29.3
-5(CH_2)	1.31 (m)	
-6-8(CH_2-CH_2-CH_2)	1.21-1.29 (br. m)	27.4, 29.6, 29.9
-9(CH_2)	1.12 (m)	39.0
-10(CH)	1.50[b]	27.9
-11(CH_3) x 2	0.83-0.86 (m)	22.7
4' (CH)	4.60 (t, J = 8.1 Hz)	71.0
5' (CH)	3.90 (m)	82.1
6' (CH_2)	3.71 (d, J = 13.0), 3.90 (m)	59.4

[a]Determined by COSY and HSQC

[b]Determined by COSY

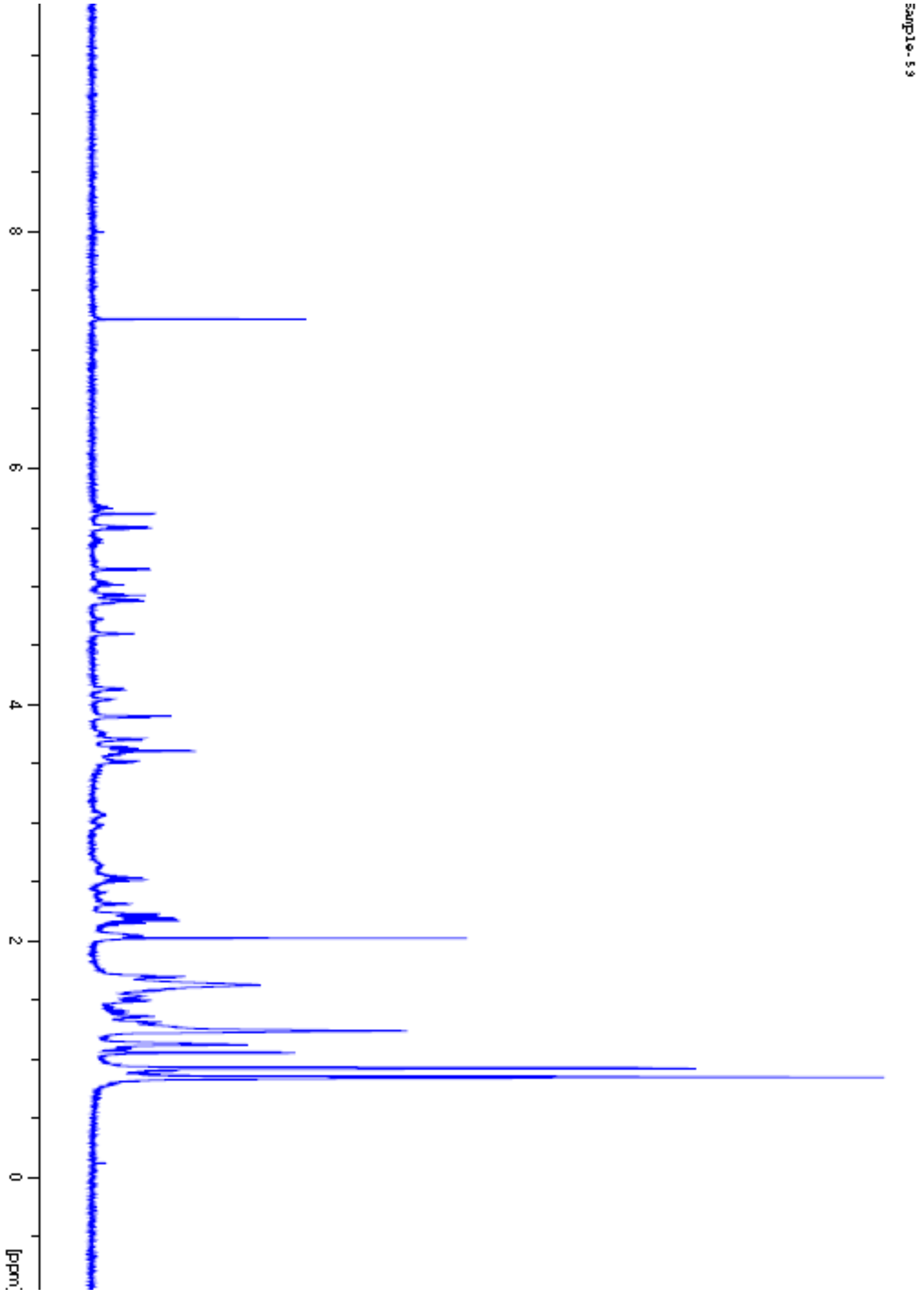
8
6
4
2
0
[ppm]

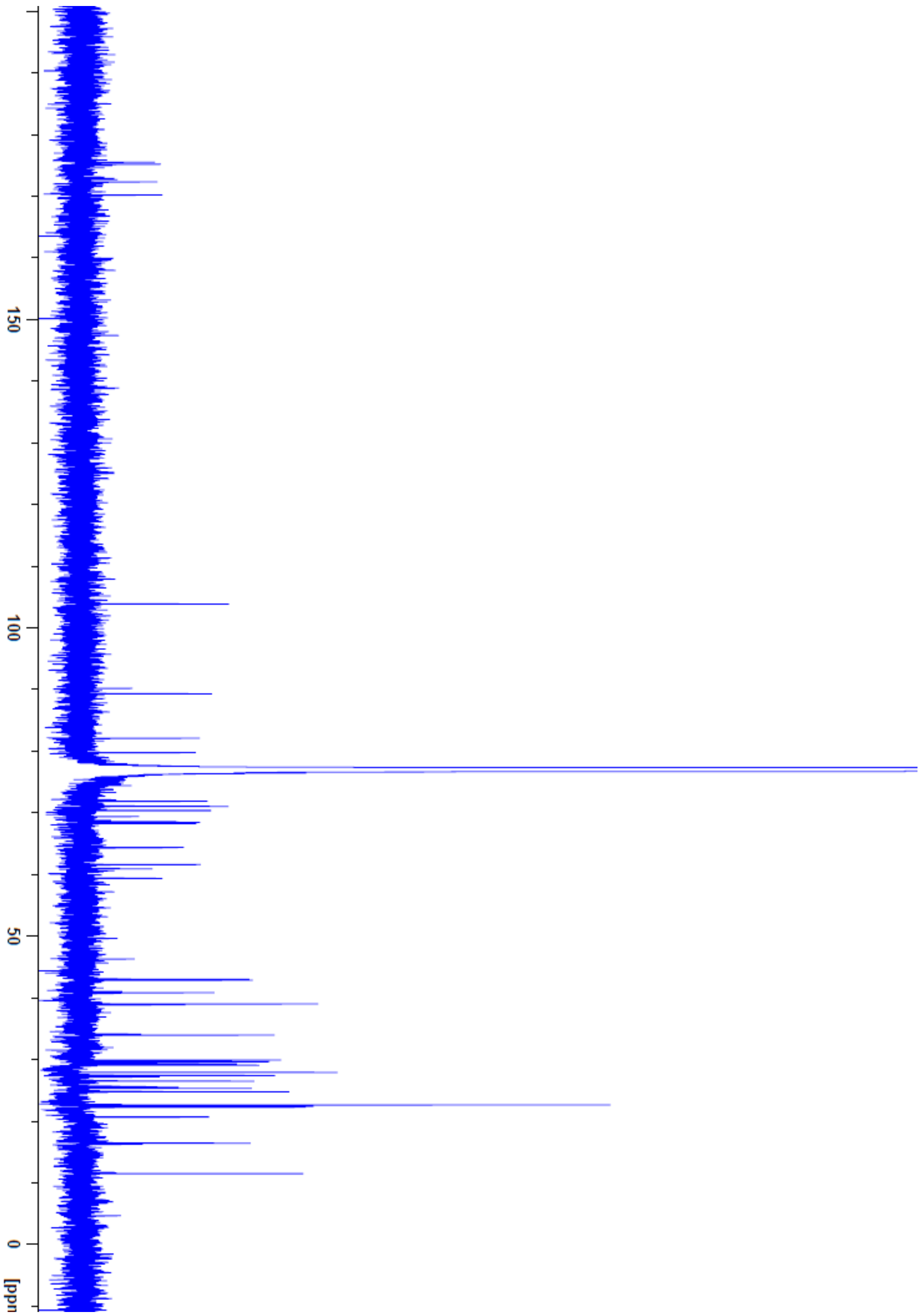
150
100
50
0
[ppm]

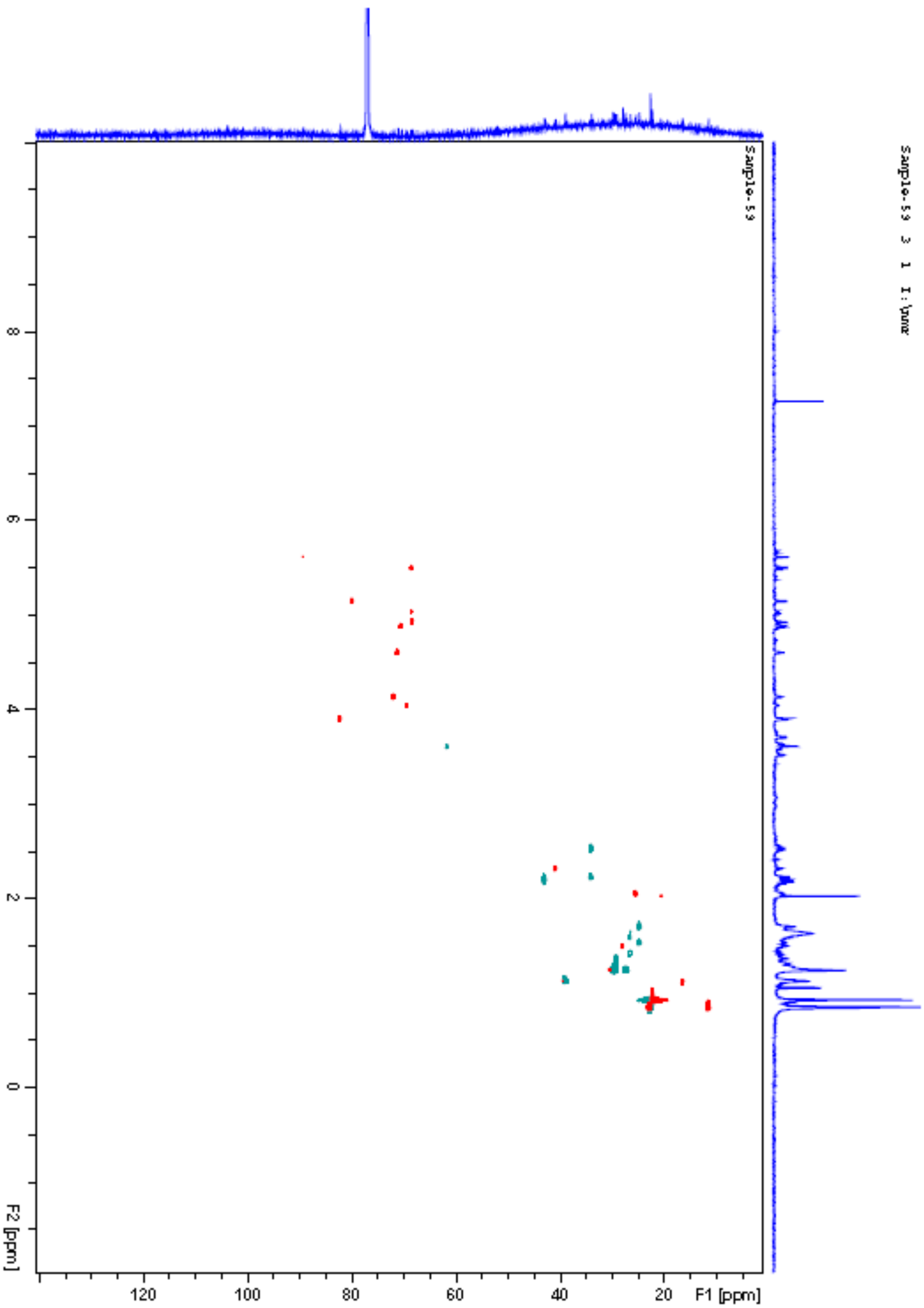
Sample-59
Sample-59 3 1 I:\nmr
F1 [ppm]
F2 [ppm]
120
100
80
60
40
20
8
6
4
2
0

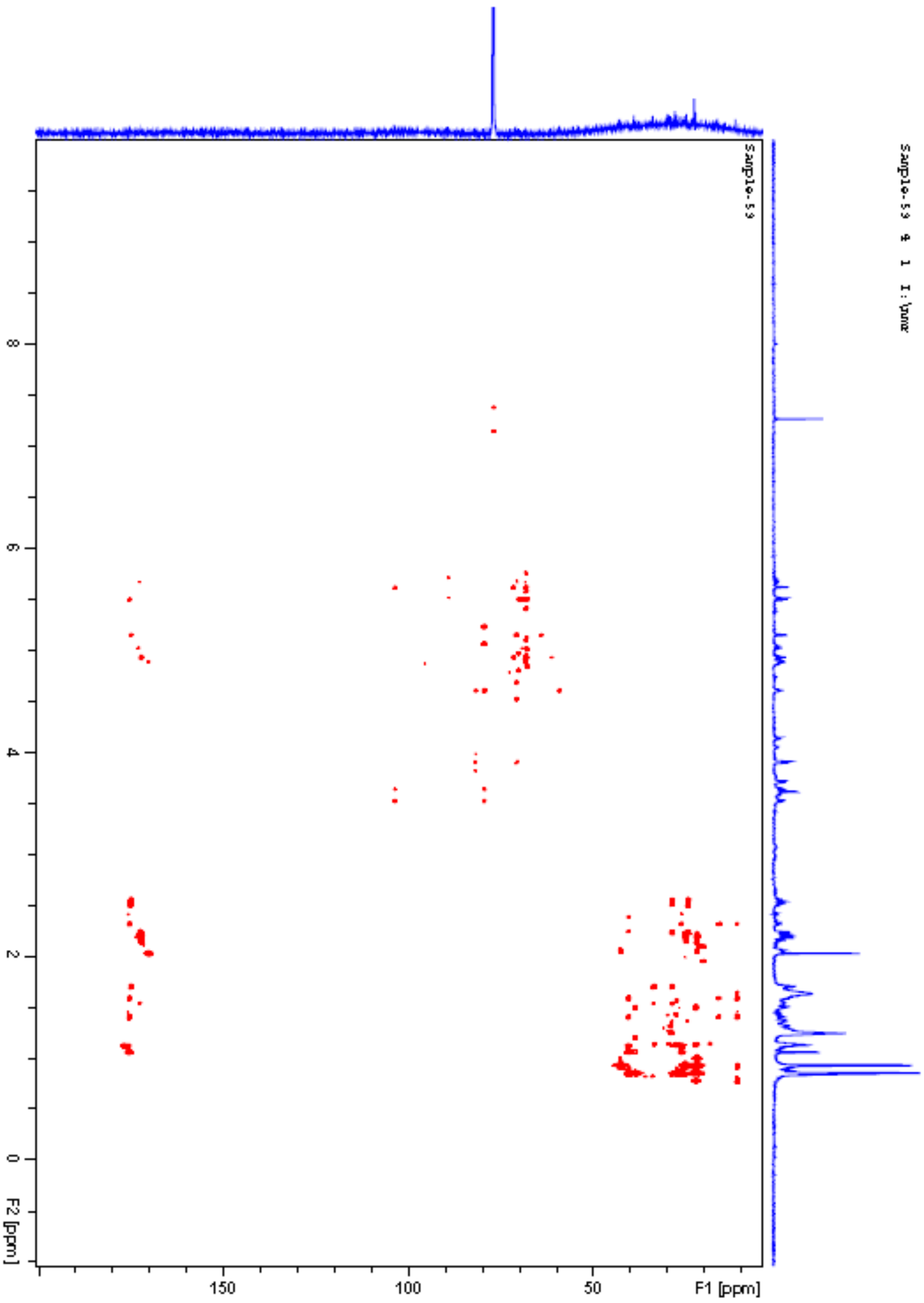
F1 [ppm]
150
100
50
F2 [ppm]
8
6
4
2
0

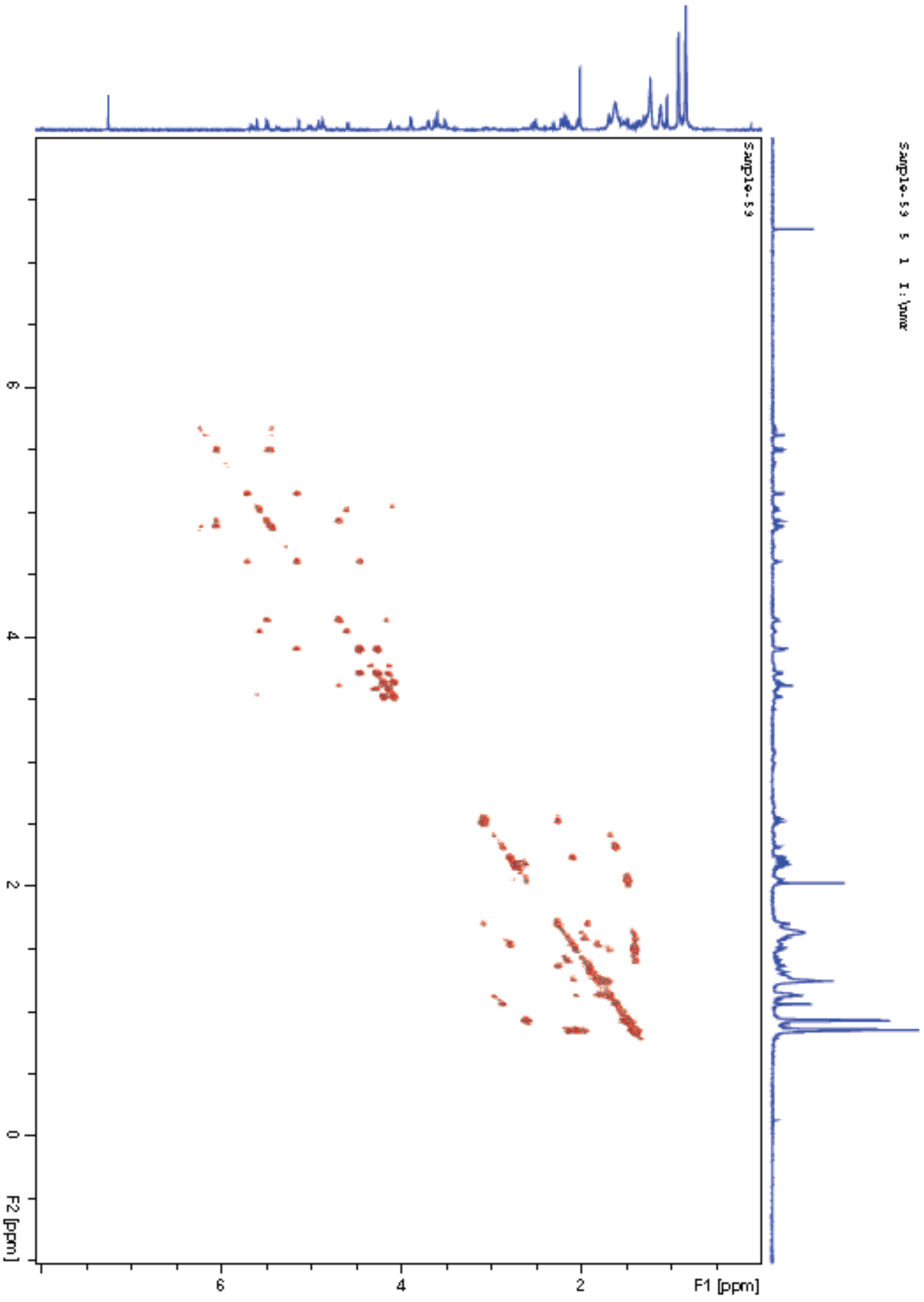

F1 [ppm]
F2 [ppm]

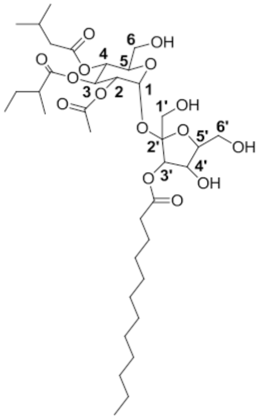

S4:24[6] (2,5,5,12)

Purified from *S. habrochaites* LA1777

HRMS: (ESI) *m/z* calcd for $C_{37}H_{63}O_{17}^{-}$ ([M+HCOO^{-}]): 779.4071, found: 779.4132

Material recovered: 1-2 mg

NMR solvent: $CDCl_3$

InChI Key: IOLOYIOKSKTZEJ-JBZCSEOXSA-N

Carbon # (group)	^{1}H (ppm)	^{13}C (ppm)
1(CH)	5.61 (d, *J* = 3.5 Hz)	89.4 ($^{1}J_{CH}$ = 177.6 Hz)
2(CH)	4.89 (dd, *J* = 10.2, 3.6 Hz)	70.6
2-O-		
-1(CO)		170.1
-2(CH_3)	2.02 (s)	20.7
3(CH)	5.50 (t, *J* = 10.0 Hz)	68.8
3-O-		
-1(CO)		175.6
-2(CH)	2.32 (m)	40.7
-2'(CH_3)	1.06 (d, *J* = 7.0)	16.5
-3(CH_2)	1.41 (m), 1.61 (m)	26.5
-4(CH_3)	0.85 (t, *J* = 7.5)	11.3
4(CH)	4.94 (t, *J* = 10.1)	68.4
4-O		
-1(CO)		172.3
-2(CH_2)	2.17 (m)	43.0
-3(CH)	2.05 (m)	25.5
-4(CH_3) x 2	0.93(m)	22.1

5(CH)	4.13 (m)	72.1
6(CH_2)	3.62 (m)	61.7
1' (CH_2)	3.52 (d, J = 12.6 Hz), 3.64 (d, J = 12.6 Hz)	64.5
2' (C)		104.0
3' (CH)	5.15 (d, J = 8.1 Hz)	80.1
3'-O		
-1(CO)		175.2
-2(CH_2)	2.52 (m)	34.1
-3(CH_2)	1.71 (quin, J = 7.6 Hz)	24.8
-4(CH_2)	1.37 (m)	29.1
-5(CH_2)	1.32 (m)	29.2
-6-9(CH_2-CH_2-CH_2-CH_2)	1.23-1.30 (br. m)	29.6
-10(CH_2)	1.23-1.30 (br. m)	31.9
-11(CH_2)	1.29[a]	22.6
-12(CH_3)	0.88 (t, J = 7.1 Hz)	14.0
4' (CH)	4.60 (t, J = 8.1 Hz)	71.3
5' (CH)	3.90 (m)	82.4
6' (CH_2)	3.71 (d, J = 12.8), 3.90 (m)	59.6
[a]Determined by COSY		

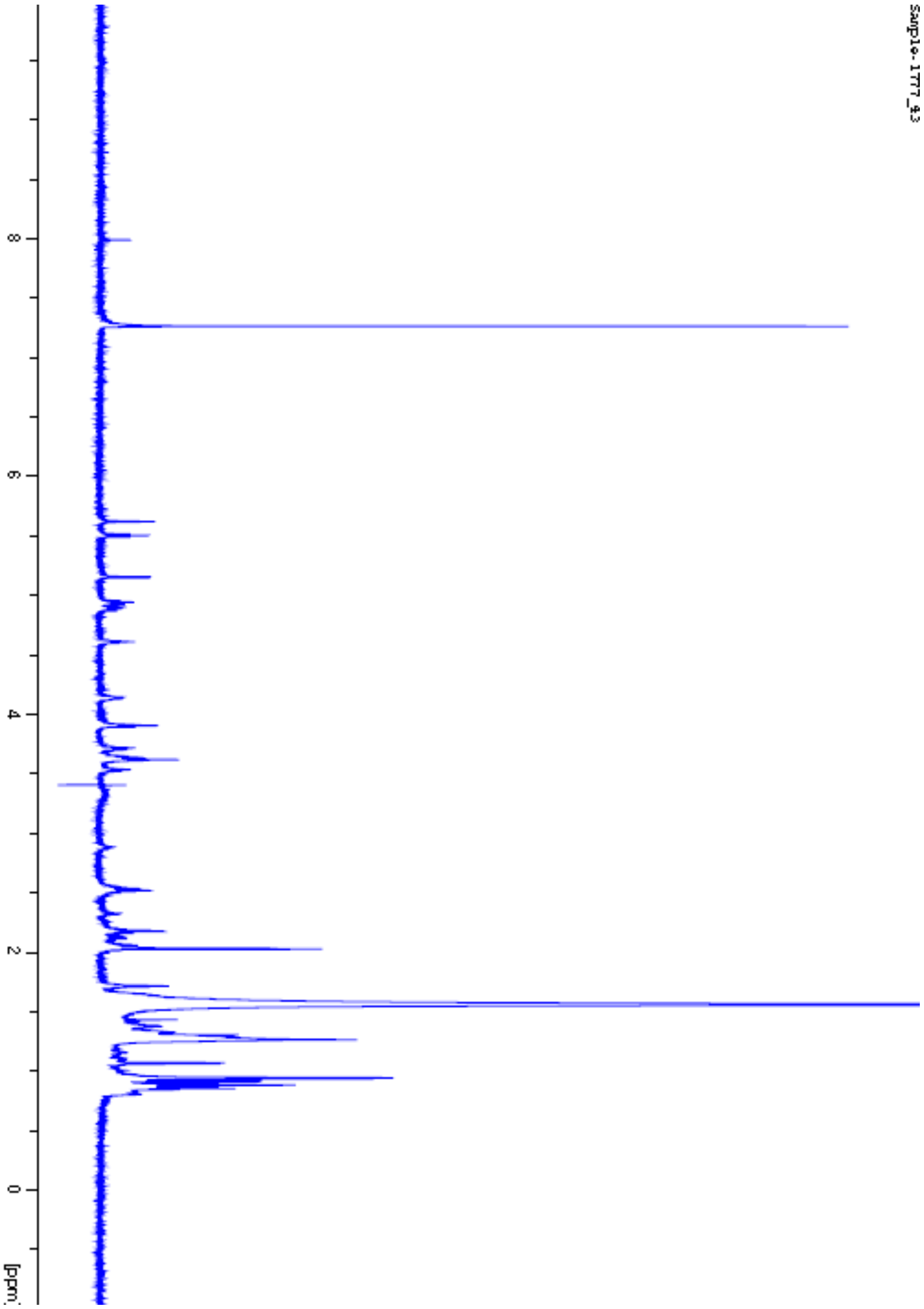
Sample-1777_43
8
6
4
2
0
[ppm]

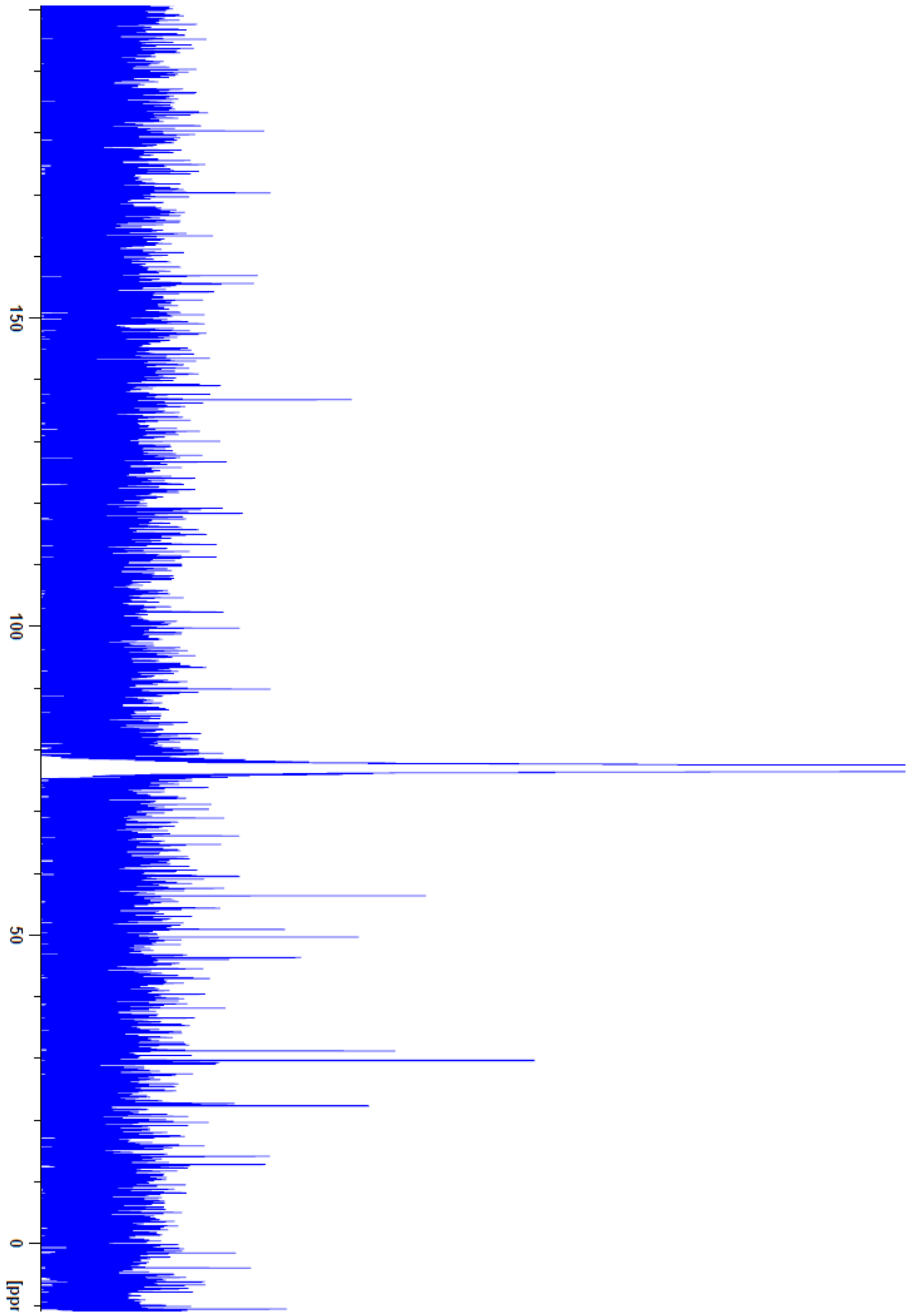
150
100
50
0

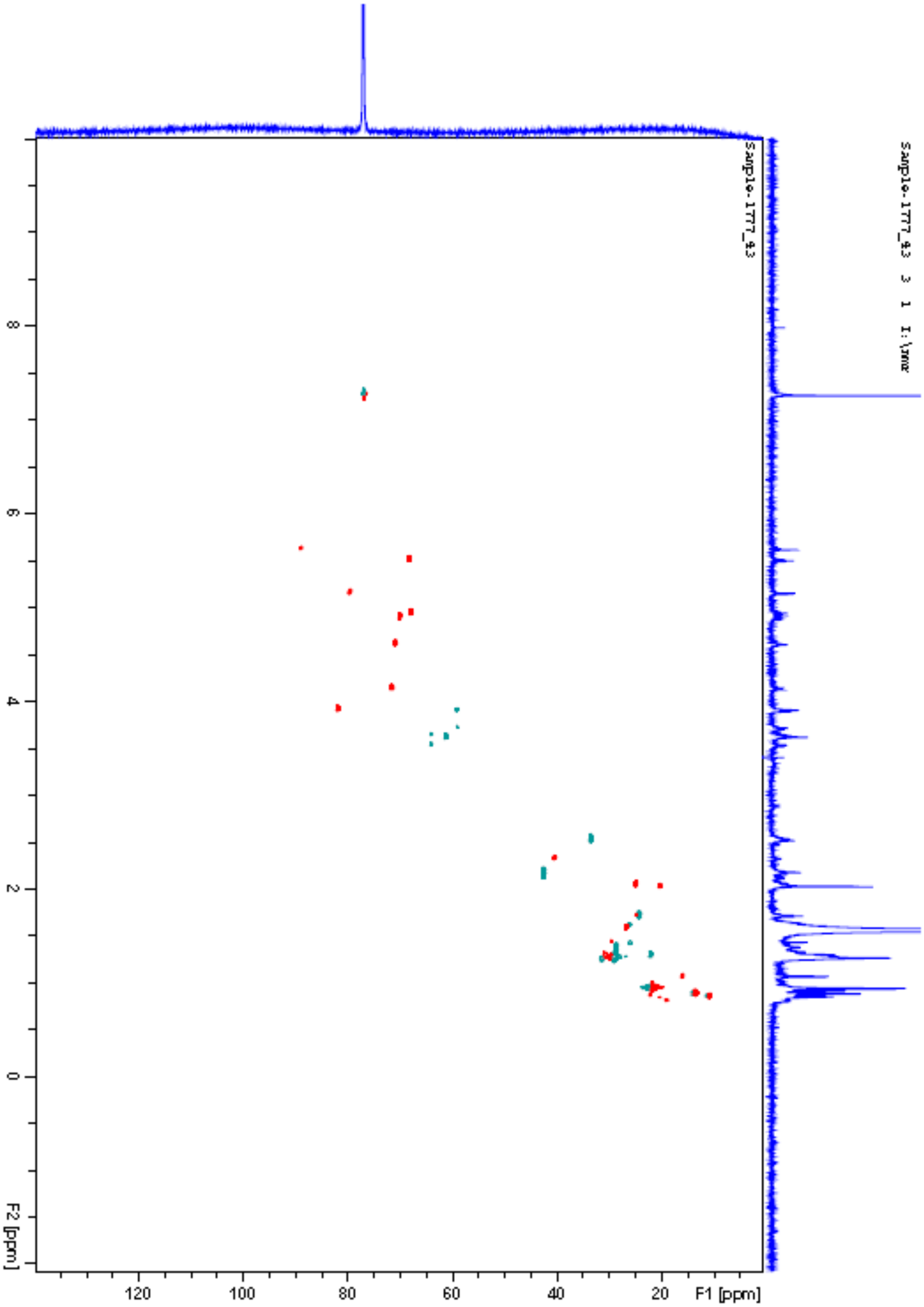
F1 [ppm]
F2 [ppm]

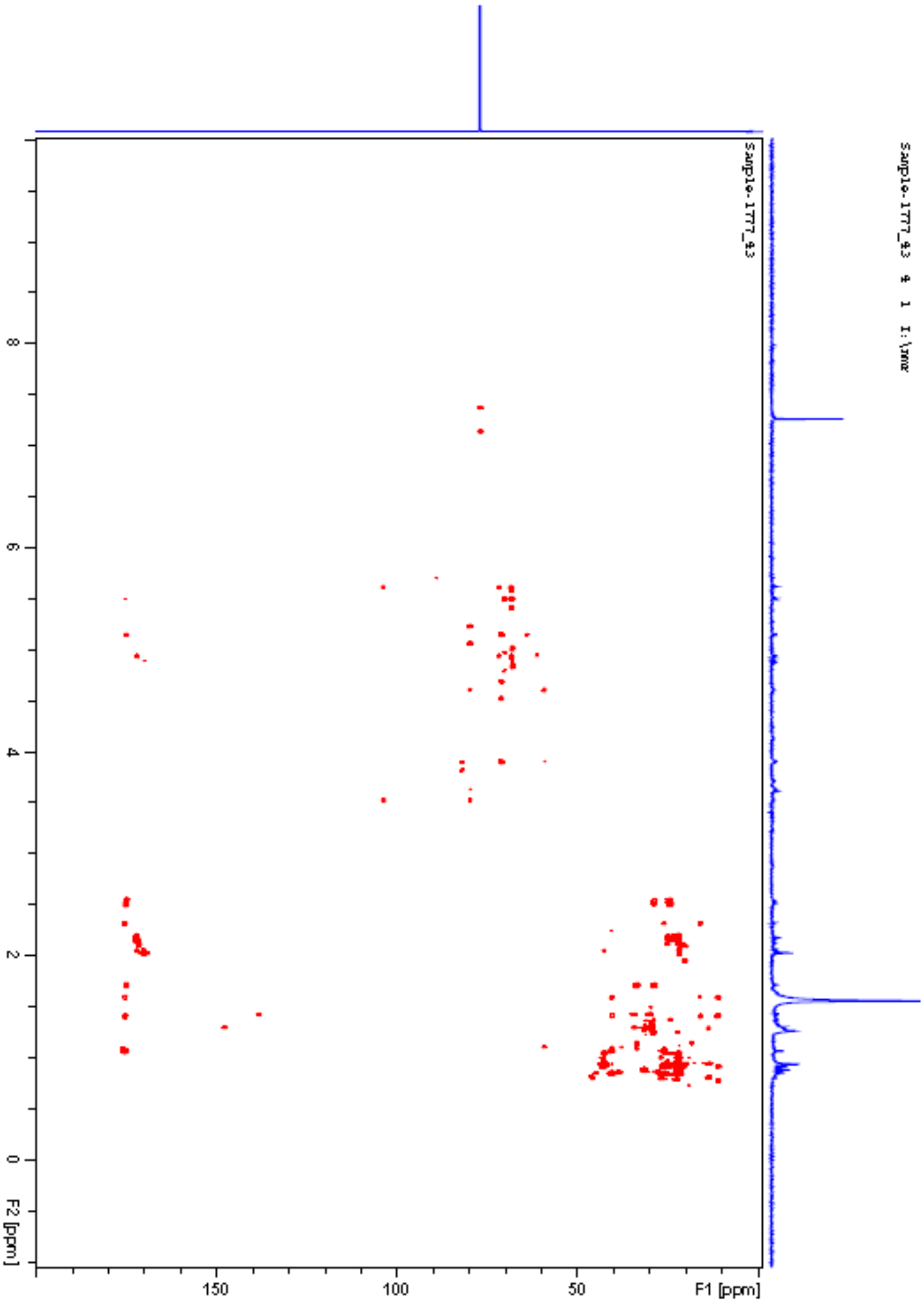
150
100
50
F1 [ppm]

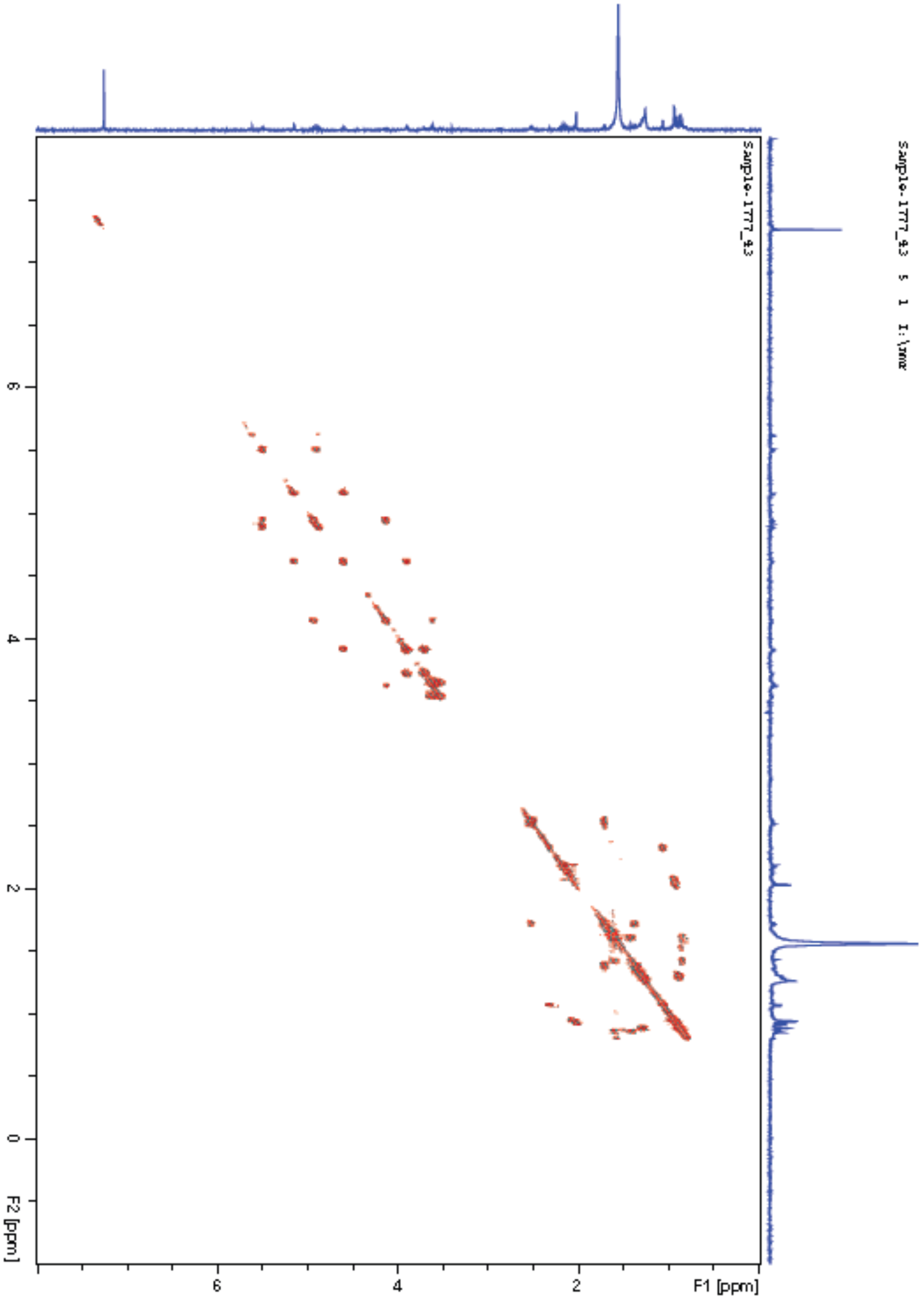
F1 [ppm]
F2 [ppm]

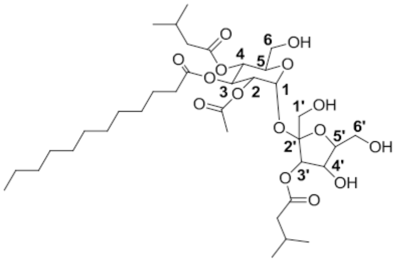

S4:24[8] (2,5,5,12)

Purified from *S. habrochaites* LA1777

HRMS: (ESI) *m/z* calcd for $C_{37}H_{63}O_{17}^-$ ([M+HCOO$^-$]): 779.4071, found: 779.4109

Material recovered: 0.5 - 1 mg

NMR solvent: $CDCl_3$

InChI Key: DXKSESFYAWUSFS-FMUUPEMKSA-N

Carbon # (group)	^{1}H (ppm)	^{13}C (ppm)[a]
1(CH)	5.60 (d, J = 3.8 Hz)	89.4 ($^1J_{CH}$ = 177.8 Hz)
2(CH)	4.90 (dd, J = 10.4, 3.8 Hz)	70.0
2-O-		
-1(CO)		170.0
-2(CH_3)	2.03 (s)	20.4
3(CH)	5.47 (t, J = 10.1 Hz)	68.6
3-O-		
-1(CO)		172.6
-2(CH_2)	2.22 (m)	33.7
-3(CH_3)	1.53[b]	24.6
-4-9(CH_2-CH_2-CH_2-CH_2)	1.24 (m)	See below[c]
-10(CH_2)	1.24 (m)	31.8
-11(CH_2)	1.29 (m)	22.6
-12(CH_3)	0.88 (t, J = 7.3 Hz)	14.0
4(CH)	4.93 (t, J = 10.0 Hz)	68.0
4-O		
-1(CO)		172.3
-2(CH_2)	2.17 (dd, J = 15.0, 7.1 Hz), 2.19 (dd, J = 15.0, 7.1 Hz)[d]	42.6
-3(CH)	2.06 (m)	25.1

-4(CH_3) x 2	0.93 (d, *J* = 6.7 Hz)	22.0
5(CH)	4.12 (m)	71.8
6(CH_2)	3.61 (m)	61.3
1' (CH_2)	3.50 (dd, *J* = 12.2, 6.4 Hz), 3.61 (m)	64.1
2' (C)		103.8
3' (CH)	5.19 (d, *J* = 8.3 Hz)	79.4
3'-O		
-1(CO)		174.3
-2(CH_2)	2.39 (dd, *J* = 15.0, 7.1 Hz), 2.42 (dd, *J* = 15.0, 7.1 Hz) [c]	42.7
-3(CH)	2.21 (m)	25.5
-4(CH_3) x 2	1.05 (d, *J* = 1.8 Hz)	22.8
4' (CH)	4.60 (ddd, *J* = 8.3, 3.5, 3.5 Hz)	70.8
5' (CH)	3.94 (ddd, *J* = 8.5, 2.3, 2.3 Hz)	82.2
6' (CH_2)	3.71 (m), 3.90 (dd, *J* = 13.0, 2.2 Hz)	59.3

[a]Determined by HSQC and HMBC

[b]Determined by COSY

[c]All six carbons have resonances at ~ 29.0 ppm and could not be determined from HSQC.

[d]Higher order multiplet derived from the constants using gNMR.

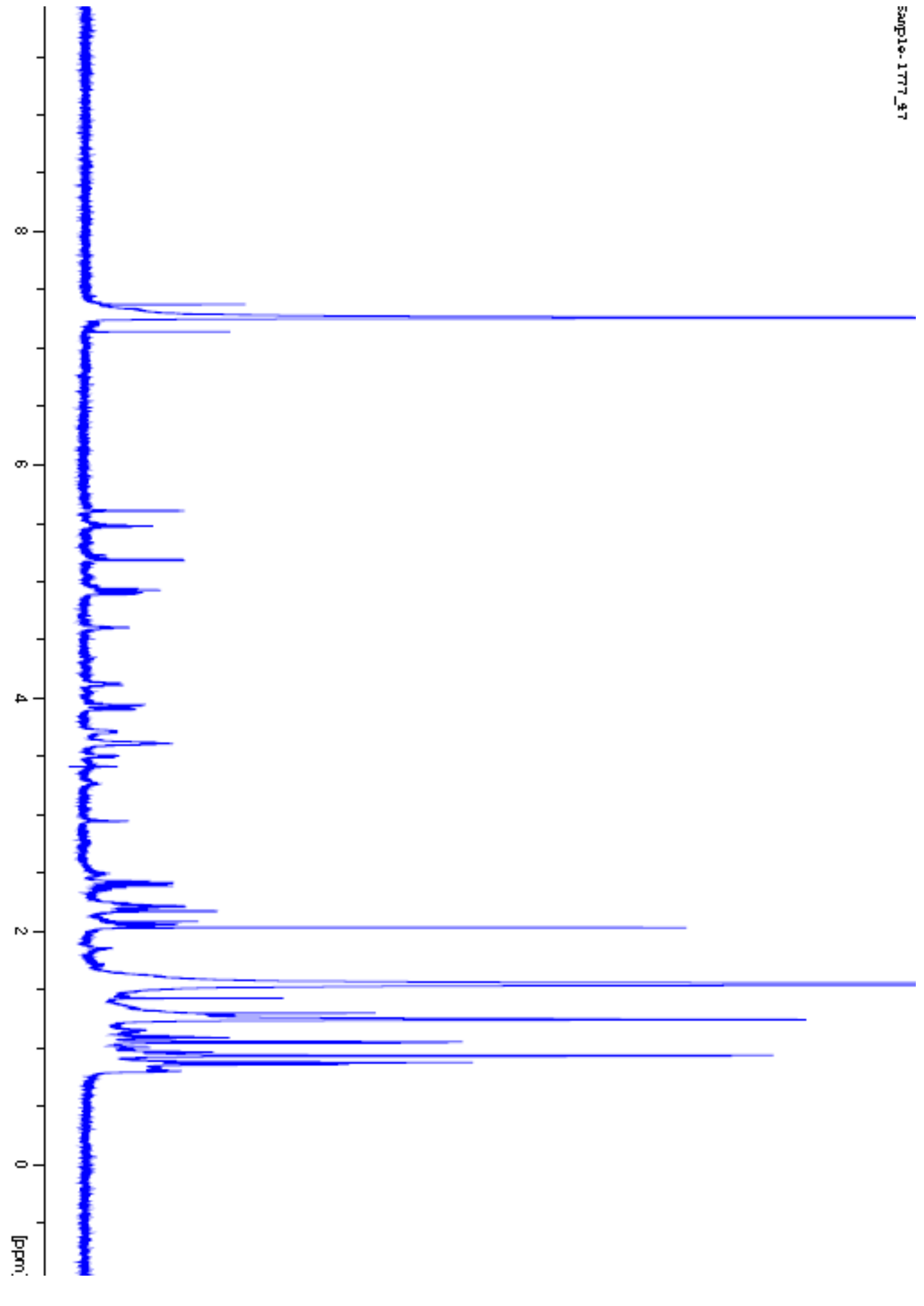
8
6
4
2
0
[ppm]

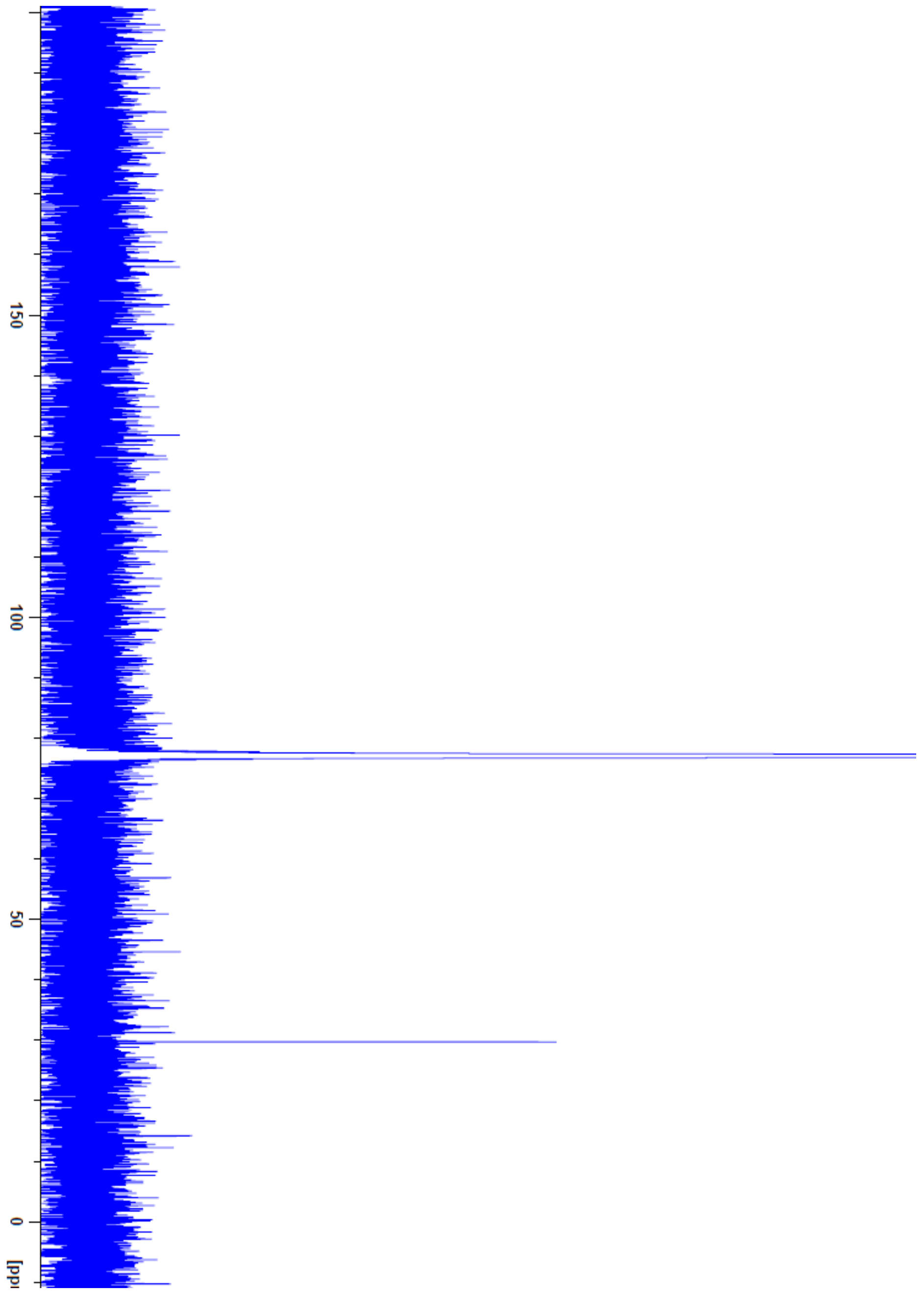
150
100
50
0

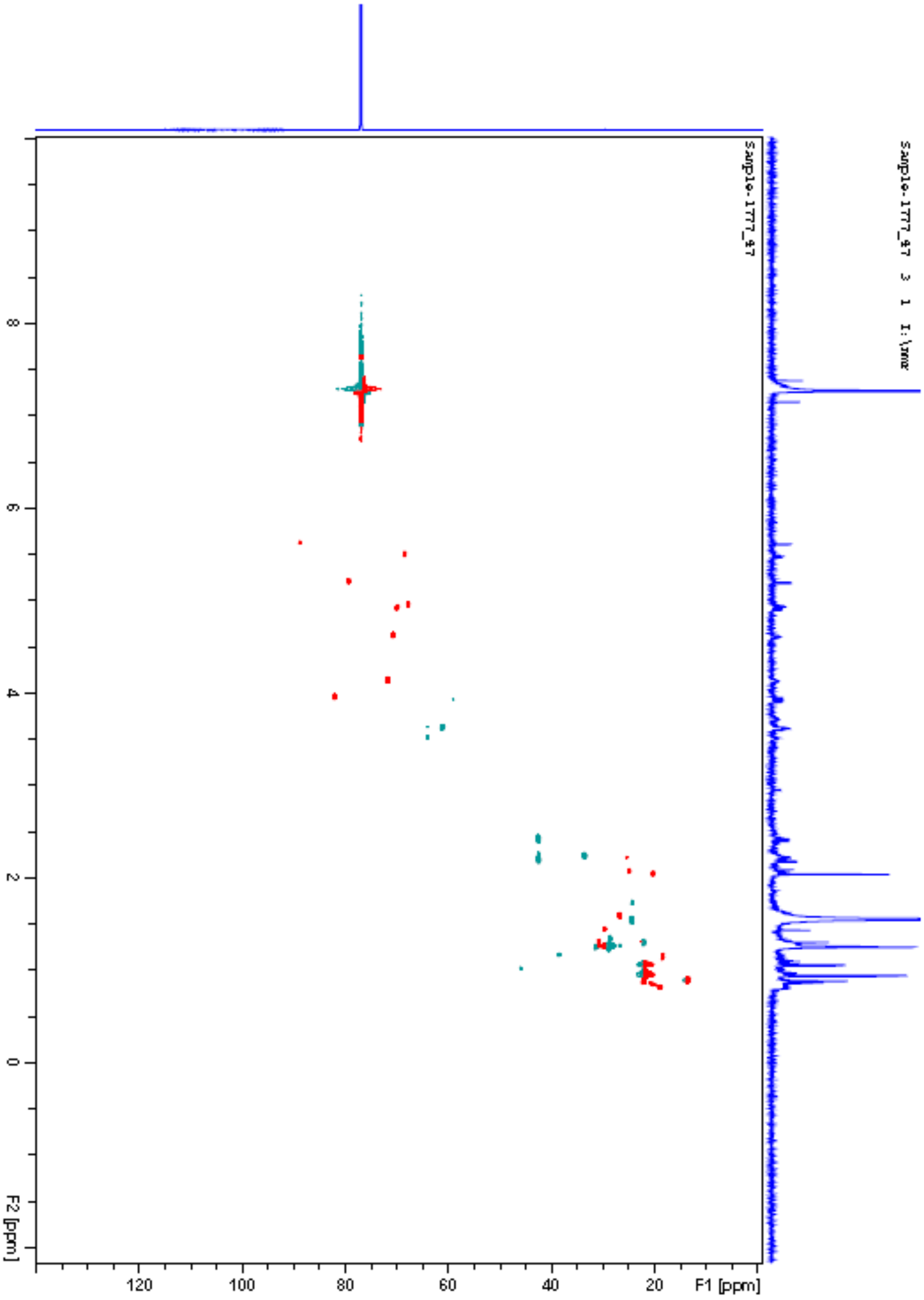
Sample-1777_47
Sample-1777_47 3 1 I:\nmr
F2 [ppm]
F1 [ppm]

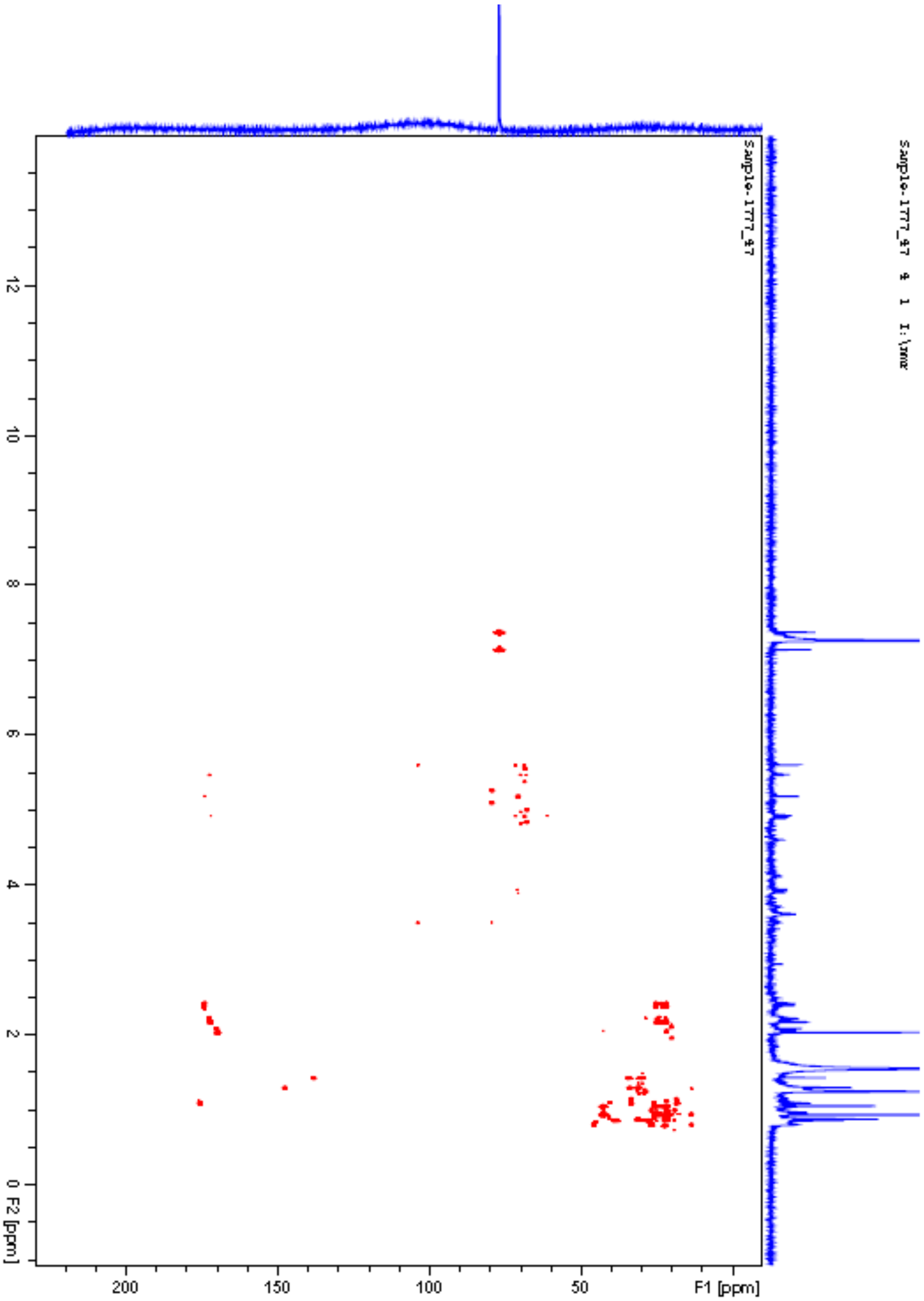
Sample-1777_47
Sample-1777_47 4 1 I:\nmr
F1 [ppm]
F2 [ppm]
200
150
100
50
12
10
8
6
4
2
0

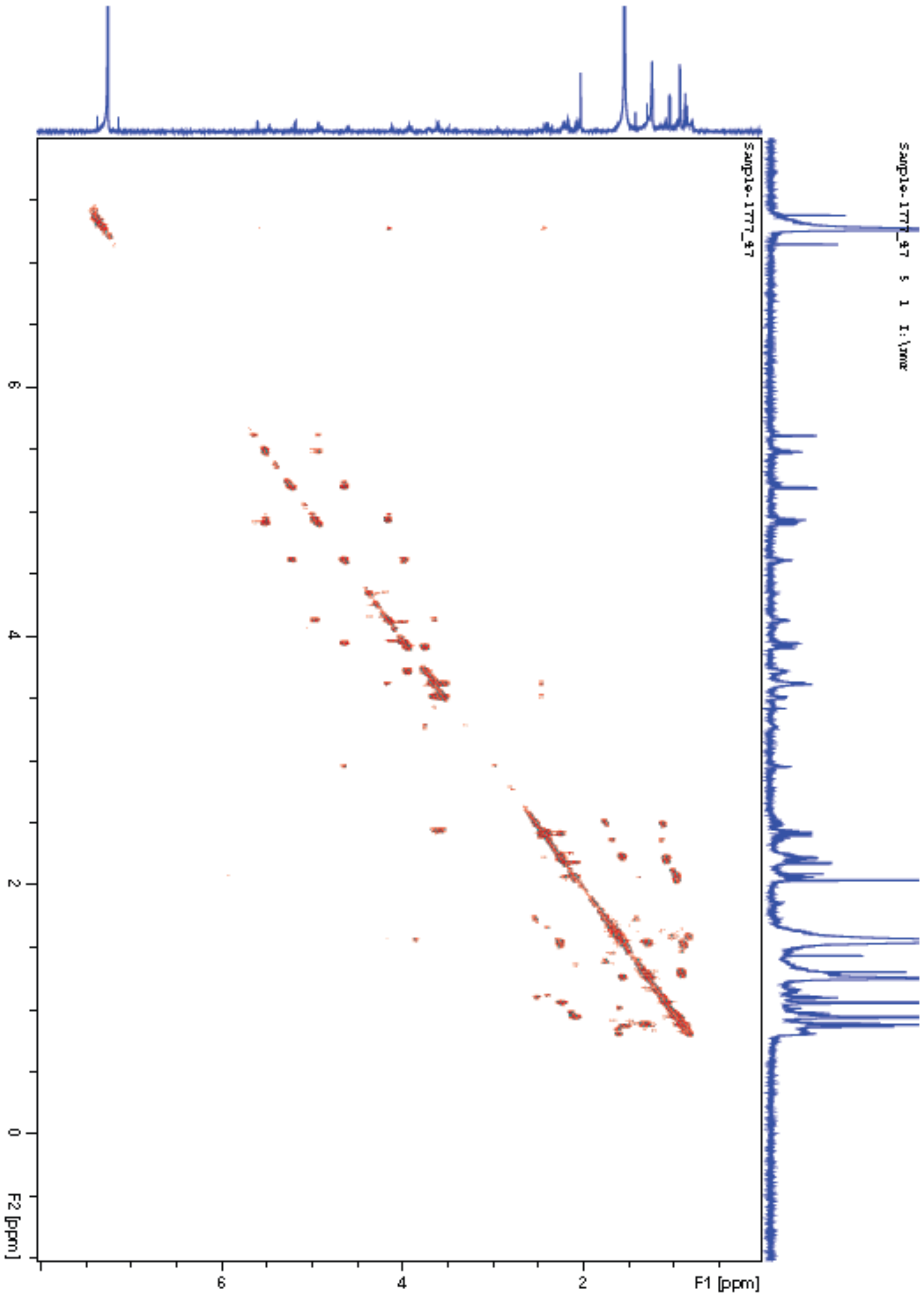
Sample-1777_47
Sample-1777_47 5 1 I:\nmr
F1 [ppm]
F2 [ppm]
6
4
2
0

<table>
<tr><td>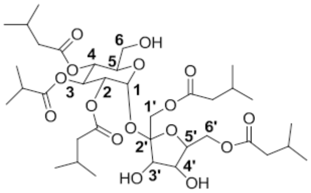
</td><td>

S5:24[3] (4,5,5,5,5)

Purified from *S. habrochaites* LA1362

HRMS: (ESI) *m/z* calcd for $C_{37}H_{61}O_{18}^{-}$ ([M+HCOO^{-}]): 793.3863, found: 793.3953

Material recovered: 2-3 mg

NMR solvent: CD_3CN

InChI Key: CTVXPRQQMZMPDD-PXSRNTGSSA-N

</td></tr>
</table>

Carbon # (group)	^{1}H (ppm)	^{13}C (ppm)
1(CH)	5.53 (d, *J* = 3.9 Hz)	90.0 ($^{1}J_{CH}$ = 177.0 Hz)
2(CH)	4.93 (dd, *J* = 10.3, 3.8 Hz)	70.8
2-O-		
-1(CO)		172.7
-2(CH)$_2$	2.10-2.25 (m)	See below[a]
-3(CH)	1.96-2.00 (m)	See below[b]
-4(CH$_3$) x 2	0.89-0.93 (m)	See below[c]
3(CH)	5.45 (t, *J* = 10.0 Hz)	70.4
3-O-		
-1(CO)		176.7
-2(CH)	2.44 (m)	34.7
-3(CH$_3$) x 2	1.05 (d, *J* = 7.0), 1.05 (d, *J* = 7.0)	19.1
4(CH)	5.02 (t, *J* = 10.0 Hz)	69.3
4-O		
-1(CO)		172.7
-2(CH$_2$)	2.10-2.25 (m)	See below[a]
-3(CH)	1.96-2.00 (m)	See below[b]
-4(CH$_3$) x 2	0.89-0.93 (m)	See below[c]

5(CH)	4.09 (m)	71.4
6(CH_2)	3.48 (dd, *J* = 12.3, 5.6 Hz), 3.59 (dd, *J* = 12.2, 2.3 Hz)	61.7
1' (CH_2)	4.00 (d, *J* = 4.9 Hz)	63.3
1'-O-		
-1(CO)		172.9
-2(CH_2)	2.17-2.25 (m)	See below[a]
-3(CH)	2.01-2.07 (m)	See below[b]
-4(CH_3) x 2	0.94 (d, *J* = 6.6 Hz), 0.94 (d, *J* = 6.6 Hz)	See below[c]
2' (C)		104.0
3' (CH)	3.97 (d, *J* = 8.7 Hz)	77.8
4' (CH)	3.94 (t, *J* = 8.6 Hz)	75.1
5' (CH)	3.82 (m)	80.3
6' (CH_2)	4.24 (m)	63.3
6'-O-		
-1(CO)		173.6
-2(CH_2)	2.17-2.25 (m)	See below[a]
-3(CH)	2.01-2.07 (m)	See below[b]
-4(CH_3) x 2	0.94 (d, *J* = 6.6 Hz), 0.94 (d, *J* = 6.6 Hz)	See below[c]
[a] ^{13}C peak could not be unequivocally assigned among resonances at 43.4, 43.4, 43.5 and 43.6 ppm<br>[b] ^{13}C could not be unequivocally assigned among resonances at 26.1, 26.1 and 26.4 and 26.4 ppm<br>[c] ^{13}C could not be unequivocally assigned among resonances at 22.4, 22.5, 22.5 and 22.5 ppm		

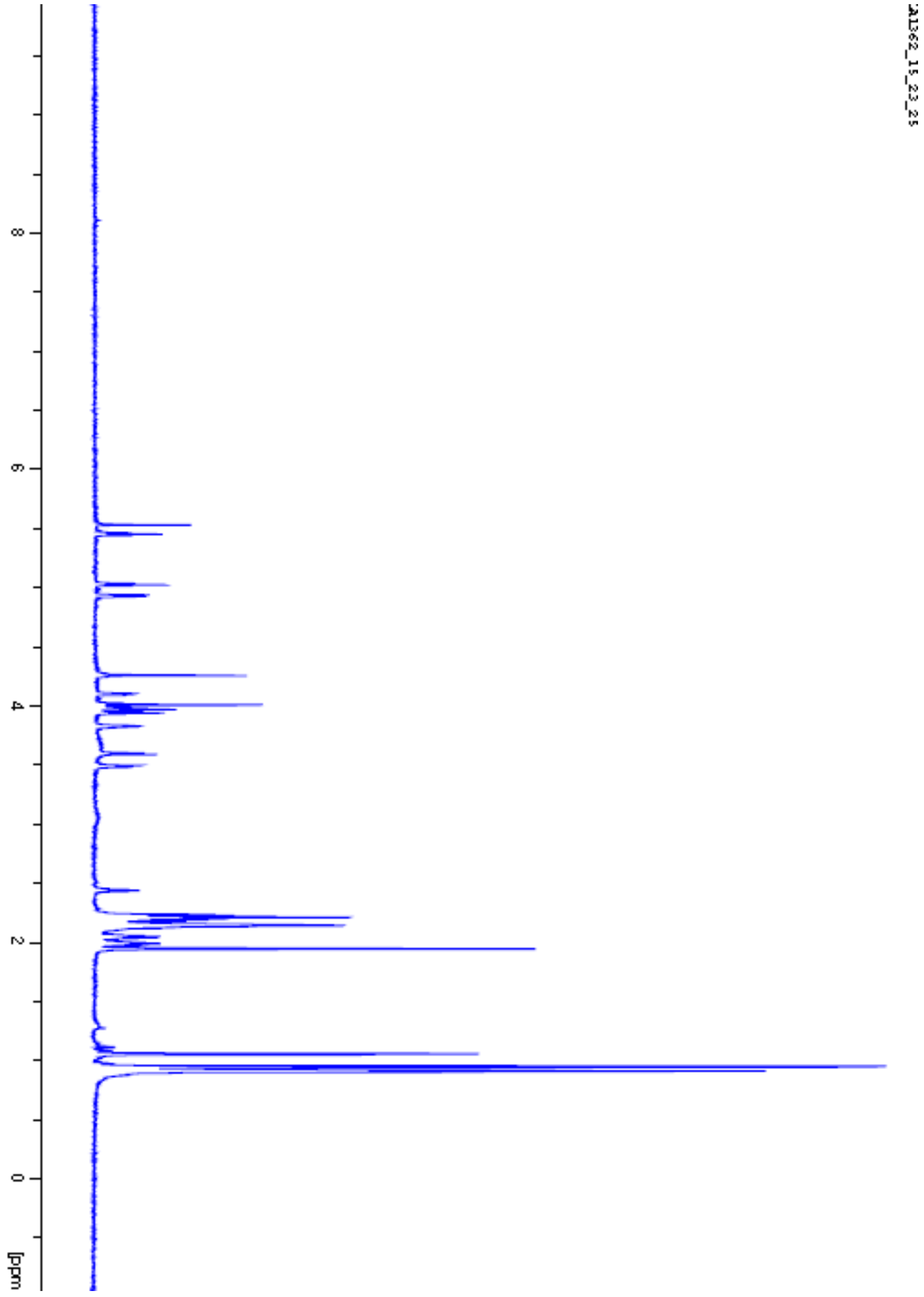
8
6
4
2
0
[ppm]

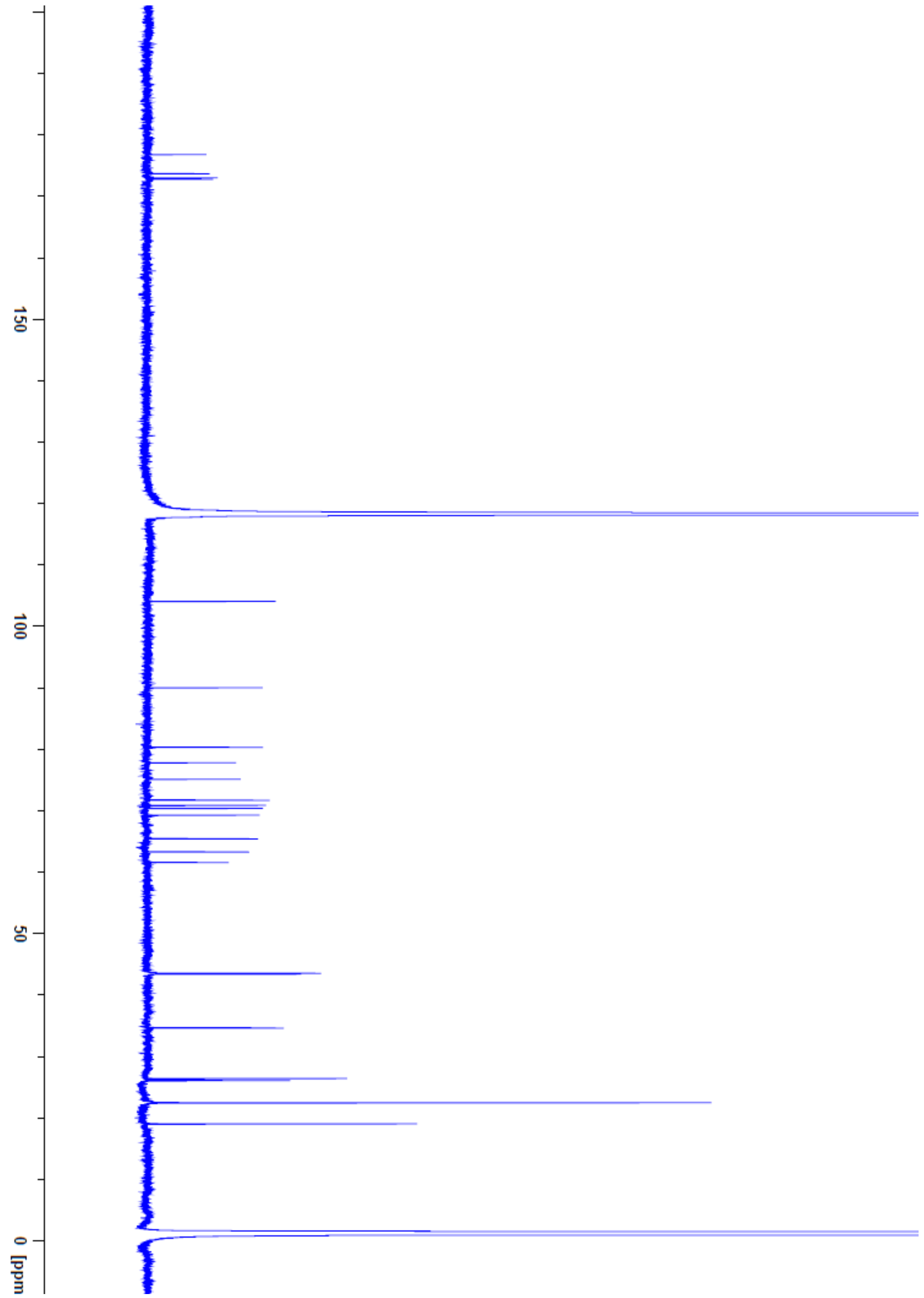
150
100
50
0 [ppm

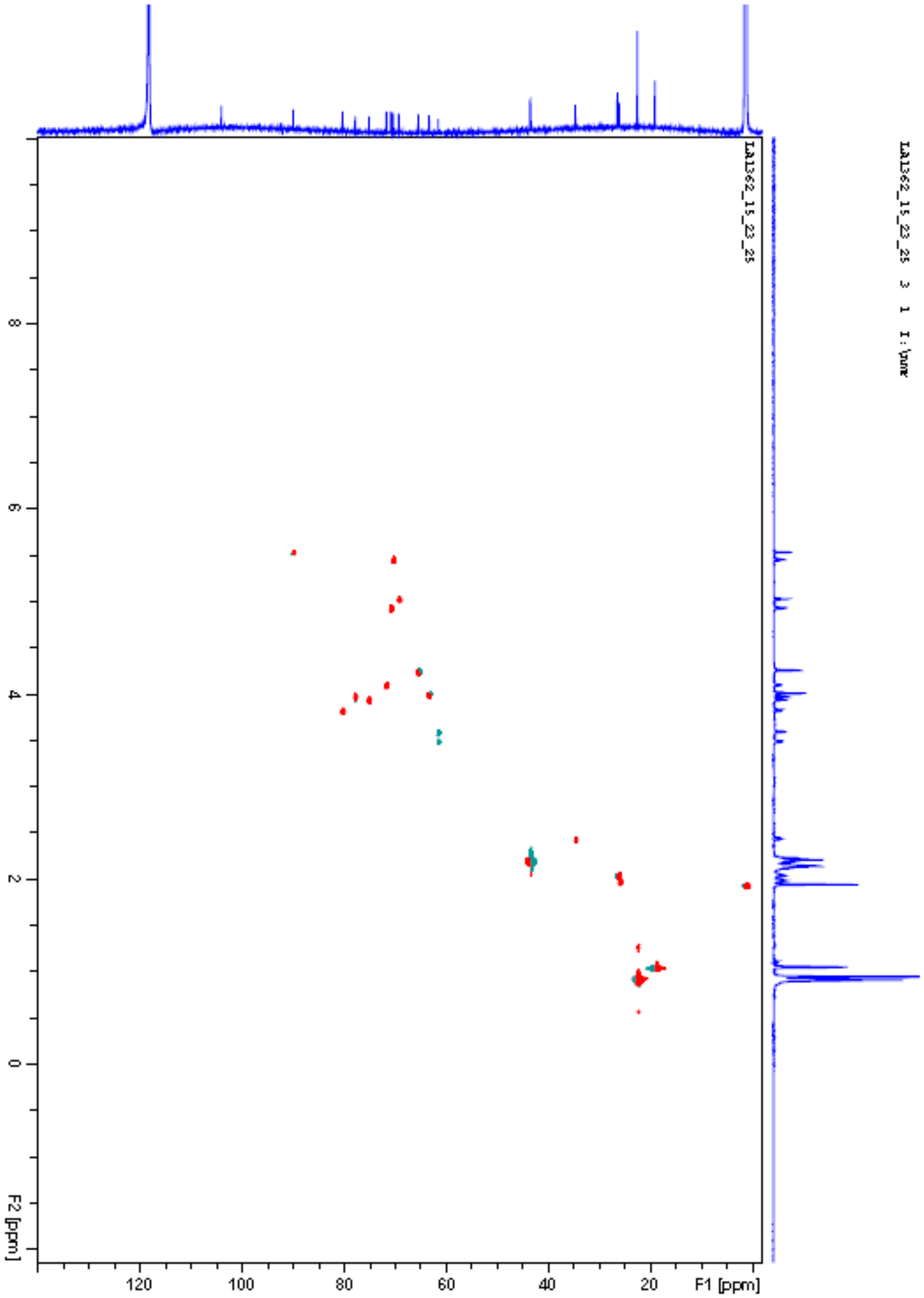

LA1362_15_23_25
F1 [ppm]
F2 [ppm]

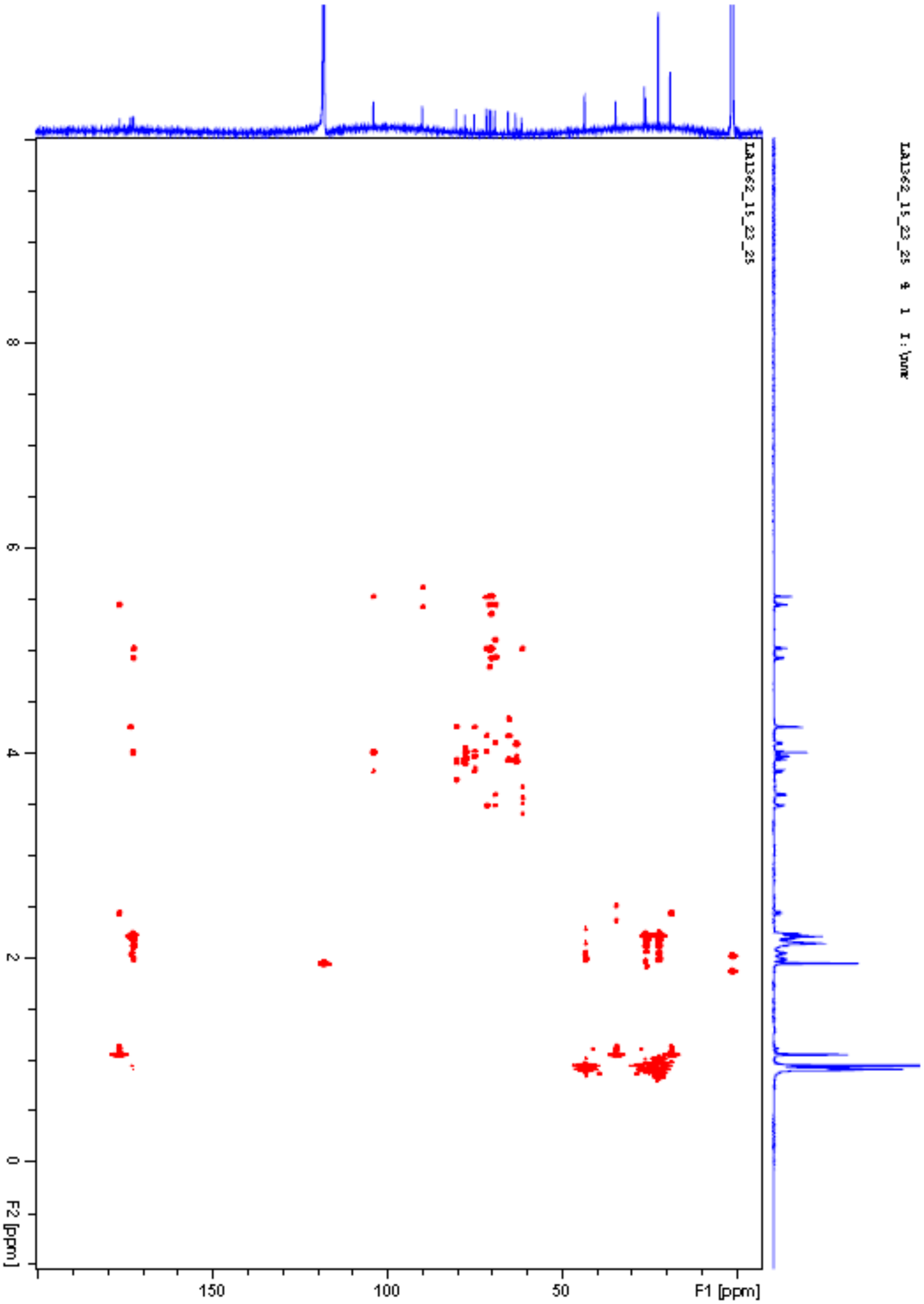
LA1362_15_23_25
F2 [ppm]
F1 [ppm]
150
100
50

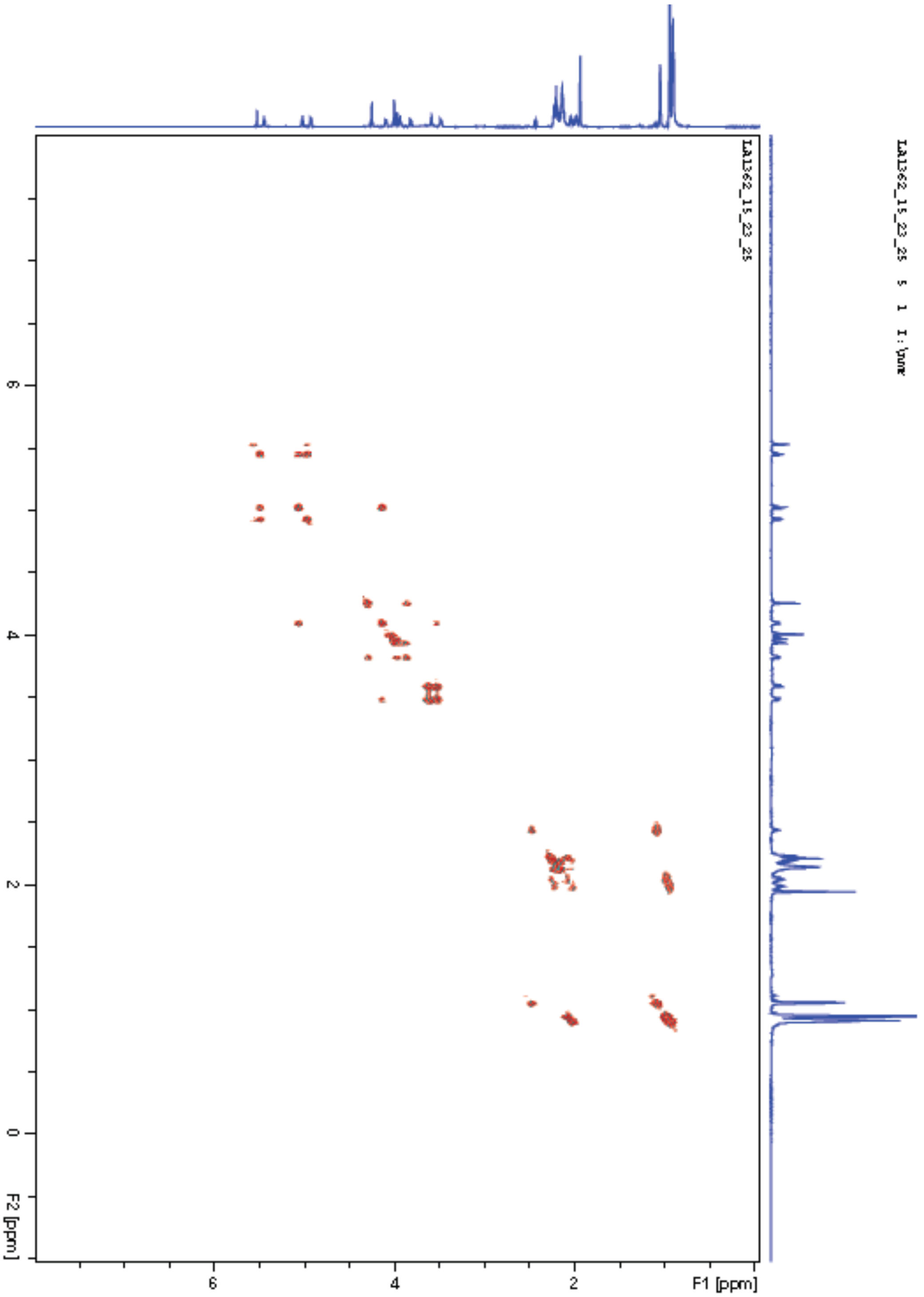
LA1362_15_23_25
6
4
2
0
F2 [ppm]
F1 [ppm]

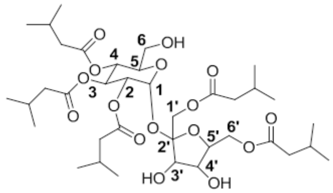	**S5:25[4]** (5,5,5,5,5)<br><br>**Purified from *S. habrochaites* LA1362**<br><br>**HRMS:** (ESI) *m/z* calcd for $C_{38}H_{63}O_{18}^-$ ([M+HCOO$^-$]): 807.4020, found: 807.4112<br><br>**Material recovered:** 2-3 mg<br><br>**NMR solvent**: CD_3CN<br><br>**InChI Key**: CYRJBUYQMOBNOS-PEIOGKDGSA-N	
Carbon # (group)	^{1}H (ppm)	^{13}C (ppm)
1(CH)	5.53 (d, J = 3.8 Hz)	89.9 ($^1J_{CH}$ = 177.3 Hz)
2(CH)	4.89 (dd, J = 10.4, 3.9 Hz)	70.9
2-O-		
-1(CO)		172.7
-2(CH)$_2$	2.08-2.24 (m)	See below[a]
-3(CH)	1.95-2.07 (m)	See below[b]
-4(CH$_3$) x 2	0.88-0.98 (m)	See below[c]
3(CH)	5.47(t, J = 10.0 Hz)	70.2
3-O-		
-1(CO)		172.7
-2(CH$_2$)	2.08-2.24 (m)	See below[a]
-3(CH)	1.95-2.07 (m)	See below[b]
-4(CH$_3$)x 2	0.88-0.98 (m)	See below[c]
4(CH)	5.00 (t, J = 10.0 Hz)	69.3
4-O		
-1(CO)		172.7
-2(CH$_2$)	2.08-2.24 (m)	See below[a]
-3(CH)	1.95-2.07 (m)	See below[b]
-4(CH$_3$) x 2	0.88-0.98 (m)	See below[c]

5(CH)	4.09 (m)	71.7
6(CH_2)	3.48 (dd, J = 12.3, 5.6 Hz), 3.59 (dd, J = 12.3, 2.0 Hz)	61.6
1' (CH_2)	4.0 (d, J = 4.9 Hz)	63.3
1'-O-		
-1(CO)		172.9
-2(CH_2)	2.08-2.24 (m)	See below[a]
-3(CH)	1.95-2.07 (m)	See below[b]
-4(CH_3) x 2	0.88-0.98 (m)	See below[c]
2' (C)		104.0
3' (CH)	3.97 (d, J = 8.9 Hz)	77.8
4' (CH)	3.93 (t, J = 8.6 Hz)	75.1
5' (CH)	3.82 (m)	80.3
6' (CH_2)	4.24 (m)	65.4
6'-O-		
-1(CO)		173.6
-2(CH_2)	2.21 (m)	See below[a]
-3(CH)	2.04 (m)	See below[b]
-4(CH_3) x 2	0.88-0.98 (m)	See below[c]

[a] ^{13}C peak could not be unequivocally assigned among resonances at 43.4, 43.5, 43.5, 43.5 and 43.5 ppm

[b] ^{13}C could not be unequivocally assigned among resonances at 26.1, 26.1, 26.1, 26.4 and 26.4 ppm

[c] ^{13}C could not be unequivocally assigned among resonances at 22.4, 22.5, 22.5, 22.5 and 22.5 ppm.

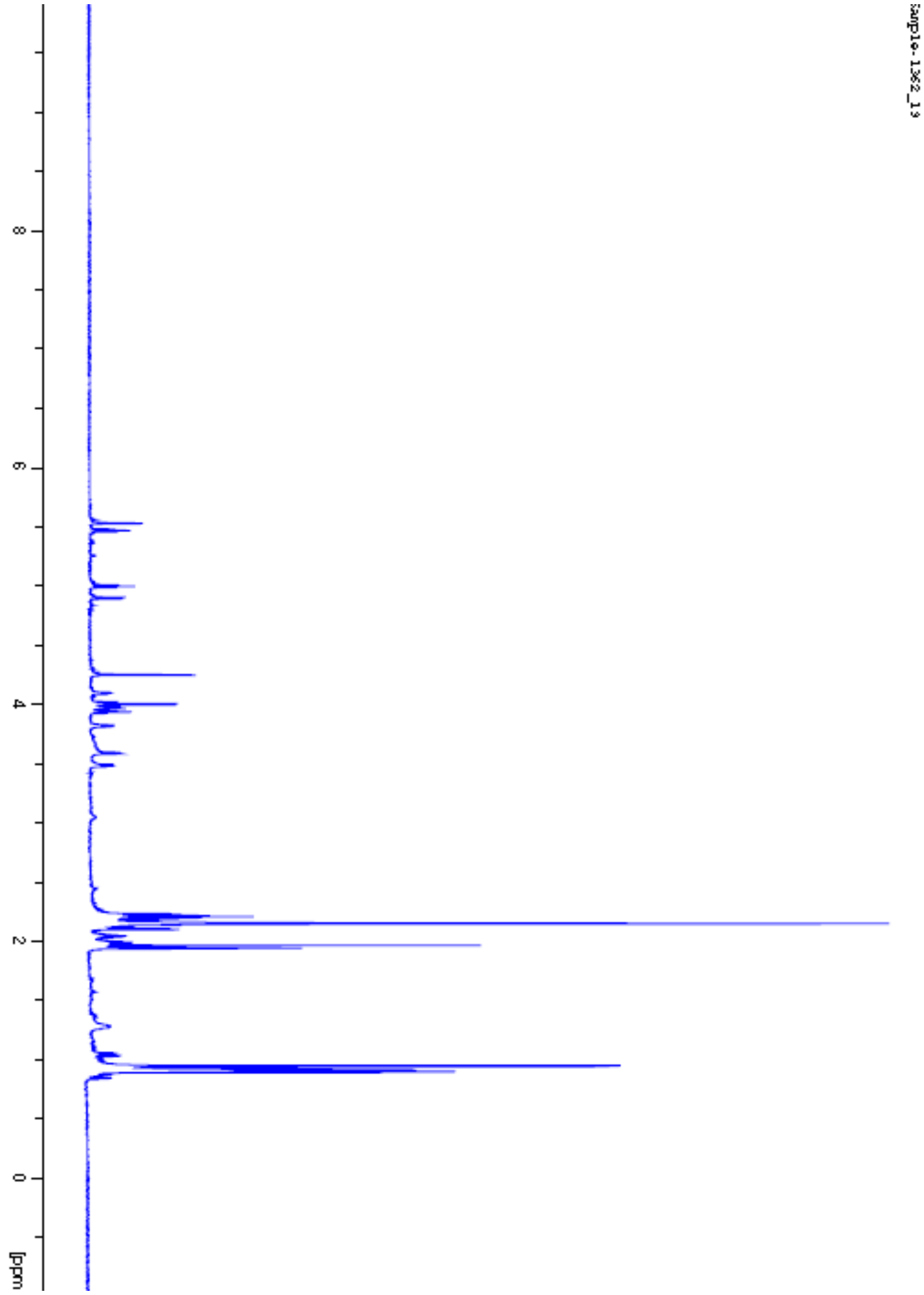
8
6
4
2
0
[ppm

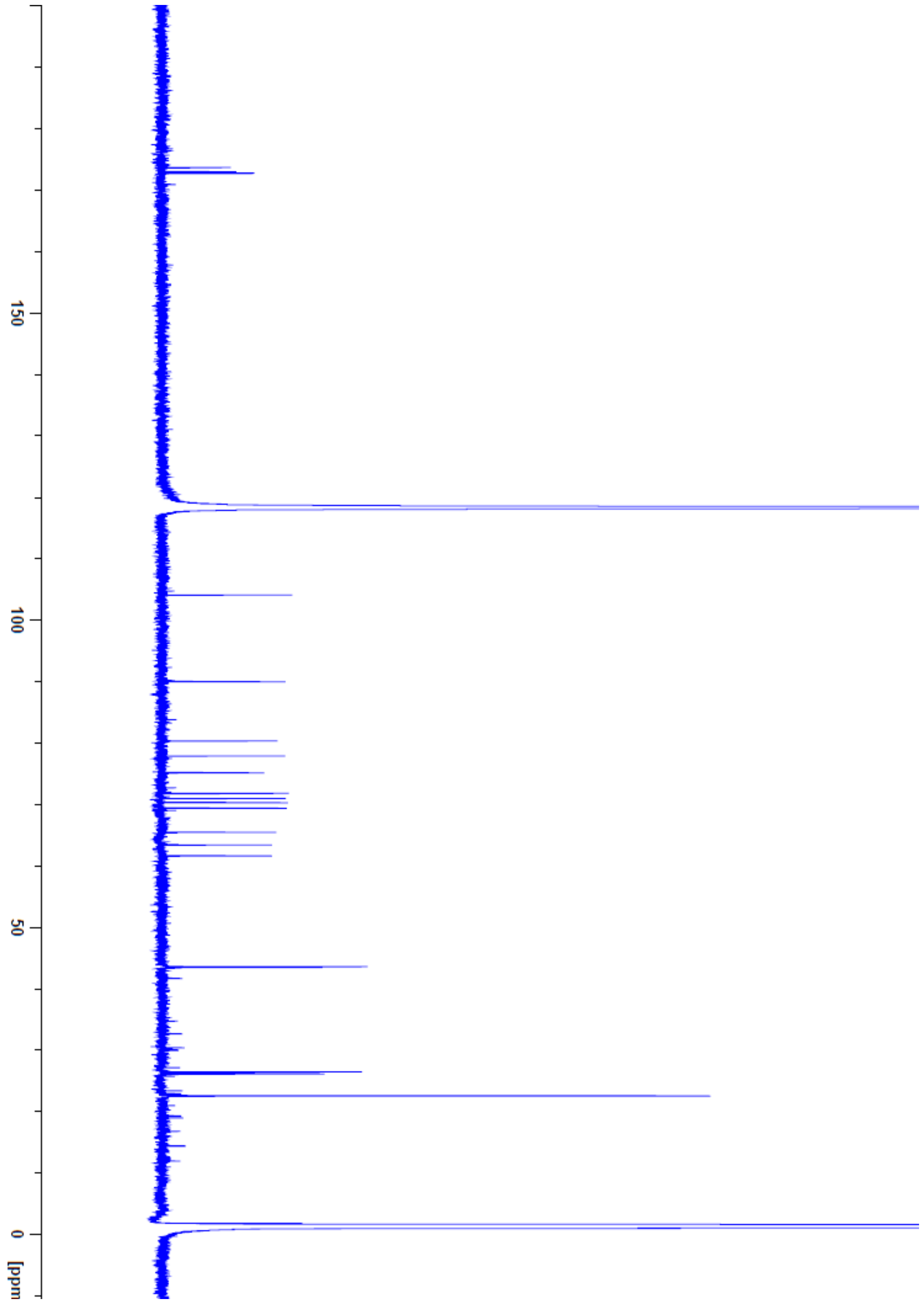
150
100
50
0
[ppm

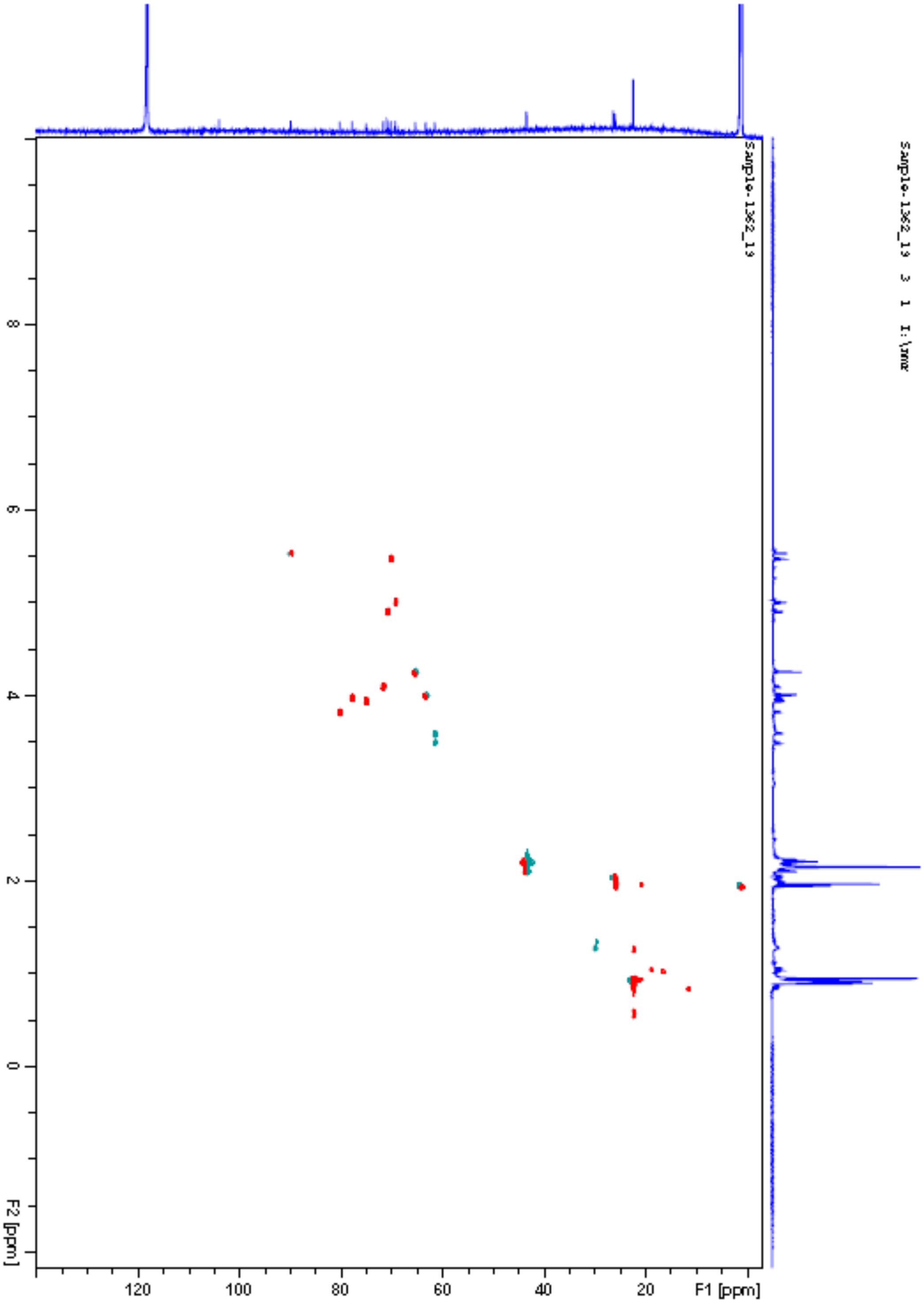
Sample-1362_19
Sample-1362_19 3 1 I:\nmr
F1 [ppm]
F2 [ppm]

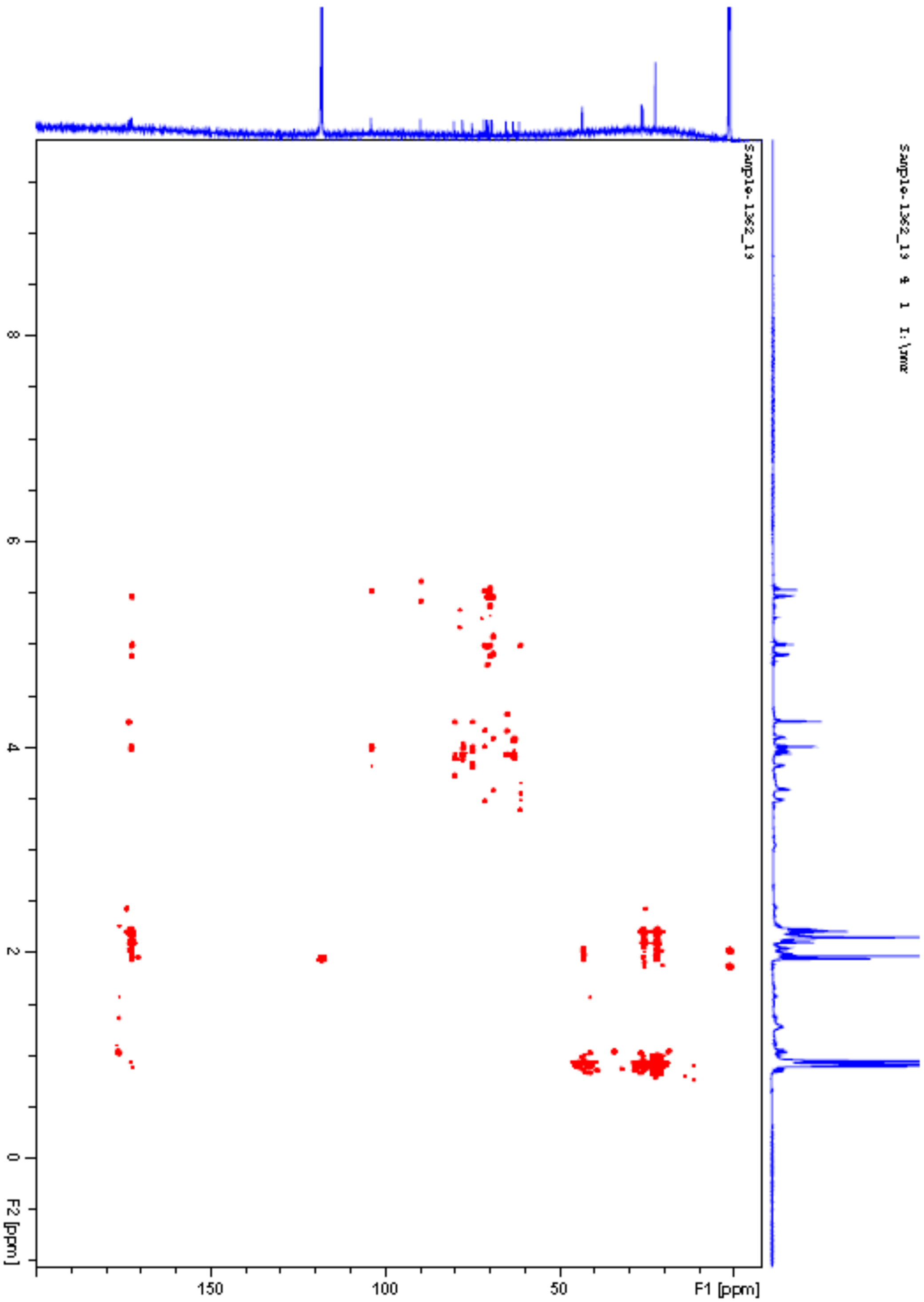
F1 [ppm]
F2 [ppm]

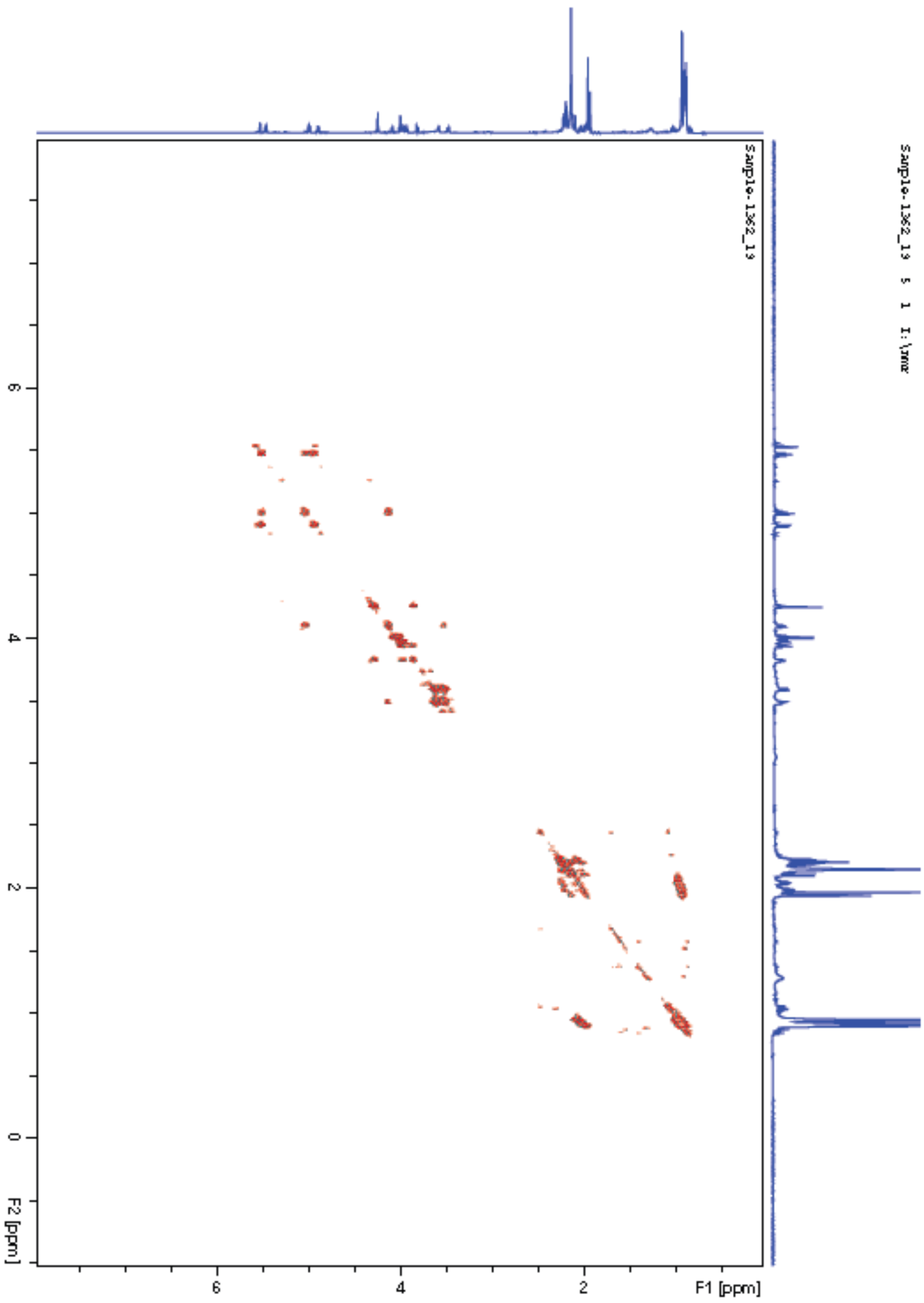
F2 [ppm]
F1 [ppm]
